# Redox–protonation landscape of indene-annulated perylenes: chemodivergent switching of multistate NIR chromophores

**DOI:** 10.1039/d6sc01695b

**Published:** 2026-06-02

**Authors:** Agata Wiencierz-Paś, Liliia Moshniaha, Piotr J. Chmielewski, Tadeusz Lis, Mateusz Waliczek, Ryota Kabe, Marcin Stępień

**Affiliations:** a Wydział Chemii, Uniwersytet Wrocławski Ul. F. Joliot-Curie 14 50-383 Wrocław Poland marcin.stepien@uwr.edu.pl; b Organic Optoelectronics Unit, Okinawa Institute of Science and Technology Graduate University 1919-1 Tancha, Onna-son Okinawa 904-0495 Japan

## Abstract

Indene-annulated perylenes merge cyclopentadiene-like benzylic acidity with the multielectron redox chemistry of rylene carbonyl scaffolds, creating discrete redox–protonation (“Pourbaix”) spaces in which electron transfer and acid–base processes become intrinsically coupled. Here we map the accessible states of doubly and singly pentannulated perylenediimides and perylene tetraesters using absorption/emission spectroscopy, electrochemistry and spectroelectrochemistry, and DFT analysis. Voltammograms of all derivatives show two quasi-reversible, largely core-centered reductions, yet the pentannulated tetraesters uniquely undergo electrochemically induced γ-deprotonation prior to core reduction, consistent with electrogenerated-base chemistry. Chemical stimulation reveals pronounced chemodivergence: potassium *tert*-butoxide readily produces deprotonated “dienolate” NIR chromophores in the pentannulated tetraester series, whereas the corresponding diimides first form perylene-centered radical anions/dianions that evolve into benzylic anions and higher multianions in a cation-dependent manner, with crown ether sequestering K^+^ and reshaping reaction trajectories. Strong reductants enable entry into highly reduced charge states, including an X-ray-verified salt of a pentannulated diimide trianion displaying diverse potassium binding motifs, underscoring the non-innocent role of ion pairing. Oxidation of deprotonated dianions furnishes weakly coupled neutral diradicaloids with NIR absorption. These results establish indene-annulated rylenes as multistate NIR chromophores whose experimentally accessible Pourbaix subsets can be programmed by redox bias, base strength, and cation coordination, with direct implications for organic electrochromism and rylene-based energy-storage chemistries.

## Abstract 2

Discrete organic Pourbaix maps show how redox bias, base strength, and cation binding program multistate NIR chromophores in indenyl-annulated perylenes.

## Graphical abstract

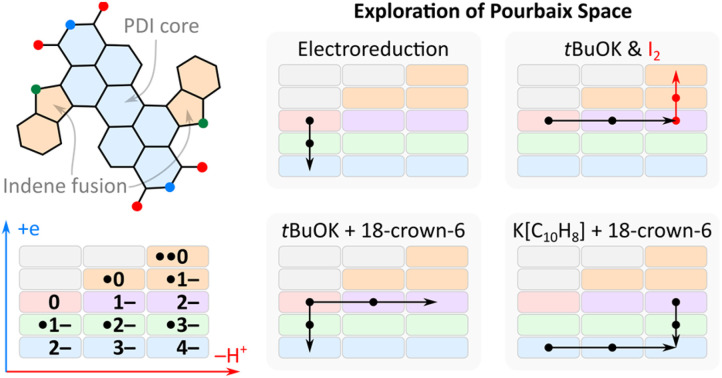

## Introduction

**Scheme 1:**
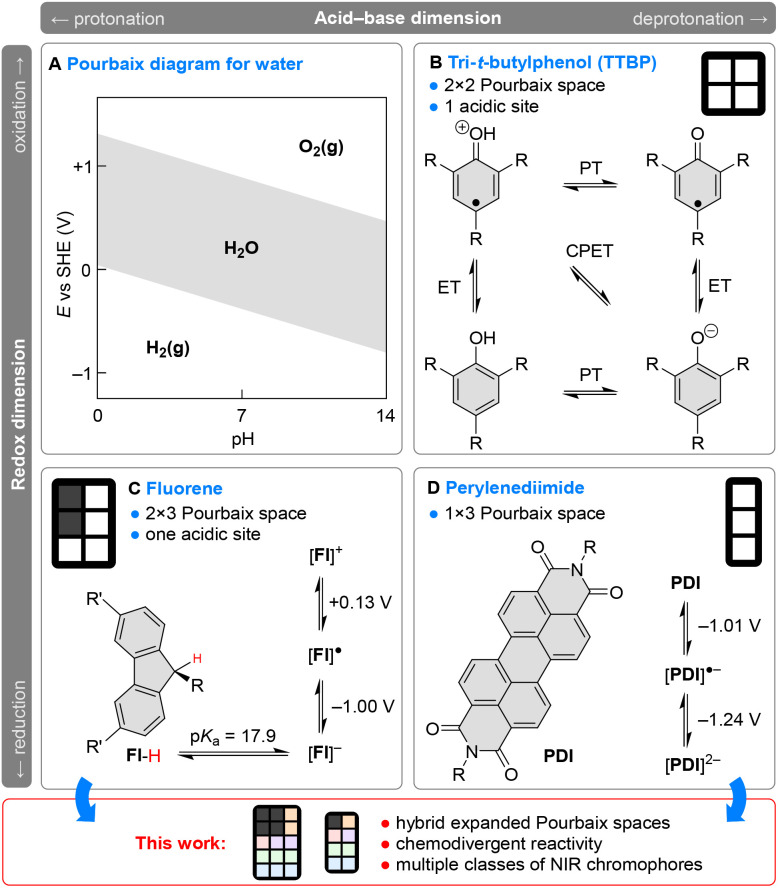
Examples of inorganic and organic Pourbaix spaces. (A) Pourbaix diagram for water.^9^ (B) A discrete 2 × 2 Pourbaix space of tri-*t*-butylphenol (TTBP, R = *t*-butyl).^15^ ET, electron transfer; PT, proton transfer; CPET, concerted proton–electron transfer. (C) Acidity of fluorene (p*K*_a_ in DMSO, R = Ph, R' = H) and redox chemistry of the fluorenyl radical (DCM solution, potentials relative to Fc/Fc^+^, *R* = 2,4,6-trichlorophenyl and R' = 4-(*tert*-butyl)phenyl).^16^ (D) Redox chemistry of perylenediimides (DCM solution, potentials relative to Fc/Fc^+^, *R* = 2,6-diisopropylphenyl = dipp).^17^ Boxes in panels B–D represent the respective Pourbaix spaces, with white and gray cells corresponding to accessible and inaccessible states.

Multistage organic redox systems underpin many emerging applications in charge and energy storage,^1–4^ organic semiconductors,^5,6^ and electrochromism.^7,8^ In most of these contexts, however, redox processes do not occur in isolation but compete or cooperate with acid–base chemistry. Classical potential–pH (Pourbaix) diagrams^9^ capture this two-dimensional thermodynamic landscape for inorganic materials and are now being extended to organic couples relevant to aqueous and non-aqueous redox-flow batteries and related technologies.^10–12^ In organic reactions, an analogous redox–protonation space is traversed by proton-coupled electron transfer (PCET) and related mechanisms, which play key roles in catalysis, energy conversion, and photochemistry (Scheme 1A–B).^13,14^ Mapping this coupled reaction space for functional organic motifs is therefore essential both for rational device design and for understanding unusual reactivity patterns.

Organic multiredox systems provide a particularly rich testing ground for such studies. Their behavior can often be rationalized in terms of aromaticity changes between different oxidation levels.^8^ In this framework, basic molecular building blocks with several accessible redox states serve as prototypes for more complex architectures. Fluorene (Fl–H, Scheme 1C) is a paradigmatic example: deprotonation at the benzylic position affords the strongly aromatic anion [Fl]^−^, which can be interconverted with the radical [Fl]˙ and the antiaromatic cation [Fl]^+^. This four-state manifold may be viewed as a subset of a 2 × 3 “Pourbaix space,” spanned by (i) the protonation state and (ii) the formal oxidation level.

**Scheme 2:**
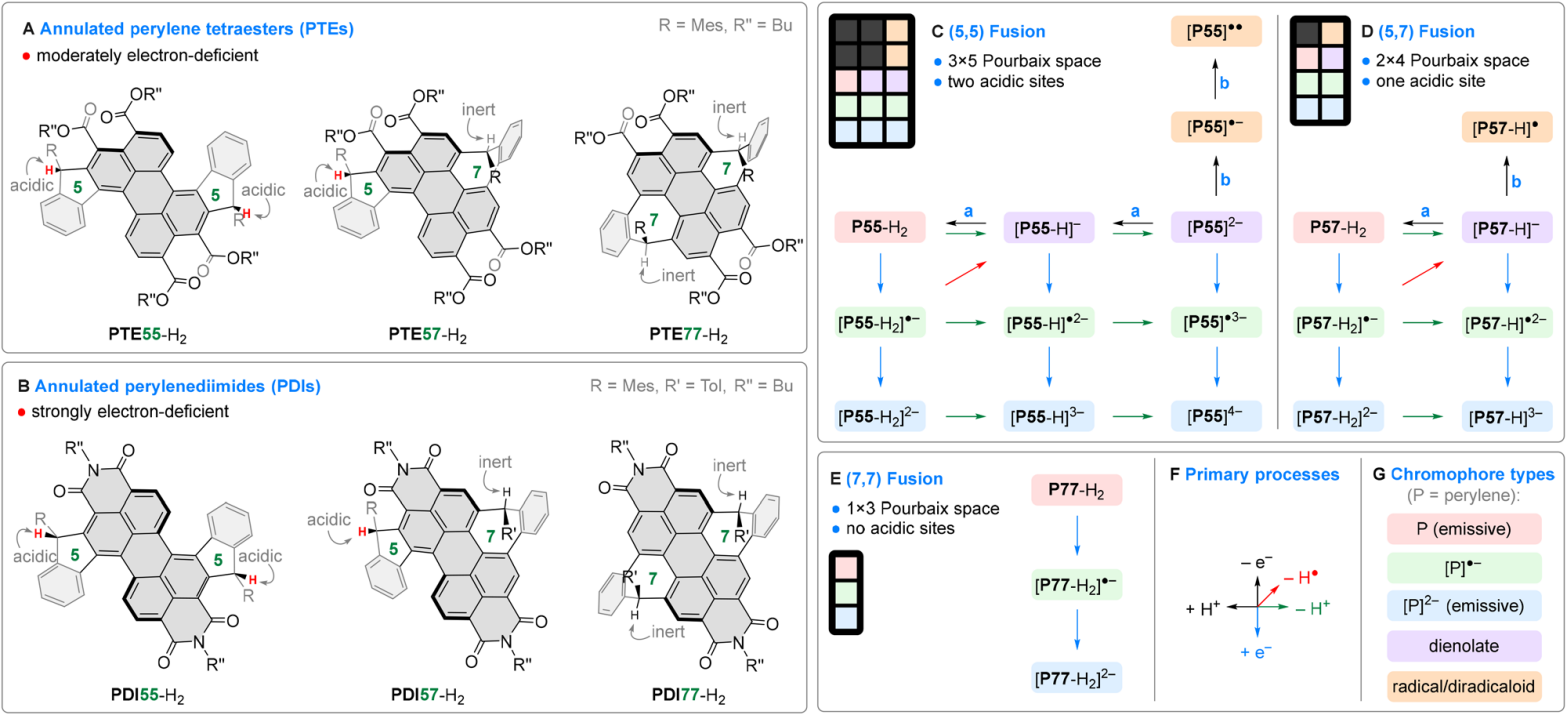
(A and B) Annulated perylene tetraesters and diimides explored in this work. (C–E) Redox–protonation manifolds observed, respectively, for (5,5), (5,7), and (7,7) ring-fusion patterns. Only experimentally accessible points in the Pourbaix space are shown. Unidirectional arrows indicate the experimentally observed direction of the transformation and do not imply strict irreversibility. Reagents and conditions (black arrows only): (a) H_2_O (or D_2_O), THF; (b) I_2_, THF. For reductions and deprotonations (colored arrows), see Scheme 3. Color coding of arrows and chromophores is defined in panels F and G, respectively. (F) Primary electron and proton transfer processes spanning the Pourbaix space. (G) Chromophore types (for discussion, see text).

Unlike conventional Pourbaix diagrams for inorganic aqueous systems (Scheme 2A), where the horizontal axis is pH and both axes (*E*, pH) are treated as continuous variables, the corresponding representation for molecular organic systems is often most practical as a discrete map of experimentally addressable states and their interconversions (Scheme 1B through D). Such mappings can, in principle, be made quantitative even in non-aqueous media if appropriate acidity scales are employed along with explicit redox potentials. A single node in such a map may encompass multiple microstates (tautomers, conformers, or spin states), but the Pourbaix representation remains useful as a coarse-grained guide to the accessible ET/PT landscape.

The Pourbaix-space concept can serve as a design heuristic for molecular systems in which proton transfer and electron transfer are both operative on comparable energy scales. Strategic integration of electron-rich and electron-poor motifs can expand the accessible redox range while retaining (or introducing) a responsive acid–base coordinate. The resulting systems can possess a larger number of addressable states than their constituent parts, and these states can reveal electronic properties that are not available in either component alone.

In this work, we explore this idea in electron-deficient indene-fused perylene derivatives, diimides (PDIs) and tetraesters (PTEs). The redox ranges of fluorenyl-based radicals overlap with those of PDIs (Scheme 1C and 1D), providing an opportunity to combine a prototypical benzylic acid–base motif with a multi-electron rylene redox manifold in a single platform. As shown below, this combination leads to unexpected chemodivergent behavior under nominally basic or reducing conditions, in which the same stimulus can select distinct sequences of proton- and electron-transfer events.

We use annulated PDI/PTE scaffolds spanning three fusion patterns as a platform to map a discrete redox–protonation landscape and the trajectories that connect its experimentally accessible states. A key finding is that, under the present conditions, potassium *tert*-butoxide can act not only as a Brønsted base but, in some cases, also as an effective reductant, thereby driving chemodivergent ET/PT pathways whose outcome depends on fusion patterns and ion pairing. The accessible states include NIR-active species, highly reduced multianions, and weakly coupled neutral diradicaloids, which we delineate using complementary spectroscopy, electrochemistry, X-ray crystallography, and theoretical calculations.

## Results and discussion

The bisannulated tetraesters PTE*mn*-H_2_ and the corresponding diimides PDI*mn*-H_2_ (where *mn* = 55, 57 or 77 denotes the corresponding (*m*,*n*) ring fusion pattern, Scheme 2) were obtained as described in our previous report, which focused on the unusual reactivity of the seven-membered rings.^18^ Under those strongly acidic conditions, the chemistry of indene units remained dormant, underscoring the orthogonal reactivities of the seven- and five-membered rings. In view of the electron-deficient nature of PDI and PTE cores, we anticipated that, when treated with bases and redox reagents, the pentannulated species might behave differently from other indenoarene systems, which are typically based on electron-rich aromatic motifs.^19,20^

We therefore set out to map the redox–protonation landscape of these annulated perylenes (Scheme 2), to identify accessible points in the formal Pourbaix space and determine how reagent choice selects trajectories among them. Our experiments were performed in THF under inert conditions and monitored by absorption, fluorescence, NMR, and ESR spectroscopies. In stepwise titrations, the reagent was added in small portions (*ca.* 0.1–0.2 equiv.), and after each addition, the sample was allowed to equilibrate for *ca.* 5 minutes prior to spectral measurements. In some experiments, however, a specific amount of the reagent (1 equiv. or more) was added, and the evolution of the reaction mixture was observed as a function of time.

### Chemodivergent reactions with *t*BuOK

**Scheme 3:**
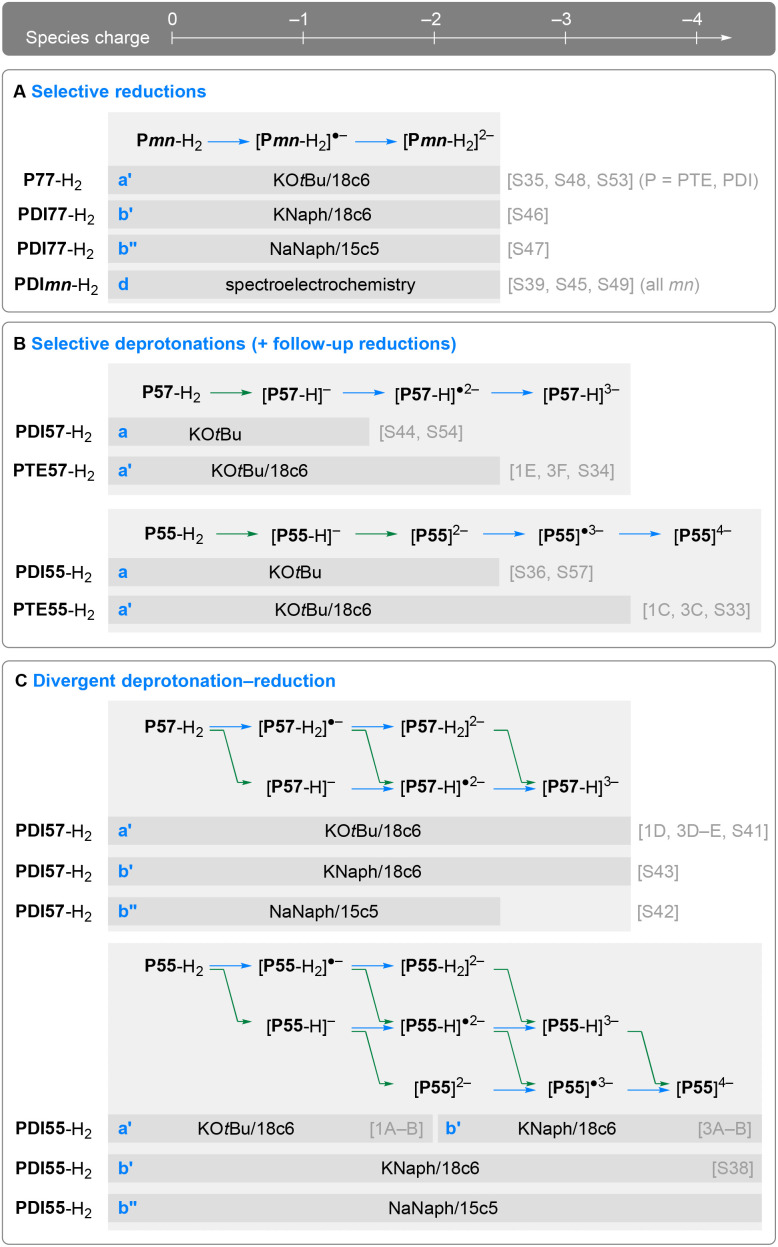
Selective and chemodivergent formation of anions observed for annulated perylenes PTE*mn*-H_2_ and PDI*mn*-H_2_ in UV–vis–NIR titrations. (a) *t*BuOK, THF; (a') *t*BuOK, 18c6, THF; (b') KNaph, 18c6, THF; (b") NaNaph, 15c5, THF. Gray bars denote charge ranges accessible with specific reagents. Speciation depends on the reagent (see text). P = PTE or PDI. Note that the horizontal axis (species charge) is not identical with the redox dimension of the Pourbaix space. References to figures are given in square brackets. Arrow colors correspond to those in Scheme 2F.

**Fig. 1:**
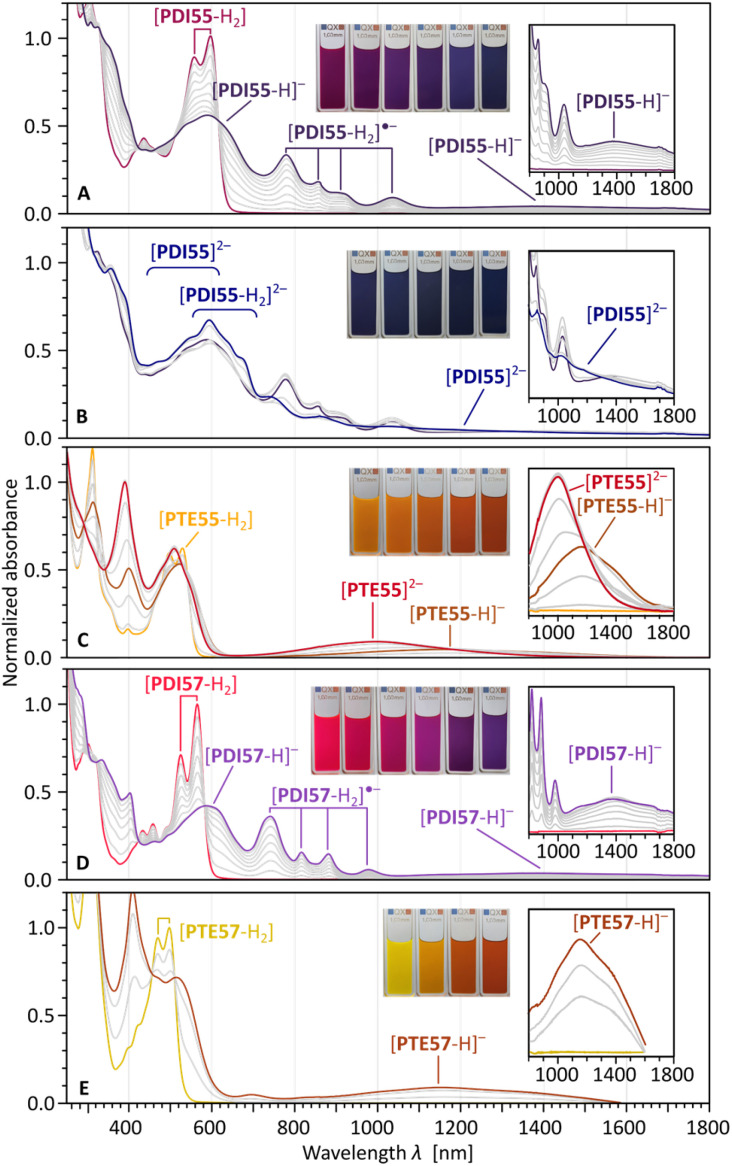
Titrations with *t*BuOK performed in THF for (5,5)- and (5,7)-fused perylenes: [PDI55-H_2_] (A and B; 0.1 equiv. steps, 50 equiv. 18c6, 5 min delays, ambient light); [PTE55-H_2_] (C); [PDI57-H_2_] (D); and [PTE57-H_2_] (E). In all cases, 18-crown-6 was added to the starting solution. In panels A, D, and E, absorbances were normalized against the initial spectrum. In panel B, absorbances were normalized against the initial spectrum in panel A. In panel C, absorbances were normalized against the spectrum of [PTE55]^2−^. Intermediate steps are shown in gray. For the corresponding TD-DFT simulations, see Fig. S96–S97.

Potassium *tert*-butoxide (*t*BuOK) is known to cleanly deprotonate many diindenoarenes^21,22^ and was thus chosen for titration experiments (conditions a, Scheme 3). In selected titrations, excess 18-crown-6 (18c6) was introduced as a coordinating additive to attenuate ion pairing (conditions a'). For PTE57-H_2_, the *t*BuOK/18c6 system yielded a one-step reaction (Fig. 1E). The resulting species, characterized by a broad NIR absorption, was interpreted as the corresponding deprotonated anion [PTE57-H]^−^ (*λ*_max_ = 1160 nm, *cf.* Table S2). Under the same conditions, PTE55-H_2_ underwent two consecutive deprotonations to give [PTE55-H]^−^ (*λ*_max_ = 1082 nm) and then [PTE55]^2−^ (*λ*_max_ = 996 nm, Fig. 1C). The broad NIR bands, reproduced by TD-DFT, likely reflect the charge-transfer character of these fused indenyl anions (Fig. S96).

Titrations carried out for the imide analogues produced strikingly different results. When treated with *t*BuOK in the presence of 50 equiv. of 18c6, PDI57-H_2_ yielded near-perfect isosbestic points (Fig. 1D). At first glance, this behavior suggested formation of a single dominant species; on closer inspection, however, we found that the reaction involved the concurrent formation of two species at a constant molar ratio.^23^ The characteristic sharp features were assigned to the radical anion [PDI57-H_2_]˙^−^, by comparison with spectroelectrochemical experiments (*vide infra*). Additional broad bands were also present, reminiscent of the deprotonated [PTE57-H]^−^ anion. We thus suspected that PDI57-H_2_ undergoes divergent reactivity toward *tert*-butoxide, with competing deprotonation and reduction producing [PDI57-H]^−^ and [PDI57-H_2_]˙^−^, respectively.

This hypothesis was confirmed in further experiments, which showed that product selectivity depends on the addition of crown ether and includes a kinetically controlled equilibration. In the presence of excess 18c6, *t*BuOK generated mainly the non-fluorescent radical anion [PDI57-H_2_]˙^−^ (Fig. S56). In contrast, in the absence of 18c6, stepwise addition of *t*BuOK afforded the also non-emissive deprotonated anion [PDI57-H]^−^ with high selectivity (*λ*_max_ = 1284 nm; Fig. S44). The switch in selectivity involves an equilibration step that becomes evident at sufficiently high dilution (∼0.05 mM). Upon adding *t*BuOK in the dark (Fig. S54), a mixture of [PDI57-H]^−^ and [PDI57-H_2_]˙^−^ formed within seconds and then evolved over ∼15 min toward higher [PDI57-H]^−^ concentration, followed by near-complete loss of [PDI57-H_2_]˙^−^ and emergence of the next reduced state [PDI57-H]˙^2−^ (see below). Remarkably, neither reduction nor deprotonation was observed when PDI57-H_2_ was treated with preformed [K(18c6)][O*t*Bu],^24^ obtained by co-dissolving equimolar *t*BuOK and 18c6 in THF (Fig. S55).

**Fig. 2:**
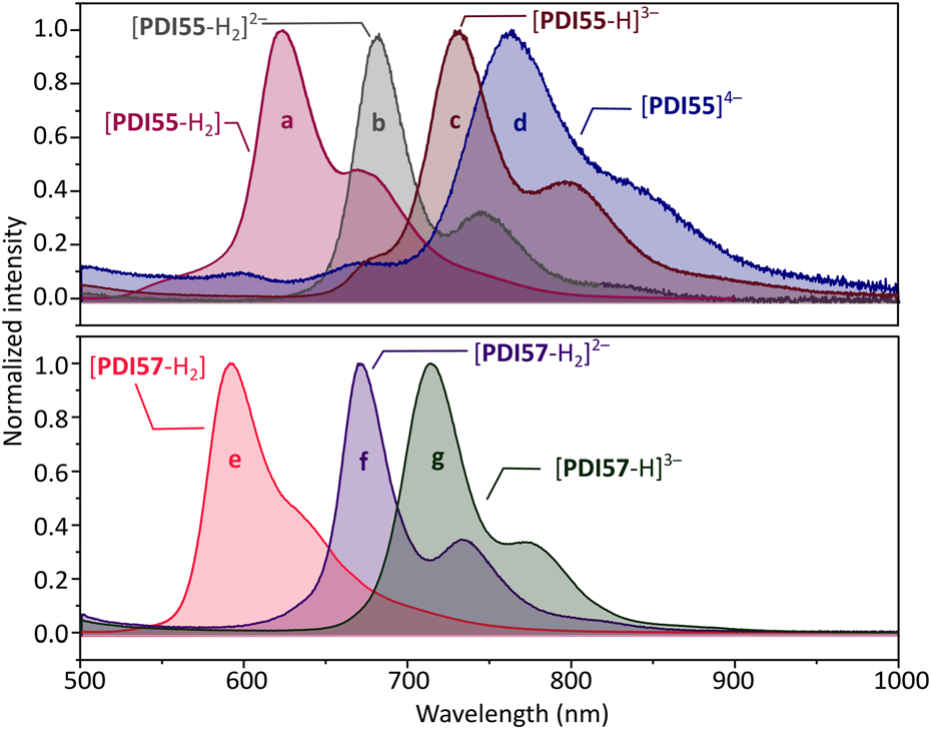
Fluorescence spectra of PDI55-H_2_, PDI57-H_2_, and their emissive reduced and deprotonated states (10 µM, THF).

A similar reactivity divergence was observed for PDI55-H_2_. A stepwise titration with *t*BuOK initially produced a mixture of [PDI55-H]^−^ and [PDI55-H_2_]˙^−^, showing no detectable fluorescence (Fig. 1A). Subsequently, products of further deprotonation and reduction were observed, *i.e.*, [PDI55]^2−^ and [PDI55-H_2_]^2−^ (Fig. 1B). The latter species produced a narrow red-shifted emission spectrum (*λ*_max_^em^ = 682 nm), detectable in the titration mixture using fluorescence spectroscopy (Fig. 2A). The mixed reduction–deprotonation product [PDI55-H]˙^2−^ may also form under these conditions, but its presence could not be confirmed in this experiment.

The (7,7)-fused systems appeared to have no acidic sites, prompting us to test whether they undergo clean electron transfer when titrated with an excess of *t*BuOK. In the presence of 18c6, PDI77-H_2_ gave spectra of [PDI77-H_2_]˙^−^ and [PDI77-H_2_]^2−^ (Fig. S48) that closely matched those obtained by spectroelectrochemical reduction. In contrast, in the absence of 18c6, the reduction stopped at [PDI77-H_2_]˙^−^ (5 equiv. of *t*BuOK, in the dark). When the resulting sample was irradiated with a 365 nm UV source, the radical anion was reduced further to [PDI77-H_2_]^2−^, indicating that photoinitiation may be required for *t*BuOK-mediated reductions occurring at lower redox potentials. Under ambient light, PTE77-H_2_ also reacted with *t*BuOK, yielding spectra similar, but not identical, to those of electrogenerated [PTE77-H_2_]˙^−^ and [PTE77-H_2_]^2−^ (Fig. S35 and S50). The outcome of these reactions depended on the rate of *t*BuOK addition (Fig. S53), implying a more complex behavior than that seen for PDI77-H_2_.

The progress of *t*BuOK-induced reactivity was also followed using low-temperature ^1^H NMR spectroscopy (THF-*d*_8_, 190 K, no added 18c6). The sharp spectra observed for [PTE55-H]^−^, [PTE55]^2−^, and [PDI57-H]^−^ confirmed the diamagnetic ground states of these ions, which were characterized using 2D correlation spectroscopy (Fig. S10–S30). Deprotonation sites were additionally identified by D_2_O quenching of [PTE55]^2−^ and [PDI57-H]^−^, which produced, respectively, PTE55-D_2_ and PDI57-DH, deuterated at the sp^3^ carbons of the 5-membered rings (Fig. S8 and S9). These data do not establish full chemoselectivity, since any paramagnetic products formed would not be observed by NMR.

### Mechanistic discussion

In organic synthesis, *t*BuOK is primarily used as a non-nucleophilic base with strongly solvent-dependent basicity,^25^ but it also acts as a reducing agent in a range of synthetically relevant processes.^26–31^ In those reactions, *t*BuOK behaves as a moderately strong reductant (*E*_p,ox_ = +0.10 V *vs.* SCE in DMF, *i.e. ca.* −0.45 V *vs.* Fc/Fc^+^),^32^ acting *via* an inner-sphere mechanism, likely involving coordination of potassium cations. The ensuing *tert*-butoxyl radical *t*BuO˙ can cleave into acetone and a methyl radical,^33^ or abstract a hydrogen atom from the solvent, which should be the fastest process in THF.^34^ The irreversible character of this chemistry may explain why *t*BuOK can reduce species with significantly more negative redox potentials, such as PDIs and PTEs. While some *t*BuOK-mediated redox processes are photoinduced,^35–39^ we found that the first electron transfer to PDI*mn*-H_2_ systems occurs in the absence of light.

**Scheme 4:**
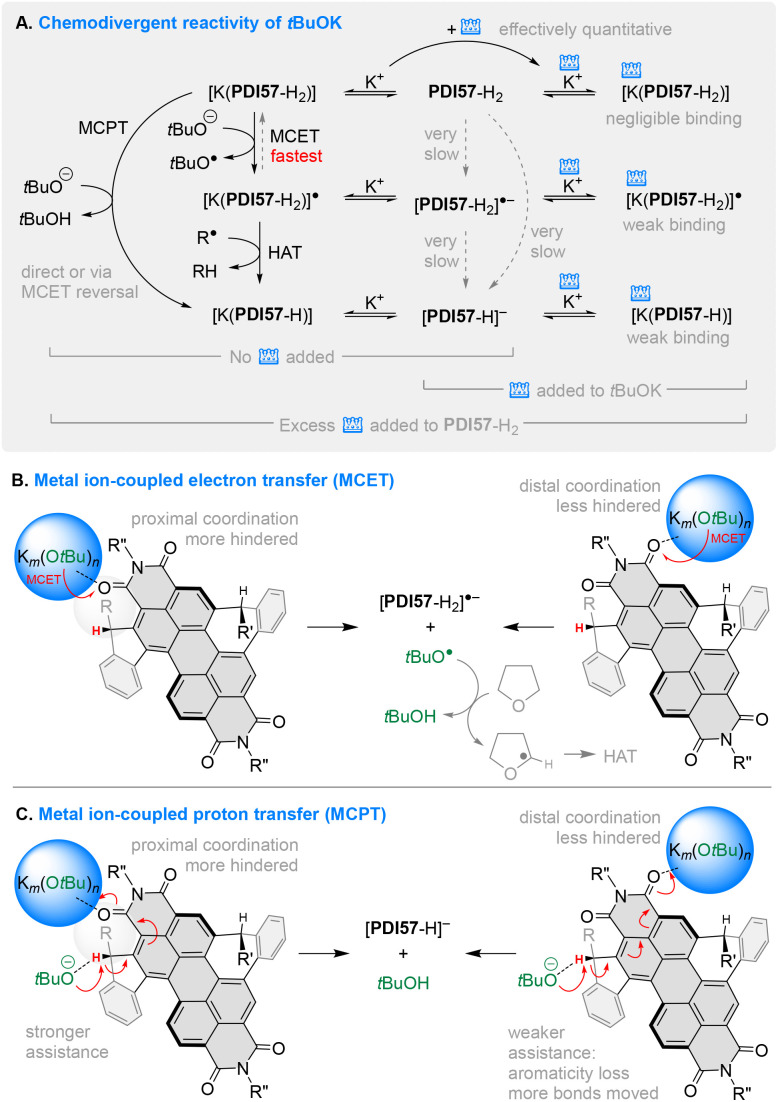
Proposed assistance of potassium cations in competing electron- and proton-transfer reactions, illustrated for PDI57-H_2_. In panel A, “K^+^” indicates any aggregated complex capable of coordinating to the carbonyl group. The crown symbol denotes 18-crown-6. Unidirectional arrows indicate the experimentally observed direction of the transformation and do not imply strict irreversibility.

The dual role of potassium *tert*-butoxide in the above reactions and the unexpected effects of 18-crown-6 on the selectivity point to a complex scenario illustrated for the key case of PDI57-H_2_ in Scheme 4. In the absence of 18c6, the sequence apparently starts with rapid coordination of a potassium cation to one of the imide carbonyls. Indirect evidence for such coordination came from voltammetry, which showed that reduction onsets of PDI57-H_2_ are shifted anodically by the addition of KPF_6_, an effect that is reversed by the subsequent addition of 18c6 (Fig. S87 and Table S4). Since *t*BuOK is tetrameric in THF,^40–42^ it is expected to bind to the weakly coordinating imide (or ester) functions in an aggregated state (denoted K_*m*_(O*t*Bu)_*n*_ in Scheme 4, where *m*, *n* ≤ 4). Under these conditions, metal ion-coupled electron transfer (MCET,^43^Scheme 4B) between the potassium-bound *t*BuO^−^ and PDI57-H_2_, yielding *t*BuO˙ and [PDI57-H_2_]˙^−^, respectively, is apparently the fastest process, as it may require minimal deaggregation of *t*BuOK.

The formation of the deprotonated anion [PDI57-H]^−^ can occur along one of two different routes. The follow-up conversion of [PDI57-H_2_]˙^−^ into [PDI57-H]^−^, observed in our kinetic experiments, implies that hydrogen atom transfer (HAT) may be a relevant process, though slower than MCET, at least after the initial ratio of reduced and deprotonated states has been established. HAT could be induced by any reactive radical present in the mixture (R˙, Scheme 4A), *e.g.*, the nascent tBuO˙ or the subsequently formed α-tetrahydrofuranyl radical (Scheme 4B). Given that some [PDI57-H]^−^ was already observed in the first UV–vis–NIR spectrum (recorded *ca.* 5 s after the addition of *t*BuOK), direct deprotonation of PDI57-H_2_ operating in parallel with MCET cannot be ruled out as an alternative to HAT. Since deprotonation of PDI57-H_2_ is unusually suppressed by 18c6 (see below), the direct formation of [PDI57-H]^−^ should involve metal ion-coupled proton transfer (MCPT, Scheme 4C), assisted by potassium binding similarly to the initial MCET step. Stronger assistance should be induced by coordination to the proximate carbonyl, which is, however, more sterically hindered by the fused indene subunit. It is also conceivable that the initially formed [PDI57-H_2_]˙^−^ slowly reverts to the neutral PDI57-H_2_, which is then deprotonated to [PDI57-H]^−^ in the MCPT step.

The lack of reactivity of PDI57-H_2_ toward the preformed [K(18c6)][OtBu] complex confirms that MCET is the only route for electron transfer under these conditions. While the coordination of [K(18c6)]^+^ cations to imide carbonyls is generally feasible, as illustrated by the crystal structure of [K(18c6)]_3_[PDI57-H] (see below), it is apparently negligible for neutral PDIs, switching off the assistance needed for MCET. The inefficiency of direct deprotonation is more surprising, in view of the well-known basicity enhancements caused by crown ethers.^44,45^ The effect is more likely kinetic than thermodynamic, given that the [PDI57-H]^−^ anion is accessible *via* the MCET + HAT route. Divergent deprotonations and reductions observed when excess 18c6 was added to the titrated solutions show that the deaggregation of *t*BuOK and the formation of [K(18c6)]^+^ are slower than the MCET (and possibly MCPT) process.

The seven-membered rings in P57-H_2_ and P77-H_2_ systems (P = PTE, PDI) contain benzylic C(sp^3^)–H bonds that could, in principle, be deprotonated with strong bases. Such reactivity was, however, not observed in either PDI57-H_2_ or PTE57-H_2_: the low acidity of tropilidene rings likely reflects (a) the weaker conjugation of the resulting carbanionic position with the imide groups, in comparison with the five-membered rings, (b) the equatorial positioning of the C(sp^3^)–H bond and (c) the thermodynamic instability of the antiaromatic cycloheptatrienyl anion,^46,47^ which should form upon proton removal. Similarly, PDI77-H_2_ is selectively reduced by *t*BuOK to the corresponding radical anion and dianion. For PTE77-H_2_, its more negative reduction potentials may rationalize the lower selectivity of *t*BuOK-induced reductions.

### Electrochemistry

Cyclic and differential pulse voltammetry show that each PTE*mn*-H_2_ and PDI*mn*-H_2_ supports two quasi-reversible one-electron reductions and an irreversible one-electron oxidation (for complete discussion, see the SI). Relative to the diimides, reductions of tetraesters are shifted cathodically by *ca.* 0.5–0.6 V, consistent with largely core-centered redox chemistry slightly perturbed by the fused subunits. Thus, voltammetry primarily probes the redox coordinate at fixed protonation within the Pourbaix space. Spectroelectrochemistry revealed more diverse behavior; as expected, the PDI*mn*-H_2_ series was reduced cleanly to PDI-centered radical anions and dianions, with absorption and emission spectra resembling those of fusion-free PDIs,^48–52^ indicating minimal chemical involvement of the annulated periphery. The tetraesters showed more varied behavior: while direct reduction was observed for PTE77-H_2_, the indene-fused PTE55-H_2_ and PTE57-H_2_ responded to cathodic bias by first undergoing partial deprotonation, to ultimately produce the expected radical anions and dianions. The initial deprotonation is tentatively attributed to the superoxide anion [O_2_]˙^−^,^53^ formed by reduction of dioxygen traces, which acts as an electrogenerated base (EGB).^54^

### Deep reductions

**Fig. 3:**
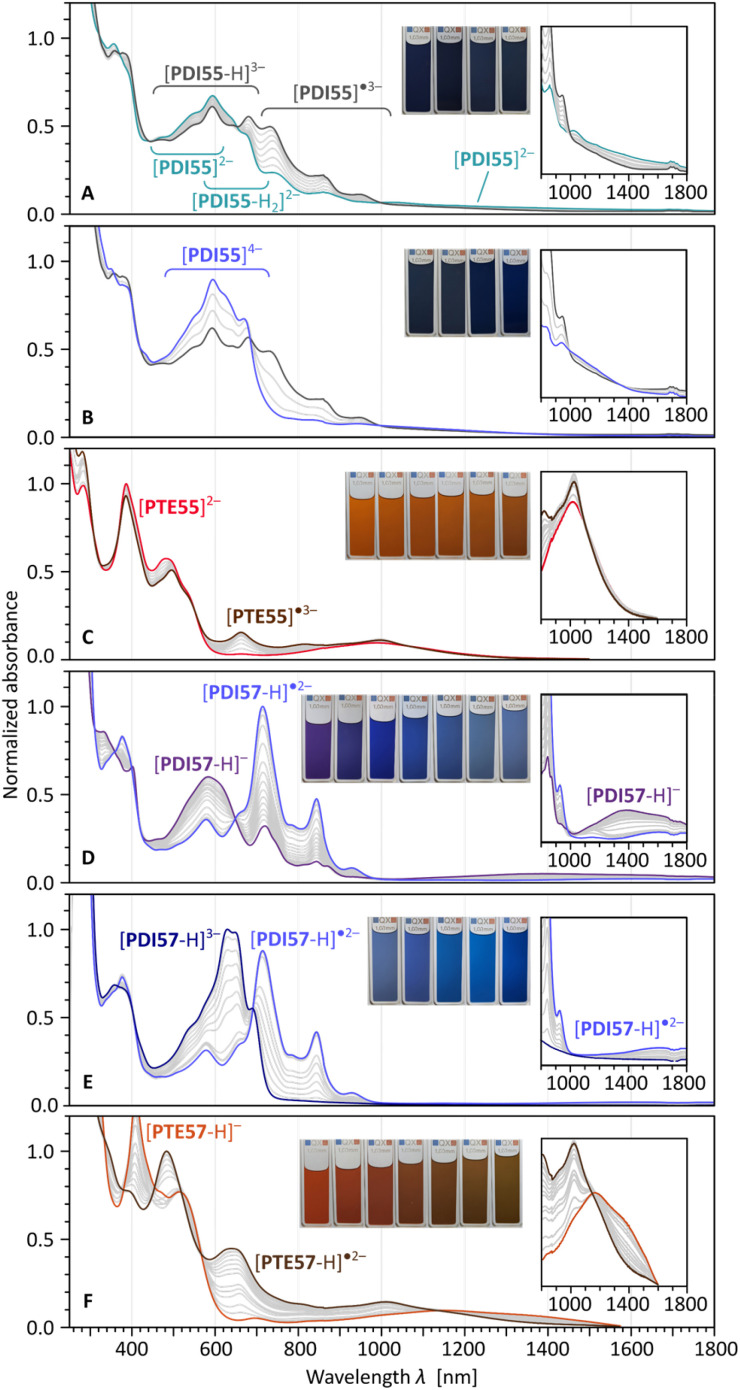
Reductive titrations of deprotonated (5,5)- and (5,7)-fused anions: [PDI55]^2−^ (A and B, excess KNaph)*;* [PTE55]^2−^ (C, excess *t*BuOK); [PDI57-H]^−^ (D–E, excess *t*BuOK); [PTE57-H]^−^ (F, excess *t*BuOK). In all cases, 18-crown-6 was added to the starting solution. Titrations are continued from those shown in Fig. 1 (note the change of the titrant in A and B), and the spectra are normalized accordingly. Intermediate steps are shown in gray. For the corresponding TD-DFT simulations, see Fig. S100–S103.

Pushing the (5,5)- and (5,7)-fused systems to more reducing conditions exposed additional, state-selective steps beyond the initial *t*BuOK responses (Scheme 3, conditions a', and Fig. 3). With excess *t*BuOK, each tetraester deprotonation product underwent one further transformation consistent with one-electron reduction: [PTE57-H]^−^ to [PTE57-H]˙^2−^ and [PTE55]^2−^ to [PTE55]˙^3−^. In the corresponding PDI57 manifold, the initially formed [PDI57-H]^−^/[PDI57-H_2_]˙^−^ mixture was driven first toward [PDI57-H]^−^ (*via* consumption of [PDI57-H_2_]˙^−^) and then reduced stepwise to [PDI57-H]˙^2−^ and [PDI57-H]^3−^. The trianion assignment is supported by fluorescence (Fig. 2): its emission resembles that of [PDI57-H_2_]^2−^, consistent with a similar emissive core, but is red-shifted (*λ*_max_^em^ = 714 nm *vs.* 683 nm, 635 cm^−1^), in line with the higher overall charge.

Access to the fully reduced PDI55 tetraanion required a stronger reductant and highlighted the multiplicity of PT/ET routes. Starting from the dianion mixture generated under conditions a′ ([PDI55]^2−^ and [PDI55-H_2_]^2−^), subsequent titration with potassium naphthalenide (KNaph, conditions b′) produced a final spectrum dominated by a structured absorption band in the 500–750 nm region, assigned to [PDI55]^4−^ based on its NIR emission (*λ*^em^_max_ = 762 nm) and its pronounced red shift relative to [PDI55-H_2_]^2−^. Several distinct sequences can connect the dianions to the tetraanion by interleaving proton- and electron-transfer steps (Scheme 3C). In the titrated mixture, [PDI55-H]^3−^ was identified by its emission at *λ*^em^_max_ = 730 nm (Fig. 2A and S69), while additional NIR absorptions above ∼700 nm, resembling those of [PDI57-H]˙^2−^, were attributed to [PDI55]˙^3−^, consistent with TD-DFT predictions of a small electronic gap for this state.

The same endpoint can also be reached directly from the neutral precursor, but with a different balance of pathways that depends on the reductant and countercation. Direct titration of PDI55-H_2_ with KNaph in the presence of excess 18c6 (conditions b') qualitatively reproduced the chemodivergent behavior of the two-step a'/b' protocol (Fig. S38): the ET route *via* [PDI55-H_2_]˙^−^ and [PDI55-H_2_]^2−^ was more prominent, yet the system still proceeded through [PDI55-H]^3−^ and [PDI55]˙^3−^ before converging to pure [PDI55]^4−^. An analogous KNaph/18c6 titration of PDI57-H_2_ likewise reached [PDI57-H]^3−^ and showed a similar preference for the ET pathway. Switching to sodium naphthalenide with 15-crown-5 (NaNaph/15c5, conditions b") produced qualitative countercation-dependent differences. For PDI55-H_2_, trianionic states were effectively unobservable, consistent with direct conversion of dianions to the tetraanion. For PDI57-H_2_, the reaction led selectively to [PDI57-H_2_]^2−^. The small early contribution of [PDI57-H]^−^, which disappeared at later stages of the titration, indicates that a deprotonated intermediate is formed transiently but does not persist, implying that reprotonation can occur along the pathway leading to [PDI57-H_2_]^2−^.

**Fig. 4:**
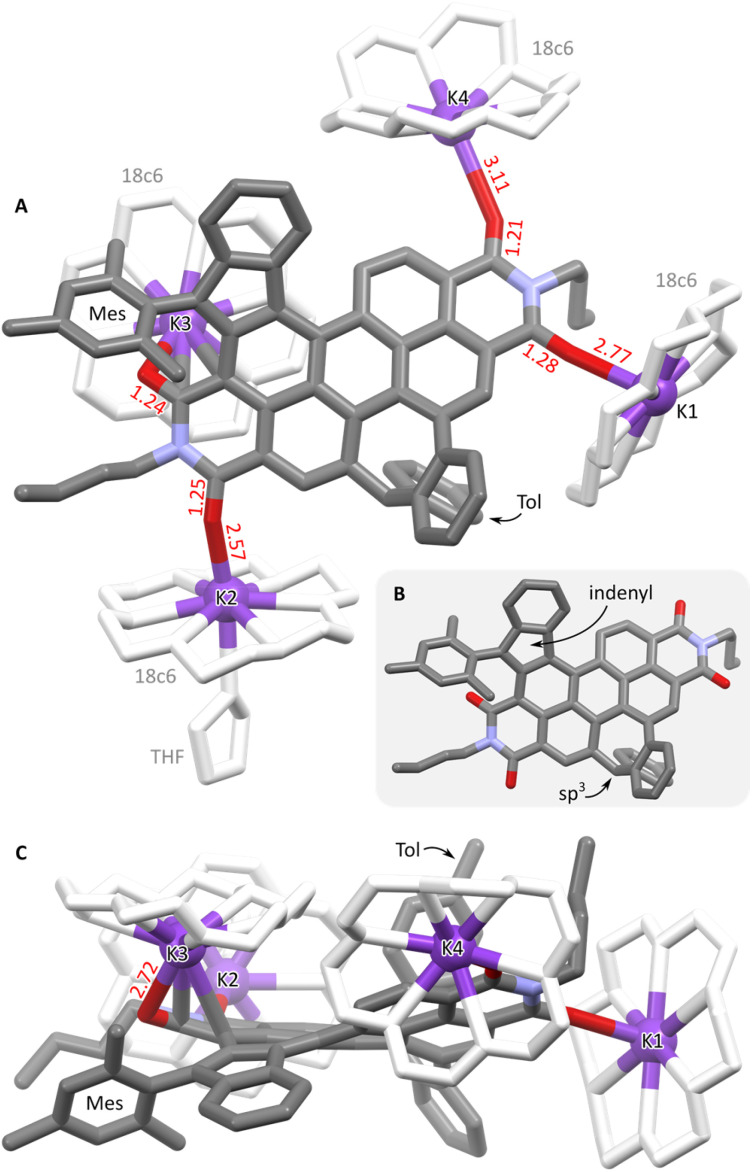
Structure and coordination mode of [PDI57-H]^3−^ as determined by X-ray diffraction analysis of [K(18c6)]_3_[PDI57-H]. Potassium cations are shown as purple spheres, and ether ligands are shown in white in panels A (top view) and C (side view). Panel B shows the uncoordinated trianion. Hydrogen atoms, disordered sites, and uncoordinated ether molecules are omitted for clarity. Bond esds are in the 0.01–0.02 Å range.

Weakly diffracting single crystals were grown from a reduced THF/18c6 solution containing [PDI57-H]^3−^. The resulting dataset is incomplete and of limited quality, but it still affords a chemically informative solid-state model of the [K(18c6)]_3_[PDI57-H] salt, revealing several distinct potassium-coordination modes (Fig. 4). Because the metric precision is limited, the structural discussion below is restricted to qualitative ion-pairing features and approximate coordination geometry. In particular, the solid-state model supports formation of the trianion [PDI57-H]^3−^, as evidenced by full potassium-cation occupancy in the crystal lattice. Four distinct potassium sites (K1–K4, Fig. 4) were refined, each with a markedly different crystallographic environment. Each K^+^ is chelated by 18-crown-6 (18c6). K1, K2, and K4 also bind to an imide oxygen of [PDI57-H]^3−^ to form slightly bent C–O⋯K contacts: K1 bridges two [PDI57-H]^3−^ moieties, whereas K4 is positionally disordered between two trianions with half occupancy at each site. By contrast, K2 is fully occupied, with its coordination sphere completed by a THF ligand. The fourth imide carbonyl of [PDI57-H]^3−^ is sterically encumbered by the Mes substituent and instead forms a distinctive *η*^3^ interaction with K3. A closely related binding mode was reported for the enolate complex [(H_2_C=CHO)K(18c6)].^55^ The K3···OCC distances in [PDI57-H]^3−^ (2.73, 3.17, and 3.34 Å) are, within the limited precision of the present dataset, slightly longer than those reported for [(H_2_C=CHO)K(18c6)] (2.67, 3.03, and 3.20 Å),^55^ consistent with greater charge delocalization in [PDI57-H]^3−^ and/or steric effects. The observed strong coordination of [K(18c6)]^+^ to the trianion helps rationalize the distinct deep-reduction behavior of NaNaph *vs.* KNaph, which may stem from differing affinities of [K(18c6)]^+^ and [Na(15c5)]^+^ for the reduced anions in solution.

### Oxidations

**Fig. 5:**
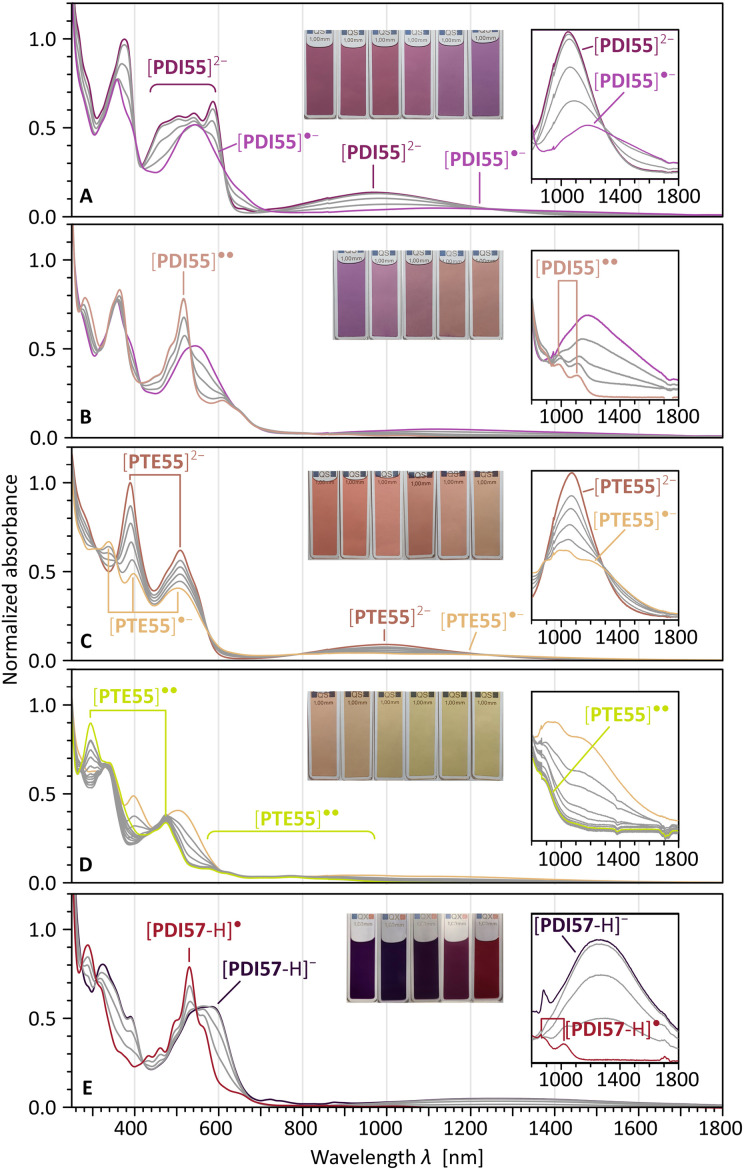
Oxidative titrations (I_2_ in THF, no 18-crown-6 added) carried out for (5,5)- and (5,7)-fused anions: [PDI55]^2−^ (A and B); [PTE55]^2−^ (C and D); [PDI57-H]^−^ (E). In each titration, absorbances were normalized against the spectrum of the initial P*mn*-H_2_ system prior to deprotonation (Fig. S32, S40 and S44). Intermediate steps are shown in gray. For the corresponding TD-DFT simulations, see Fig. S104–S107.

**Fig. 6:**
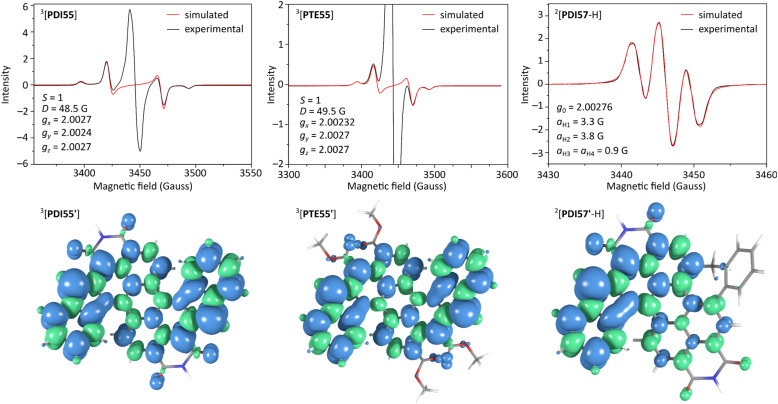
Top: ESR spectra of ^3^[PDI55]^0^ (frozen toluene, 109 K), ^3^[PTE55]^0^ (frozen toluene, 107 K), and ^2^[PDI57-H]^0^ (toluene, 300 K). Bottom: calculated spin density distributions of ^3^[PDI55']^0^ and ^2^[PDI57'-H]^0^ (R=R' = H), and ^3^[PTE55']^0^ (R = H, R' = Me; CAM-B3LYP-GD3BJ/6-31G(d,p); isovalue: 0.001 a.u.). For additional spin density plots, see Fig. S94 and S95.

Chemical oxidation provided access to the upper region of the redox–protonation landscape and revealed open-shell neutral and radical states. When [PDI57-H]^−^ was treated with 0.5 equiv. of I_2_ in THF, the broad NIR absorption of the anion disappeared and was replaced by several weak NIR features at 772, 876, and 1028 nm (Fig. 5E). A low-temperature ^1^H NMR titration (THF-*d*_8_, 190 K) produced extremely broadened spectra, consistent with a paramagnetic product. Accordingly, the oxidation product was assigned as the neutral radical [PDI57-H]˙. This assignment was corroborated by ESR spectroscopy, which revealed a doublet species with *g*_0_ = 2.00276 and hyperfine couplings attributable to four protons (Fig. 6). DFT calculations on [PDI57'-H]˙ indicated an unequal spin distribution over the PDI core, with significant delocalization onto the *ortho*-fused benzene ring. This asymmetry is expected for the intrinsically unsymmetrical (5,7)-fused framework, in which the unpaired spin is primarily associated with the indenyl-derived segment and extends into the fused PDI π-system. Protons on the *ortho*-fused benzene ring are therefore the most plausible origin of the observed hyperfine pattern.

The dianions [PDI55]^2−^ and [PTE55]^2−^ each underwent two sequential iodine oxidations, consistent with stepwise conversion to the corresponding radical anions and then to neutral species. In both cases, the addition of 0.5 equiv. of I_2_ generated a spectrum with a characteristically broad, weak NIR band, assigned to [PDI55]˙^−^ and [PTE55]˙^−^, respectively (Fig. 5A and C). A further 0.5 equiv. of I_2_ produced a second spectral change consistent with formation of the neutral states [PDI55]˙˙ and [PTE55]˙˙ (Fig. 5B and D). The diradicaloid character of these neutral species is suggested by the similarity between the spectrum of [PDI55]˙˙ and that of the neutral radical [PDI57'-H]˙ (Fig. 5B and E).

Further evidence for an open-shell neutral state was obtained for [PTE55]˙˙ by *in situ*^1^H NMR oxidation of [PTE55]^2−^ (Fig. S31). Addition of 1 equiv. of I_2_ caused complete broadening of all aromatic signals even at 190 K, consistent with an open-shell system with a small absolute singlet–triplet gap. ESR spectroscopy of [PTE55]˙˙ (107 K, frozen toluene; Fig. 6) displayed a triplet-like zero-field splitting pattern. Within the point-dipole approximation,^56^ the simulated *D* value of 49.5 G corresponds to an effective spin–spin distance of 7.21 Å, substantially shorter than the 9.77 Å separation between the indenyl carbons in the DFT-optimized structure of ^3^[PTE55']˙˙. This discrepancy is consistent with extensive delocalization of spin density into the perylene core, as found by DFT (Fig. 6). The analogous species [PDI55]˙˙, prepared and characterized similarly, gave a nearly identical *D* value (48.5 G). Although the imide groups in ^3^[PDI55']˙˙ appear more involved in spin delocalization than the ester groups in ^3^[PTE55']˙˙ (Fig. 6), [PDI55]˙˙ likewise shows a marked difference between the point-dipole distance (7.26 Å) and the geometrical DFT estimate (9.91 Å for ^3^[PDI55']˙˙; see below), consistent with closely related open-shell characteristics. Variable-temperature EPR measurements in frozen toluene showed only a weak temperature dependence of the doubly integrated intensity of the triplet region. Because the spectra contain an overlapping doublet contribution from a coexisting radical component, these data do not permit a reliable extraction of Δ*E*_ST_. They are nevertheless consistent with a small singlet–triplet gap, in line with the low-temperature NMR observations and the DFT analysis.

### Computational analysis

**Fig. 7:**
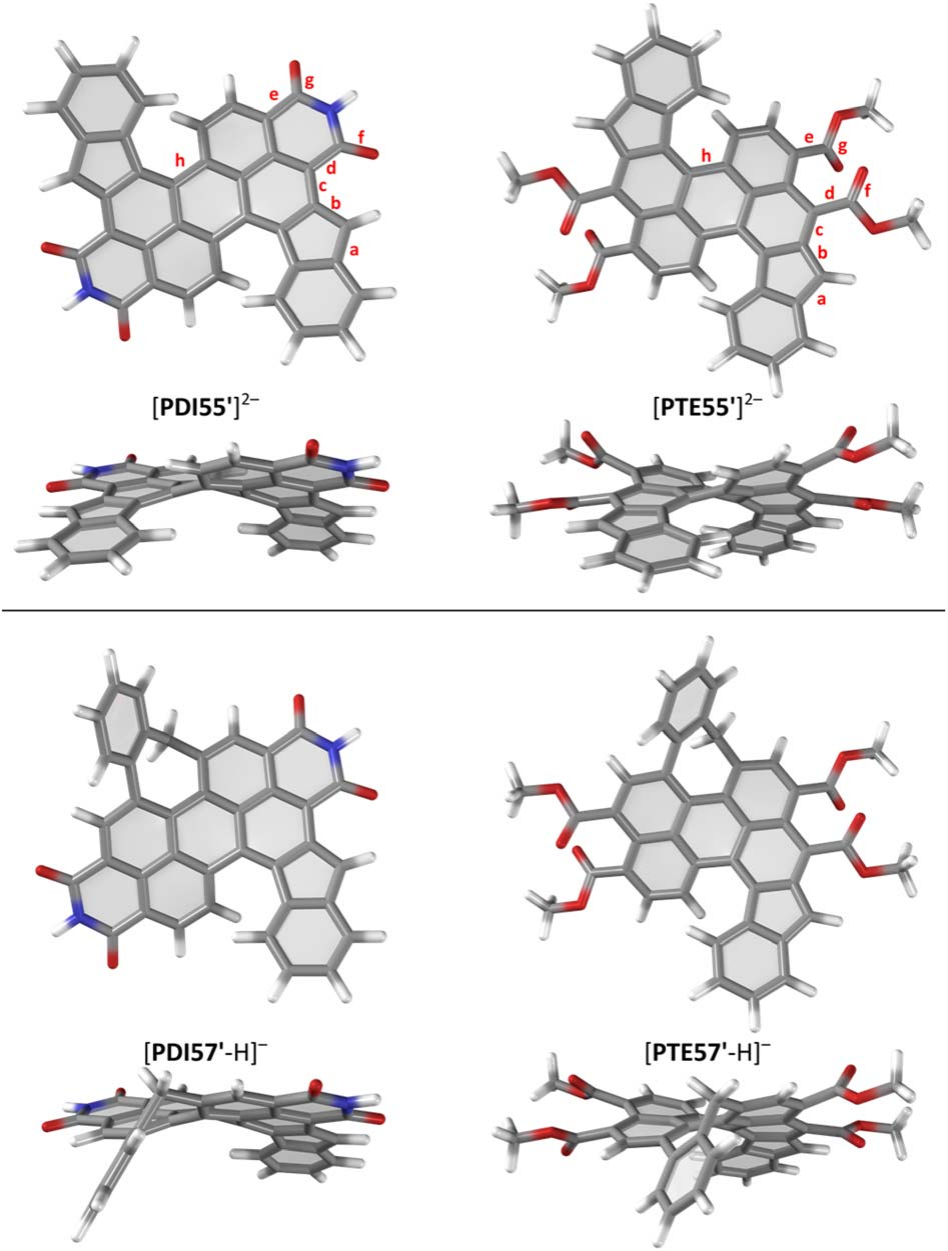
DFT-optimized geometries of the deprotonated anions [PDI55']^2−^, [PTE55']^2−^, [PDI57'-H]^−^, and [PTE57'-H]^−^ (CAM-B3LYP-GD3BJ/6-31G(d,p)). For each structure, the top and side views are shown above and below the label, respectively. Key bonding distances in the dianions are labeled in red.

Structures of the (5,5)- and (5,7)-fused anions were investigated by density functional theory (DFT) at the CAM-B3LYP-GD3BJ/6-31G(d,p) level, with simplified substitution patterns denoted PDI*mn*' (R = R' = H, R" = H) and PTE*mn*' (R = R' = H, R" = Me, Fig. 7). In all cases, the π system is twisted along the long axis of the perylene substructure, either to relieve steric repulsions with the indenyl fragments or to accommodate the nonplanarity of the tropilidene rings. In the PTE series, the ester groups are sterically congested and rotated out of plane, weakening conjugation with the aromatic core and thereby diminishing the electron-deficient character of PTEs relative to their PDI counterparts. These conformational features persist across other oxidation levels of PDI55' and PTE55', discussed below.

Relative to the neutral [PDI55'-H_2_] and [PTE55'-H_2_], the dianions [PDI55']^2−^ and [PTE55']^2−^ show shortened d and e bonds and elongated f and g bonds, with the largest changes occurring closer to the five-membered ring (Fig. 7 and Table S7). This behavior is consistent with partial delocalization of negative charge onto the imide and ester fragments, respectively. The magnitude of the effect is likely smaller for the Mes-substituted experimental PTE anions, where the ester groups are expected to be more strongly twisted due to steric bulk. By contrast, the h bonds become slightly longer upon double deprotonation, implying that the two benzo[*b*]fluorenyl halves of the fused core remain only weakly conjugated with one another.

**Chart 1:**
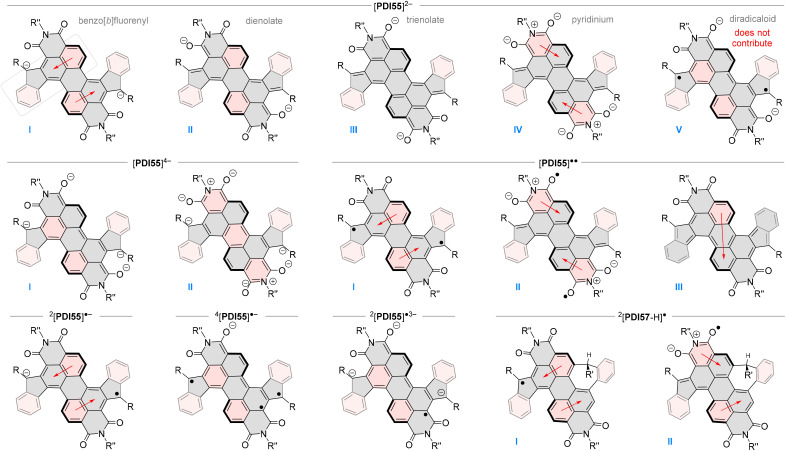
Selected valence structures for selected oxidation levels of PDI55 and PDI57. Clar sextets and their possible shifts are indicated in red.

These trends support extensive delocalization in the diimide anions, largely confined within each naphthalimide half of the PDI, as illustrated by canonical structures I–IV of [PDI55]^2−^ (Chart 1). In particular, structures II and IV enable charge transfer into the imide ring while preserving one Clar sextet. The open-shell structure V is conceptually relevant because it corresponds to a doubly reduced PDI core bearing two fused indenyl radicals. While the apparent thermodynamic stability of PDI dianions could, in principle, suggest such a contribution, its weight in the valence description of [PDI55]^2−^ appears negligible, consistent with the experimentally inferred diamagnetism and with the closed-shell wavefunction obtained for [PDI55']^2−^ in the DFT calculations.

Stepwise reduction of the (5,5)-fused dianions [PTE55']^2−^ and [PDI55']^2−^ leads to progressive lengthening of the f and g bonds and further shortening of the d and e bonds, indicative of increasing enolate-like resonance in the tri- and tetraanions, as in fusion-free PDI/PTE radical anions and dianions. These changes, together with the notable shortening of the h bond, are captured by canonical structures I and II proposed for [PDI55']^4−^ in Chart 1. The tetraanion is thus best described as a reduced PDI dianion core fused to two indenyl anions, a valence picture that rationalizes the emissive character of [PDI55]^4−^. Additional stabilization of the dianionic PDI core can arise from charge-separated contributions such as II, which contain three Clar sextets. Analogous canonical descriptions apply to other species containing a dianionic PDI core, including [PDI57-H]^3−^ and [PDI77-H_2_]^2−^.

Unlike the radical trianions ^2^[PTE55']^3−^ and ^2^[PDI55']^3−^, which display *C*_2_-symmetric geometries and equal distribution of spin density, the radical monoanions ^2^[PTE55']^−^ and ^2^[PDI55']^−^ exhibit symmetry breaking (Fig. 7 and Table S7), with the spin preferentially localized in one benzo[*b*]fluorenyl half of the π system. The extremely broad NIR bands of the monoanions can therefore be interpreted as intervalence charge-transfer transitions characteristic of class-II mixed-valence systems.^58,59^ Interestingly, the quartet state ^4^[PDI55']^−^ remains *C*_2_-symmetric (Fig. 7 and Table S7) and lies only *ca.* 4 kcal mol^−1^ above the doublet state (Table 1). This behavior suggests that ^4^[PDI55']^−^ can be viewed as a pair of indenyl radicals coupled to a PDI radical anion: the doublet results when one indenyl spin pairs with the PDI-centered spin, leading to stabilization and concomitant symmetry breaking (Chart 1). In contrast to the radical monoanion, the absolute Δ*E*_DQ_ gap of [PDI55']^3−^ is much larger (*ca.* −48 kcal mol^−1^), suggesting that the system behaves as a union of two indenyl anions and a PDI radical anion. These conclusions align with the computed spin-density distributions for the mono- and trianion radicals (Fig. S94).

**Table 1:** Calculated open-shell characteristics for (5,5)-fused diradicaloids

Structure^a^	Δ*E*_LH_^b^^,^^c^ kcal mol^−1^	Δ*E*_LH_^b^^,^^d^ kcal mol^−1^	〈*Ŝ*^2^〉 e	*y*_0_ f	*n*_U_ g
^1^[PDI55']	−0.71	−0.76	1.74	0.992	2.20
^3^[PDI55']			2.01	1.000	2.12
^2^[PDI55']^−^	−4.62	−3.88	0.76		1.08
^4^[PDI55']^−^			3.76		3.12
^2^[PDI55']^3−^	−50.46	−47.91	0.75		1.04
^4^[PDI55']^3−^			3.75		3.08
^1^[PTE55']	−0.37	−0.40	1.53	0.996	2.18
^3^[PTE55']			2.01	1.000	2.13
^2^[PTE55']^−^	−7.11	−6.20	0.76		1.09
^4^[PTE55']^−^			3.76		3.13
^2^[PTE55']^3−^	h	h	0.75		1.04

a CAM-B3LYP-GD3BJ/6-31G(d,p) level of theory.

b *E*_low-spin_ − *E*_high-spin_, *i.e.* Δ*E*_ST_ for singlets and Δ*E*_DQ_ for doublets.

c SCF energies.

d SCF + ZPV energies.

e Spin-projected expectation value of the total spin-squared operator.

f Diradicaloid index.

g Number of unpaired electrons.^57^

h Not calculated.

Neutral PDI55' and PTE55' are predicted to have small Δ*E*_ST_ gaps (Table 1), implying weak spin–spin interaction in both diradicals. In line with this assumption, all computational indicators obtained for the singlet states confirm their high open-shell character. Given the topological and geometrical distances between the formal spin centers in these systems (9 bonds and *ca.* 10 Å, respectively), the interaction appears to be weaker than that observed in other diindenoarenes with similar inter-spin separations, for which singlet ground states were observable by ^1^H NMR.^60,61^ This difference can be rationalized by the topology of the π system, which favors separate spin delocalization in the two halves of the diindenoperylene core, as illustrated by canonical structures I and II of [PDI55]˙˙ (Chart 1). Type-II structures preserve the number of Clar sextets present in I and help rationalize the appreciable spin density on the imide oxygens in [PDI55]˙˙ (*cf.*Fig. 6). Although apparently a minor contributor, the closed-shell structure III illustrates the Kekulé-like character of the diindeno[1,2-*a*:1′,2′-*j*]perylene core. Thus, even though the ground states of [PDI55]˙˙ and [PTE55]˙˙ could not be experimentally verified, the predicted preference for the singlet (Table 1) is in line with the topology of the π system.

Time-dependent DFT (TD-DFT) calculations for the simplified models reproduce the key features of the experimental absorption spectra with high fidelity (Fig. S96 and S97). In particular, the charge-transfer bands of the monoanions [PDI57-H]^−^ and [PTE57-H]^−^ are correctly predicted in the NIR region (1302 and 1571 nm for ^1^[PDI57'-H]^−^ and ^1^[PTE57'-H]^−^, respectively), and the hypsochromic shift upon second deprotonation (*e.g.*, [PDI55-H]^−^ to [PDI55]^2−^) is also reproduced. More broadly, the relative changes observed in reductive titrations of neutral (7,7)-fused systems and of deprotonated (5,7)- and (5,5)-fused species are reflected in the computed spectra, providing additional support for assignment of specific redox states. For example, the experimentally observed increase of the optical gap in [PDI57-H]^3−^ relative to [PDI57-H]˙^2−^ is paralleled by the TD-DFT data. Simulated spectra of oxidized products, including the (5,5)-fused radical anions and diradicaloids, are likewise consistent with the experiment. Finally, the triplet and singlet states are predicted to yield very similar spectral envelopes for both [PTE55]˙˙ and [PDI55]˙˙, in line with the weak spin–spin interaction inferred above.

## Conclusions

We mapped the discrete redox–protonation (Pourbaix) spaces of indene-annulated perylenes and found that their experimentally accessible states depend strongly on annulation patterns, carbonyl functionality, and reaction conditions. Under cathodic bias, PDI derivatives largely follow clean, core-centered reductions, while the (5,5)- and (5,7)-fused tetraesters are transiently deprotonated prior to core reduction, consistent with electrogenerated-base chemistry. Potassium *tert*-butoxide acts chemodivergently, enabling both PT- and ET-dominated trajectories, which are kinetically selectable with a crown ether additive. From these nodes in the Pourbaix space, deeper reductions accessed highly charged anions, whereas oxidation furnished neutral open-shell species. The resulting manifold of redox–protonation states features several distinct classes of vis–NIR chromophores: those with formally neutral and dianionic perylene cores are emissive, facilitating their detection in reaction mixtures, whereas dienolate anions, perylene-centered radical anions, and indenyl-based radicals possess broad absorptions in the NIR range.

More broadly, these results establish indene-fused rylenes as a platform for engineering “addressable” Pourbaix spaces in purely organic π systems, where redox level, benzylic protonation, and cation binding jointly define which states can be reached and how they interconvert. This perspective is directly relevant to organic energy-storage concepts, such as redox flow batteries, where proton activity and ion pairing often determine practical redox windows and apparent reversibility. Looking forward, extending this strategy to other aromatic scaffolds and acidic moieties should enable systematic tuning of multiredox NIR chromophores and oligoradicaloids, while providing a general framework for accessing desired states within a formal organic Pourbaix space.

## Author contributions

Agata Wiencierz-Paś: investigation, methodology, conceptualization, writing, and visualization. Liliia Moshniaha: investigation and methodology. Piotr J. Chmielewski: investigation and methodology. Tadeusz Lis: investigation and methodology. Mateusz Waliczek: investigation and methodology. Ryota Kabe: supervision. Marcin Stępień: conceptualization, writing, visualization, supervision, and funding acquisition.

## Conflicts of interest

There are no conflicts to declare.

## Supplementary Material

SC-OLF-D6SC01695B-s001

SC-OLF-D6SC01695B-s002

SC-OLF-D6SC01695B-s003

## Data Availability

Data for this article, including raw UV-vis-NIR absorption spectra, emission spectra, electrochemical data, ESR data, NMR data, DFT/TD-DFT output files (Gaussian logs), and X-ray crystallography files, are available in the University of Wrocław research data repository (RODbuk Dataverse) at https://doi.org/10.34616/17QMKH (CC0 license). Additional supporting data and experimental details are provided in the supplementary information (SI). Supplementary information is available. See DOI: https://doi.org/10.1039/d6sc01695b. CCDC 2497536 contains the supplementary crystallographic data for this paper.^62^
