# Supplementary material for: Redox–protonation landscape of indene-annulated perylenes: chemodivergent switching of multistate NIR chromophores
Source: Chem Sci. 2026 Jun 2;17(28):13850–61. doi: 10.1039/d6sc01695b (PMC13248289; doi:10.1039/d6sc01695b)
Supplement: SC-017-D6SC01695B-s002 [file SC-017-D6SC01695B-s002.pdf]

Supplementary Information (SI) for Chemical Science.
This journal is © The Royal Society of Chemistry 2026

Redox–Protonation Landscape of Indenyl-Annulated Perylenes. Chemodivergent Switching of Multistate NIR Chromophores

Supplementary Information

by Agata Wiencierz-Paś, Liliia Moshniaha, Piotr J. Chmielewski, Tadeusz Lis, Mateusz Waliczek, Ryota Kabe, Marcin Stępień

Table of Contents

1. General 2
  1.1. Synthetic Methods 2
  1.2. Analytical Methods 2
  1.3. Computational Methods 4
2. Synthesis and Characterization 5
  Compound **PTE55-**H_2 5
  Compound **PTE57**-H_2 8
3. Experimental Data 11
  3.1. X-ray Crystallography 11
  3.2. NMR Spectroscopy 13
  3.3. Absorption Spectroscopy 24
  3.4. ESR Spectroscopy 39
  3.5. Emission Spectroscopy 40
  3.6. Electrochemistry 47
  3.7. Mass spectrometry 54
4. Computational Data 59
  4.1. Energies and Geometries 59
  4.2. Orbitals and Densities 64
  4.3. Spectroscopy 66
5. References 78

1. General

In all the Supporting Information, we use **KN** as the abbreviation of **potassium naphthalenide**, and **NaN** as the abbreviation of **sodium naphthalenide**. The abbreviation **18c6** stands for 18-crown-6, and the abbreviation **15c5** stands for 15-crown-5.

1.1. Synthetic Methods

All syntheses were thoroughly described in the previous report[1]. Here, we describe and characterize the two compounds, **PTE55**-H_2 and **PTE57**-H_2, which were not previously obtained in pure form.

All reagents and solvents were obtained from commercial suppliers and used without further purification unless specified otherwise. Dry dichloromethane was distilled from CaH_2 and stored over 3Å or 4Å molecular sieves. Dry tetrahydrofuran and dry toluene were passed through an activated alumina column. Silica gel 60 (0.063–0.200 mm) or neutral alumina (Brockmann III) were used for column chromatography. The active neutral alumina was deactivated to Brockmann III grade using 6 mL of deionized water for 100 g of active alumina.

1.2. Analytical Methods

NMR spectroscopy

^{1}H and ^{13}C NMR spectra were recorded on JEOL JNM-ECZ500R 500 MHz, Bruker Avance III 500 MHz, or Bruker Avance III 600 MHz spectrometers and referenced using solvent signals[2]. Spectra measured on Bruker instruments were processed and analyzed using TopSpin 4.1.3. Spectra measured on a JEOL instrument were processed and analyzed using JASON Version 4.0.7735 by JEOL UK.

Voltammetry

Electrochemical measurements (cyclic and differential pulse voltammetry) were performed using an Autolab (Metrohm) potentiostat/galvanostat system in tetrahydrofuran (THF) solutions, with a glassy carbon electrode, a platinum wire, and Ag/AgCl (sat. KCl) as the working, auxiliary, and pseudoreference electrodes, respectively. Tetrabutylammonium hexafluorophosphate ($TBAPF_6$) was used as a supporting electrolyte (0.1 M). The potentials were referenced using the ferrocene/ferrocenium (Fc/Fc^+) couple as an internal standard. Measurement parameters for cyclic voltammetry (CV): scan rate 100 mV/s. Measurement parameters for differential pulse voltammetry (DPV): modulation time 0.05 s; interval time 0.2 s; step potential 0.00195 V; modulation amplitude 0.025 V. Spectroelectrochemistry was performed using a Room Temperature OTTLE Electrochemical cell (Specac Omni Cell, 0.1 mm path length) with a platinum mesh working electrode and an internal silver wire pseudoreference. 0.1 M $TBAPF_6$ in THF was used as a supporting electrolyte. Scan rate: 1 mV/s.

Mass spectrometry

MALDI mass spectra were recorded on a Bruker Daltonics ultrafleXtreme or a JMSS3000 SpiralTOF™-plus 2.0 MALDI–TOF spectrometer. ESI–MS spectra were recorded on a Bruker Compact spectrometer with an ESI ion source.

Absorption spectroscopy

UV–vis–NIR spectra were recorded on a PerkinElmer Lambda 1050+ UV/Vis/NIR spectrophotometer. UV–vis–NIR spectra were measured during spectroelectrochemistry using a JASCO V–700 spectrophotometer.

During titrations, we started with THF solutions of all compounds, using *t*BuOK (2 M in THF) as a base. To all solutions, 18–crown–6 (18c6) was added to improve solubility.

Emission spectroscopy

Emission measurement during bulk electrolysis. The emission spectra of the dianions [**PDI*mn***-H_2]$^{2-}$ were obtained during bulk electrolysis (WaveNow Wireless Potentiostat, Pine Research Instrumentation) in THF solutions containing 0.1 M $TBAPF_6$ as electrolyte in a nitrogen-filled glovebox. The spectra were recorded with a UV–vis–NIR spectrometer (ULS2048CL-EVO, Avantes) and NIR spectrometer (AvaSpec-NIR256-2.5-HSC-EVO, Avantes), the Thorlabs 365 nm LED with 10 nm bandpass filter was used as a source of excitation and a focus lens was installed to precisely excite only the area of the working electrode (platinum mesh 6 x 6 mm was used as a working electrode). Emission spectra were measured using a 1 mm quartz cuvette in a right-angle (90°) detection geometry. The excitation beam was directed onto the surface of the working electrode at an angle of 45°.

Emission measurements during titration with *t*BuOK and KN. The changes of the emission spectra of [**PDI*mn***-H_2] during basic and reductive titrations were measured for 10^{-5} M solutions in THF in 10 mm quartz cuvettes in a nitrogen-filled glovebox. The spectra were recorded with a UV–vis–NIR spectrometer (ULS2048CL-EVO, Avantes) and the Thorlabs 365 nm LED with a 10 nm bandpass filter was used as a source of excitation.

Electron paramagnetic resonance spectroscopy

EPR spectra were recorded on a Bruker ELEXSYS E500 CW-EPR spectrometer. For the neutral open-shell products derived from [**PTE55**]$^{2-}$ and [**PDI55**]$^{2-}$, spectra were recorded in frozen toluene solutions. In both cases, the experimental spectra contain two superimposed components: (i) a broad triplet-state signal with resolved zero-field splitting, assigned to the neutral diradicaloid, and (ii) a narrower doublet contribution, assigned to a residual monoradical species formed concurrently under the oxidative conditions. The simulated spectra shown in Figures S58 and S60 were intended primarily to reproduce the characteristic triplet features and the corresponding *D* values; the intense central signal was truncated in the main text figure to make the weaker zero-field-splitting pattern visible on a readable scale. The triplet contribution is the key feature supporting the open-shell assignment of the neutral (5,5)-fused products. In frozen toluene, [PTE55]$^{••}$ gives a clear triplet-like spectrum with a simulated *D* value of 49.5 G, and the analogous [PDI55]$^{••}$ species gives a very similar *D* value of 48.5 G. Within the point-dipole approximation, these values correspond to effective spin-spin separations substantially shorter than the geometric separations between the formally radical-bearing indenyl carbons in the corresponding DFT-optimized structures. This discrepancy is consistent with significant spin delocalization into the perylene core, in agreement with the calculated spin-density distributions discussed in the main text.

Because the doublet and triplet signals partially overlap, the variable-temperature EPR data were evaluated only qualitatively. In principle, the doublet component should follow Curie-type behavior, whereas the triplet contribution from a singlet-triplet manifold is expected to show a weaker and model-dependent temperature dependence. In the present case, the spectral regions dominated by the triplet component can still be identified, and their doubly integrated intensities show only a weak variation over the experimentally accessible temperature range in frozen toluene. However, the total dynamic range of this variation is small, and overlap with the residual doublet component prevents a reliable quantitative extraction of the singlet-triplet gap by fitting procedures such as a Bleaney-Bowers analysis. Accordingly, the EPR data are not used here to determine ΔE_{ST} numerically. The EPR results are instead interpreted in a more limited way. First, they establish the presence of triplet-state species in the oxidized solutions of the (5,5)-fused systems. Second, the weak temperature dependence of the triplet-dominated signal is consistent with a small singlet-triplet energy gap, although it is not by itself a definitive measure of that gap. This interpretation is supported independently by the low-temperature ^{1}H NMR behavior, which shows strong paramagnetic broadening even at 190 K, and by the DFT results, which predict small singlet-triplet gaps and pronounced open-shell character for the neutral [PTE55]$^{••}$ and [PDI55]$^{••}$ states. By contrast, oxidation of [**PDI57**-H]$^{-}$ gives a monoradical species, [PDI57-H]$^{•}$, whose room-temperature toluene spectrum is well described as a doublet with resolved hyperfine structure (Figure S59). In

this case, no triplet contribution is expected, and the EPR analysis is correspondingly more straightforward. The observed hyperfine pattern is consistent with the computed spin-density distribution for the unsymmetrical (5,7)-fused framework, in which the unpaired spin is primarily associated with the indenyl-derived segment and delocalizes into the fused PDI π-system.

X-ray crystallography

Single crystal X-ray diffraction was performed on an XtaLAB Synergy-R DW diffractometer with a HyPix ARC 150° detector using Cu Kα radiation. Data collection and reduction were performed using CrysAlisPro 1.171.42.42a software (Rigaku Oxford Diffraction, 2022). Single crystals of [**PDI57**-H]$^{3-}$ were obtained by slow evaporation of THF from a mixture of [**PDI57**-H_2], potassium naphthalenide (25 equiv.), and 18c6 (50 equiv.) in THF under an inert atmosphere (glovebox). The crystal structure was deposited in CCDC with the number **2497536**. The checkCIF alerts were addressed by noting that the crystal was extremely sensitive to oxygen and moisture, necessitating a rapid measurement in air, which led to a low observed/unique reflection ratio and a high wR2 value; additional alerts concerning atypical displacement parameters (C73 high Ueq and K2 low Ueq relative to neighbors) were attributed to substantial disorder in the structure. Accordingly, the structure is used only for a qualitative discussion of the gross shape of the trianion salt, the presence of multiple potassium sites, and the distinction between O-bound and η3-type coordination modes. No detailed quantitative interpretation of bond metrics is attempted.

1.3. Computational Methods

Density functional theory (DFT) calculations were performed using Gaussian 16.[3] DFT geometry optimizations were carried out in unconstrained C_1 symmetry, using molecular mechanics or semiempirical models as starting geometries. The calculations were performed using the hybrid functional B3LYP[3–6], including the CAM[7] and GD3BJ[8] corrections, and the 6-31G(d,p) basis set. Wavefunctions were tested for instabilities and, if required, reoptimized until a stable solution was found. Each structure was optimized to meet standard convergence criteria. The existence of a local minimum was verified by a normal mode frequency calculation. Natural population analysis[9] was performed as implemented in Gaussian 16.

2. Synthesis and Characterization

Compound **1** (tetrabutyl 1,7-bis(2-formylphenyl)perylene-3,4,9,10-tetracarboxylate) was synthesized as described previously.[1]

General procedure for the synthesis of **PTEmn**-H_2

To a flame-dried 250 mL round-bottomed flask, compound **1** (4066 mg, 4.72 mmol) in 100 mL of tetrahydrofuran was added. The solution was purged with nitrogen for 15 minutes, then 2-mesitylmagnesium bromide (38.55 mL of 1M solution in tetrahydrofuran, 37.8 mmol) under nitrogen atmosphere was added. The mixture was stirred at room temperature for 18 hours. The reaction was quenched with 1M hydrochloric acid and extracted with dichloromethane. The organic phase was dried over anhydrous sodium sulfate and evaporated under a vacuum. The crude product was dissolved in 100 mL of dichloromethane in a dry round-bottomed flask. The solution was purged with nitrogen for 15 minutes, and boron trifluoride diethyl etherate (2.38 mL, 18.9 mmol) was added. The mixture was stirred at room temperature for 10 minutes. The reaction was quenched with water and extracted with dichloromethane. The organic phase was dried over anhydrous sodium sulfate and evaporated under a vacuum. The crude mixture containing **PTE77**-H_2, **PTE57**-H_2, and **PTE55**-H_2 was roughly separated by column chromatography (silica gel, 10% ethyl acetate in *n*-hexane), then purified by second column chromatography (neutral alumina, Brockmann **III**, 10% ethyl acetate in *n*-hexane), and the pure **PTE55**-H_2 was obtained as a red solid (100 mg, 0.01 mmol, 2%), pure **PTE57**-H_2 was obtained as an orange solid (3163 mg, 2.97 mmol, 63%), pure PTE77-H_2 was obtained as a yellow solid (687 mg, 0.65 mmol, 14%).

Compound **PTE55-**H_2

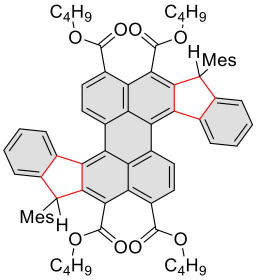

¹H NMR (500 MHz, chloroform-*d* , 300 K): δ [ppm] 8.64 (d, *J* = 7.7 Hz, 2H), 8.42 (d, *J* = 8.0 Hz, 2H), 7.80 (d, *J* = 7.7 Hz, 2H), 7.25 (t, *J* = 7.3 Hz, 2H), 7.16 (t, *J* = 7.6 Hz, 2H), 7.08 (d, *J* = 7.5 Hz, 2H), 7.00 (s, 2H), 6.53 (s, 2H), 6.14 (s, 2H), 4.31-4.18 (m, 4H), 3.57 (m, 2H), 3.42 (m, 2H), 2.80 (s, 6H), 2.22 (s, 6H), 1.71 (m, 4H), 1.42 (m, 4H), 1.32 (m, 8H), 0.94 (s, 6H), 0.93 (t, *J* = 7.5 Hz, 6H), 0.87 (t, *J* = 7.5 Hz, 6H).

¹³C NMR (125 MHz, chloroform-*d* , 300 K): δ [ppm] 168.87, 167.93, 150.70, 148.89, 139.38, 138.76, 137.97, 136.89, 136.22, 133.63, 132.31, 131.63, 130.43, 129.84, 129.63, 129.53, 128.81, 128.47, 126.59, 126.46, 125.89, 125.42, 124.87, 121.71, 65.43, 65.25, 49.70, 30.79, 30.19, 29.84, 21.87, 20.98, 19.41, 19.35, 19.28, 14.01, 13.91.

HRMS (ESI–TOF): *m*/*z*: $[M + Na]^+$ Calcd for $C_{72}H_{72}O_8Na$: 1087.5119; Found 1087.5107

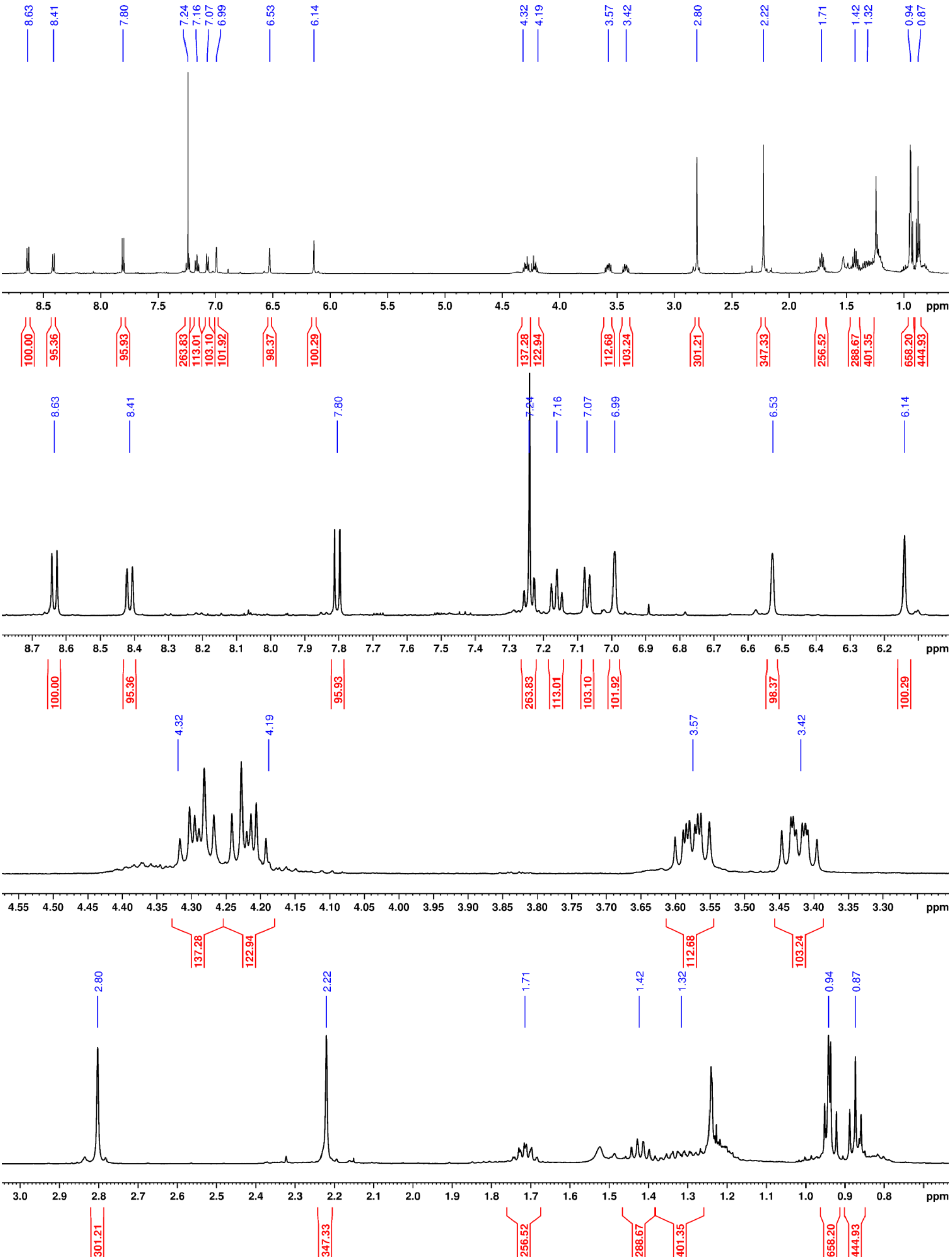

Figure S1. ^{1}H NMR spectrum and zoom-ins of **PTE55**-H_2. Signal at 7.24 ppm (triplet) overlaps with $CHCl_3$ singlet. (500 MHz, chloroform-*d* , 300 K).

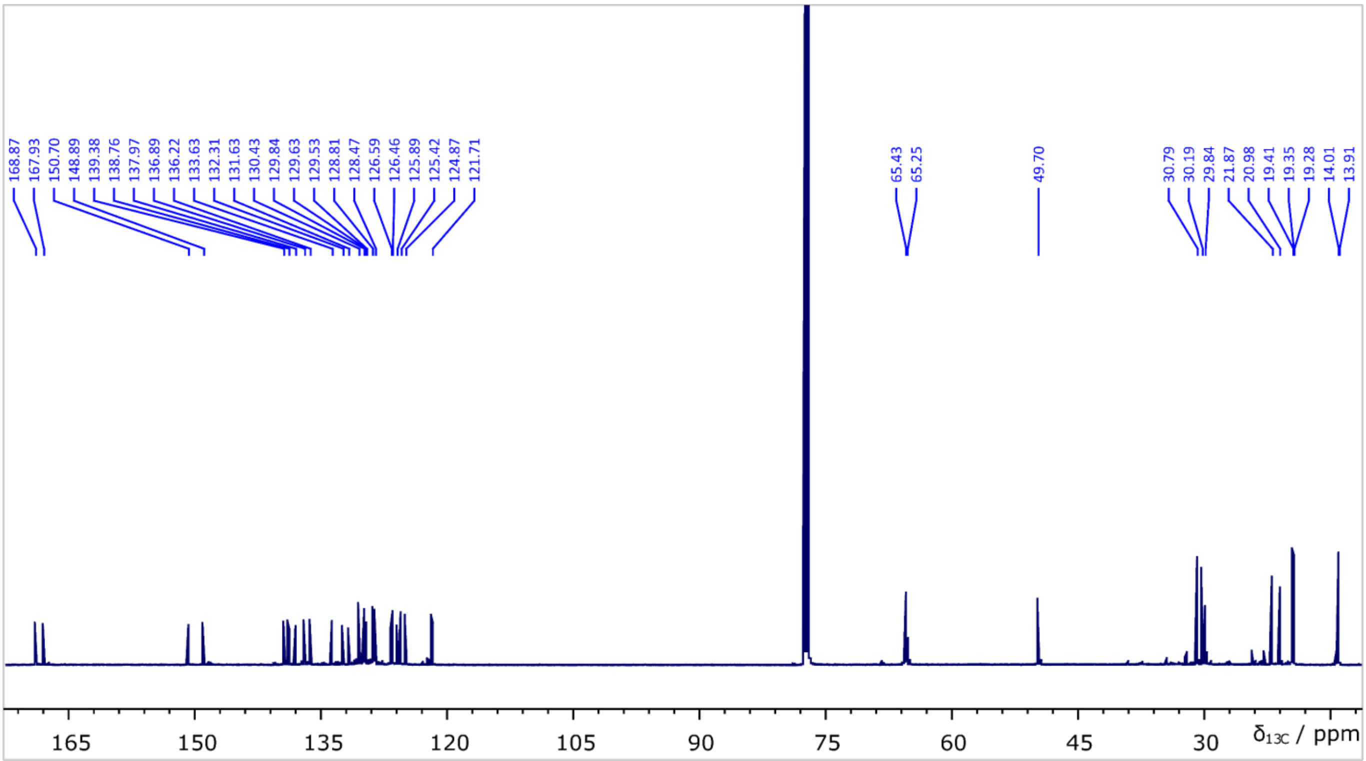

Figure S2. ^{13}C NMR spectrum of **PTE55**-H_2 (125 MHz, chloroform-*d* , 300 K).

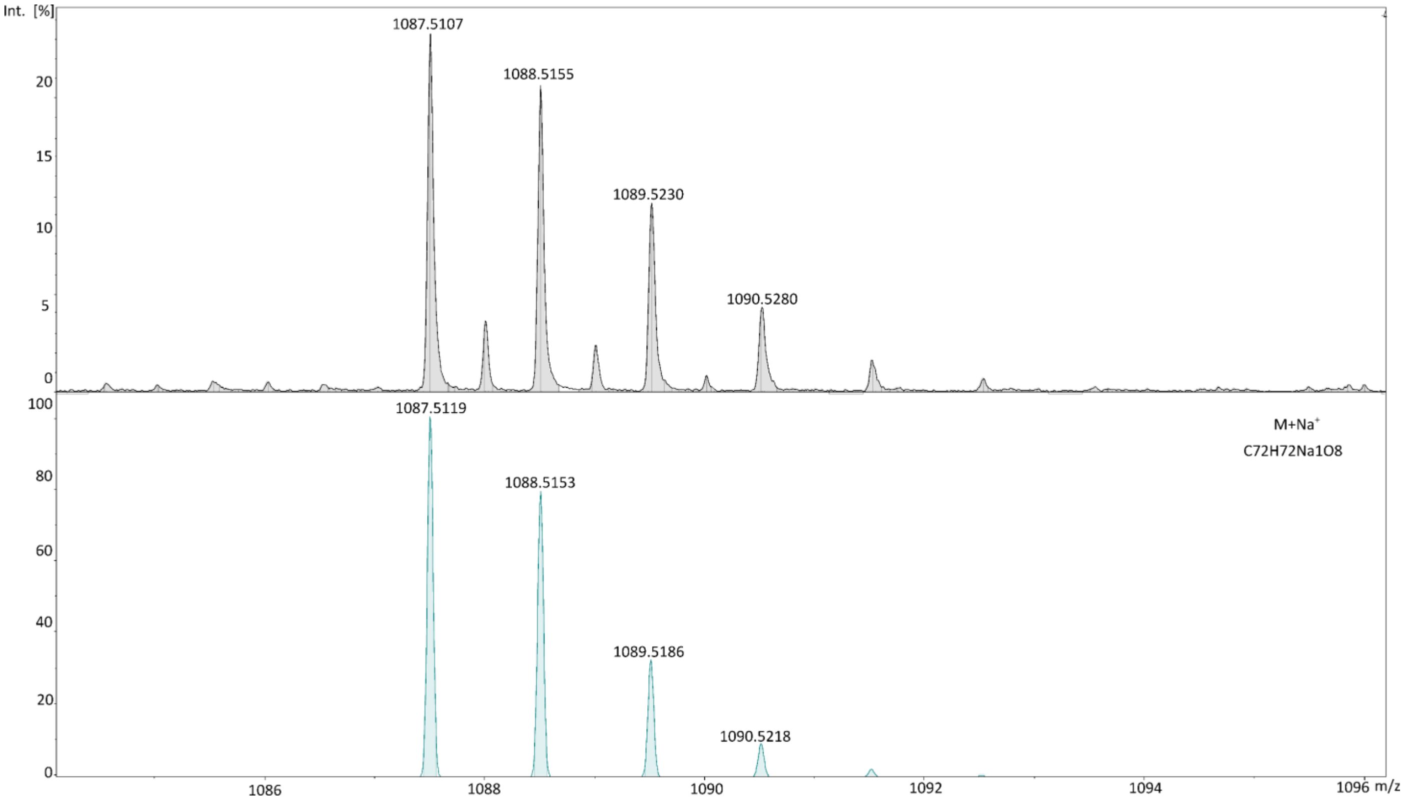

Figure S3. High resolution mass spectrum of **PTE55**-H_2+Na^+ (MALDI–TOF, top: experimental, bottom: simulated).

Compound **PTE57**-H_2

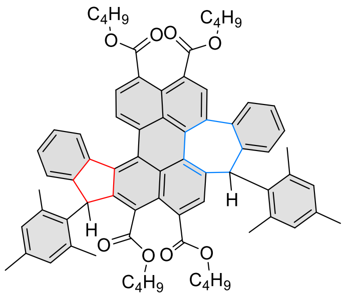

^{1}H NMR (500 MHz, chloroform-*d* , 300 K): δ [ppm] 8.71 (d, *J* = 7.8 Hz, 1H), 8.24 (s, 1H), 8.20 (d, *J* = 8.0 Hz, 1H), 8.09 (d, *J* = 7.7 Hz, 1H), 7.93 (s, 1H), 7.48 (m, 1H), 7.30 (m, 2H), 7.25 (m, 1H), 7.21 (td, *J* = 7.4, 1.0 Hz, 1H), 7.11 (t, *J* = 8.0, 1H), 7.06 (d, *J* = 7.8 Hz, 1H), 6.93 (s, 1H), 6.40 (s, 1H), 6.17 (s, 1H), 5.47 (s, 1H), 4.40 (m, 4H), 4.21 (m, 1H), 4.04 (m, 1H), 3.47 (m, 1H), 3.41 (m, 1H), 2.78 (s, 3H), 2.36 (s, 3H), 2.16 (s, 3H), 1.83 (m, 4H), 1.62 (m, 2H), 1.50 (m, 4H), 1.35 (m, 2H), 1.20 (m, 2H), 1.12 (m, 2H), 1.01 (t, *J* = 7.3 Hz, 3H), 0.99 (t, *J* = 7.3 Hz, 3H), 0.93 (t, *J*= 7.3 Hz, 3H), 0.78 (t, *J* = 7.3 Hz, 3H), 0.65 (s, 3H).
^{13}C NMR (125 MHz, chloroform-*d* , 300 K): δ [ppm]168.95, 168.83, 168.30, 167.50, 150.01, 149.12, 144.09, 139.56, 139.47, 138.82, 138.65, 138.39, 138.14, 136.75, 136.59, 135.98, 134.14, 133.61, 132.16, 131.54, 131.49, 131.48, 131.29, 130.63, 130.40, 130.32, 130.23, 129.64, 129.61, 129.39, 128.85, 128.61, 127.67, 127.46, 126.98, 126.88, 126.38, 126.07, 125.47, 125.18, 124.89, 124.87, 121.37, 65.69, 65.64, 65.14, 64.85, 49.33, 46.35, 30.79, 30.77, 30.76, 29.99, 21.74, 20.98, 20.84, 19.40, 19.37, 19.36, 19.19, 18.99, 13.94, 13.93, 13.92, 13.82.
HRMS (ESI–TOF): *m/z*: $[M + Na]^{+}$ Calcd for ; $C_{72}H_{72}O_8Na^{+}$: 1087.5119; Found 1087.5113.

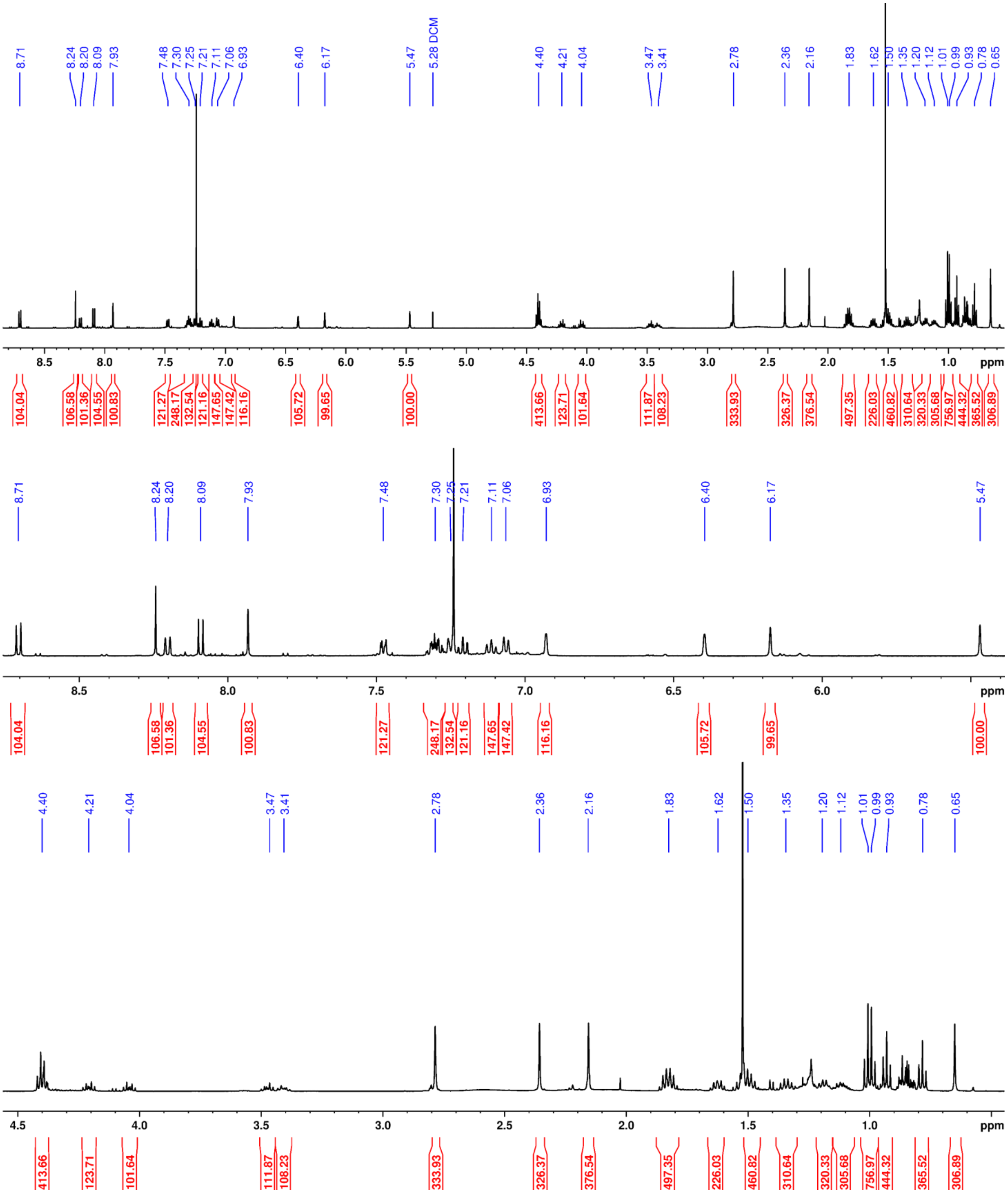

Figure S4. ^{1}H NMR spectrum and zoom-ins of **PTE57**-H_2 (500 MHz, chloroform-*d* , 300 K).

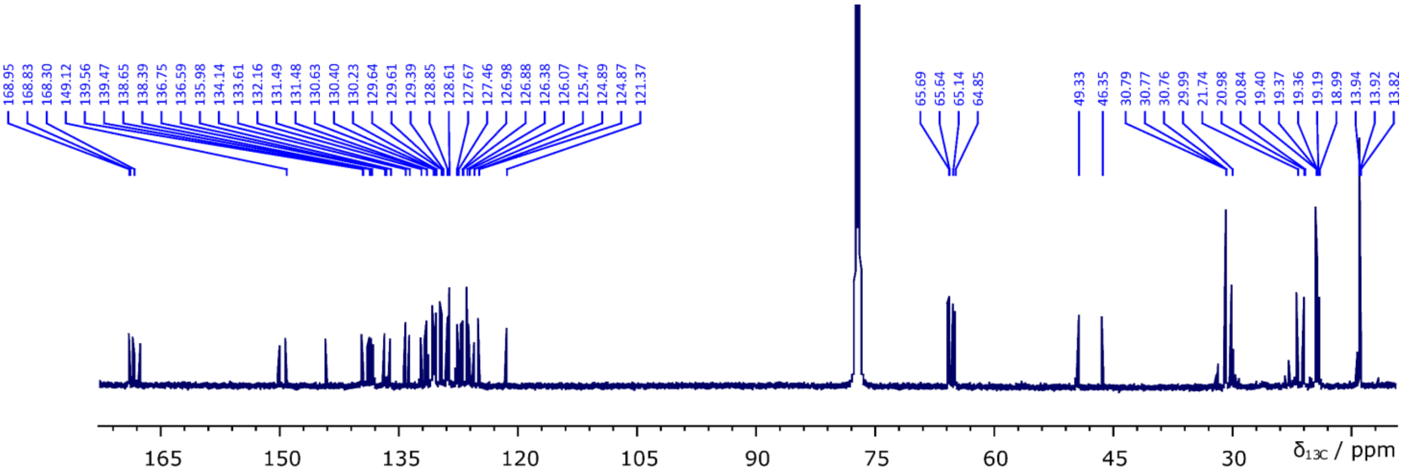

Figure S5. ^{13}C NMR spectrum of **PTE57**-H_2 (125 MHz, chloroform-*d* , 300 K).

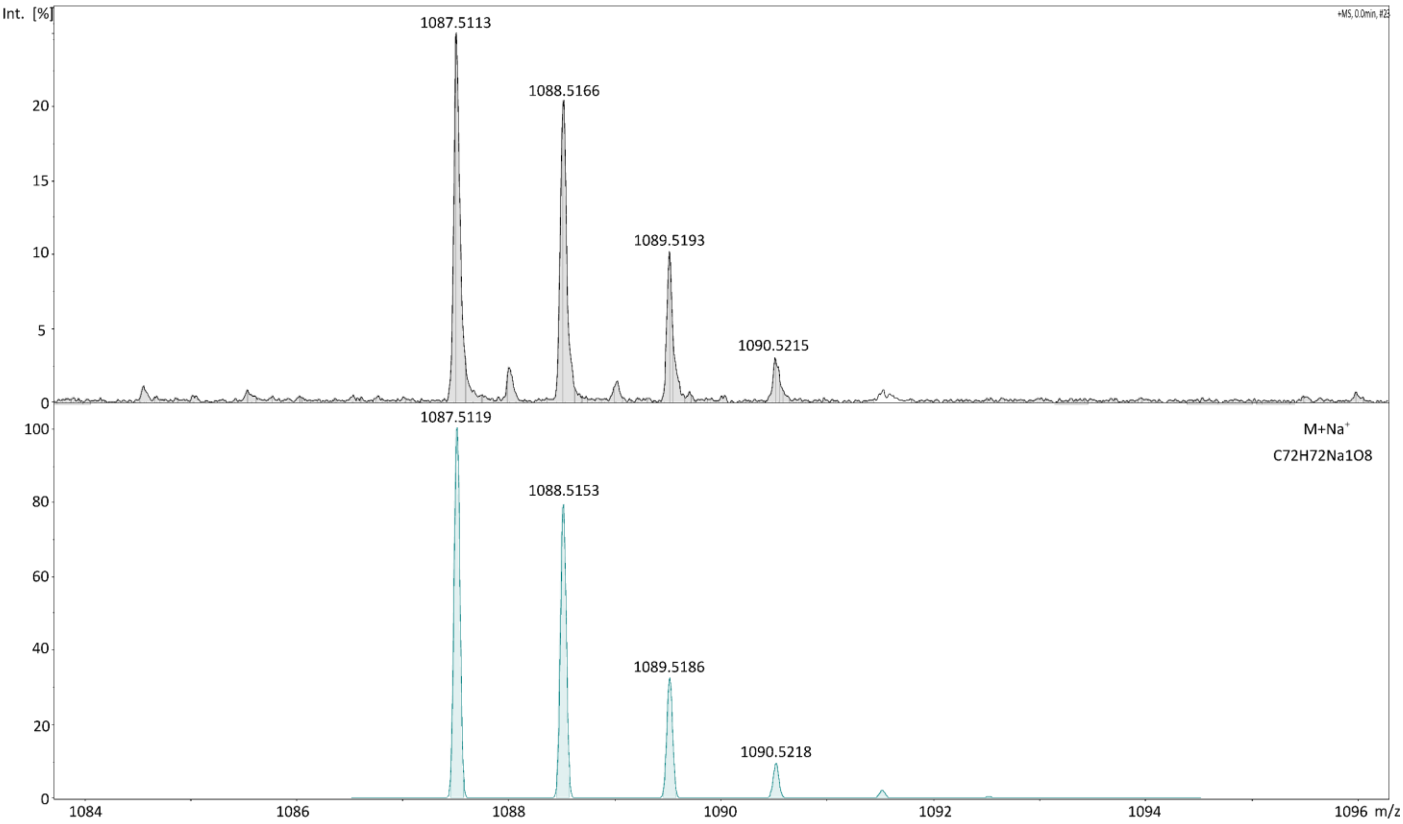

Figure S6. High resolution mass spectrum of **PTE57**-H_2+Na^+ (ESI–TOF, top: experimental, bottom: simulated).

3. Experimental Data

3.1. X-ray Crystallography

Table S1. Crystal data and structure refinement for $[K(18c6)]_3$[**PDI57**-H].

Identification code	aw785t
Empirical formula	$[K(18c6)]_3$[**PDI57**-H]·1.5(18c6)·0.9THF<br>$C_{119.6}H_{164.2}N_2O_{31.9}K_3$
Formula weight	2257.62
Temperature/K	100(2)
Crystal system	monoclinic
Space group	I2/a
a/Å	22.908(9)
b/Å	37.122(14)
c/Å	30.280(10)
α/°	90
β/°	98.54(4)
γ/°	90
Volume/Å^3	25464(16)
Z	8
ρ_{calc}g/cm^3	1.178
μ/mm^{-1}	1.543
F(000)	9664.0
Crystal size/mm^3	0.31 × 0.07 × 0.05
Radiation	Cu Kα (λ = 1.54184)
2Θ range for data collection/°	4.57 to 148.93
Index ranges	$-28 \le h \le 28$, $-45 \le k \le 43$, $-37 \le l \le 37$
Reflections collected	58969
Independent reflections	58969 [R_{int} = n/a*, R_{sigma} = 0.0636]
Data/restraints/parameters	58969/518/1318
Goodness-of-fit on F^2	1.783
Final R indexes [I>=2σ (I)]	R_1 = 0.1914, wR_2 = 0.4127
Final R indexes [all data]	R_1 = 0.3549, wR_2 = 0.4550
Largest diff. peak/hole / e Å^{-3}	0.61/–0.47

* Twinned refinement

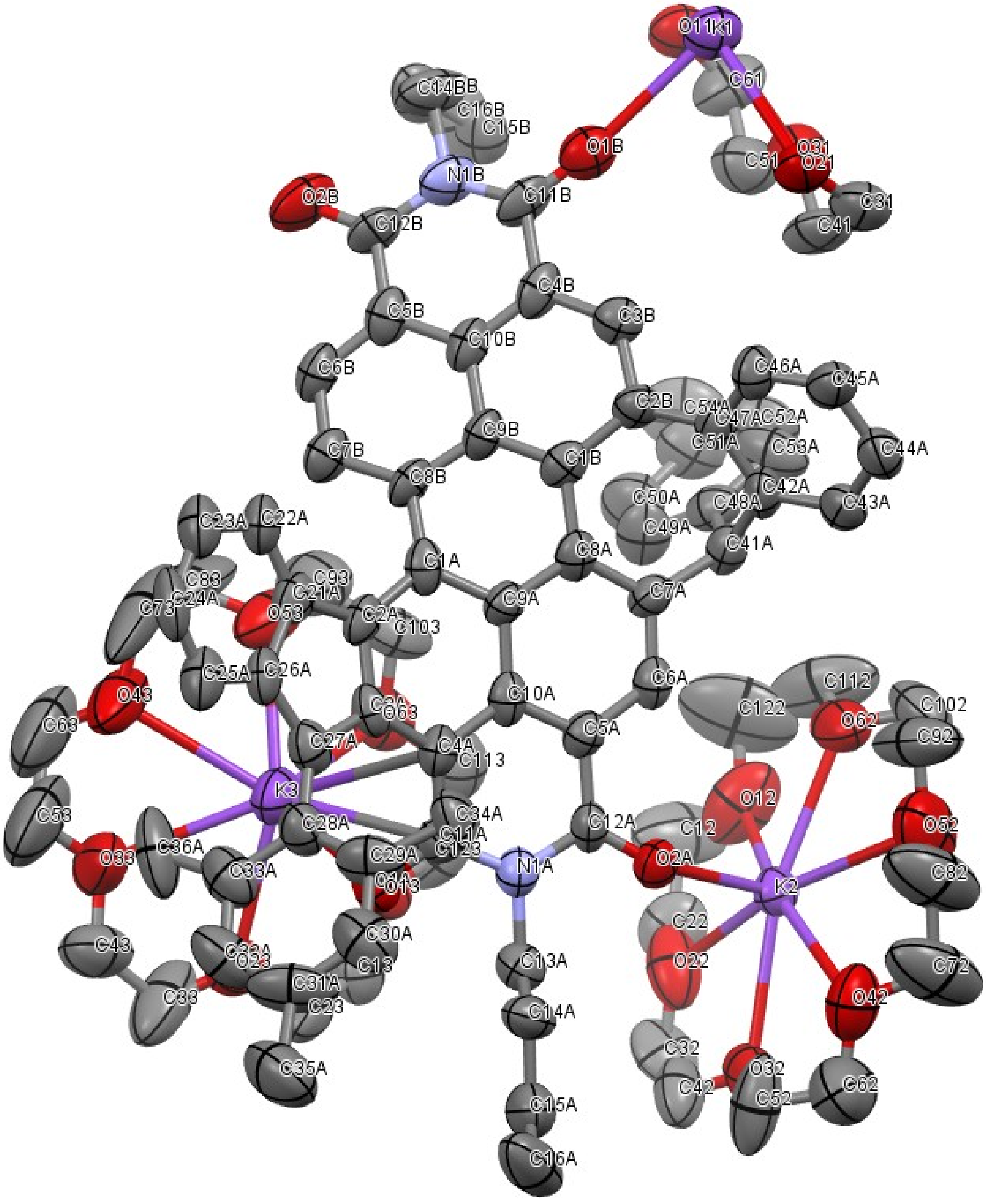

Figure S7. Asymmetric unit of K_3(**PDI57**-H)(18c6)$_{4.8}$. Hydrogen atoms and solvent molecules omitted for clarity.

3.2. NMR Spectroscopy

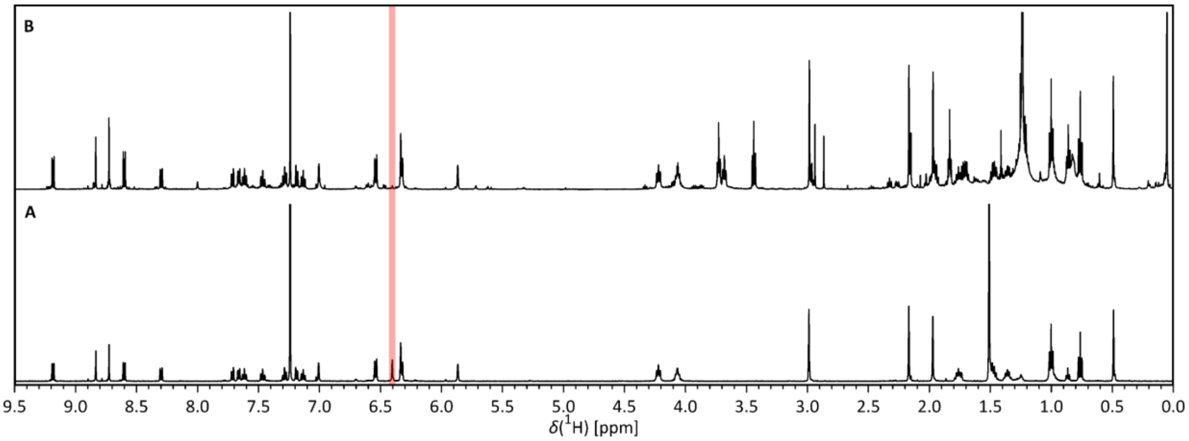

Figure S8. ^{1}H NMR spectra (500 MHz, chloroform-*d*) of A: [**PDI57**-H_2], B: [**PDI57**-DH]. Deuteration of sp^{3} bridge indicated in red.

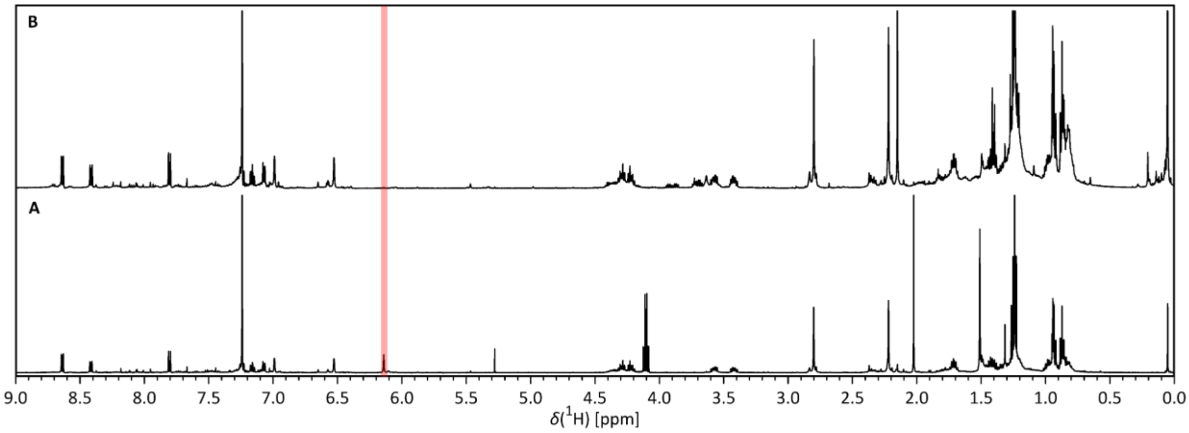

Figure S9. ^{1}H NMR spectra (500 MHz, chloroform-*d*) of A: [**PTE55**-H_2], B: [**PTE55**-D_2]. Deuteration of sp^{3} bridge indicated in red.

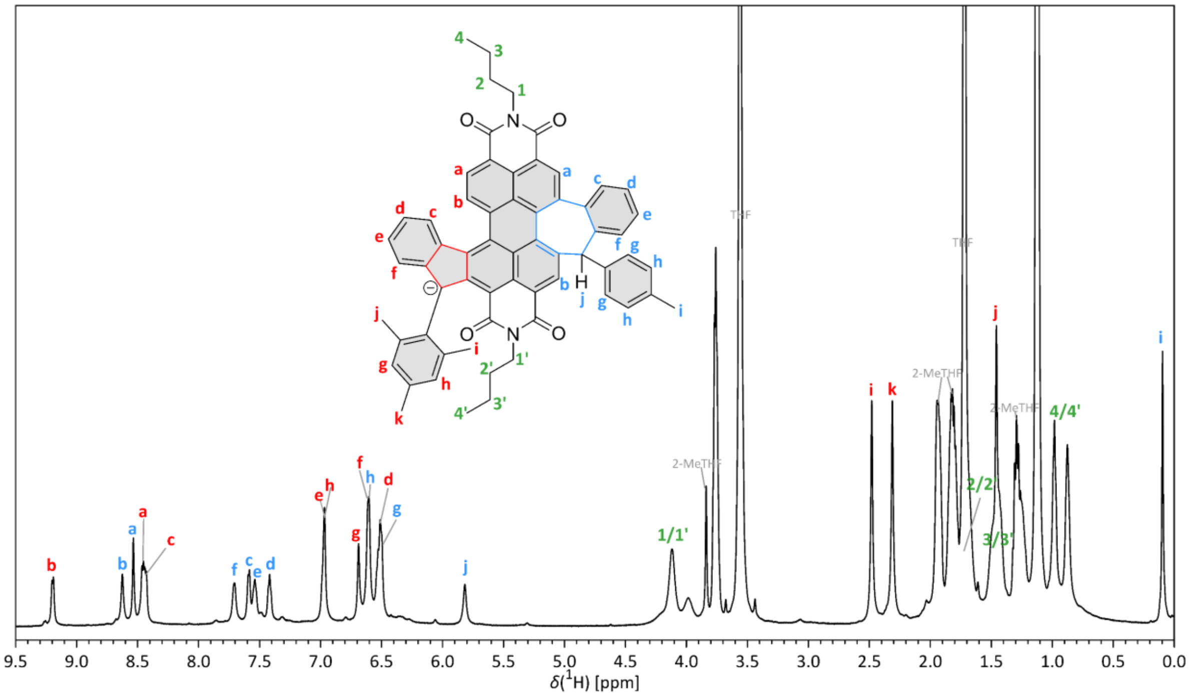

Figure S10. ^{1}H NMR spectrum (600 MHz, THF-d_8, 190 K) of [**PDI57**-H]$^{-}$.

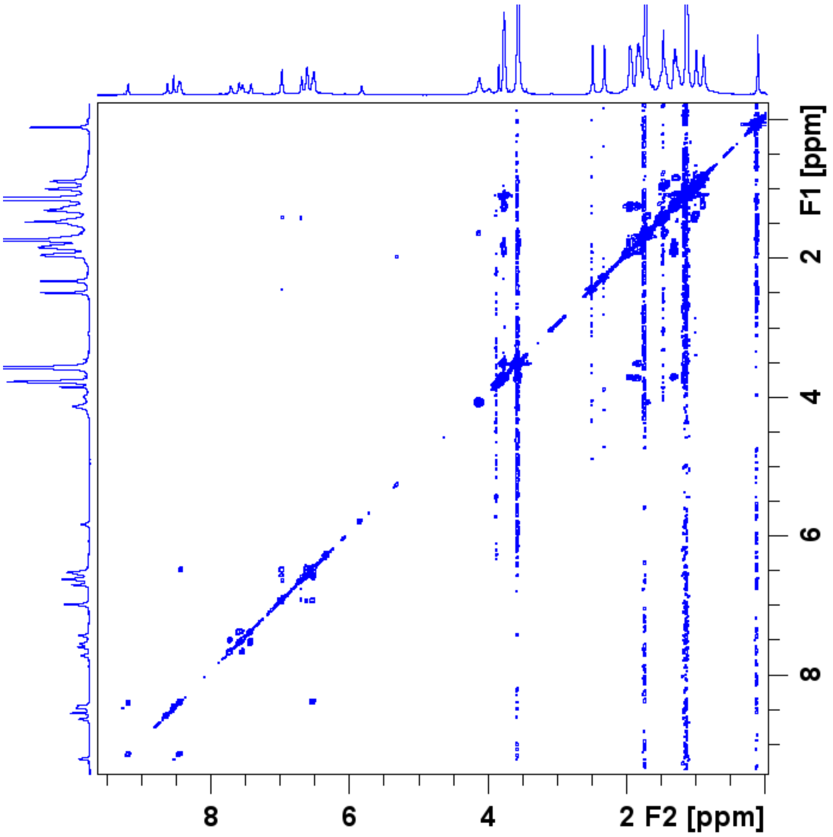

Figure S11. ^{1}H COSY NMR spectrum of [**PDI57**-H]$^-$ in THF-d_8, 190 K.

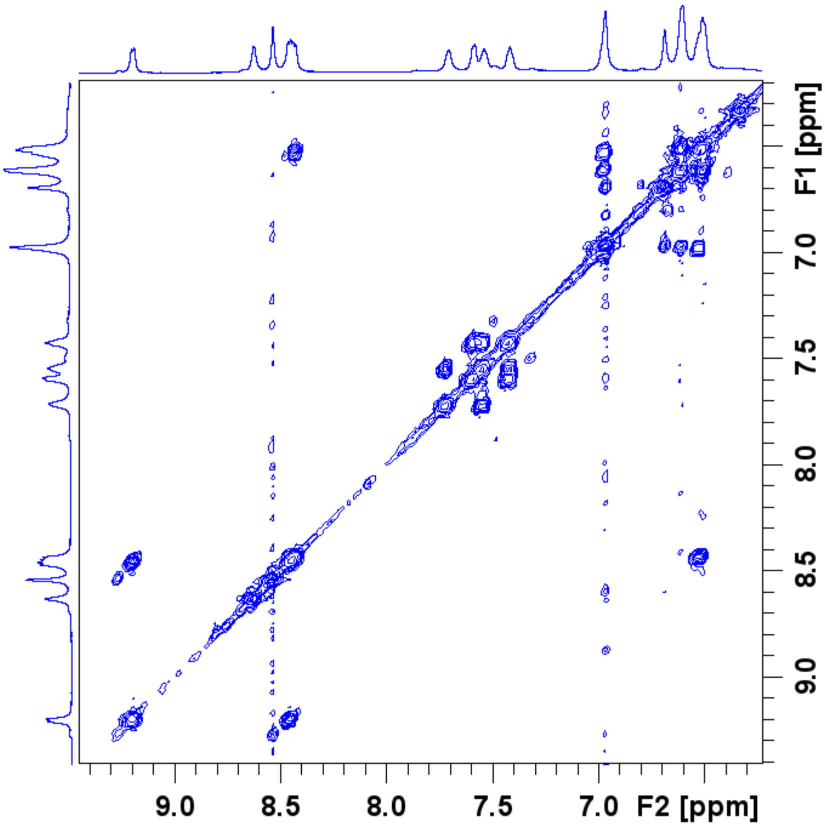

Figure S12. Partial ^{1}H COSY NMR spectrum of [**PDI57**-H]$^-$ in THF-d_8, 190 K.

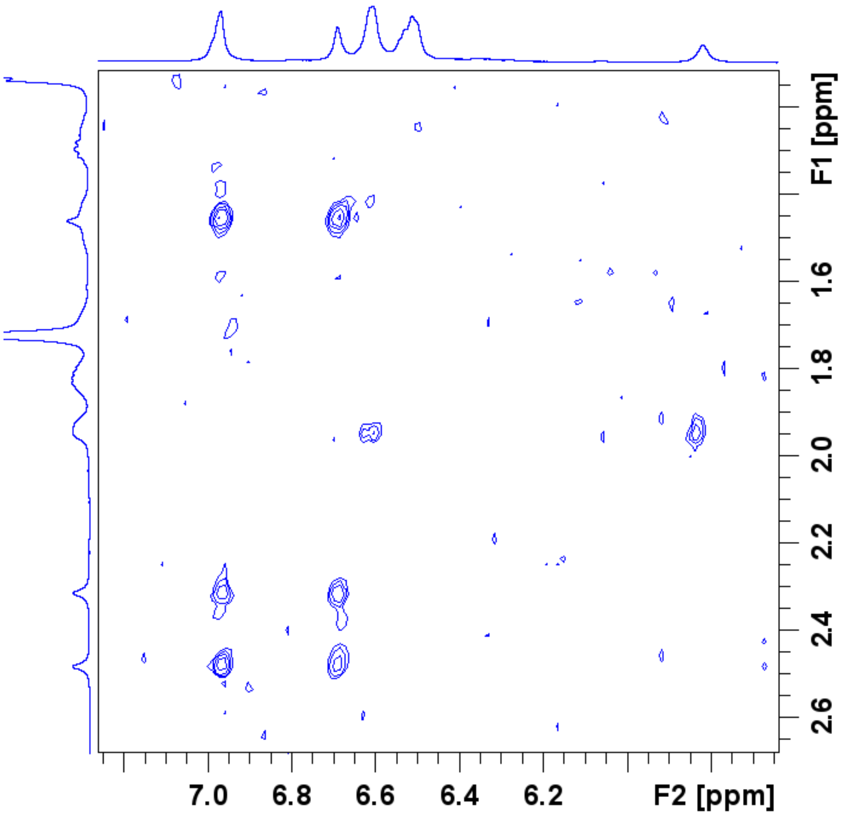

Figure S13. Partial ^{1}H COSY NMR spectrum of [**PDI57**-H]$^{-}$ in THF-d_8, 190 K.

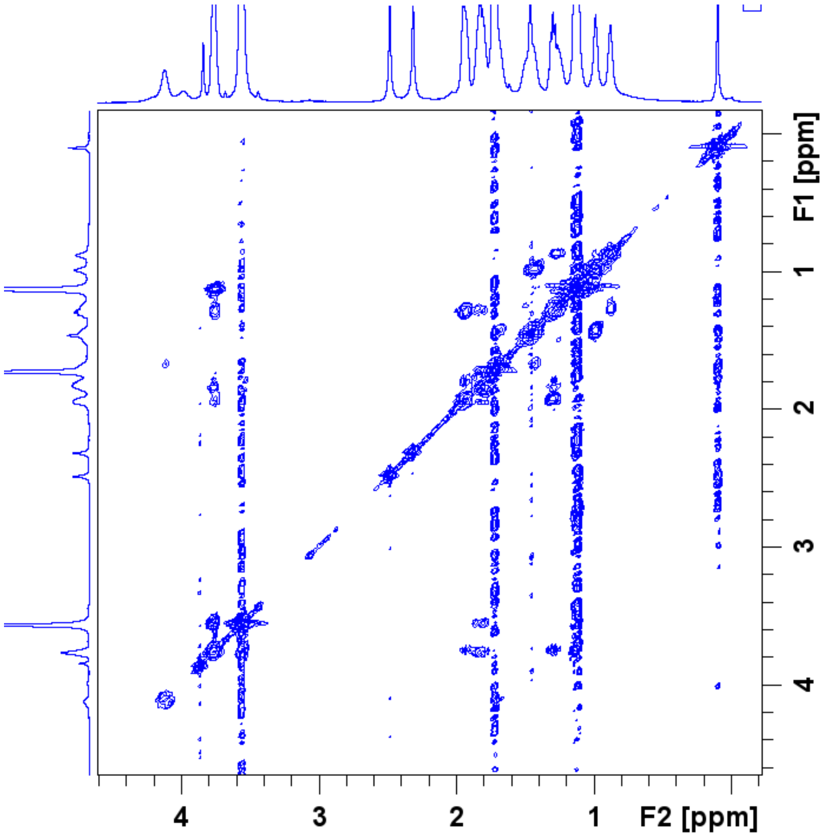

Figure S14. Partial ^{1}H COSY NMR spectrum of [**PDI57**-H]$^{-}$ in THF-d_8, 190 K.

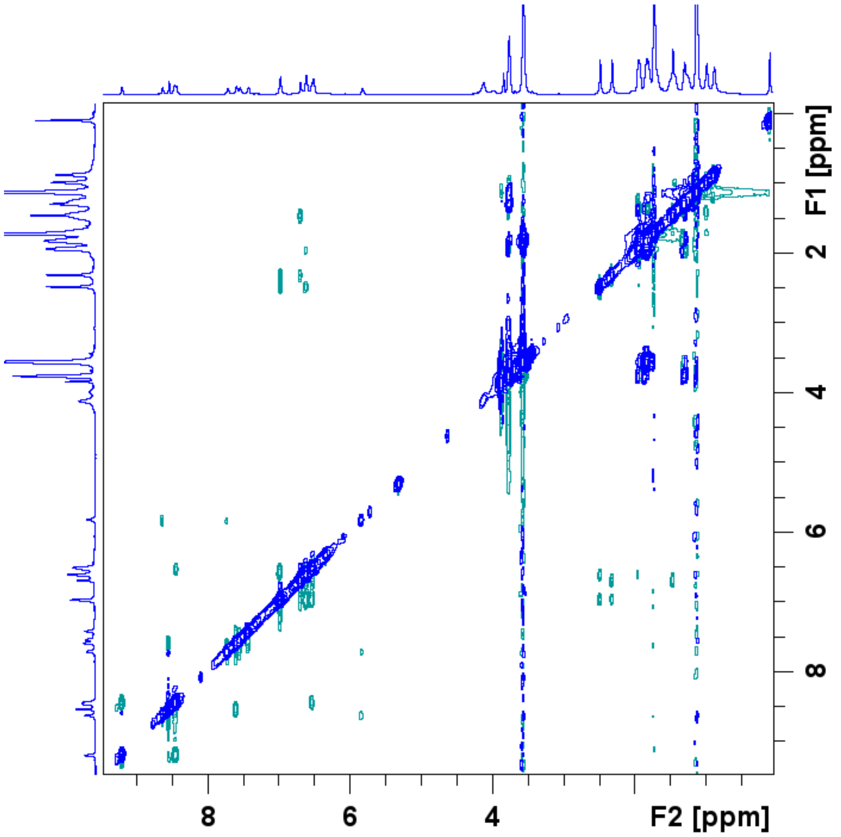

Figure S15. ^{1}H ROESY NMR spectrum of [**PDI57**-H]$^{-}$ in THF-d_8, 190 K.

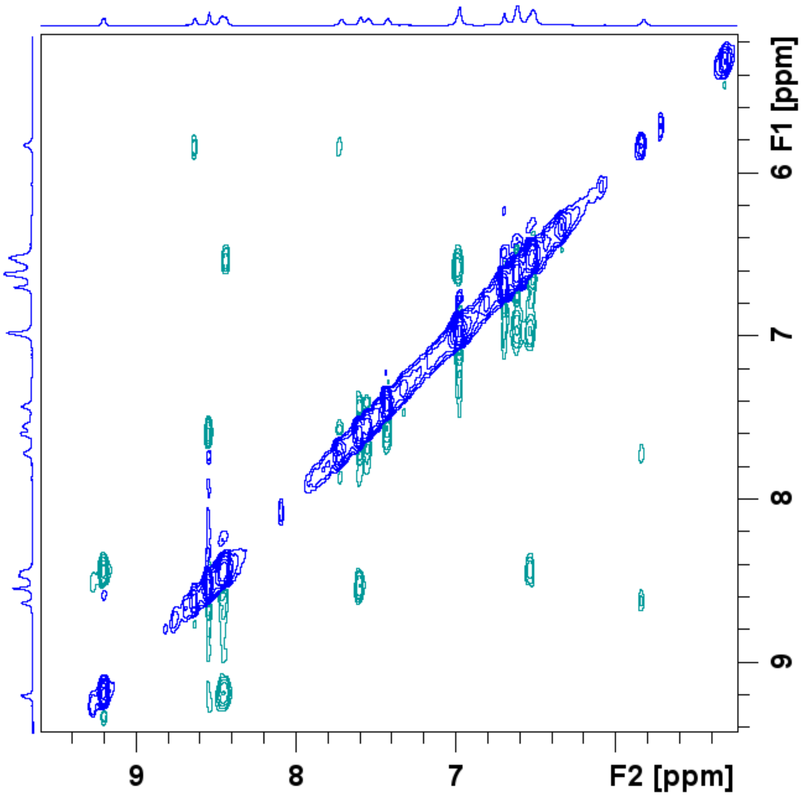

Figure S16. Partial ^{1}H ROESY NMR spectrum of [**PDI57**-H]$^{-}$ in THF-d_8, 190 K.

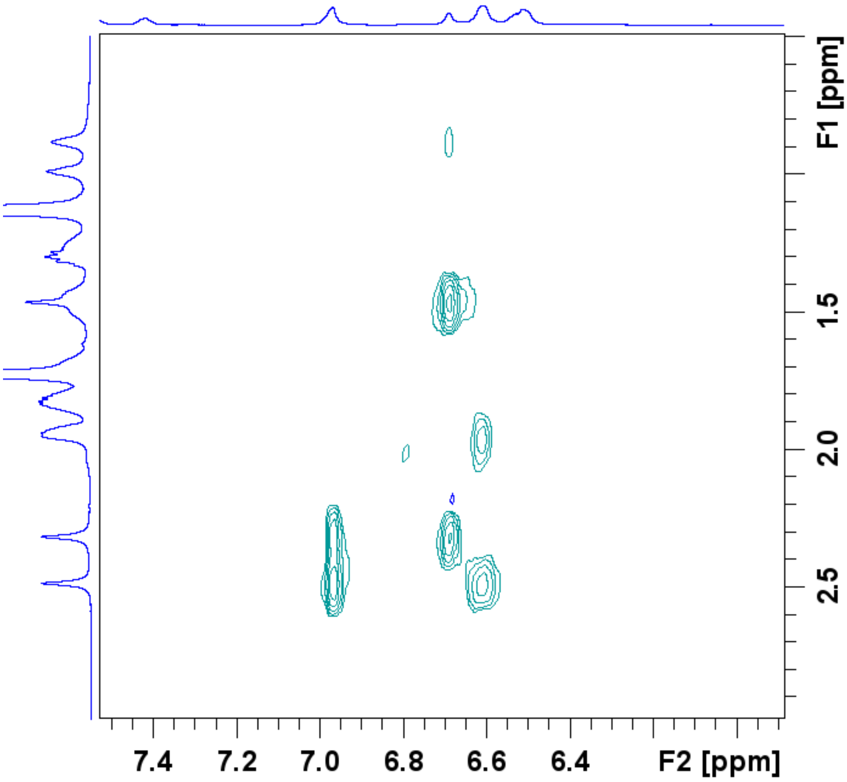

Figure S17. Partial ^{1}H ROESY NMR spectrum of [**PDI57**-H]$^-$ in THF-d_8, 190 K.

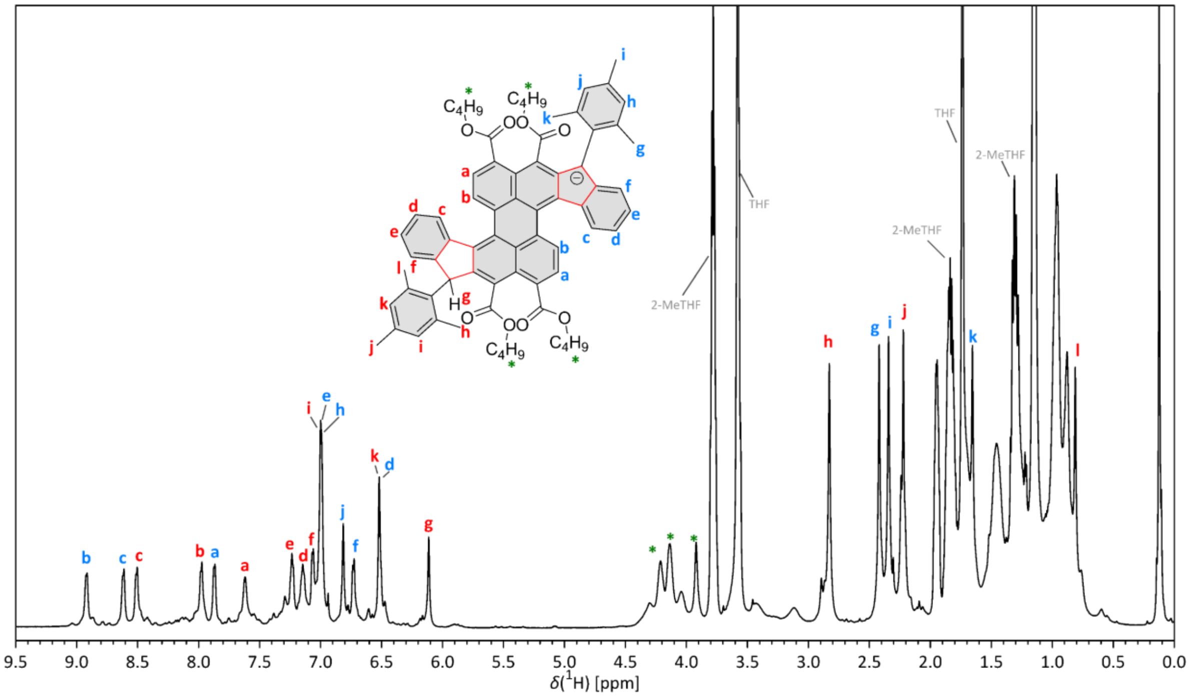

Figure S18. ^{1}H NMR spectrum (600 MHz, THF-d_8, 190 K) of [**PTE55**-H]$^-$.

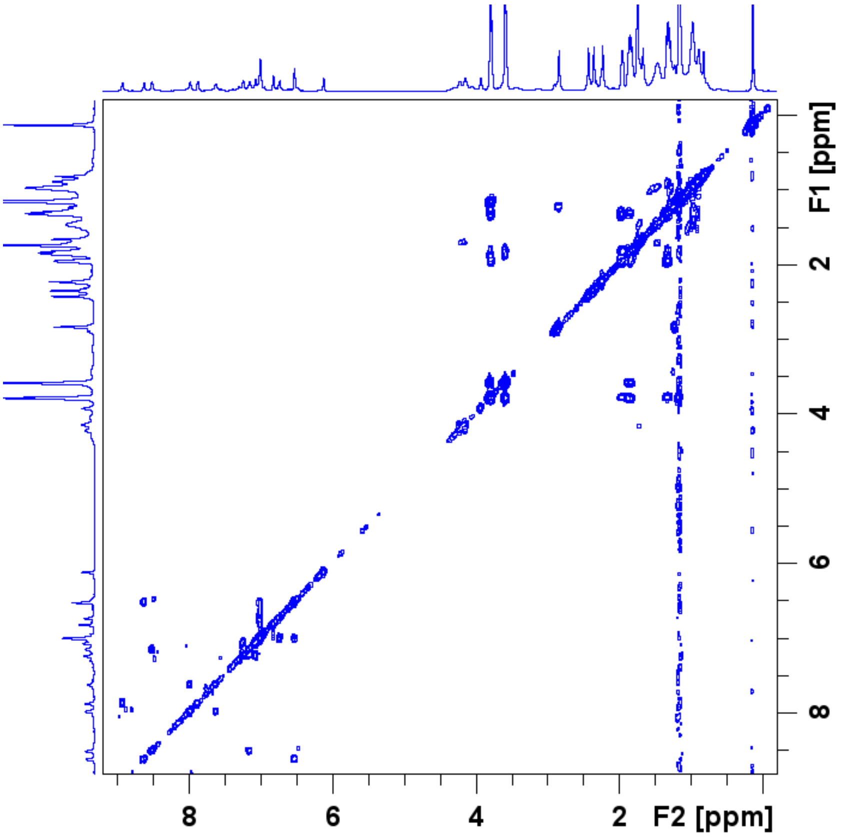

Figure S19. ^{1}H COSY NMR spectrum of [**PTE55**-H]$^{-}$ in THF-d_8, 190 K..

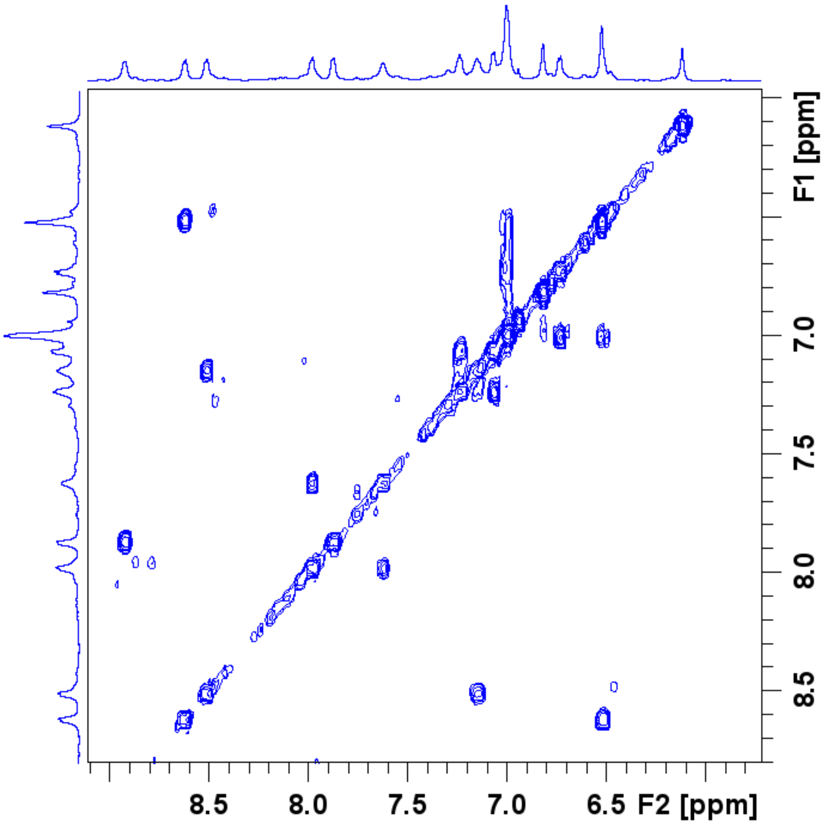

Figure S20. Partial ^{1}H COSY NMR spectrum of [**PTE55**-H]$^{-}$ in THF-d_8, 190 K.

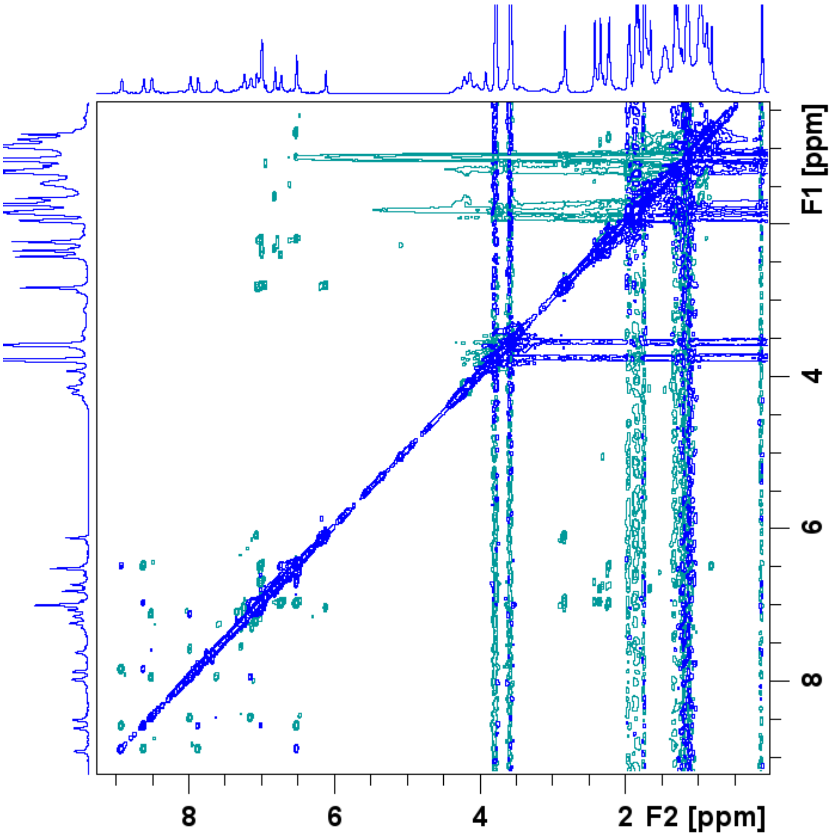

Figure S21. ^{1}H ROESY NMR spectrum of [**PTE55**-H]$^{-}$ in THF-d_8, 190 K.

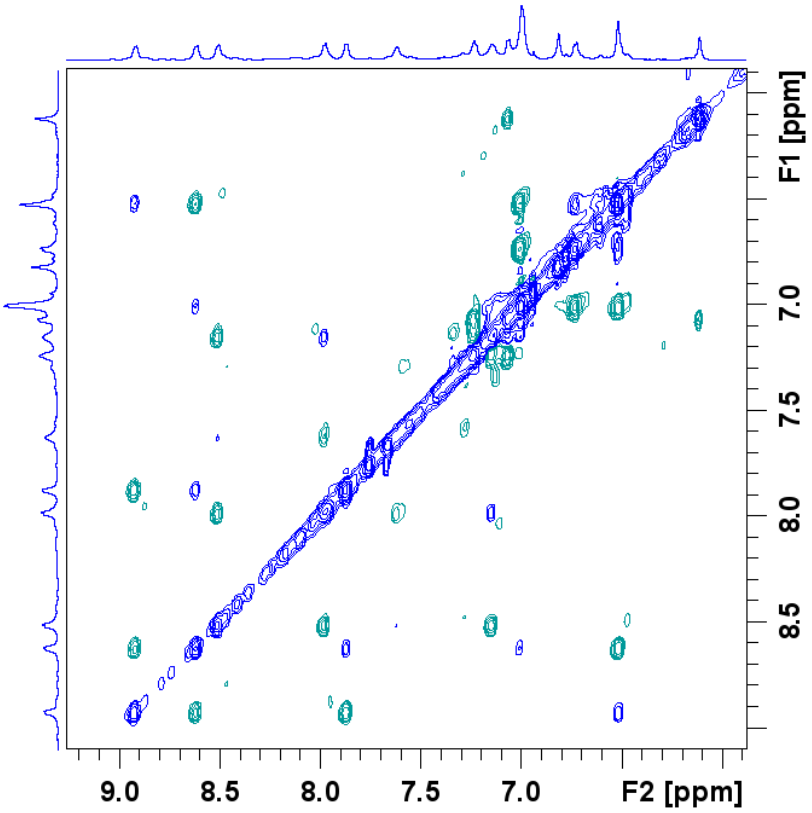

Figure S22. Partial ^{1}H ROESY NMR spectrum of [**PTE55**-H]$^{-}$ in THF-d_8, 190 K.

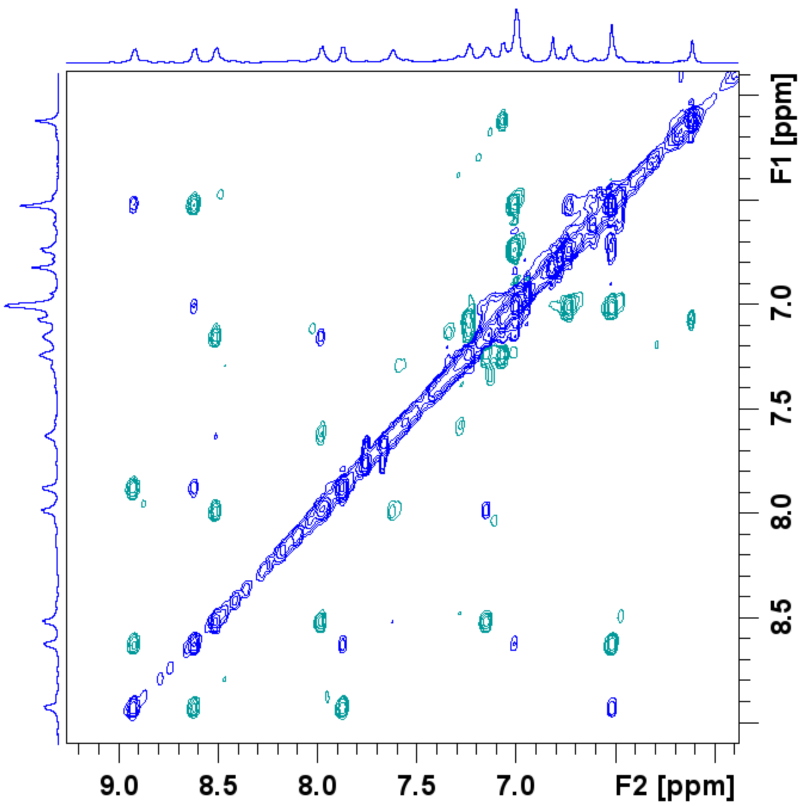

Figure S23. Partial ^{1}H ROESY NMR spectrum of [**PTE55**-H]$^{-}$ in THF-d_8, 190 K.

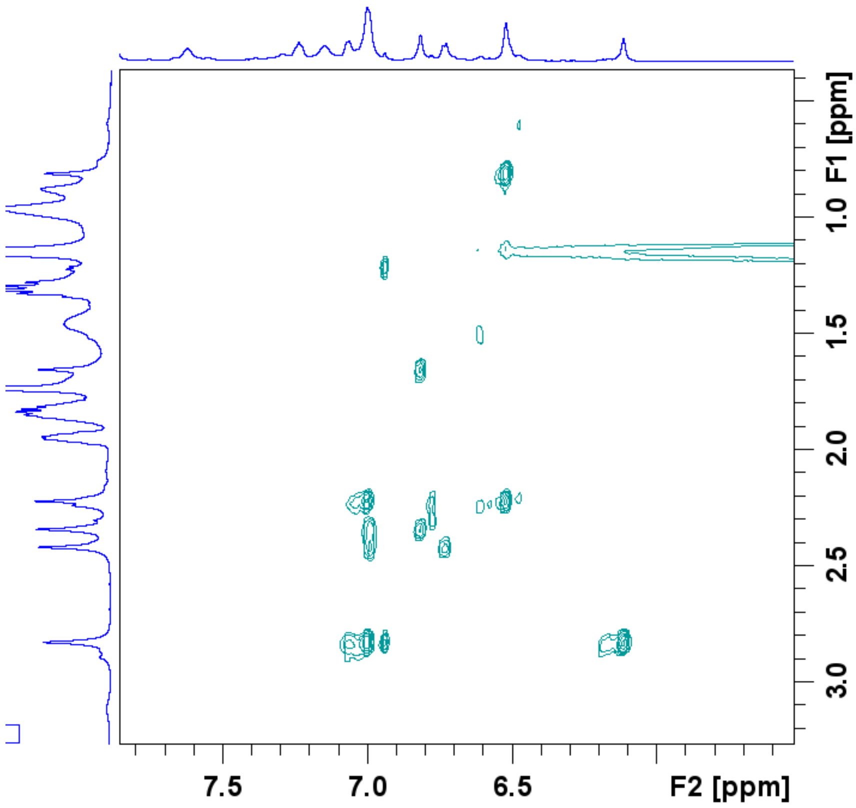

Figure S24. Partial ^{1}H ROESY NMR spectrum of [**PTE55**-H]$^{-}$ in THF-d_8, 190 K.

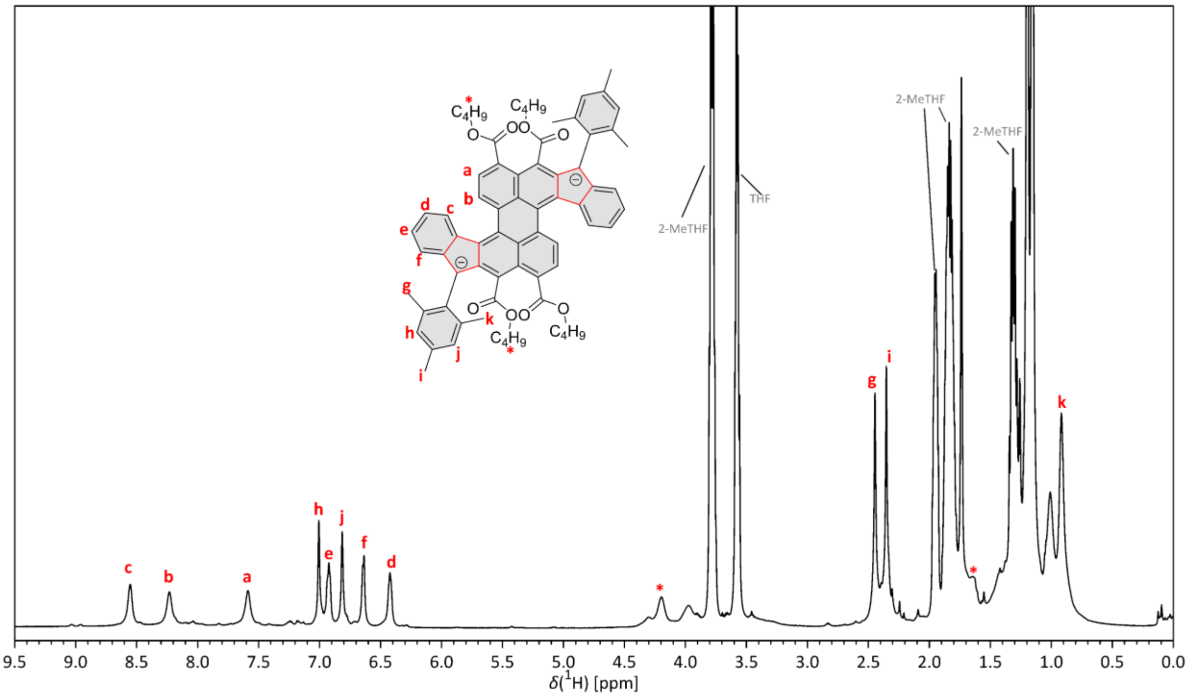

Figure S25. ^{1}H NMR spectrum (600 MHz, THF-d_8, 190 K) of [**PTE55**]$^{2-}$.

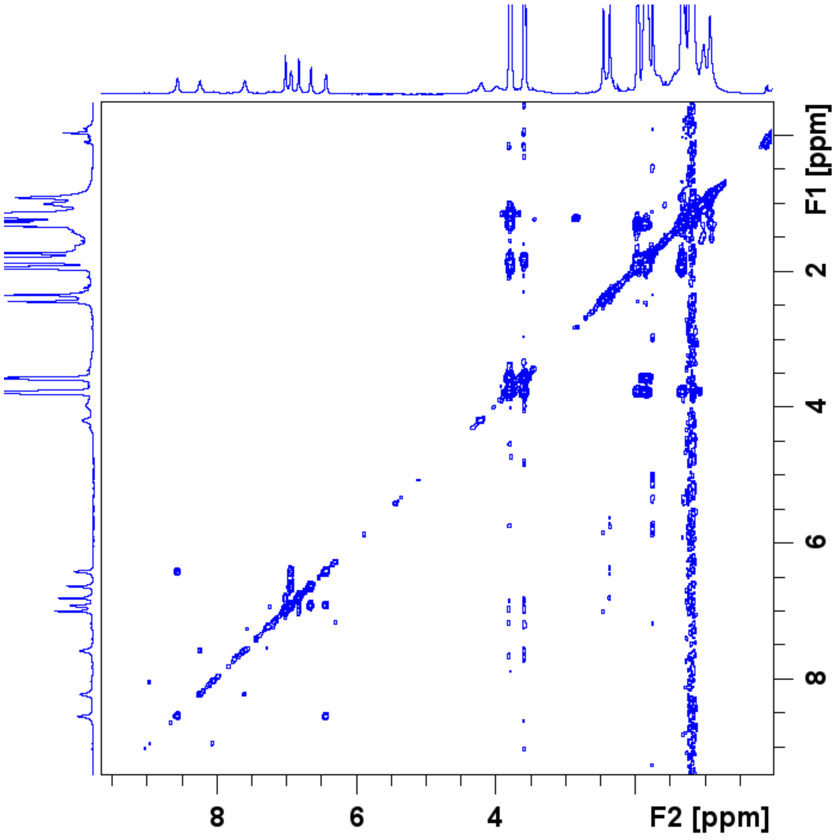

Figure S26. ^{1}H COSY NMR spectrum of [**PTE55**]$^{2-}$ in THF-d_8, 190 K.

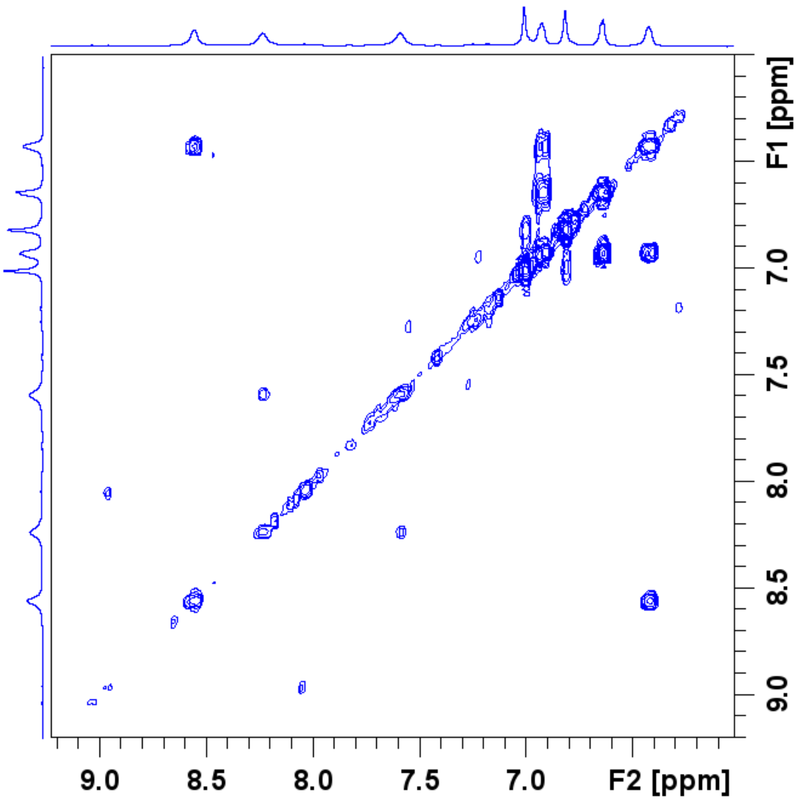

Figure S27. Partial ^{1}H COSY NMR spectrum of [**PTE55**]$^{2-}$ in THF-d_8, 190 K.

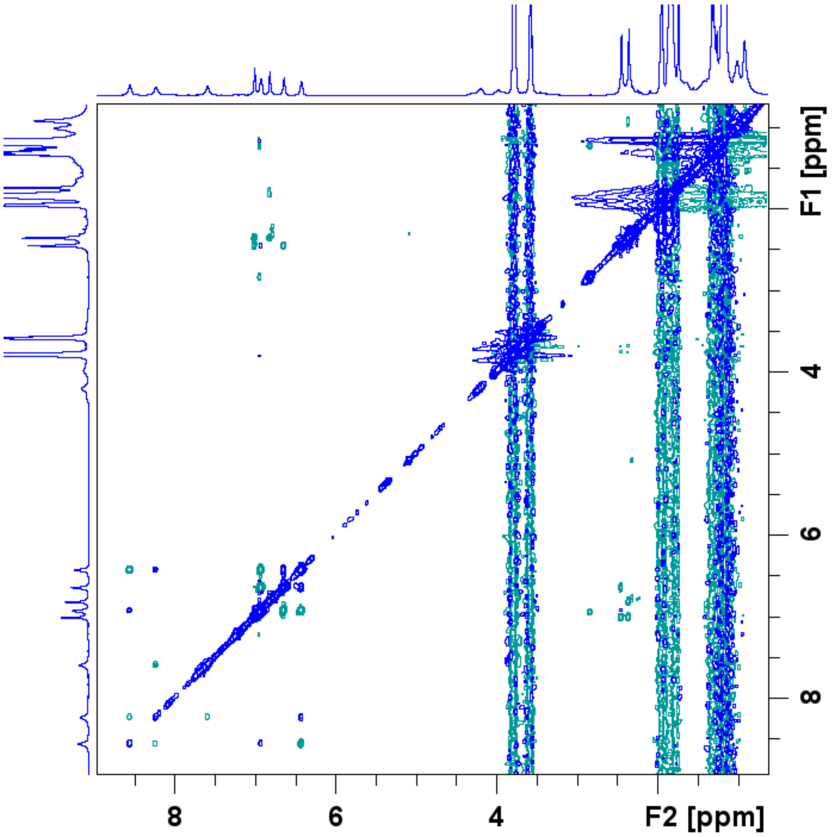

Figure S28. ^{1}H ROESY NMR spectrum of [**PTE55**]$^{2-}$ in THF-d_8, 190 K.

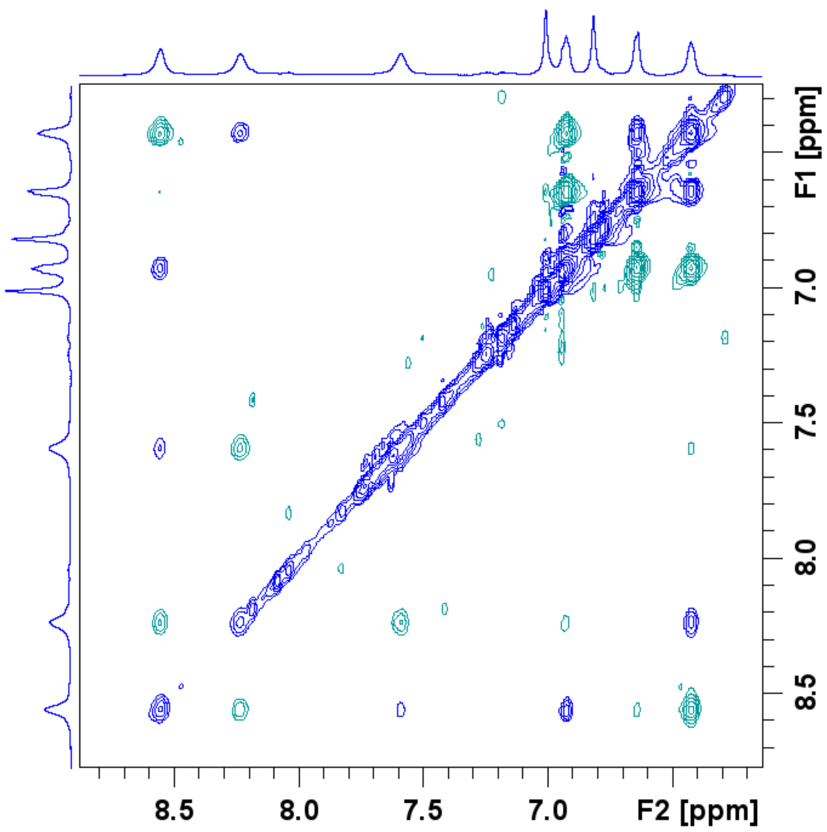

Figure S29. Partial ^{1}H ROESY NMR spectrum of [**PTE55**]$^{2-}$ in THF-d_8, 190 K.

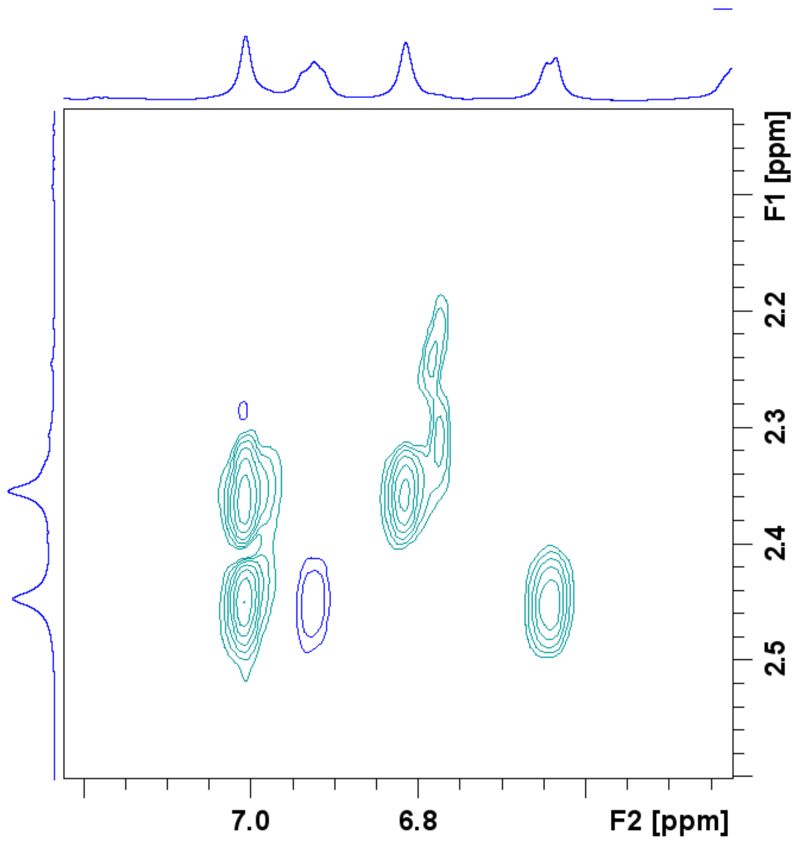

Figure S30. Partial ^{1}H ROESY NMR spectrum of [**PTE55**]$^{2-}$ in THF-d_8, 190 K.

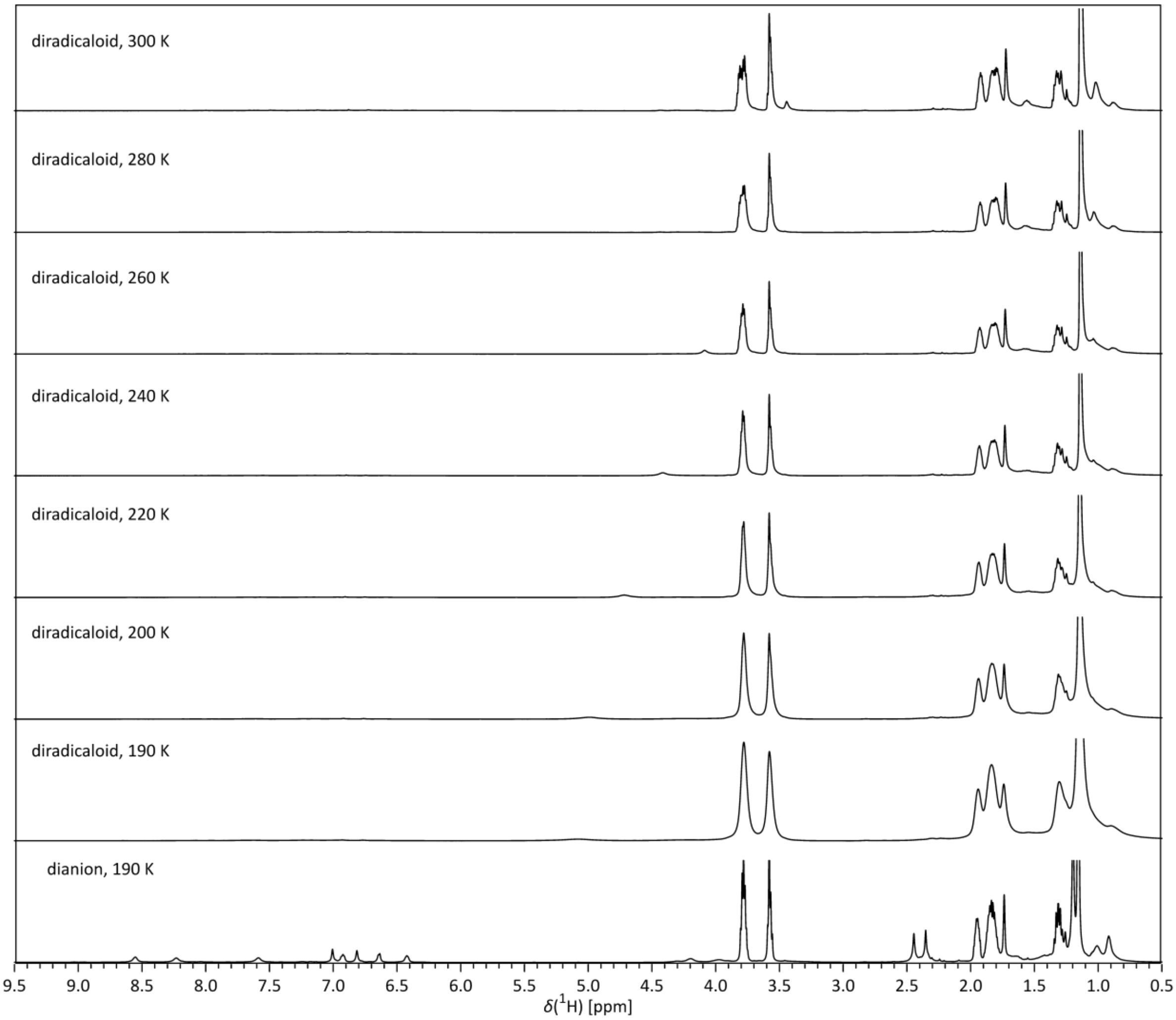

Figure S31. VT ^{1}H NMR (THF-d_8, 600 MHz) of [**PTE55**]$^{\bullet\bullet}$ generated in situ from [**PTE55**]$^{2-}$ by addition of iodine, 190 – 300 K. Spectra are scaled to the same intensity of solvent signal.

3.3. Absorption Spectroscopy

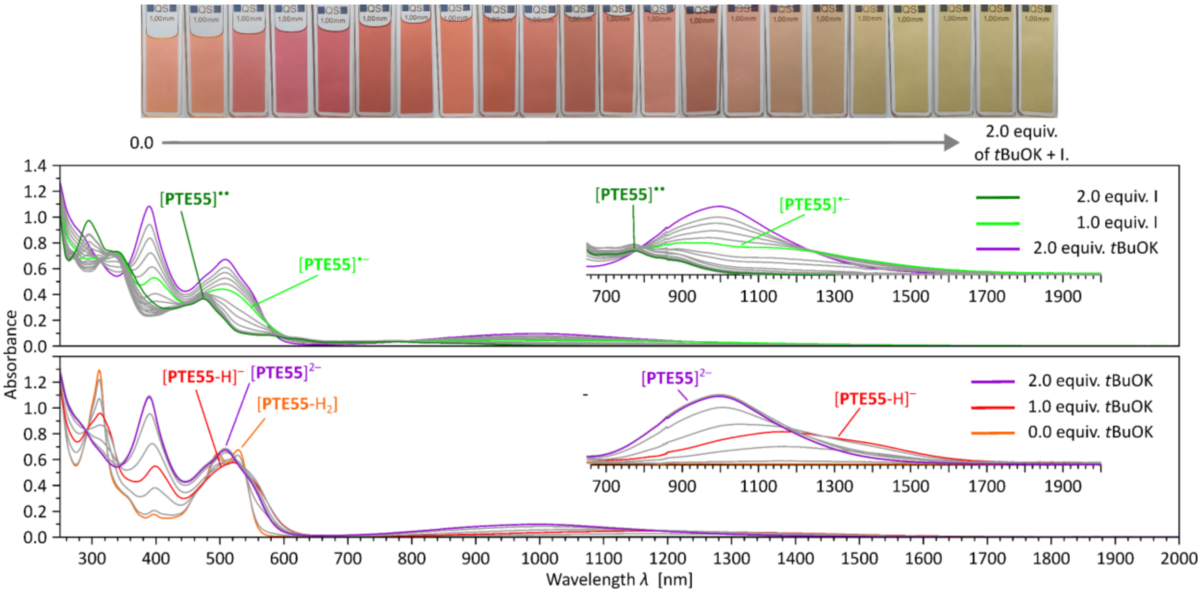

Figure S32. UV–vis–NIR spectra of [**PTE55**-H_2] (0.5 mM, THF, 18c6 **not added**) titrated with *t*BuOK (4 mM, THF) and iodine solutions (1.5 mM, THF).

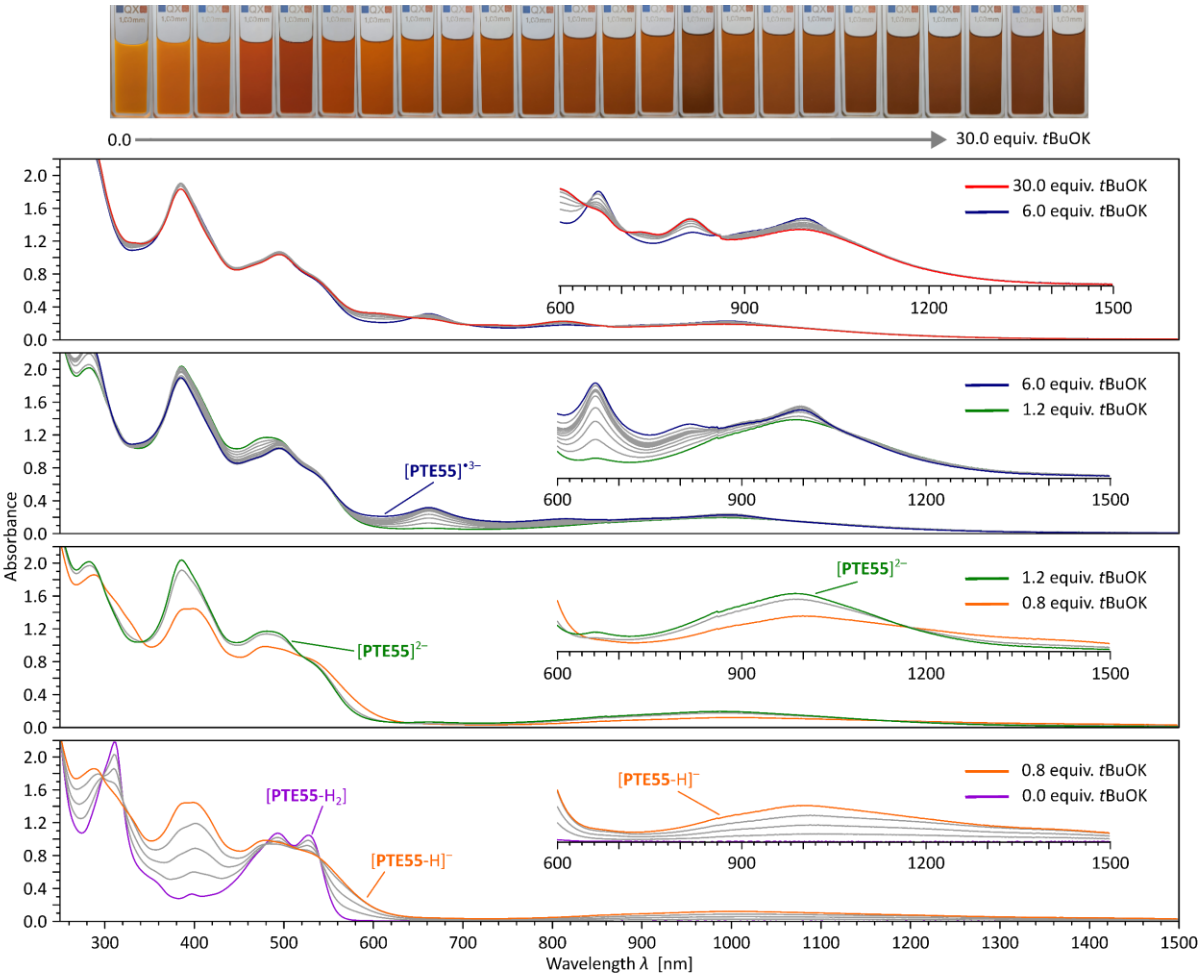

Figure S33. UV–vis–NIR spectra of [**PTE55**-H$_2$] (0.7 mM, THF, 18c6) titrated with *t*BuOK (10 mM, THF). The evolution observed above 6 equiv tBuOK is presumed to originate from coorination of [K(18c6)]$^+$ to the dianion.

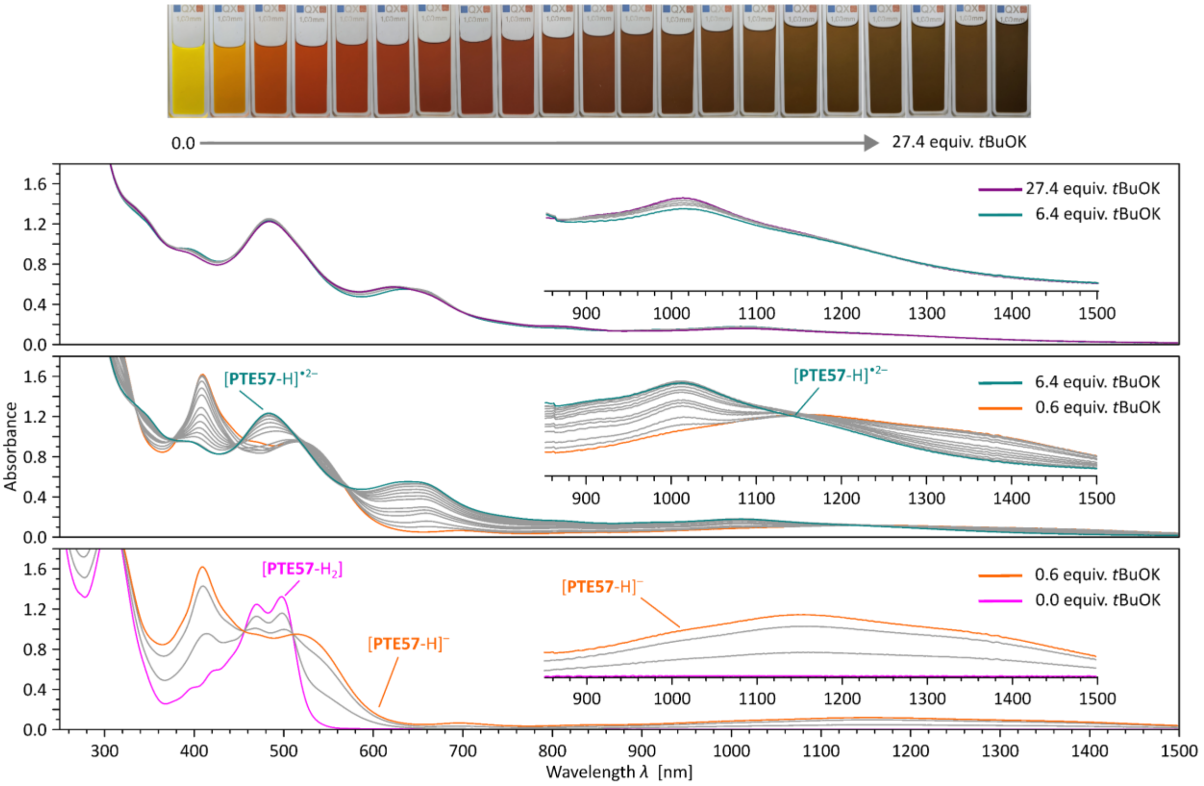

Figure S34. UV–vis–NIR spectra of [**PTE57**-H$_2$] (0.7 mM, THF, 18c6) titrated with *t*BuOK (10 mM, THF). The evolution observed above 6 equiv tBuOK is presumed to originate from coorination of [K(18c6)]$^+$ to the dianion.

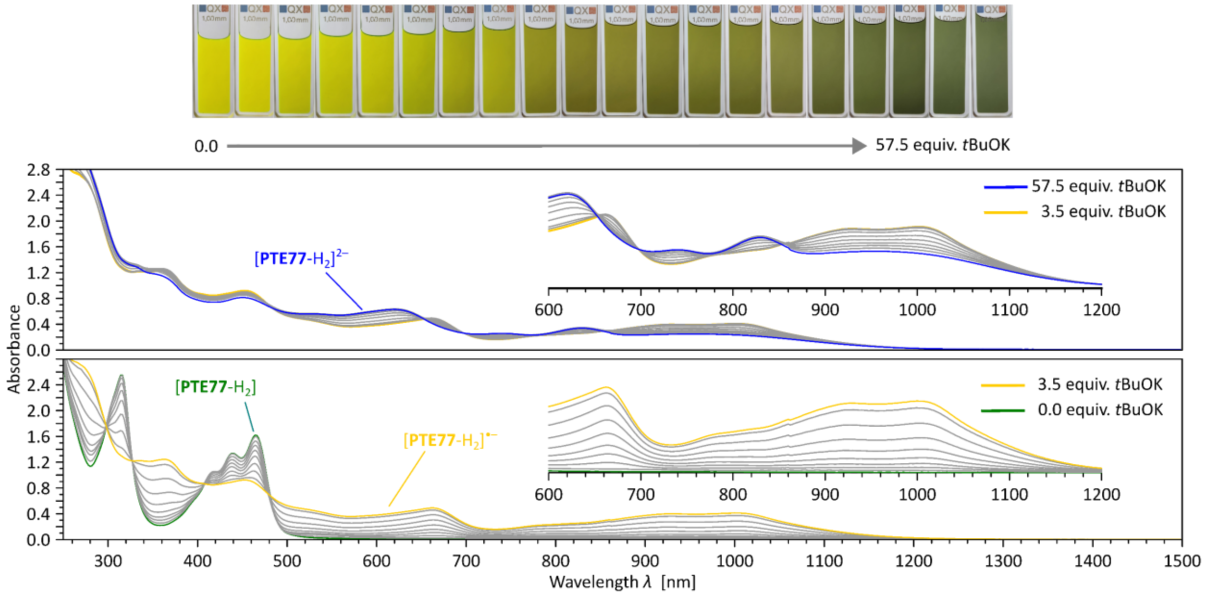

Figure S35. UV–vis–NIR spectra of [**PTE77**-H_2] (0.7 mM, THF, 18c6) titrated with *t*BuOK (10 mM, THF). Presented spectra do not correspond to pure radical anion and dianion (cf. spectroelectrochemistry data in Figure S50. For the corresponding TD-DFT simulations, see Figure S98.

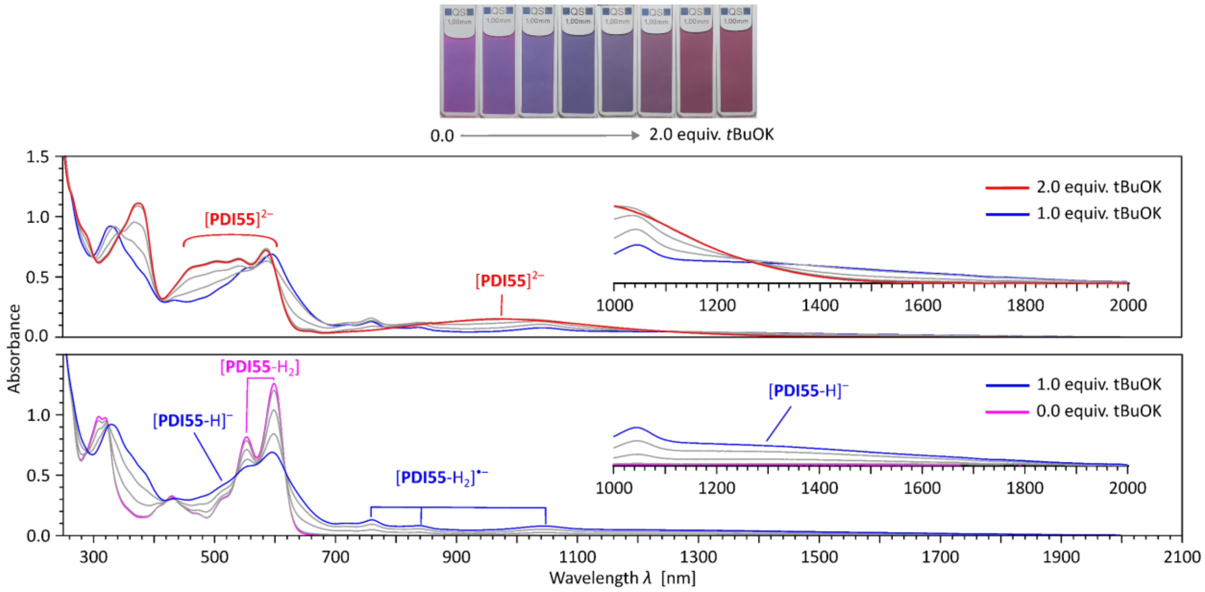

Figure S36. UV–vis–NIR spectra of [**PDI55**-H_2] (0.13 mM, THF) titrated with *t*BuOK (2 mM, THF) without addition of 18c6.

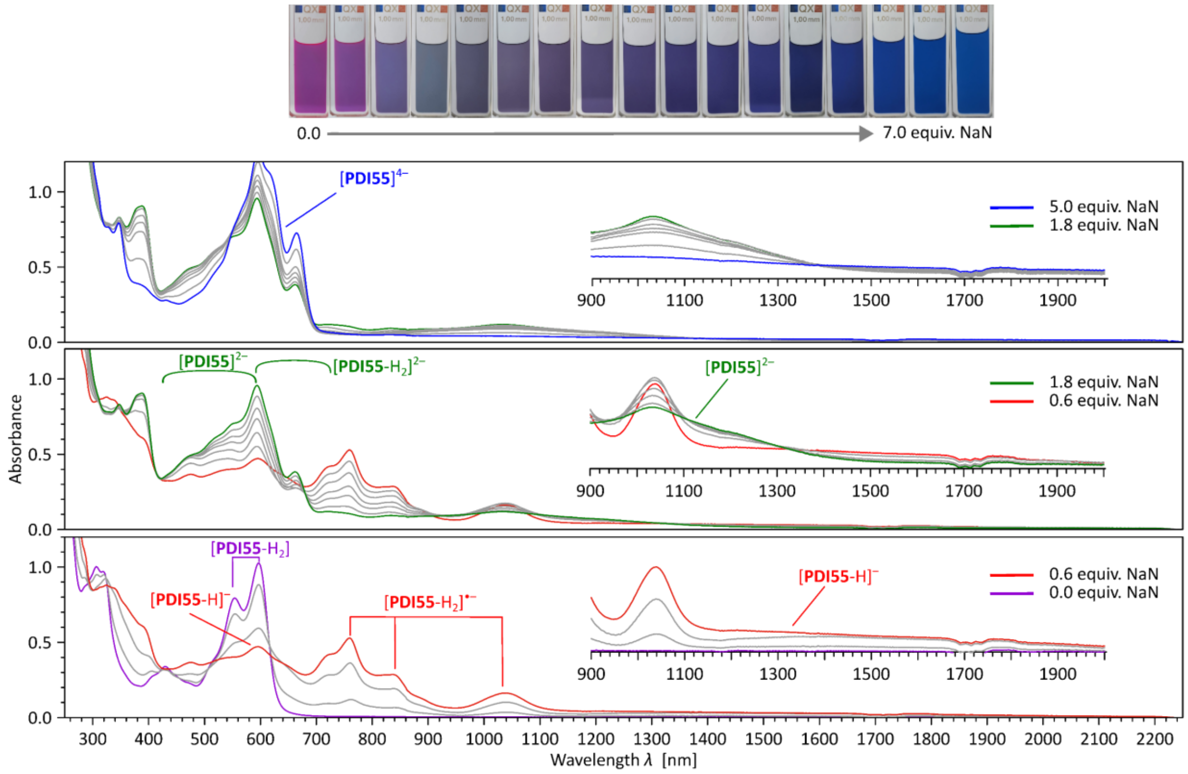

Figure S37. UV–vis–NIR spectra of [**PDI55**-H_2] (0.5 mM, THF, 15-crown-5) titrated with NaN (8.9 mM, THF).

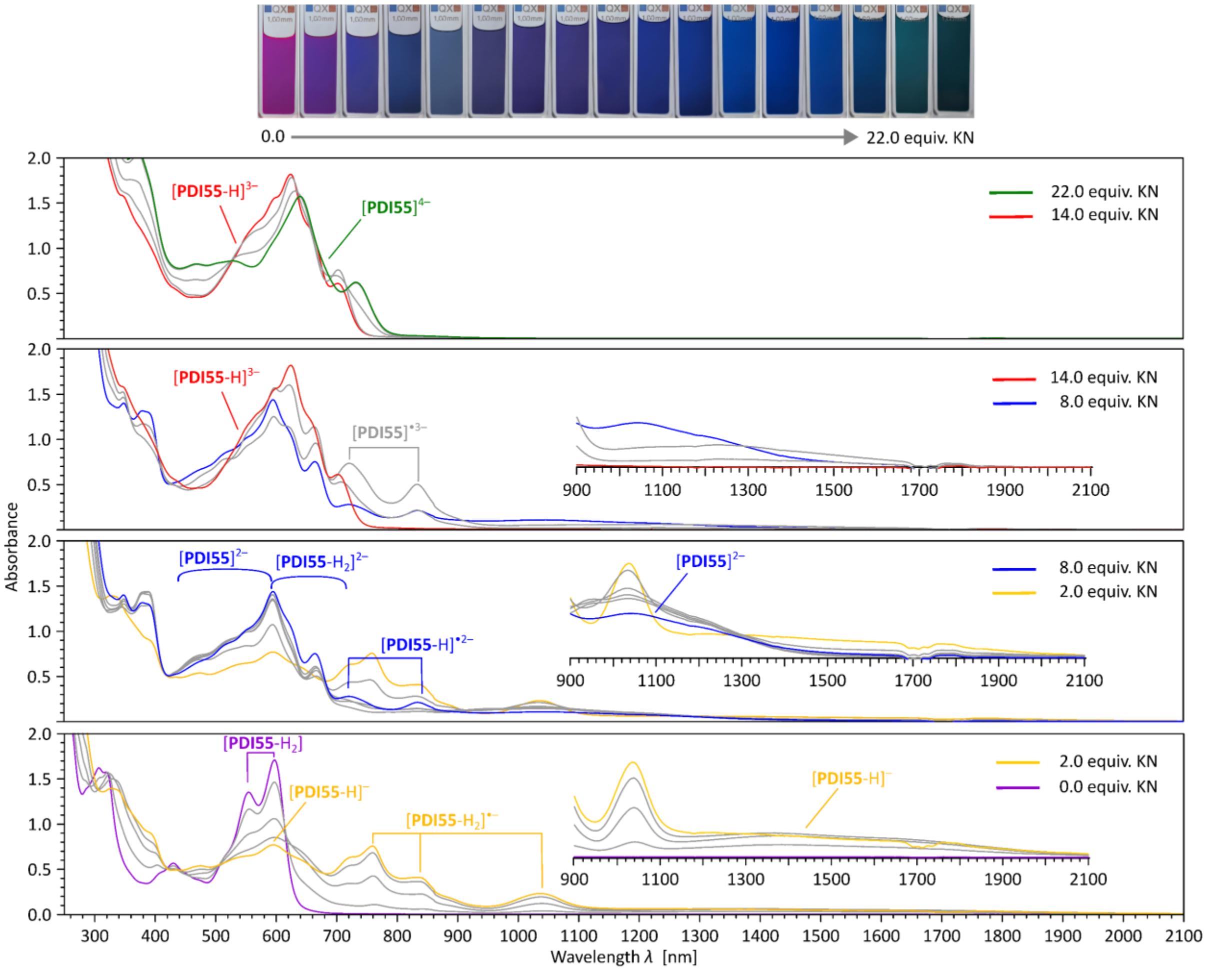

Figure S38. UV–vis–NIR spectra of [**PDI55**-H_2] (0.7 mM, THF, 18c6) titrated with KN (18.3 mM, THF).

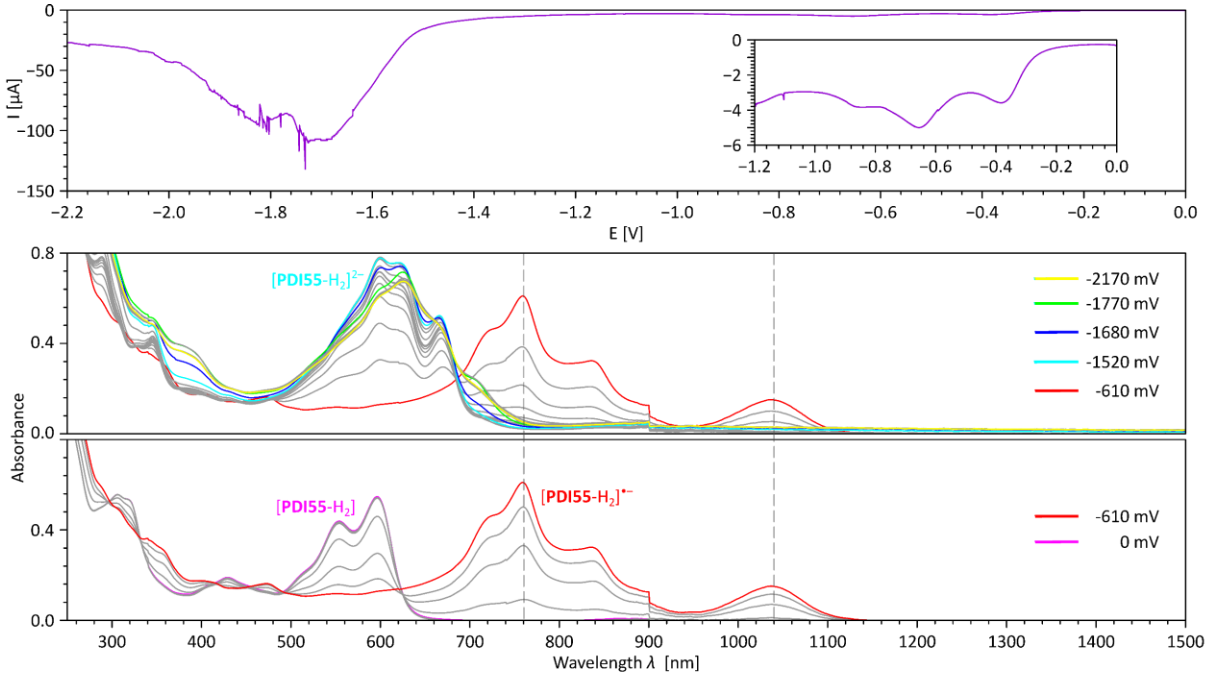

Figure S39. Spectroelectrochemistry of [**PDI55**-H$_2$]. Two bottom spectra – UV–vis–NIR spectra during measurement, top spectrum – electrochemical trace. Potentials measured vs. Ag/Ag$^+$ pseudoreference. Starting potential: 0 mV. The potentials reported for the individual absorption spectra are the applied values recorded at the start of acquisition of the respective spectra.

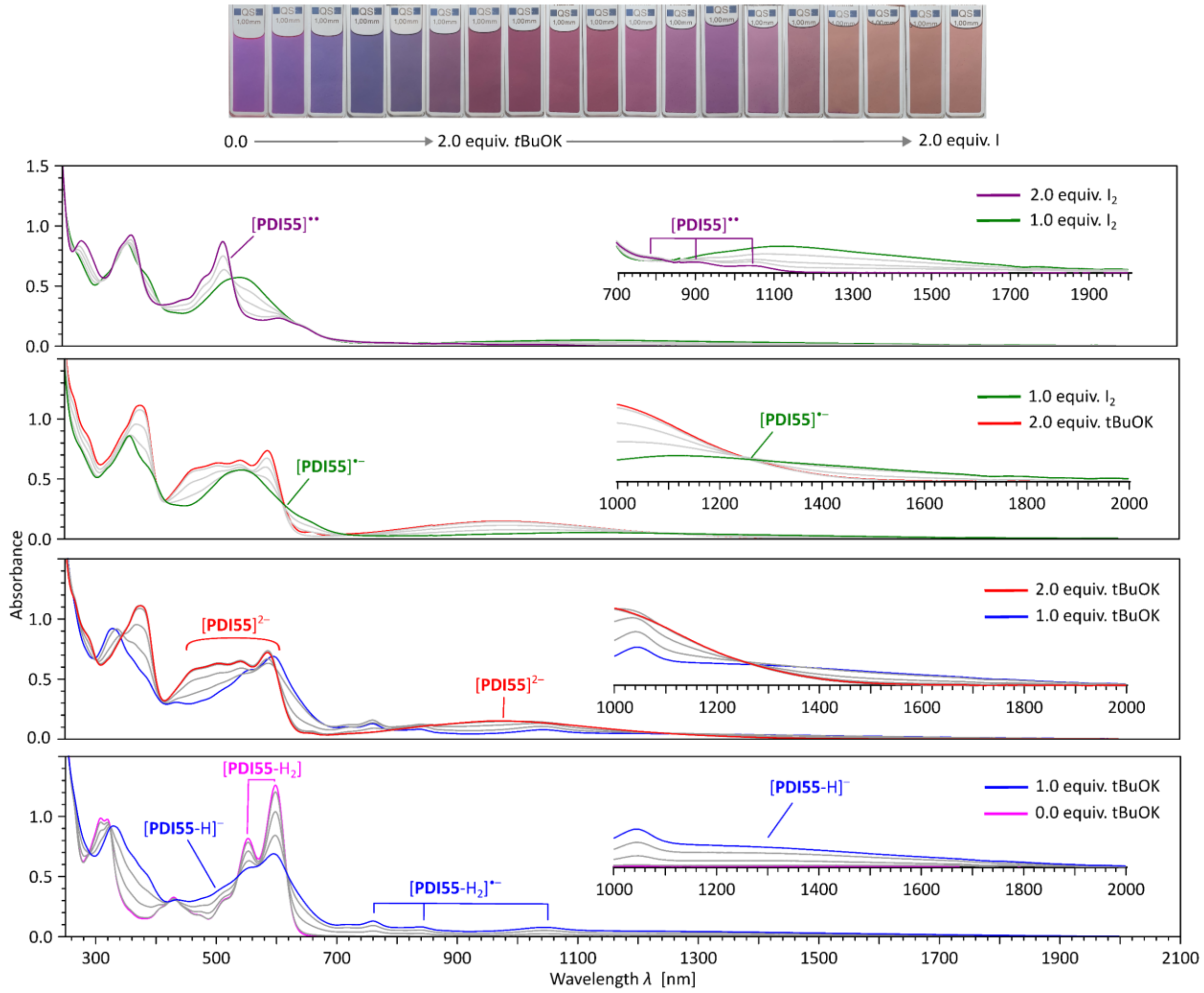

Figure S40. UV–vis–NIR spectra of [**PDI55**-H$_2$] (0.3 mM, THF, 18c6 **not added**) titrated with *t*BuOK (2 mM, THF) and I$_2$ (2 mM, THF).

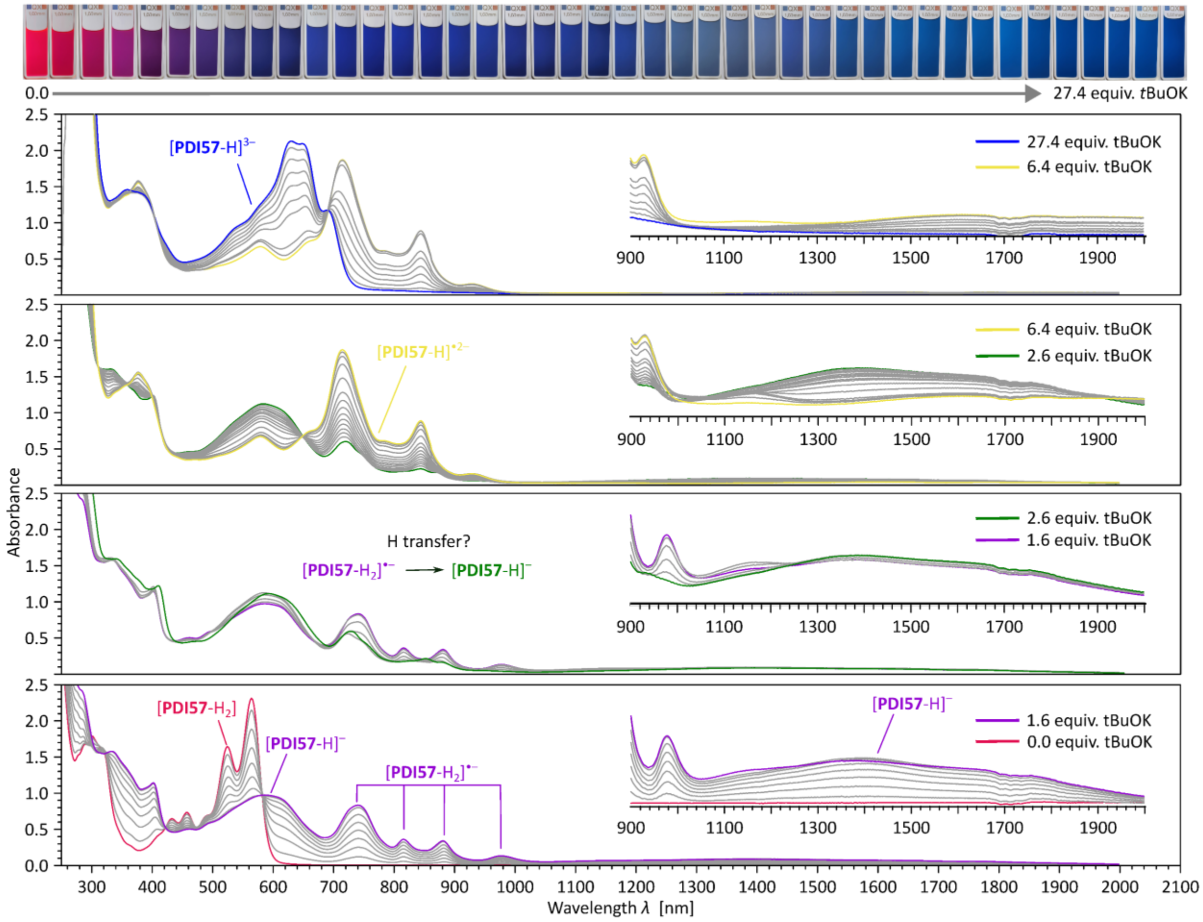

Figure S41. UV–vis–NIR spectra of [**PDI57**-H_2] (0.75 mM, THF, 18c6) titrated with *t*BuOK (10 mM, THF).

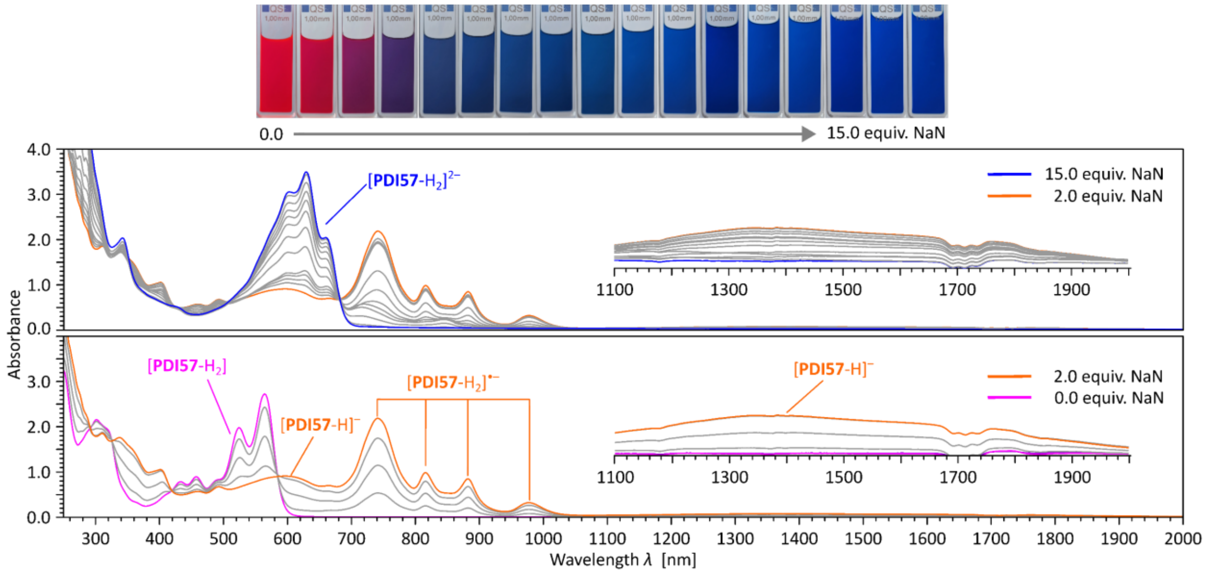

Figure S42. UV–vis–NIR spectra of [**PDI57**-H_2] (0.7 mM, THF, 15-crown-5) titrated with NaN (17.4 mM, THF).

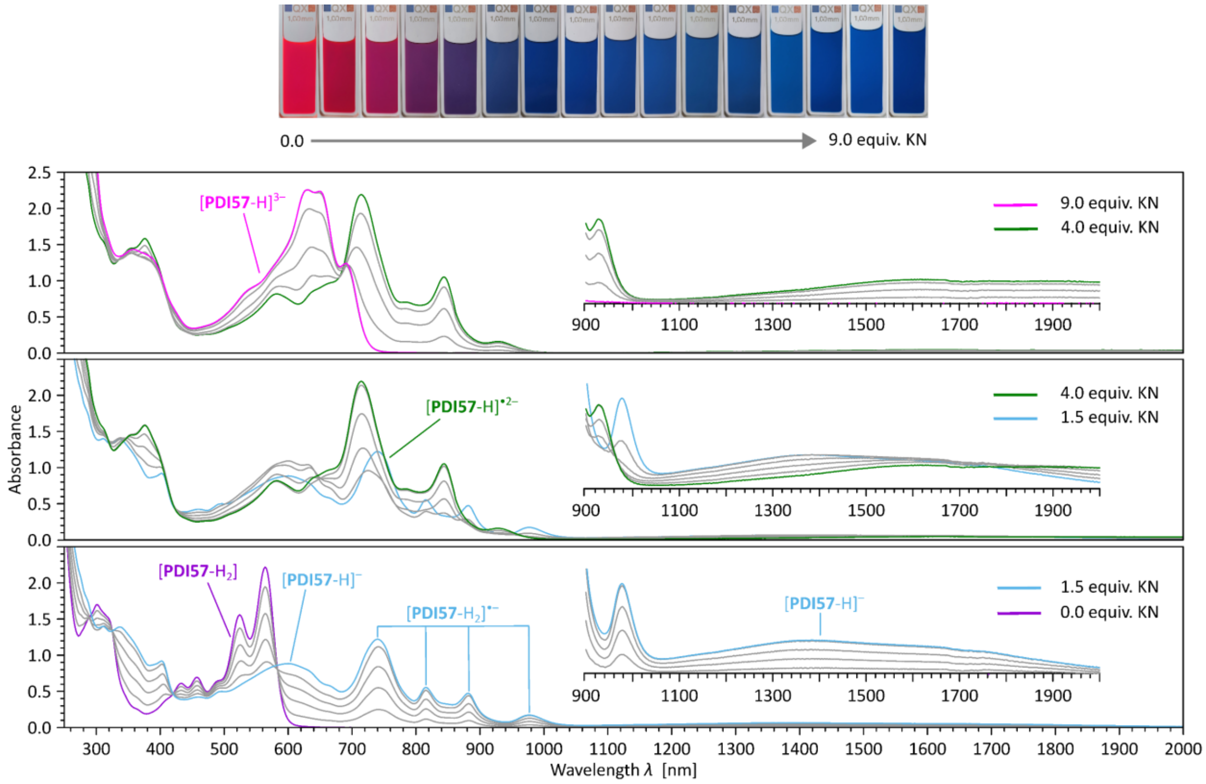

Figure S43. UV–vis–NIR spectra of [**PDI57**-H_2] (0.6 mM, THF,18c6) titrated with KN (18.4 mM, THF).

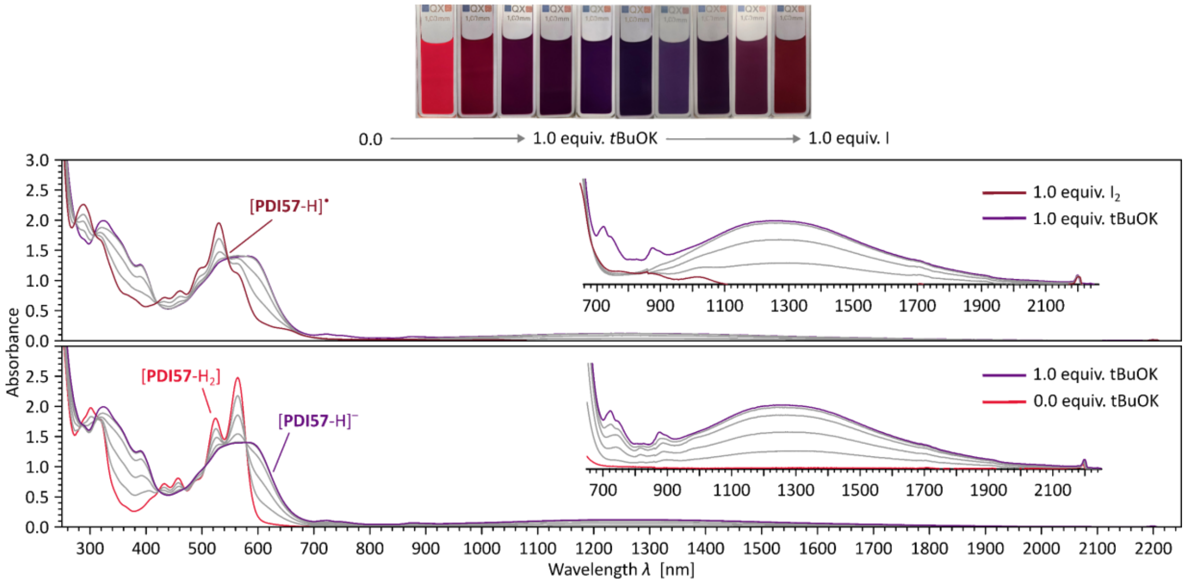

Figure S44. UV–vis–NIR spectra of [**PDI57**-H_2] (0.5 mM, THF, 18c6 **not added**) titrated with *t*BuOK (10 mM, THF) and I_2 (15 mM, THF).

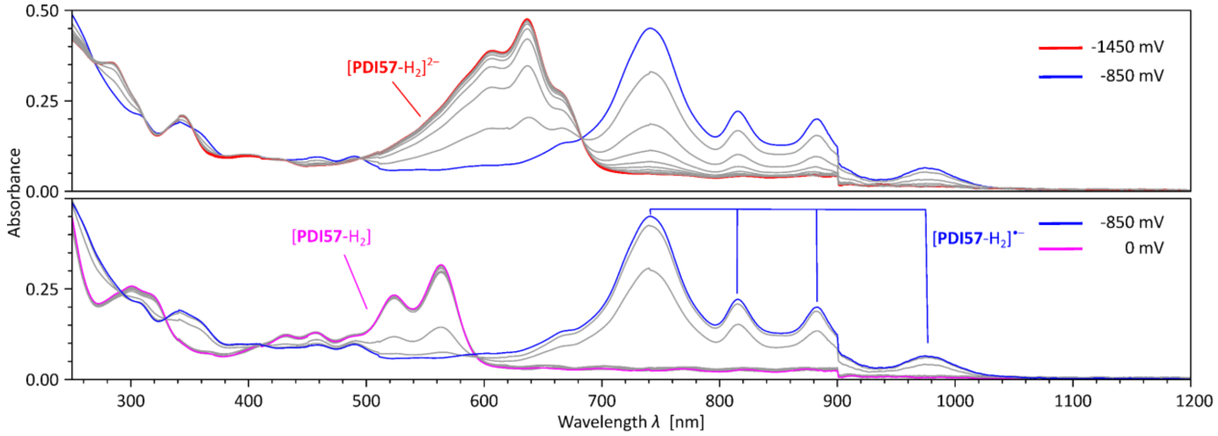

Figure S45. Spectroelectrochemistry of [**PDI57**-H_2]. Potentials measured vs. Ag/Ag^+ pseudoreference. Starting potential: 0 mV. The potentials reported for the individual absorption spectra are the applied values recorded at the start of acquisition of the respective spectra.

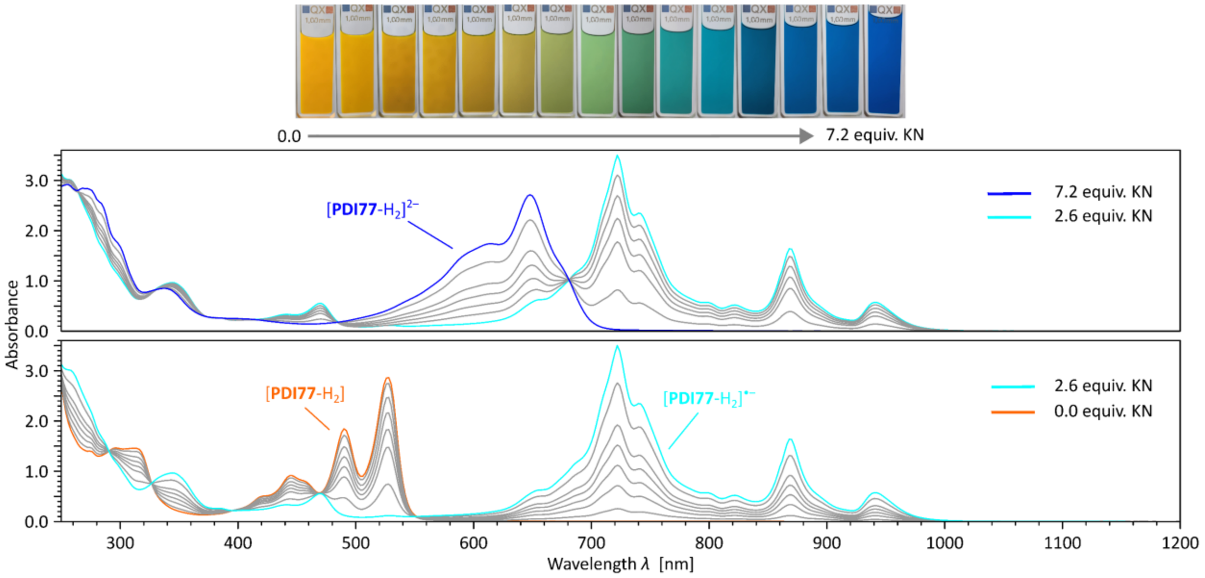

Figure S46. UV–vis–NIR spectra of [**PDI77**-H_2] (0.5 mM, THF, 18c6) titrated with KN (7.8 mM, THF).

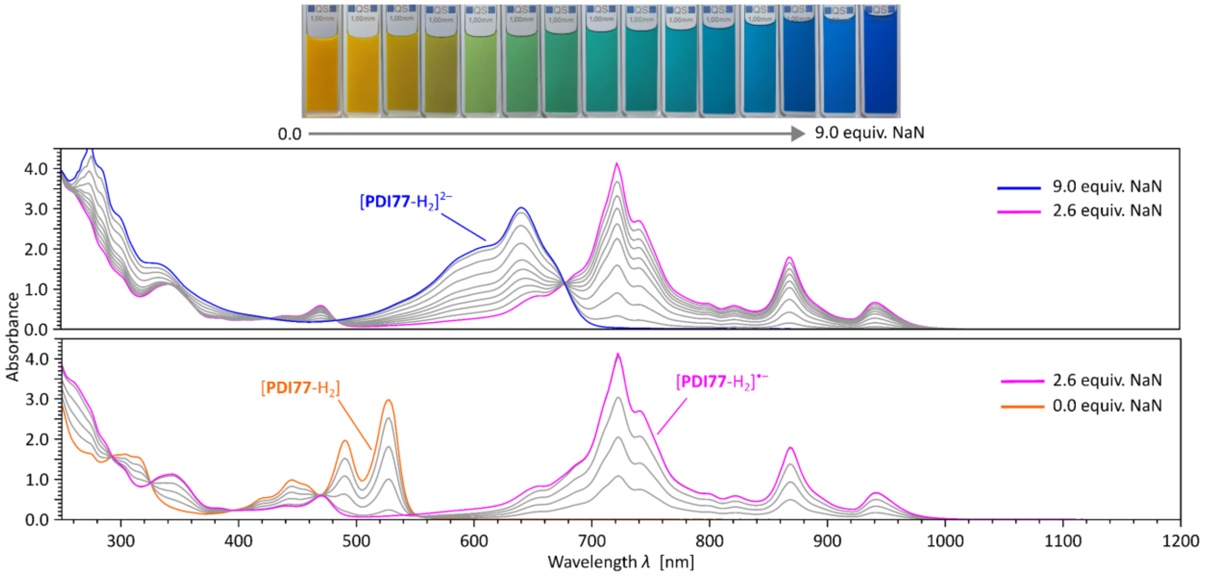

Figure S47. UV–vis–NIR spectra of [**PDI77**-H_2] (0.8 mM, THF, 15-crown-5) titrated with NaN (17 mM, THF).

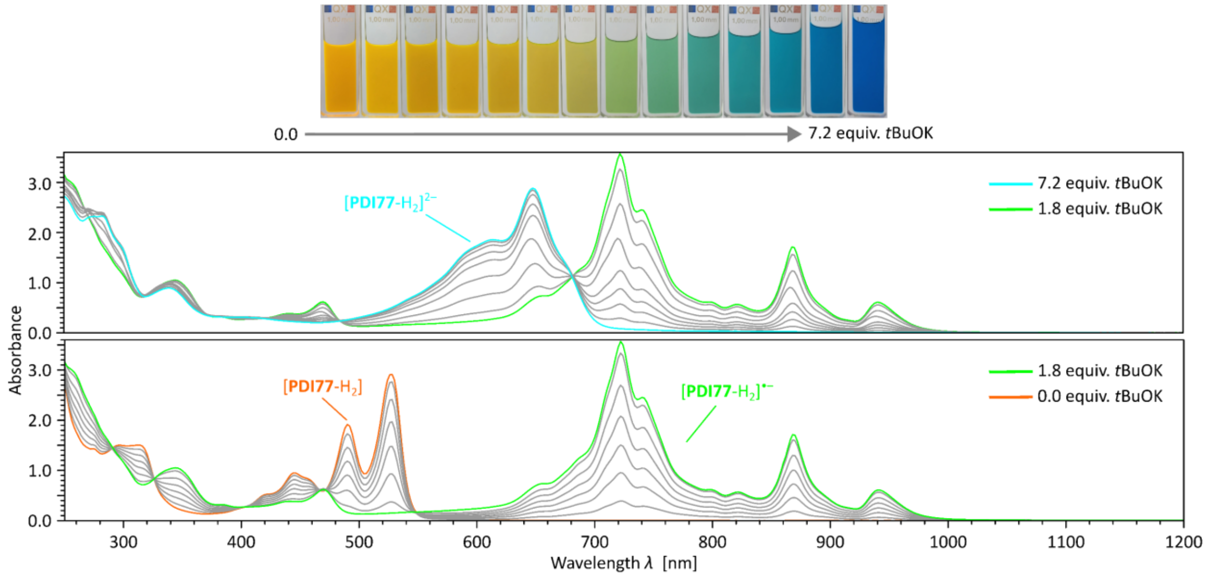

Figure S48. UV–vis–NIR spectra of [**PDI77**-H_2] (0.58 mM, THF, 18c6) titrated with *t*BuOK (10 mM, THF).

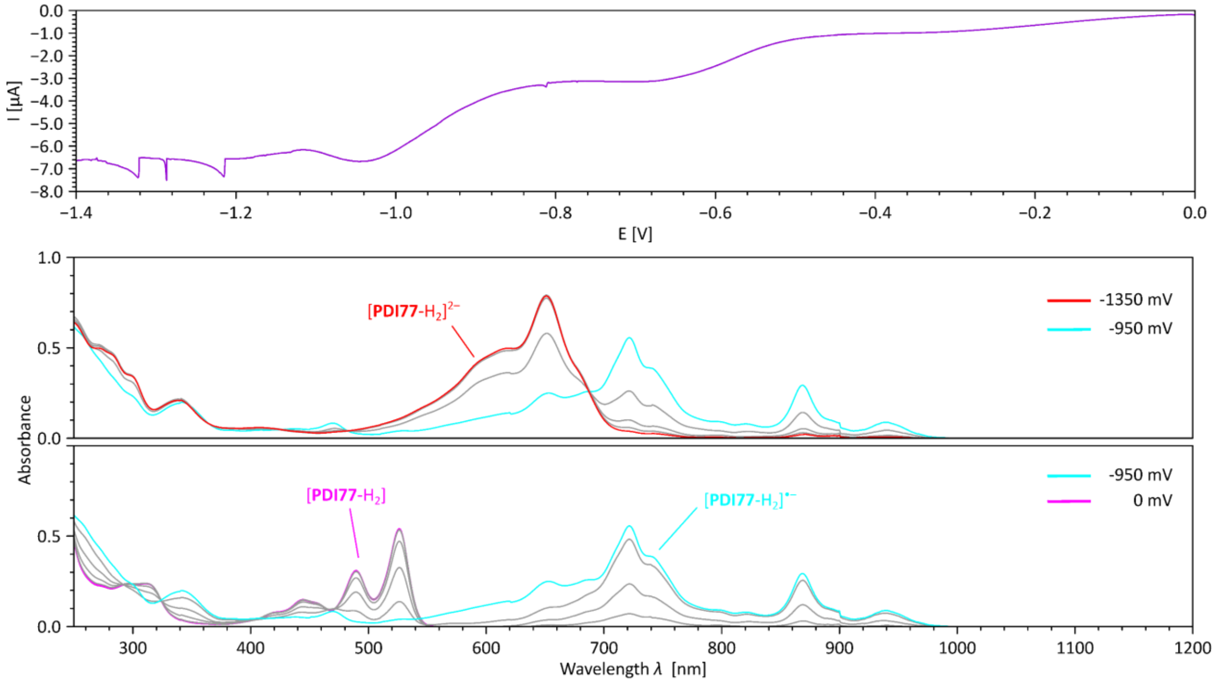

Figure S49. Spectroelectrochemistry of [**PDI77**-H_2]. Two bottom spectra – UV–vis–NIR spectra during measurement, top spectrum – pseudoreference. Starting potential: 0 mV. The potentials reported for the individual absorption spectra are the applied values recorded at the start of acquisition of the respective spectra.

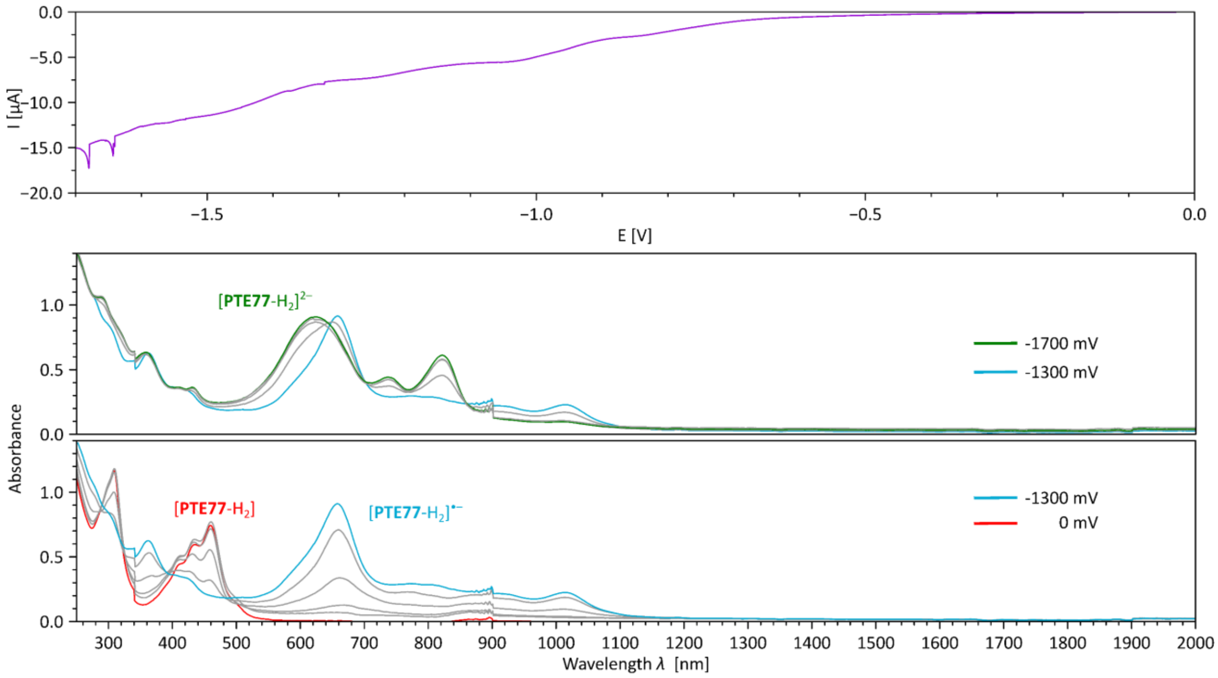

Figure S50. Spectroelectrochemistry of [**PTE77**-H$_2$]. Two bottom spectra – UV–vis–NIR spectra during measurement, top spectrum – electrochemical trace. Potentials measured vs. Ag/Ag$^+$ pseudoreference. Starting potential: 0 mV. The potentials reported for the individual absorption spectra are the applied values recorded at the start of acquisition of the respective spectra.

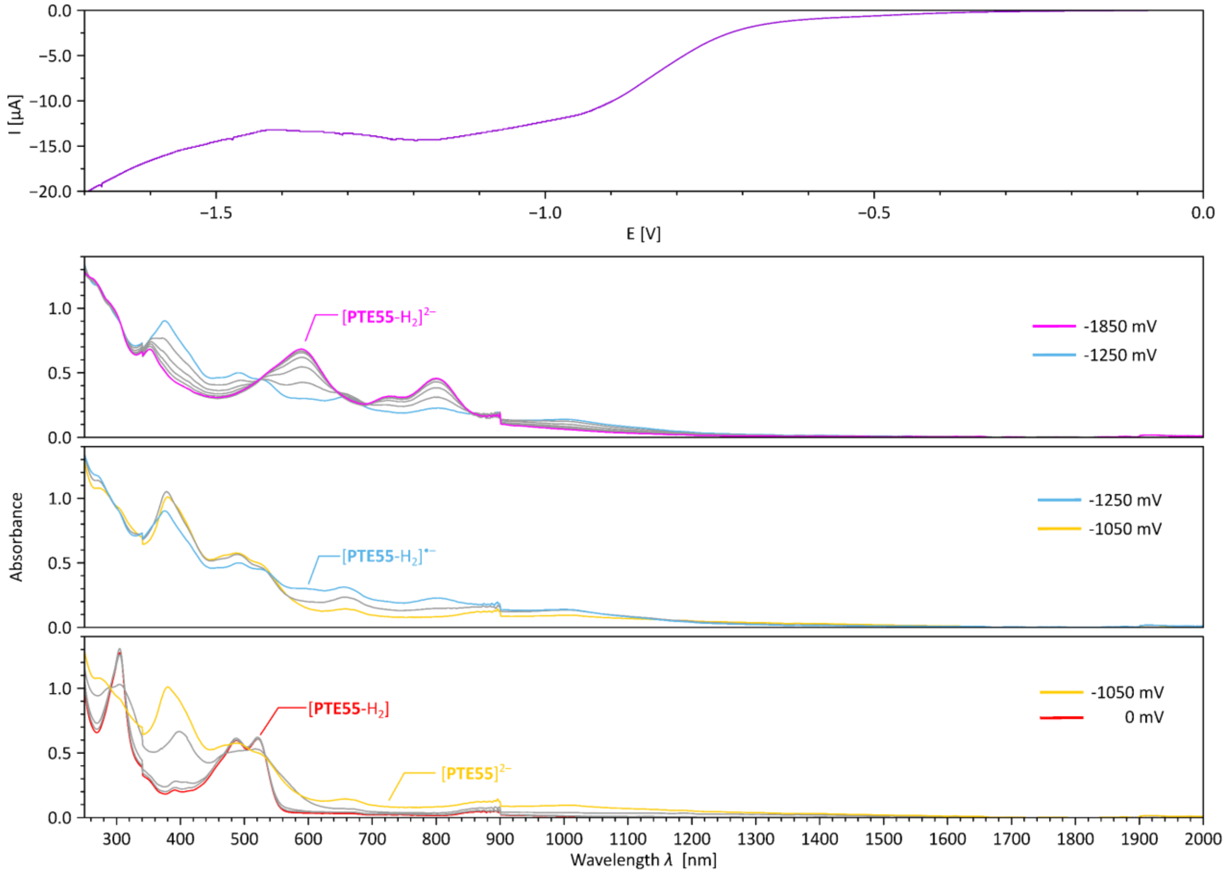

Figure S51. Spectroelectrochemistry of [**PTE55**-H$_2$]. Three bottom spectra – UV–vis–NIR spectra during measurement, top spectrum – electrochemical trace. Potentials measured vs. Ag/Ag$^+$ pseudoreference. Starting potential: 0 mV. The potentials reported for the individual absorption spectra are the applied values recorded at the start of acquisition of the respective spectra.

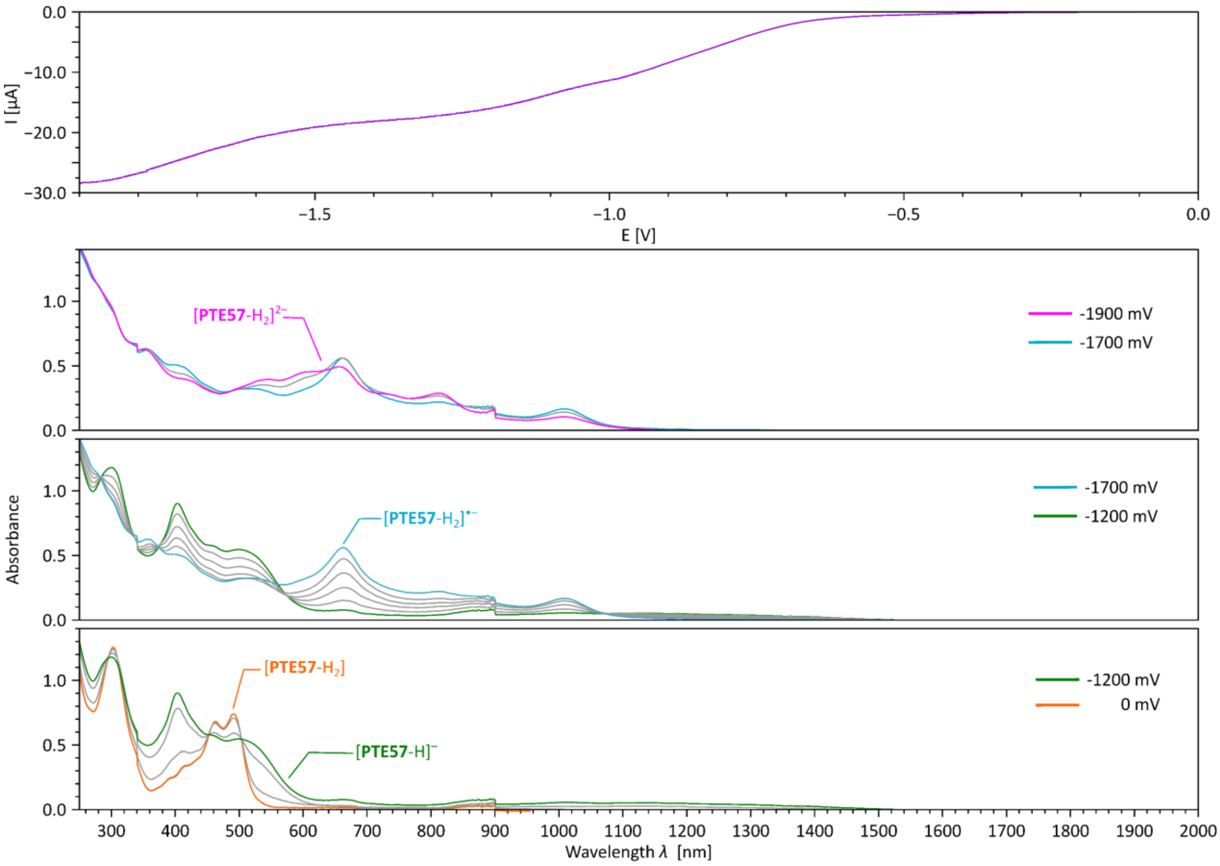

Figure S52. Spectroelectrochemistry of [**PTE57**-H_2]. Three bottom spectra – UV–vis–NIR spectra during measurement, top spectrum – electrochemical trace. Potentials measured vs. Ag/Ag^+ pseudoreference. Starting potential: 0 mV. The potentials reported for the individual absorption spectra are the applied values recorded at the start of acquisition of the respective spectra.

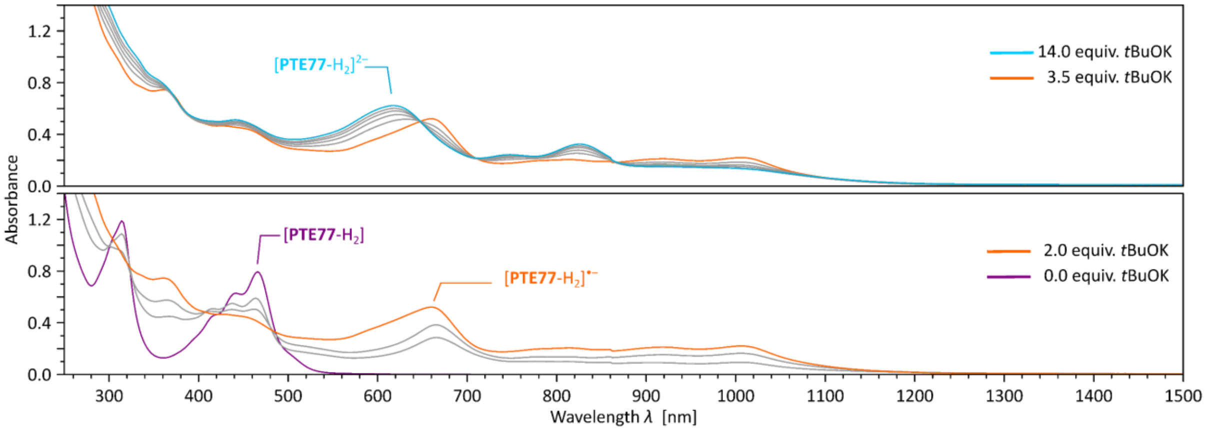

Figure S53. UV–vis–NIR spectra of [**PTE77**-H_2] (0.4 mM, THF, 18c6) titrated with *t*BuOK (400 mM, THF).

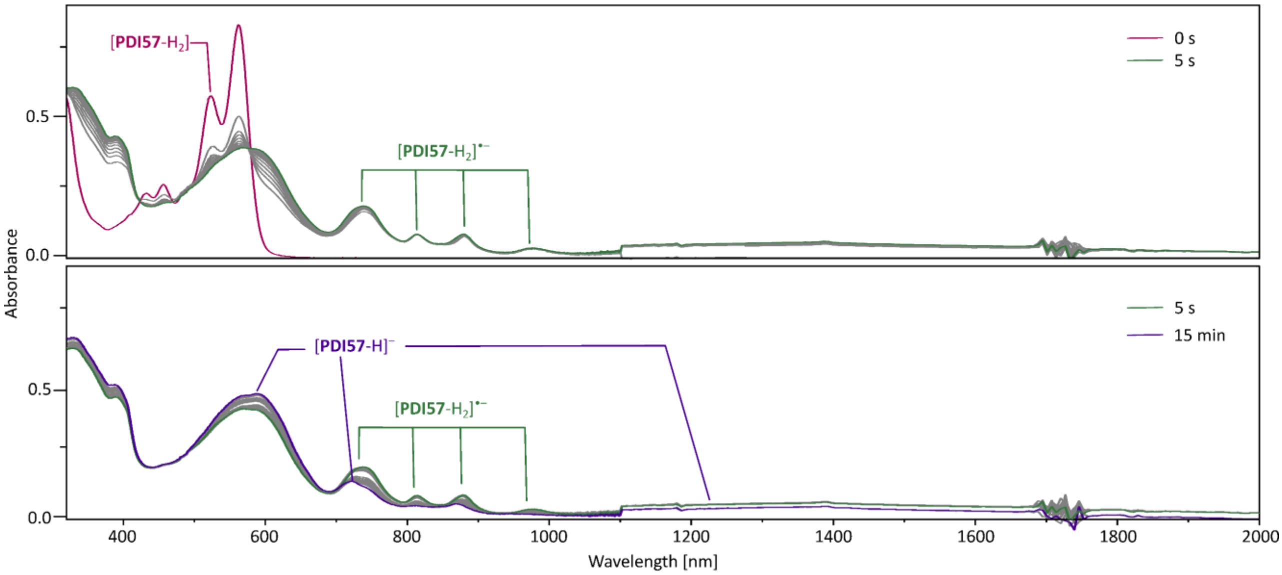

Figure S54. Evolution of absorption spectra after addition of *t*BuOK (1 equiv, THF) to the [**PDI57**-H_2] THF solution (**in the absence of** 18c6). The solution was kept in the dark.

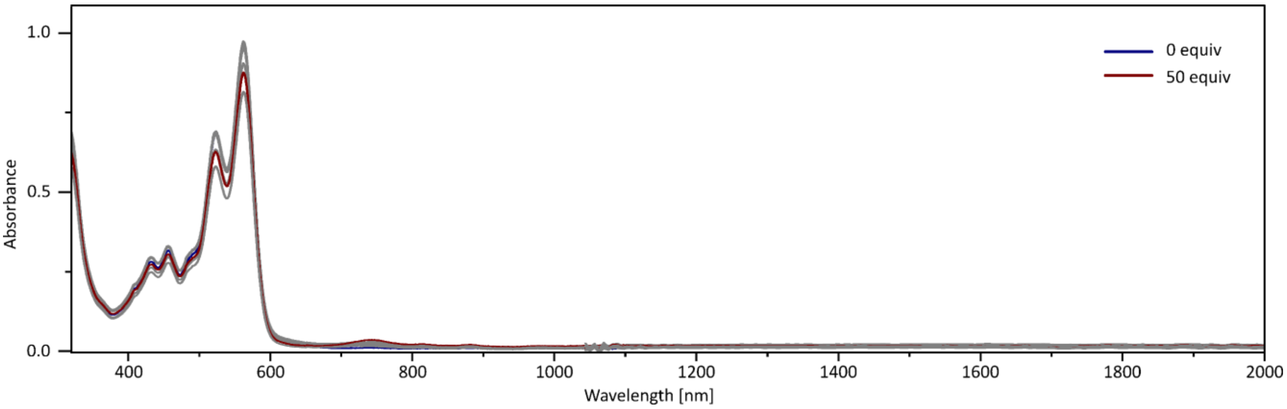

Figure S55. Absorption spectra of [**PDI57**-H_2] titrated with [K(18c6)O*t*Bu] complex (0.05 mM, THF).

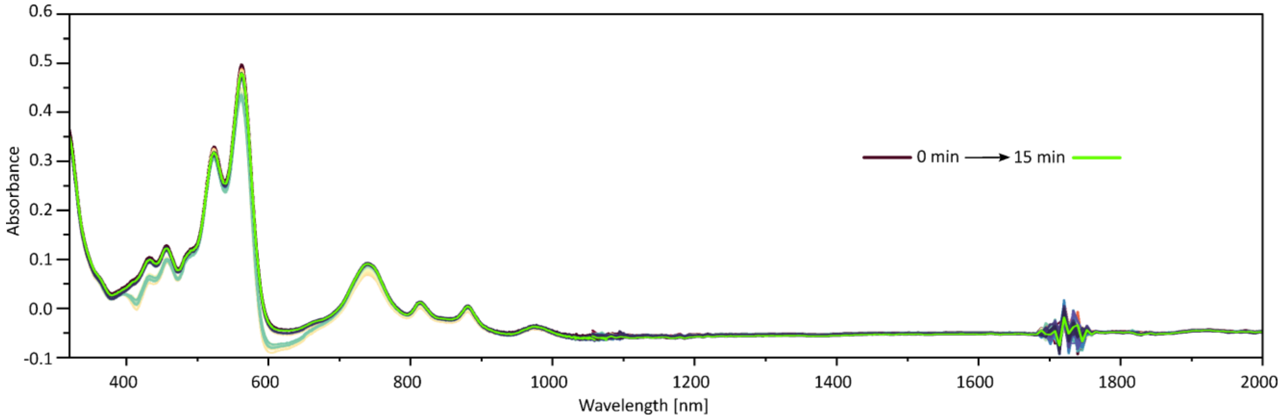

Figure S56. Absorption spectra of [**PDI57**-H_2] treated with 3 equiv of tBuOK and 100 equiv of 18c6.

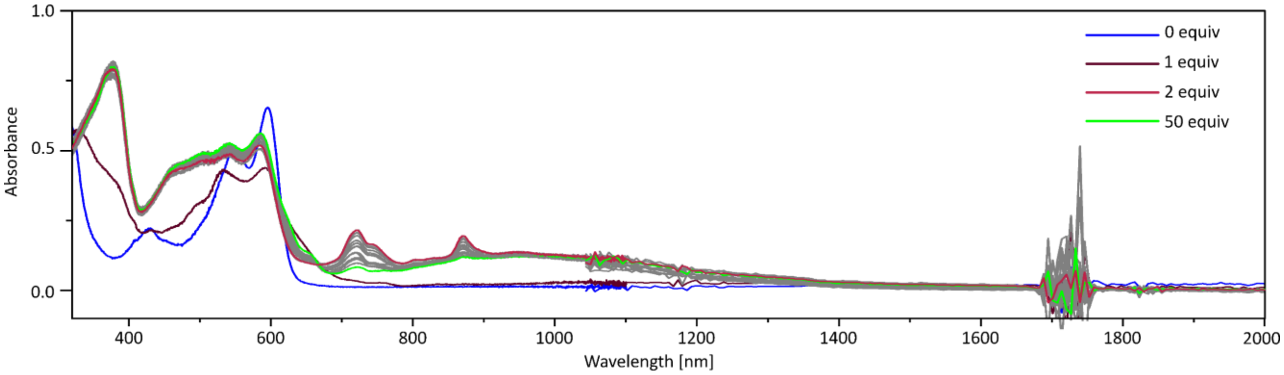

Figure S57. Absorption spectra of [**PDI55**-H_2] titrated with *t*BuOK without addition of 18c6.

Table S2. Absorption maxima (λ_{max}, nm) observed in spectrophotometric titrations of **P*mn***-H_2 systems. If multiple forms are present, corresponding maxima are indicated by underlining and/or *italics*.

**Titration/Observed forms**	[**PTE55**-H_2]	[**PTE55**-H]$^{-}$	[**PTE55**]$^{2-}$	[**PTE55**]$^{\bullet-}$	[**PTE55**]$^{\bullet\bullet}$	[**PTE55**]$^{\bullet 3-}$	
**PTE55**-H_2 + *t*BuOK + I_2[a+c]	312, 499, 528	395, 518, 1082 (up to 1700)	389, 506, 996 (up to 1500)	397, 505, 964, 1250 (up to 1700)	296, 476, 569, 609, 778, 903	not assigned	
**PTE55**-H_2 + *t*BuOK[a']	312, 499, 528	400, 480, 1016 (up to 1500)	386, 484, 998 (up to 1500)	not assigned	not assigned	386, 500, 660, 820, 1002 (up to 1450)	
	[**PTE57**-H_2]	[**PTE57**-H]$^{-}$	[**PTE57**-H]$^{\bullet 2-}$				
[**PTE57**-H_2] + *t*BuOK + 18c6[a']	308, 396, 426, 468, 500	304, 410, 516, 696, 1160 up to 1500	392, 482, 1012 up to 1450	not assigned	not assigned	not assigned	
	[**PTE77**-H_2]	[**PTE77**-H_2]$^{\bullet-}$	[**PTE77**-H_2]$^{2-}$				
[**PTE77**-H_2] + *t*BuOK + 18c6[a']	316, 416, 438, 466	358, 454, 654, 928, 1008 up to 1250	356, 456, 626, 740, 836, 966 up to 1200				
	[**PDI55**-H_2]	[**PDI55**-H]$^{-}$<br>[**PDI55**-H_2]$^{\bullet-}$	[**PDI55**]$^{2-}$<br>[**PDI55**-H_2]$^{2-}$<br>*[**PDI55**-H]$^{\bullet 2-}$*	[**PDI55**]$^{\bullet-}$	[**PDI55**]$^{\bullet\bullet}$	[**PDI55**]$^{\bullet 3-}$<br>[**PDI55**-H]$^{3-}$	[**PDI55**]$^{4-}$
[**PDI55**-H_2] + *t*BuOK + I_2[a+c]	310, 320, 434, 550, 596	340, 552, 582, 728, 756, 1038, broad band up to 2000	380, 512, 546, 590, 962 up to 1500	356, 540, 1208 up to 2000	284, 364, 446, 480, 520, 620, 786, 886, 1038	not assigned	not assigned
[**PDI55**-H_2] + tBuOK + 18c6 + KN[a'+b']	432, 560, 598	596 broad, 772, 850, 922, 1030, broad band up to 2100	464, 552, 588/588, 678, *738, 856,* 1028 up to 2100	not assigned	not assigned	464, 566, 586, 674, 724, 852, 944, broad band up to 1600	600, 676
[**PDI55**-H_2] + NaN + 15c5[b'']	304, 322, 434, 560, 598	326, 486, 596, 726, 760, 838, 1040, broad band up to 2250	390, 556, 596/596, 664, 1046 broad up to 1650	not assigned	not assigned	not assigned	600, 616, 664
[**PDI55**-H_2] + KN + 18c6[b']	296, 324, 414, 560, 598	338, 394, 478, 554, 602, 726, 762, 842, 1046, broad band up to 2100	388, 598/598, 668, *728, 822,* 1056 up to 1450	not assigned	not assigned	598, 628, 706	474, 536, 644, 736
	[**PDI57**-H_2]	[**PDI57**-H]$^{-}$<br>[**PDI57**-H_2]$^{\bullet-}$	[**PDI57**-H]$^{\bullet 2-}$<br>[**PDI57**-H_2]$^{2-}$	[**PDI57**]$^{\bullet}$	[**PDI57**-H]$^{3-}$		
[**PDI57**-H_2] + *t*BuOK + I_2[a+c]	300, 430, 456, 522, 562	320, 394, 592 (broad), 722, 756, 882 (very small), 1284 up to 2000	not assigned	284, 428, 458, 500, 532, 566, 650	not assigned		
[**PDI57**-H_2] + *t*BuOK + 18c6[a']	298, 434, 458, 526, 566	336, 404, 462, 496, 586 (broad), 740, 814, 878, 984, 1420 up to 2000	374, 576, 712, 842, 926	not assigned	356, 628, 646, 688		

[**PDI57**-H_2] + NaN + 15c5[b”]	298, 434, 458, 526, 566	310, 334, 398, 466, 490, 594 (broad), 738, 814, 884, 974, 1420 up to 2000	344, 608, 630, 656	not assigned	not assigned
[**PDI57**-H_2] + KN + 18c6[b’]	298, 434, 458, 526, 566	304, 330, 406, 454, 488, 612 (broad), 746, 820, 886, 970, 1442 broad up to 2250	380, 576, 710, 848, 926		
	[**PDI77**-H_2]	[**PDI77**-H_2]$^{\bullet-}$	[**PDI77**-H_2]$^{2-}$		
[**PDI77**-H_2] + *t*BuOK + 18c6[a’]	290, 312, 422, 448, 492, 528	342, 436, 466, 654, 722, 738, 796, 820, 870, 940	336, 618, 646		

[a] THF, *t*BuOK [a’] THF, *t*BuOK, 18c6 [b] THF, KN [b’] THF, KN, 18c6 [b”] THF, NaN, 15c5 [c] THF, I_2.

3.4. ESR Spectroscopy

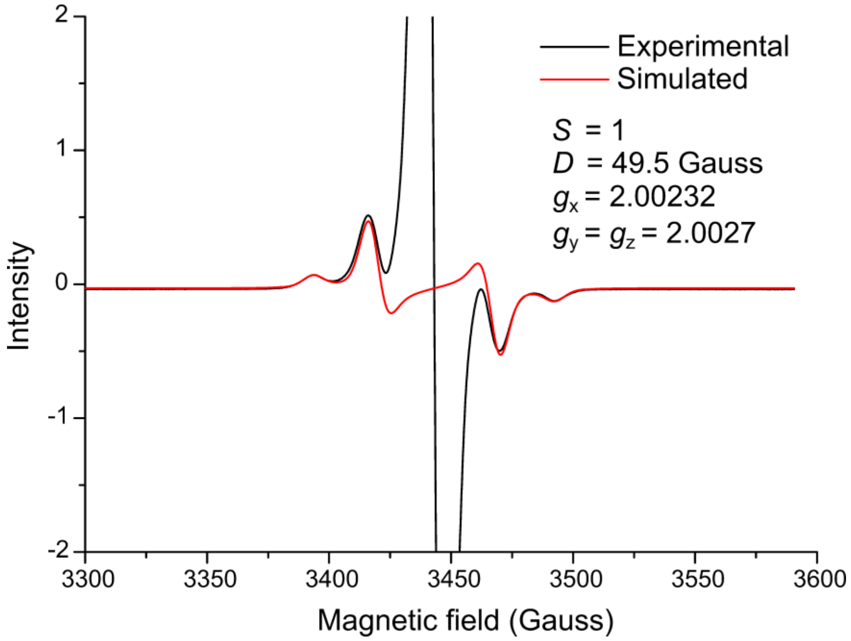

Figure S58. EPR [**PTE55**]••, frozen toluene, 107 K: black, measured; red, simulated.

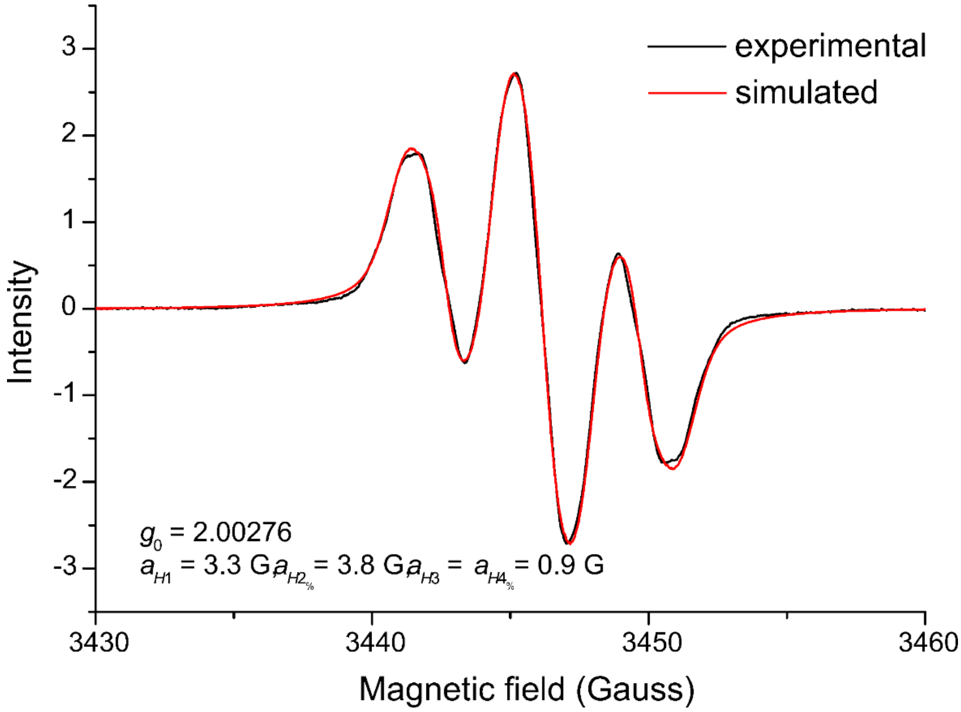

Figure S59. EPR of [**PDI57**-H]•, toluene, 300 K: measured (black trace), simulated (red trace).

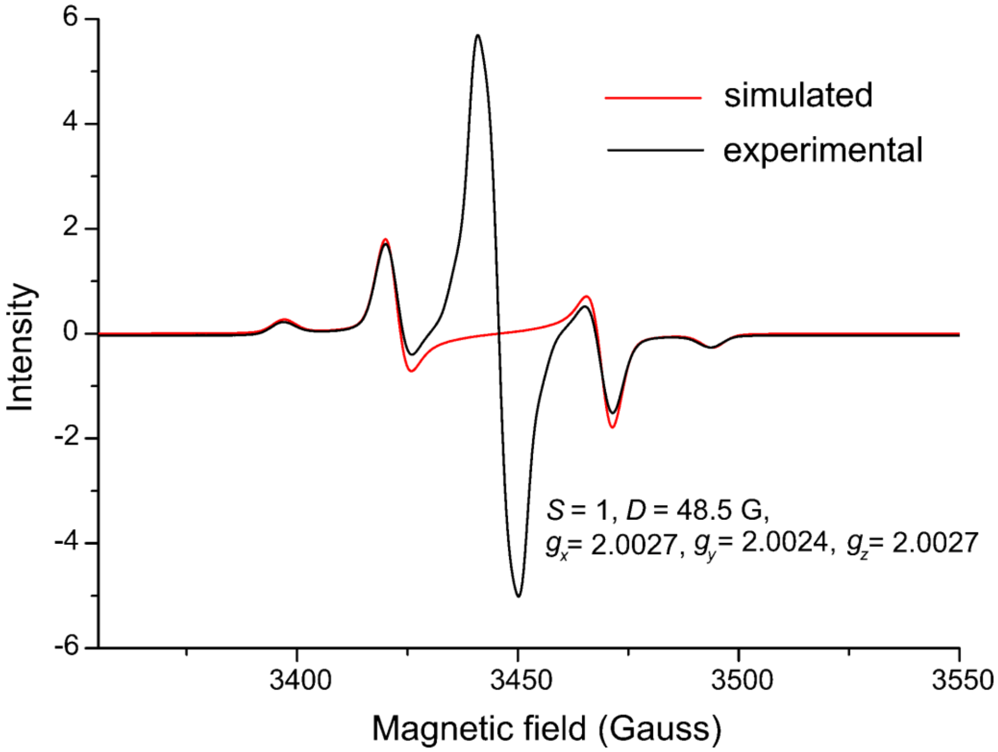

Figure S60. EPR of [**PDI55**]$^{••}$, frozen toluene, 109 K: measured (black trace), simulated (red trace).

3.5. Emission Spectroscopy

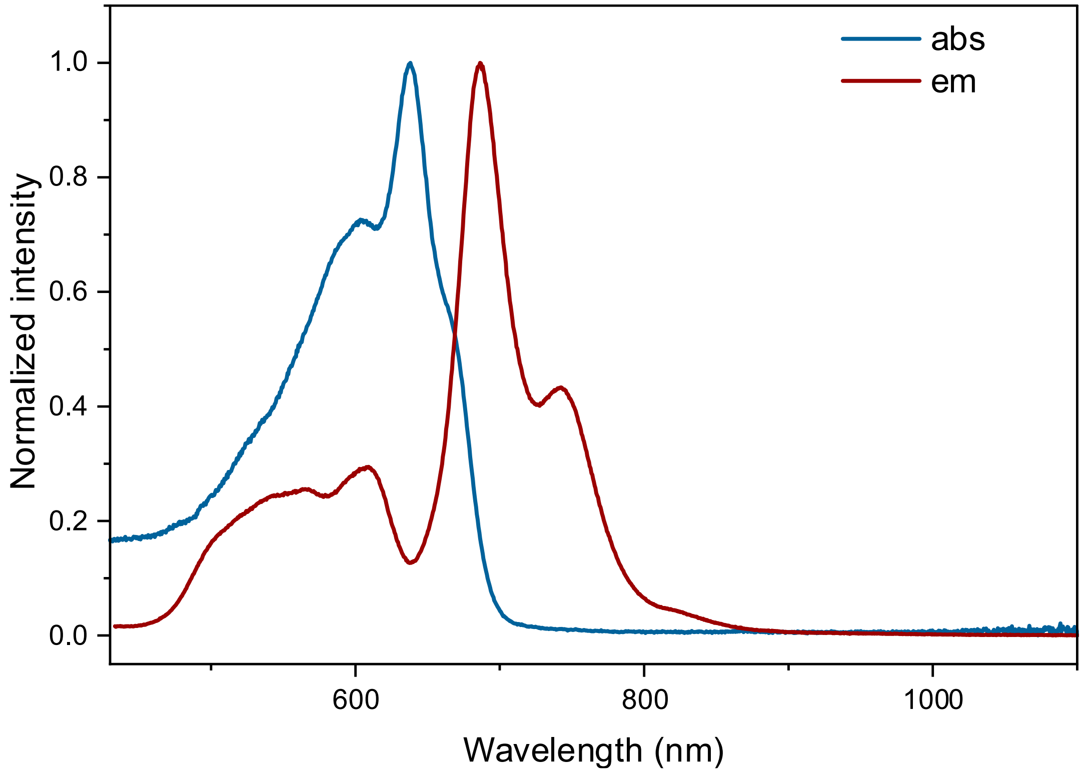

Figure S61. Absorption (blue trace) and emission (red trace) spectra of [**PDI77**]$^{2–}$ obtained by reduction with KN in THF.

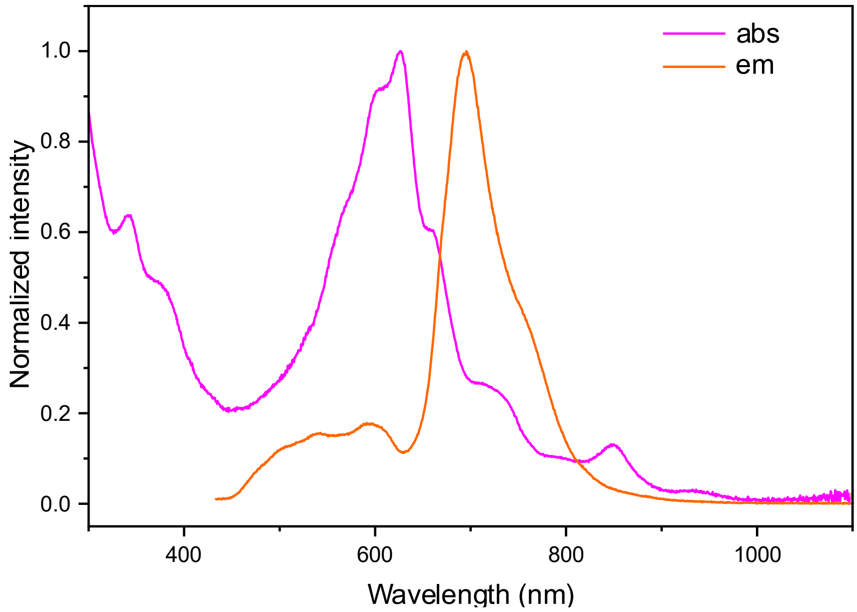

Figure S62. Overlay of absorption (pink) and emission (orange) spectra recorded at an intermediate stage of the reductive titration of **PDI57**-H_2 with KN in THF. At this stage, the solution contains a mixture of the radical anion [**PDI57**-H_2]$^{\bullet-}$ and the dianion [**PDI57**-H_2]$^{2-}$. The emission originates from the emissive dianion, whereas the red-shifted absorption features arise from the coexisting, non-emissive radical anion.

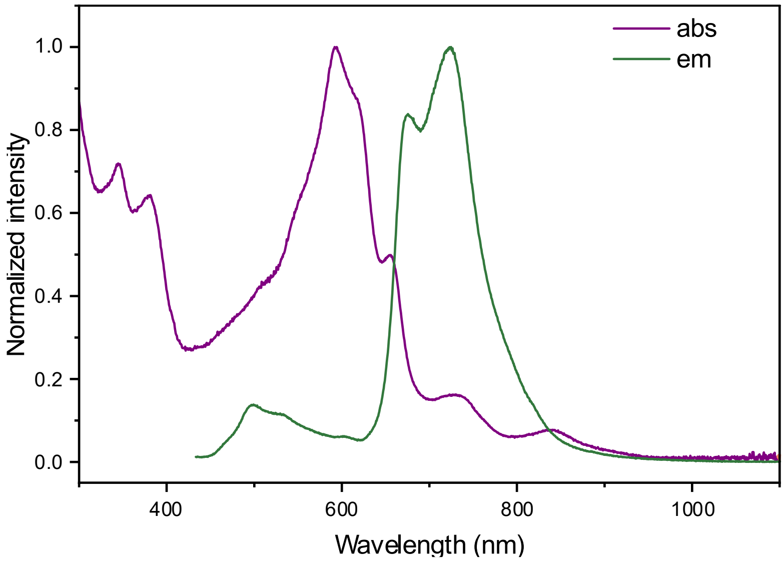

Figure S63. Overlay of absorption (violet) and emission (green) spectra recorded at an intermediate stage of the reductive titration of **PDI55**-H_2 with KN in THF. The absorption spectrum reflects a mixture of the radical anion [**PDI55**-H_2]$^{\bullet-}$ and the dianion [**PDI55**-H_2]$^{2-}$. The observed emission is assigned to the dianion, while the red-shifted absorption bands originate from the non-emissive radical anion present in the mixture.

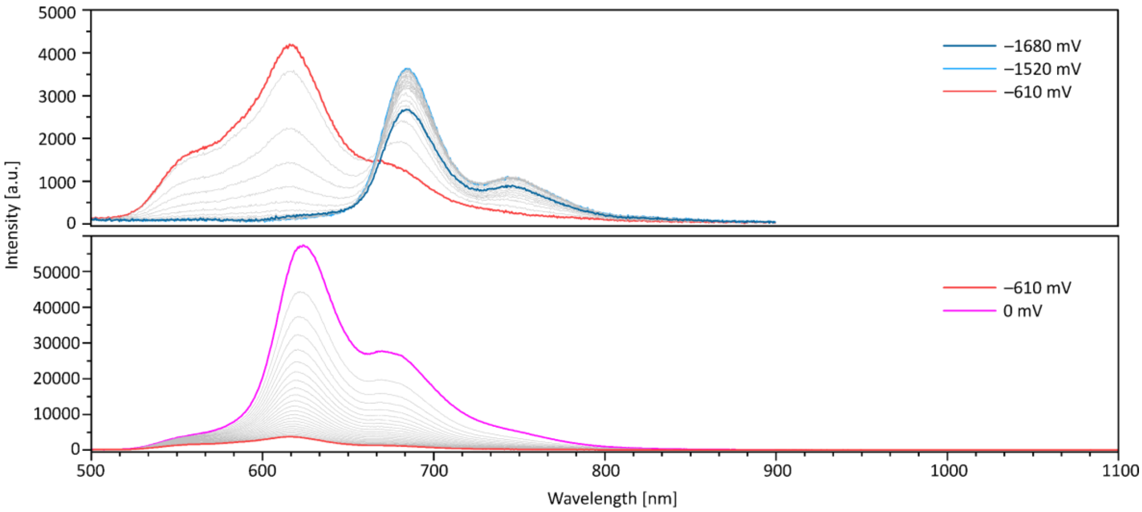

Figure S64. Change of the emission spectra observed during the bulk electrolysis of [**PDI55**-H_2].

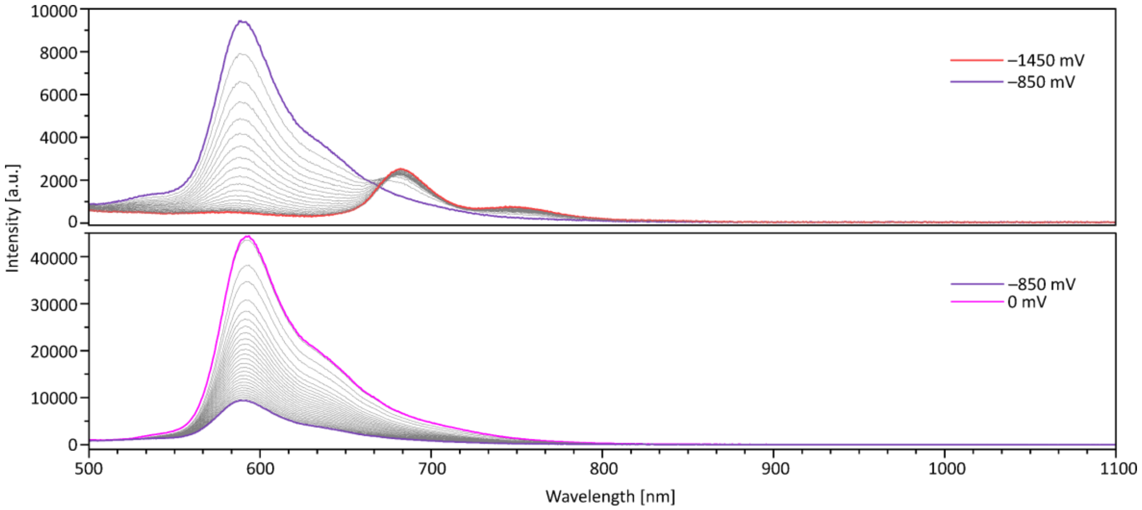

Figure S65. Change of the emission spectra observed during the bulk electrolysis of [**PDI57**-H_2].

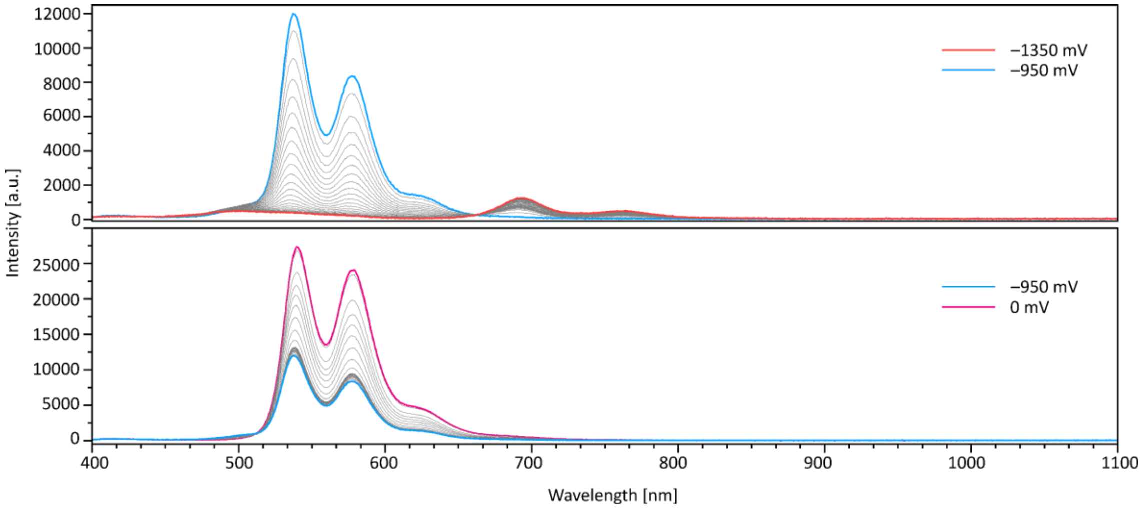

Figure S66. Change of the emission spectra observed during the bulk electrolysis of [**PDI77**-H_2].

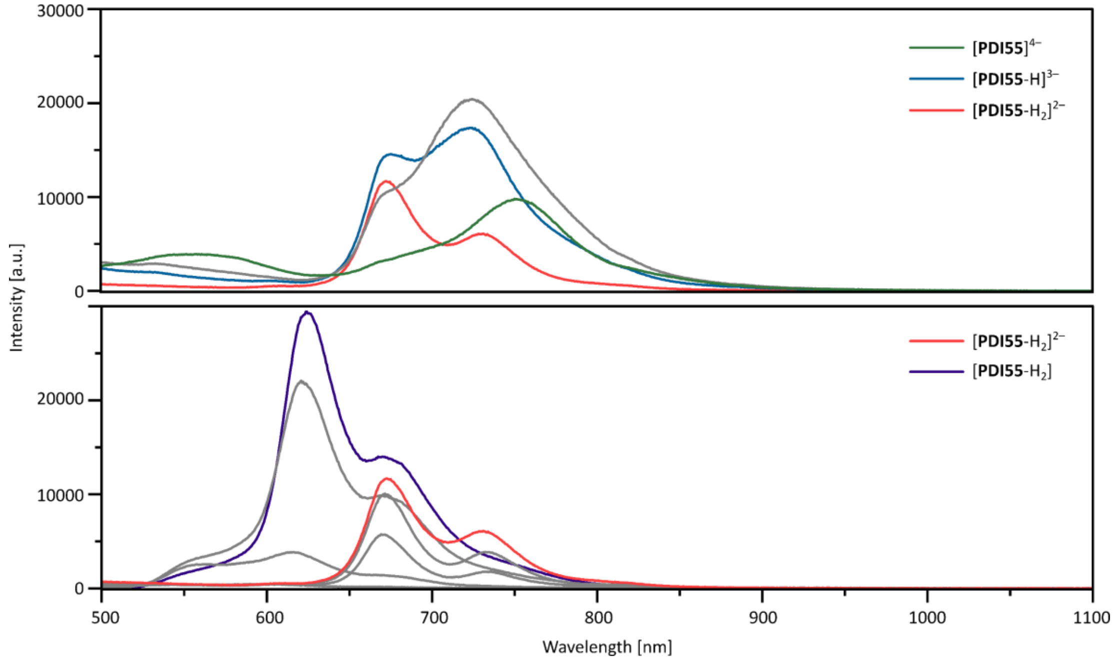

Figure S67. Change of the emission spectra observed during the titration of [**PDI55**-H_2] with KN without 18c6.

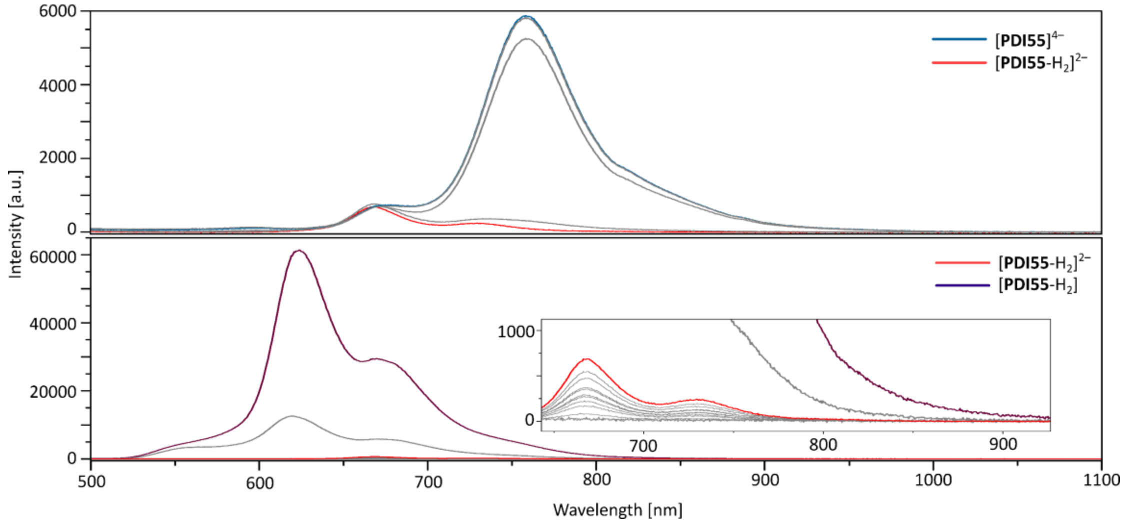

Figure S68. Change of the emission spectra observed during the titration of [**PDI55**-H_2] with *t*BuOK followed by KN without 18c6.

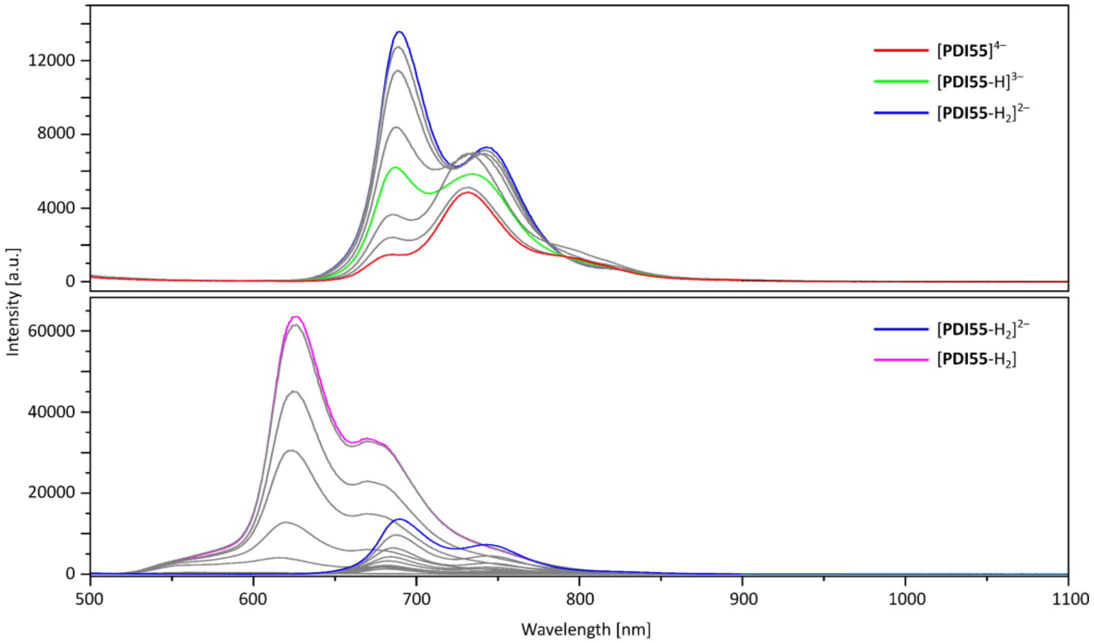

Figure S69. Change of the emission spectra observed during the titration of [**PDI55**-H_2] with *t*BuOK followed by KN in the presence of 18c6.

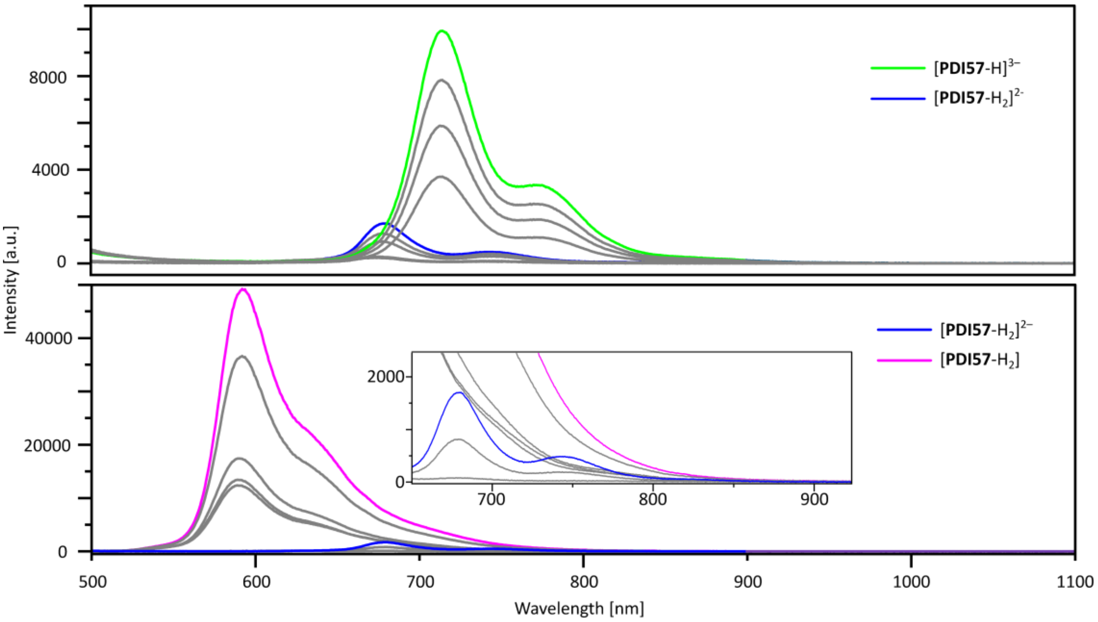

Figure S70. Change of the emission spectra observed during the titration of [**PDI57**-H_2] with *t*BuOK in the presence of 18c6.

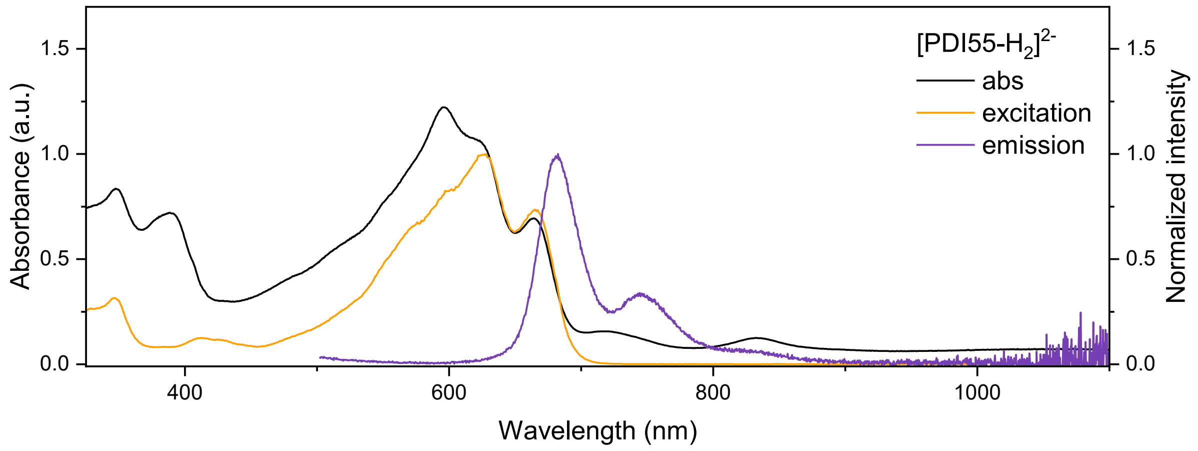

Figure S71. Absorption (black), excitation (orange), and emission (purple) spectra recorded at an intermediate stage of the titration of **PDI55**-H_2 with *t*BuOK in THF in the presence of 18c6. The spectra correspond to a mixed state in which deprotonated and reduced states are both present. The excitation spectrum follows the absorption features of the emissive reduced component indicated in the figure ([**PDI55**-H_2]$^{2-}$), confirming that the observed luminescence originates from this species, whereas the remaining absorption bands arise from coexisting non-emissive components present in the reaction mixture.

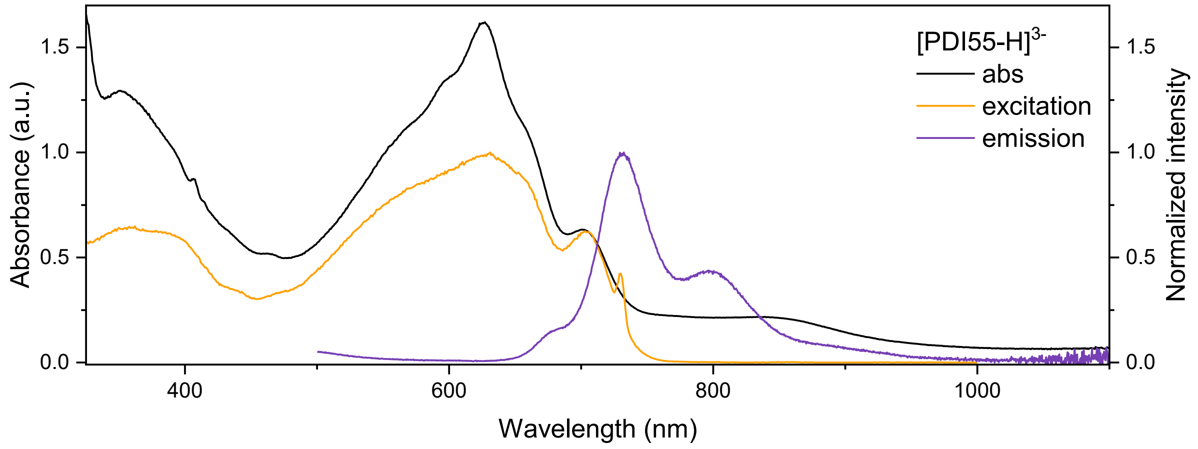

Figure S72. Absorption (black), excitation (orange), and emission (purple) spectra recorded at an intermediate stage of the reductive titration of **PDI55**-H_2 with KN in THF after prior treatment with *t*BuOK in the presence of 18c6. The solution contains a mixture of reduced and deprotonated species. The excitation spectrum matches the absorption of the emissive species [**PDI55**-H]$^{3-}$, demonstrating that the emission originates from this component, while the remaining absorption features are due to coexisting non-emissive species in the mixture and excessive KN (broad peak 750 – 900 nm).

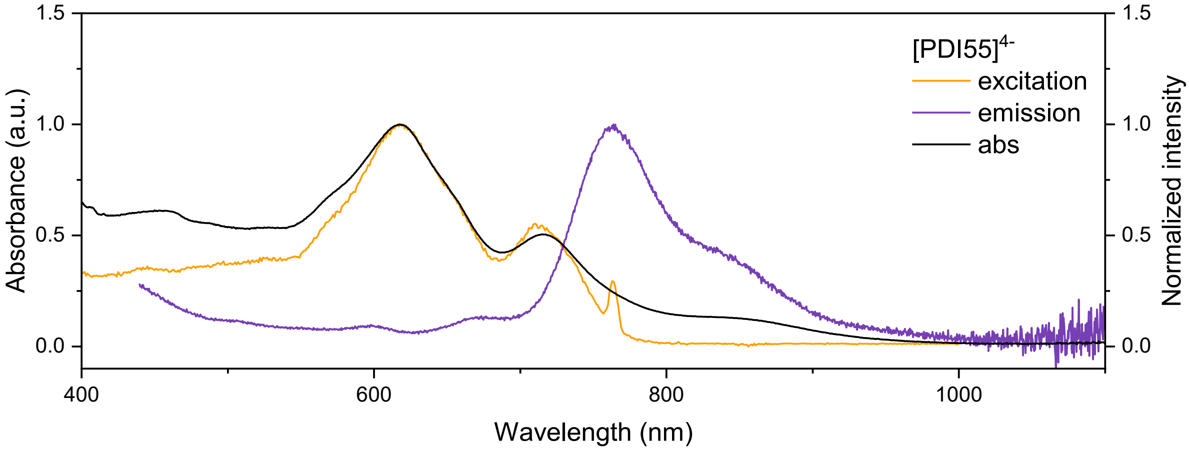

Figure S73. Absorption (black), excitation (orange), and emission (purple) spectra recorded at final stage of the titration of **PDI55**-H_2 with KN in THF after prior treatment with *t*BuOK in the presence of 18c6. The excitation spectrum matches the absorption of the emissive species [**PDI55**-H]$^{4-}$, demonstrating that the emission originates from this component, while the remaining absorption features are due excess of KN (750 – 900 nm).

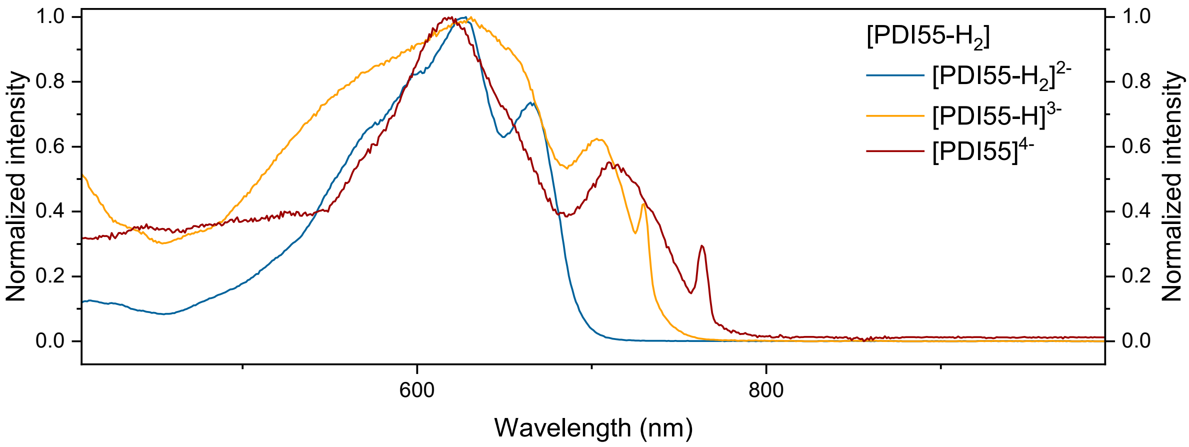

Figure S74. Comparison of excitation spectra of emissive forms obtained during the titration of [**PDI55**-H_2] with tBuOK followed by titration with KN in the presence of 18c6.

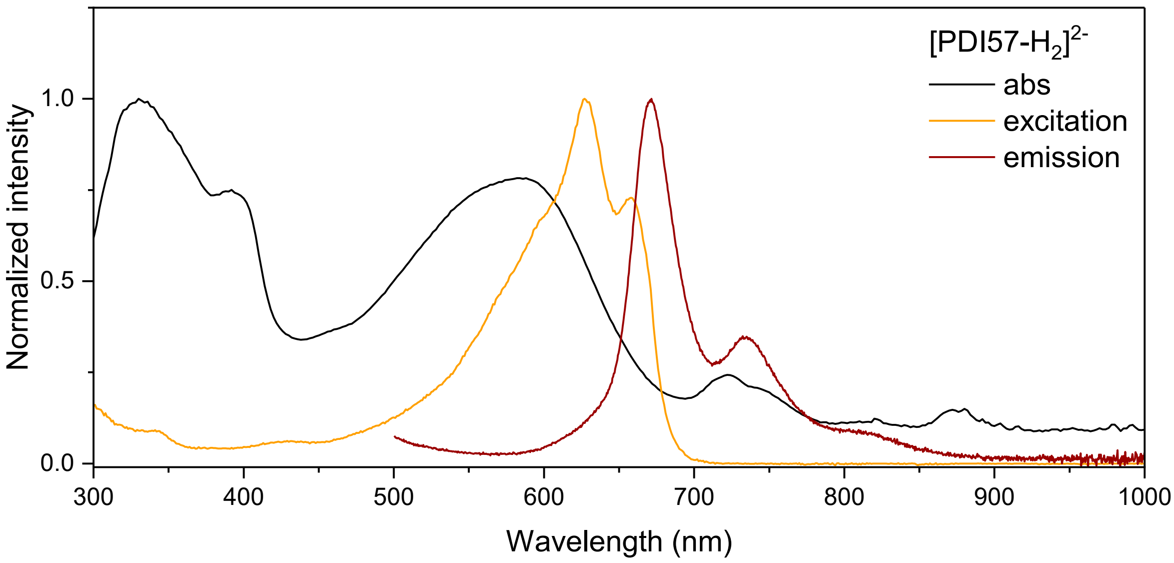

Figure S75. Absorption (black), excitation (orange), and emission (red) spectra recorded at an intermediate stage of the titration of **PDI57**-H_2 with *t*BuOK in THF in the presence of 18c6. The spectra correspond to a mixed state in which reduction and deprotonation are both observed. The excitation spectrum follows the absorption

features of the emissive reduced species indicated in the figure ([**PDI57**-H_2]$^{2-}$), confirming that the observed luminescence originates from this component, whereas the remaining absorption bands arise from coexisting non-emissive [**PDI57**]$^{2-}$ and [**PDI57**-H_2]$^{\bullet-}$present in the reaction mixture.

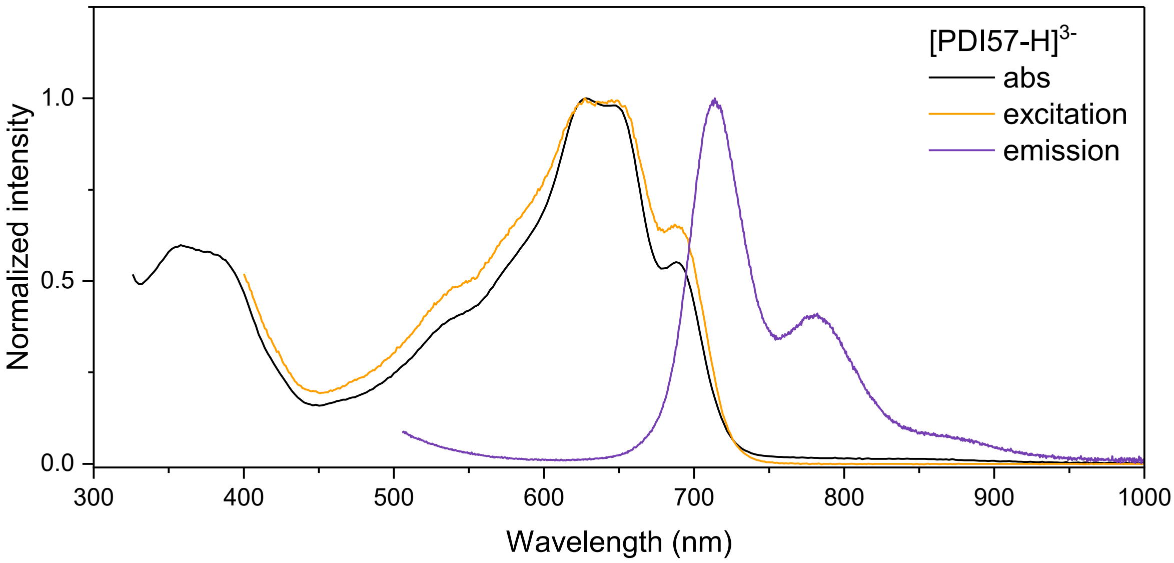

Figure S76. Absorption (black), excitation (orange), and emission (purple) spectra recorded at final stage of the titration of **PDI57**-H_2 with KN in THF after prior treatment with *t*BuOK in the presence of 18c6. The excitation spectrum matches the absorption of the emissive species [**PDI57**-H]$^{3-}$.

3.6. Electrochemistry

Each **PTE*mn***-H_2 and **PDI*mn***-H_2 exhibits two quasi-reversible one-electron reductions and an irreversible one-electron oxidation (Figures S77–S86; Table S3). For the diimides, the first and second reductions occur at $E_{1/2} \approx$ –1.11 to –1.18 V and –1.45 to –1.51 V, respectively. For the tetraesters, both steps are shifted cathodically by ca. 0.5–0.6 V (E1/2 ≈ –1.65 to –1.77 V and –1.97 to –2.17 V). The close similarity of these potentials to those of the parent PDI and PTE derivatives, together with a small additional cathodic shift of ca. 0.1 V, supports a largely core-centered redox assignment, with slight perturbation by the fused subunits. These measurements, therefore, provide a baseline for the redox coordinate at fixed protonation within the formal Pourbaix space.

Spectroelectrochemical measurements were used to correlate the voltammetric waves with the optical signatures of the reduced states and to assess whether cathodic bias induces additional chemistry. For the **PDI*mn***-H_2 series, spectroelectrochemistry cleanly generated the corresponding [**PDI*mn***-H_2]$^{\bullet-}$ and [**PDI*mn***-H_2]$^{2-}$ anions at potentials approximately matching the first and second reductions in CV/DPV (Figures S39, S45, S49). In all cases, the resulting absorption spectra closely resemble those reported for fusion-free PDI radical anions and dianions,[10–14] consistent with PDI-centered reduction and minimal chemical involvement of the annulated five- and seven-membered rings under electrochemical conditions.

For the tetraesters, spectroelectrochemistry showed a broader range of behavior. **PTE77**-H_2 underwent an overall two-step reduction to [**PTE77**-H_2]$^{\bullet-}$ and [**PTE77**-H_2]$^{2-}$ with spectra consistent with PTE-centered reduced states (Figure S50), as expected for a PTE-like system.[15] In contrast, for **PTE57**-H_2 and **PTE55**-H_2, cathodic bias produced a more complex outcome (Figures S51, S52). At potentials more negative than –0.9 V vs Ag/Ag$^+$ (ca. –1.1 V vs Fc/Fc$^+$), broad NIR absorptions attributable to deprotonated anions were observed, assigned to [**PTE57**-H]$^-$ and [**PTE55**-H]$^-$/[**PTE55**]$^{2-}$, respectively. At potentials corresponding to the first one-electron reduction of the PTE core, these deprotonated species were progressively supplanted by the radical anions [**PTE57**-H_2]$^{\bullet-}$ and [**PTE55**-H_2]$^{\bullet-}$, and at still more negative potentials, they were ultimately reduced to the corresponding dianions.

The initial deprotonation observed for **PTE57**-H_2 and **PTE55**-H_2 is tentatively attributed to electrogenerated base chemistry. In aprotic media, trace dioxygen can be reduced to superoxide $[O_2]^{\bullet-}$,[16] which can act as an electrogenerated base (EGB)[17] toward sufficiently acidic substrates. This pathway is expected to become relevant to the PTE series because their first reductions require more cathodic potentials, at which EGB formation becomes more efficient. In line with this rationale, no analogous deprotonation is observed for the **PDI*mn***-H_2 series, which is reduced at less negative potentials than those at which significant EGB formation is expected. The relative separation of oxidation and reduction peaks observed in CV data for the tetraesters may potentially be linked to proton transfer processes discussed herein.

Bulk electrolysis of the **PDI*mn***-H_2 series was additionally monitored by photoluminescence (Figures S64–S66) to provide an independent diagnostic for reduced-state formation and reversibility. The fluorescence of the neutral states was quenched upon generation of the non-emissive radical anions. The dianions displayed weak, red-shifted, vibronically structured emission, which fully reverted to the original emission profile upon reoxidation.[14,18] The near-identical dianion emission profiles observed for **PDI55**-H_2, **PDI57**-H_2, and **PDI77**-H_2 are consistent with minimal involvement of the annulated periphery in the emissive state.

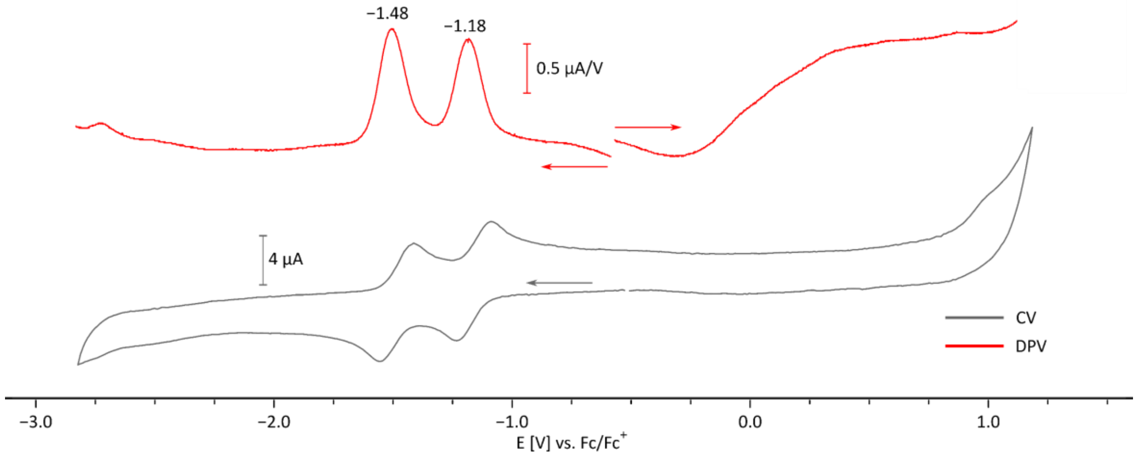

Figure S77. Cyclic (gray trace) and pulse (red trace) voltammograms of [**PDI77**-H_2] measured in THF solution with $TBAPF_6$ electrolyte. Potentials measured vs. Fc/Fc^+. Scan rate: 100 mV/s.

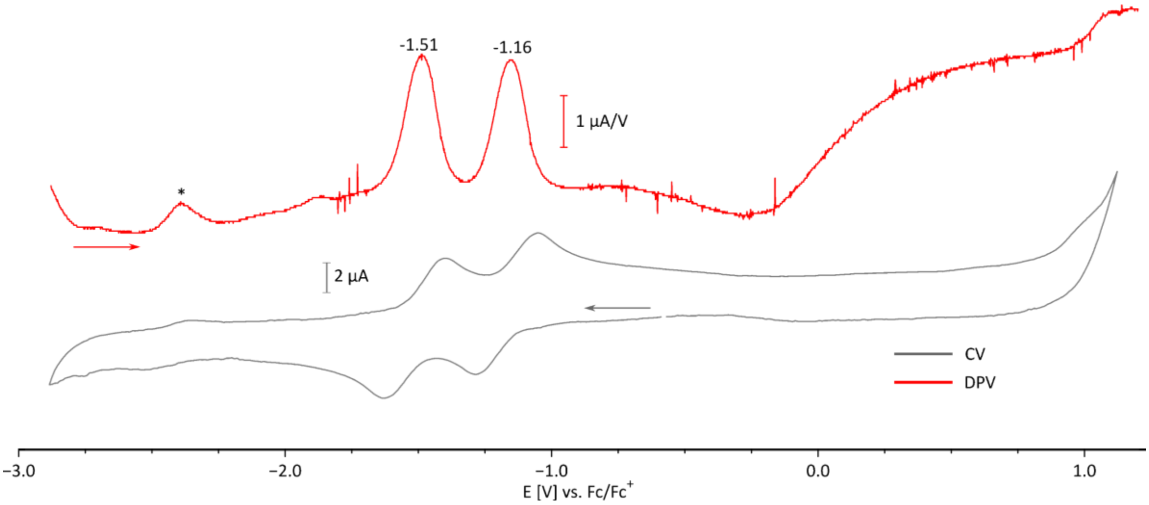

Figure S78. Cyclic (gray trace) and pulse (red trace) voltammograms of [**PDI57**-H_2] measured in THF solution with $TBAPF_6$ electrolyte. Potentials measured vs. Fc/Fc^+. An unidentified impurity is marked with * Scan rate: 100 mV/s.

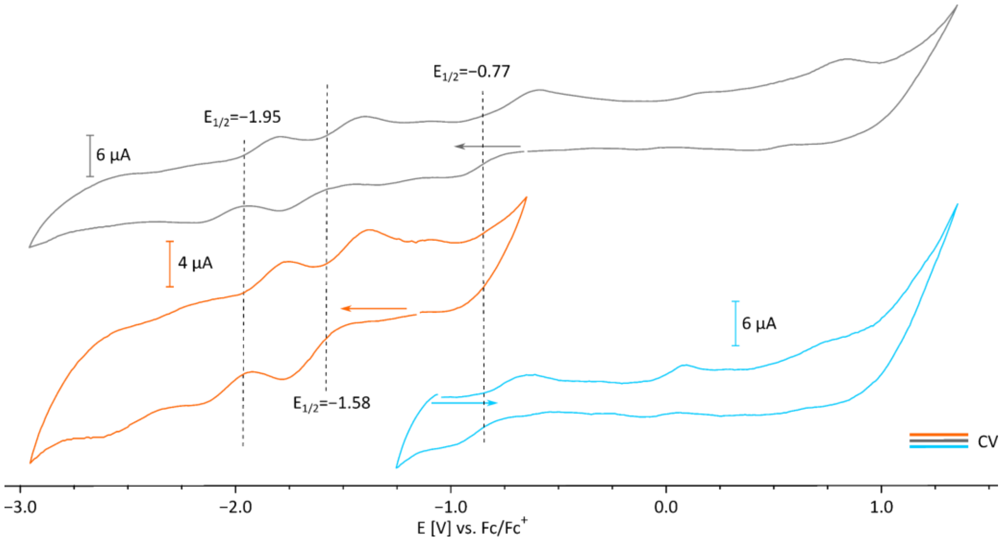

Figure S79. Cyclic (gray trace) voltammogram of [**PDI57**-H]$^-$ measured in THF solution with TBAPF$_6$ electrolyte. Potentials measured vs. Fc/Fc$^+$. Residual **PDI57**-H$_2$ is also observable. Scan rate: 100 mV/s.

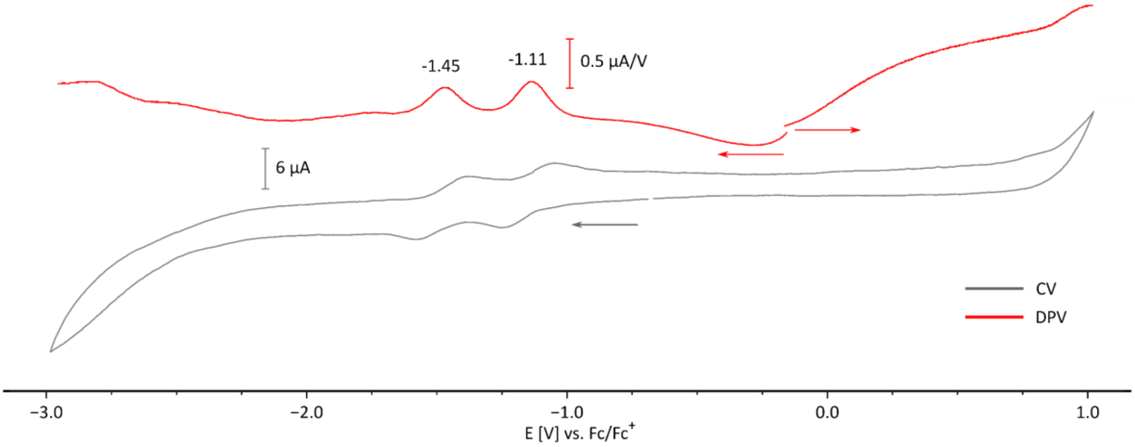

Figure S80. Cyclic (gray trace) and pulse (red trace) voltammograms of [**PDI55**-H$_2$] measured in THF solution with TBAPF$_6$ electrolyte. Potentials measured vs. Fc/Fc$^+$. The cathodic DPV signal is plotted as −Δi. Scan rate: 100 mV/s.

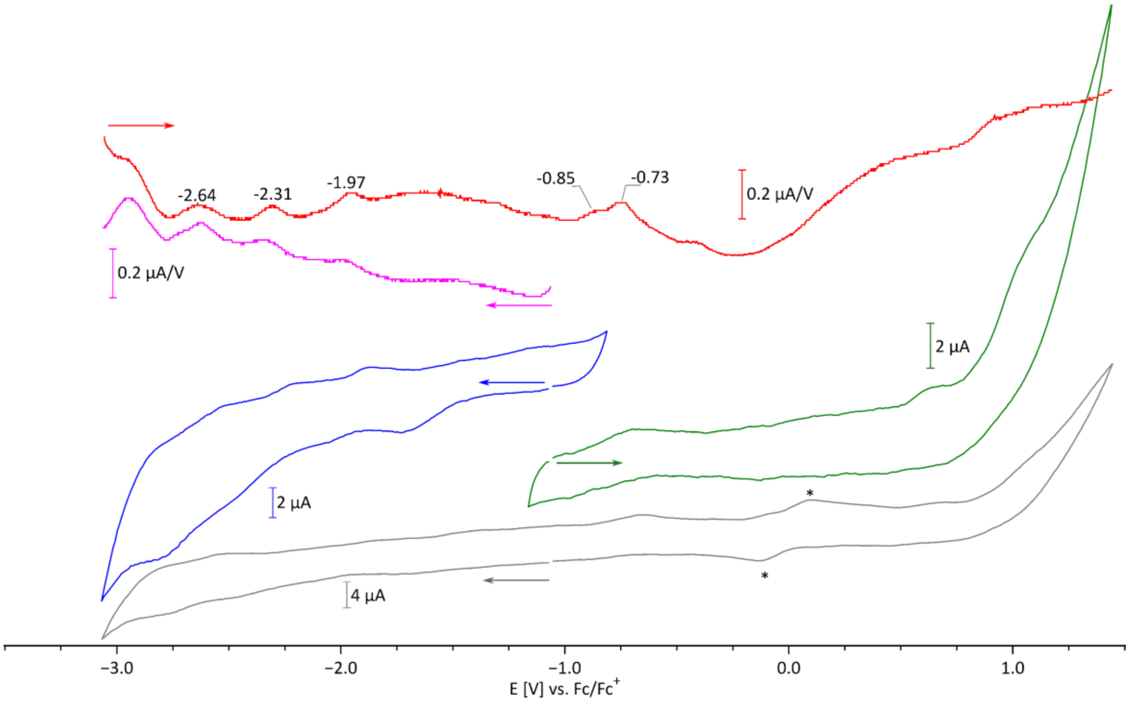

Figure S81. Cyclic (gray, blue, and green traces) and pulse (red trace) voltammograms of [**PDI55**]$^{2-}$ measured in THF solution with $TBAPF_6$ electrolyte. *Fc/Fc$^+$. Potentials measured vs. Fc/Fc$^+$. The cathodic DPV signal is plotted as −Δi. Scan rate: 100 mV/s.

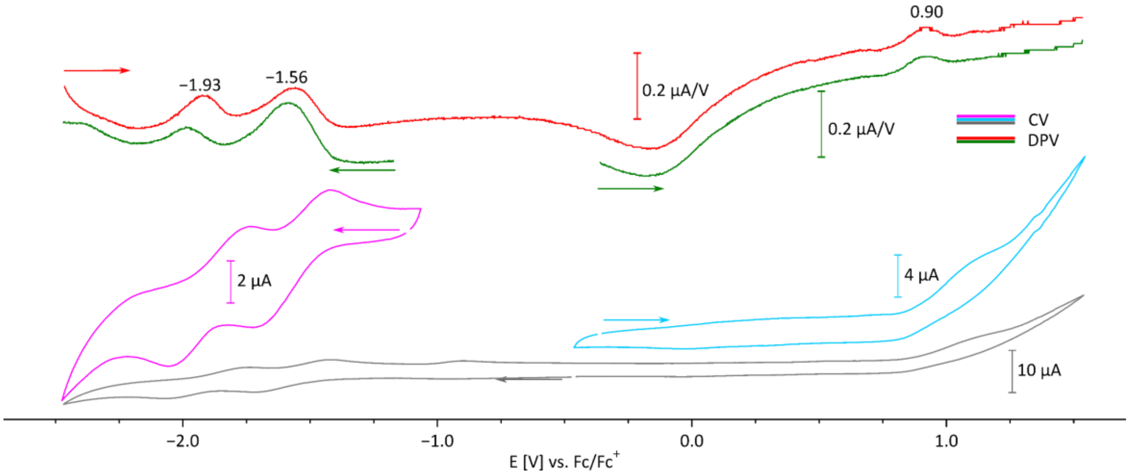

Figure S82. Cyclic (gray trace) and pulse (red trace) voltammograms of [**PTE55**-H_2] measured in THF solution with $TBAPF_6$ electrolyte. Potentials measured vs. Fc/Fc$^+$. The cathodic DPV signal is plotted as −Δi. Scan rate: 100 mV/s.

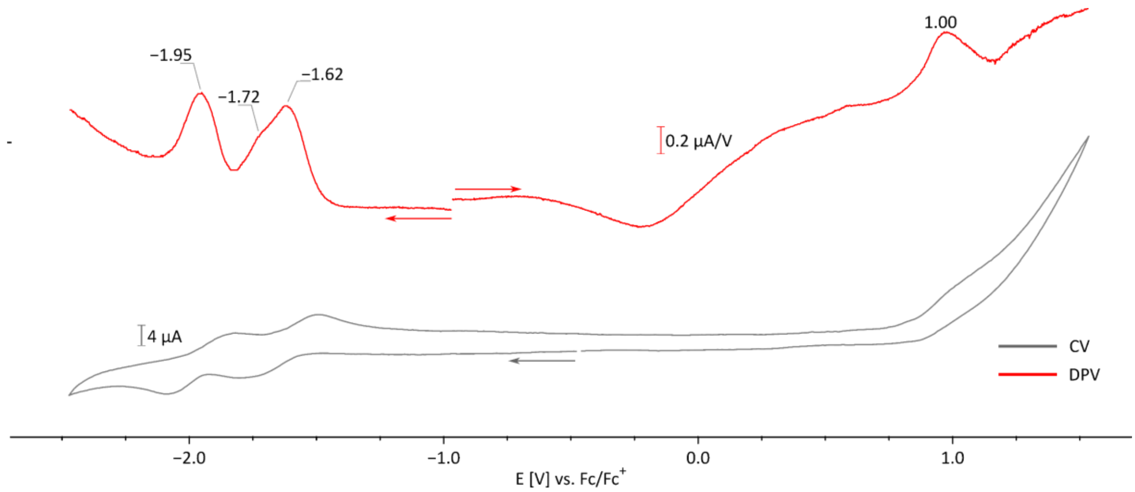

Figure S83. Cyclic (gray trace) and pulse (red trace) voltammograms of [**PTE57**-H_2] measured in THF solution with $TBAPF_6$ electrolyte. Potentials measured vs. Fc/Fc^+. The cathodic DPV signal is plotted as −Δi. Scan rate: 100 mV/s.

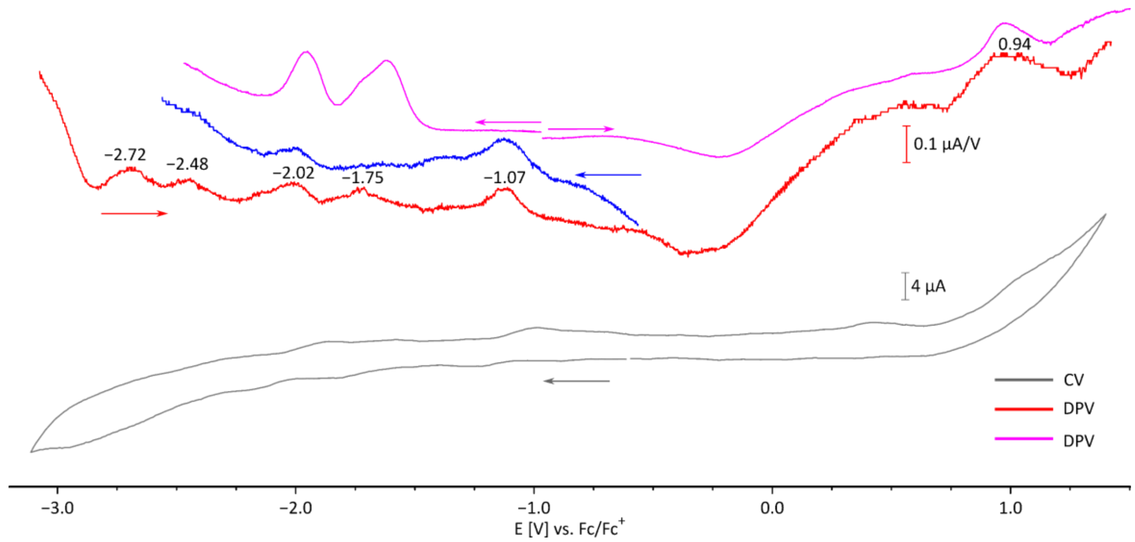

Figure S84. Cyclic (gray trace) and pulse (red trace) voltammograms of [**PTE57**-H]$^-$ measured in THF solution with $TBAPF_6$ electrolyte. Potentials measured vs. Fc/Fc^+. The cathodic DPV signal is plotted as −Δi. Scan rate: 100 mV/s. Pink trace shows [**PTE57**-H_2] pulse voltammogram for comparison.

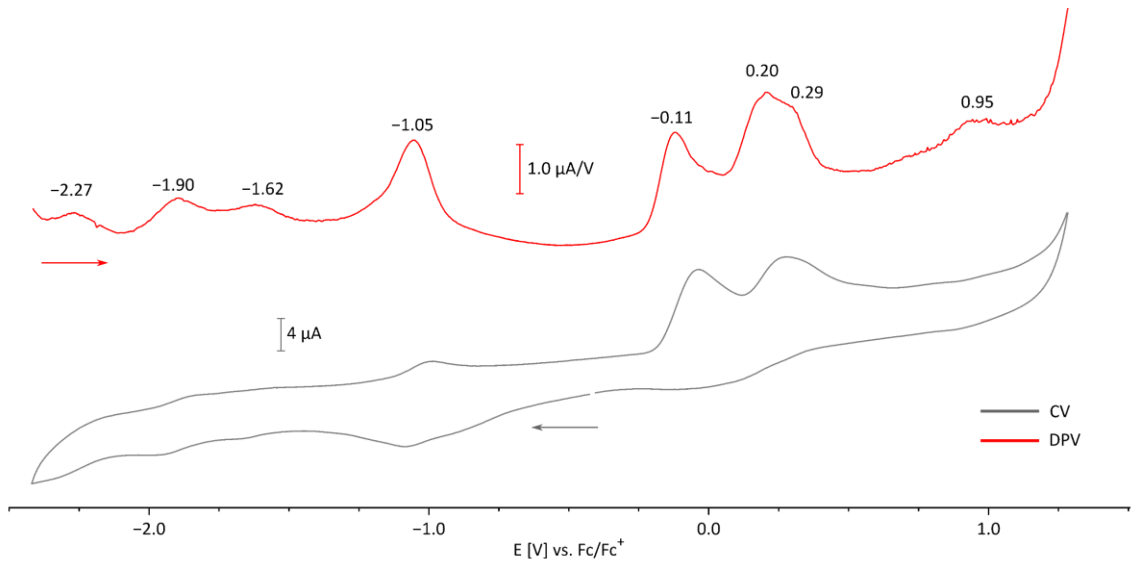

Figure S85. Cyclic (gray trace) and pulse (red trace) voltammograms of [**PTE55**]$^{\bullet\bullet}$ measured in DCM solution with $TBAPF_6$ electrolyte. Potentials measured vs. Fc/Fc^+.

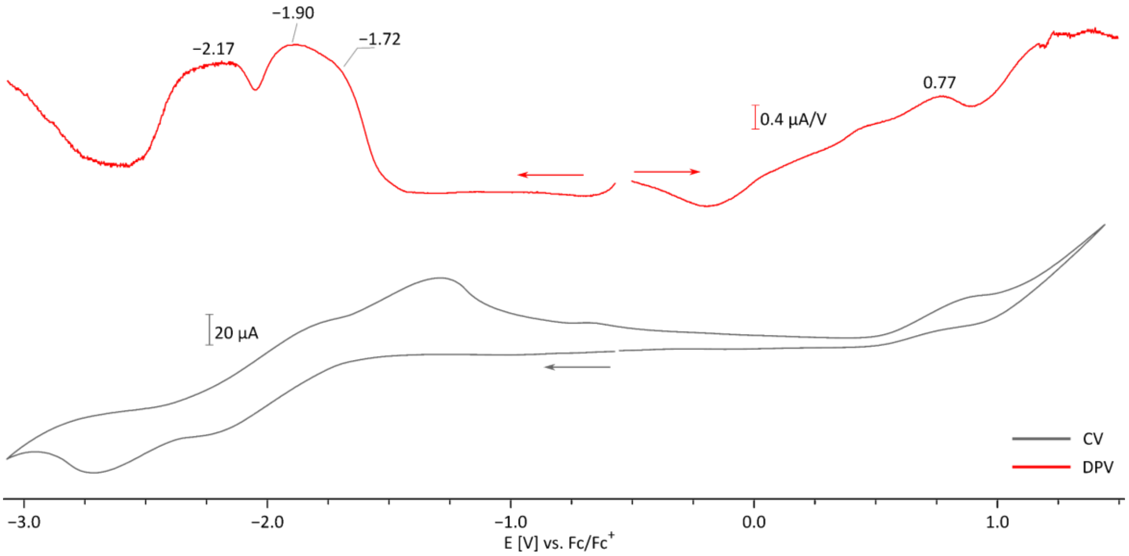

Figure S86. Cyclic (gray trace) and pulse (red trace) voltammograms of [**PTE77**-H_2] measured in THF solution with $TBAPF_6$ electrolyte. Potentials measured vs. Fc/Fc^+. The cathodic DPV signal is plotted as $-\Delta i$. Scan rate: 100 mV/s.

Table S3. Redox properties of the **P*mn***-H_2 and their anions. Half-wave potentials determined by DPV measurements in THF (0.1 M $TBAPF_6$) vs. Fc/Fc^+ at room temperature (20 °C).

	$E_{1/2}^{Red2}$ [V]	$E_{1/2}^{Red1}$ [V]	$E_{1/2}^{Ox1}$ [V]	$E_{1/2}^{Ox2}$ [V]
[**PDI55**-H_2]	~–1.45	–1.11	–[a]	–[a]
[**PDI57**-H_2]	~–1.51	–1.16	–[a]	–[a]
[**PDI77**-H_2]	~–1.48	–1.18	–[a]	–[a]
[**PTE55**-H_2]	~–1.93	–1.56	0.90	–[a]
[**PTE57**-H_2]	~–1.95	(–1.6)–(–1.72)	(~1.00)	–[a]
[**PTE77**-H_2]	~–2.17	(–1.7)–(–1.9)	(~0.77)	–[a]
^{1}[**PDI57**-H]$^-$[c]	~–1.95	–1.58	–0.77	(~0.84)
^{1}[**PTE57**-H]$^-$	–	–	–1.07	(~0.94)
^{1}[**PDI55**]$^{2-}$	~–2.31	–1.97	~–0.85	~0.73[b]
^{1}[**PTE55**]$^{2-}$				

[a] Not observed. [b] overlap. [c] $E_{1/2}$ from CV

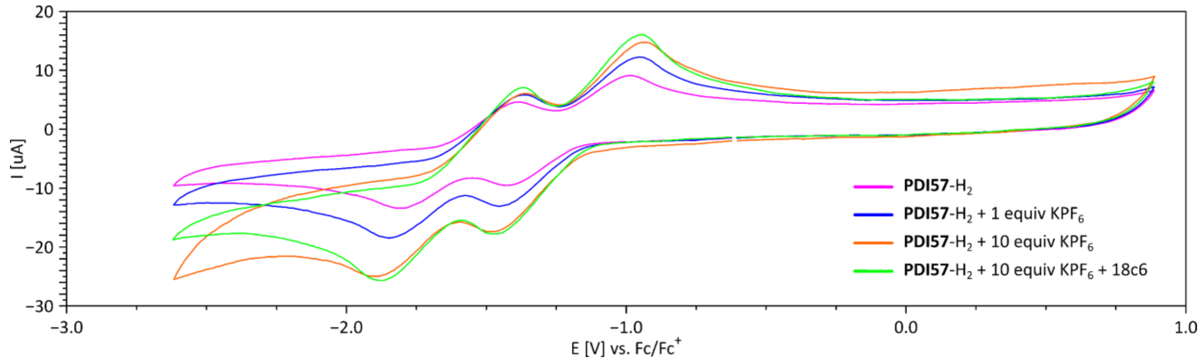

Figure S87. Cyclic voltammograms of [**PDI57**-H_2] measured in THF solution (0.05 mM) with $TBAPF_6$ electrolyte. Potentials measured vs. Fc/Fc^+.

Table S4. Changes of $E_{1/2}$ reduction potentials during cycling voltammetry (THF, $TBAPF_S$), KPF_6 and 18c6 as additives.

	**PDI57**-H_2	**+ 1 equiv KPF_6**	**+ 10 equiv KPF_6**	**+ 10 equiv KPF_6 + 18c6**
**$E_{1/2}$ red1** [V][a]	−1.213	−1.202	−1.197	−1.212
**$E_{1/2}$ red2** [V][a]	−1.595	−1.605	−1.627	−1.625

[a] Potentials vs. Fc/Fc^+.

3.7. Mass spectrometry

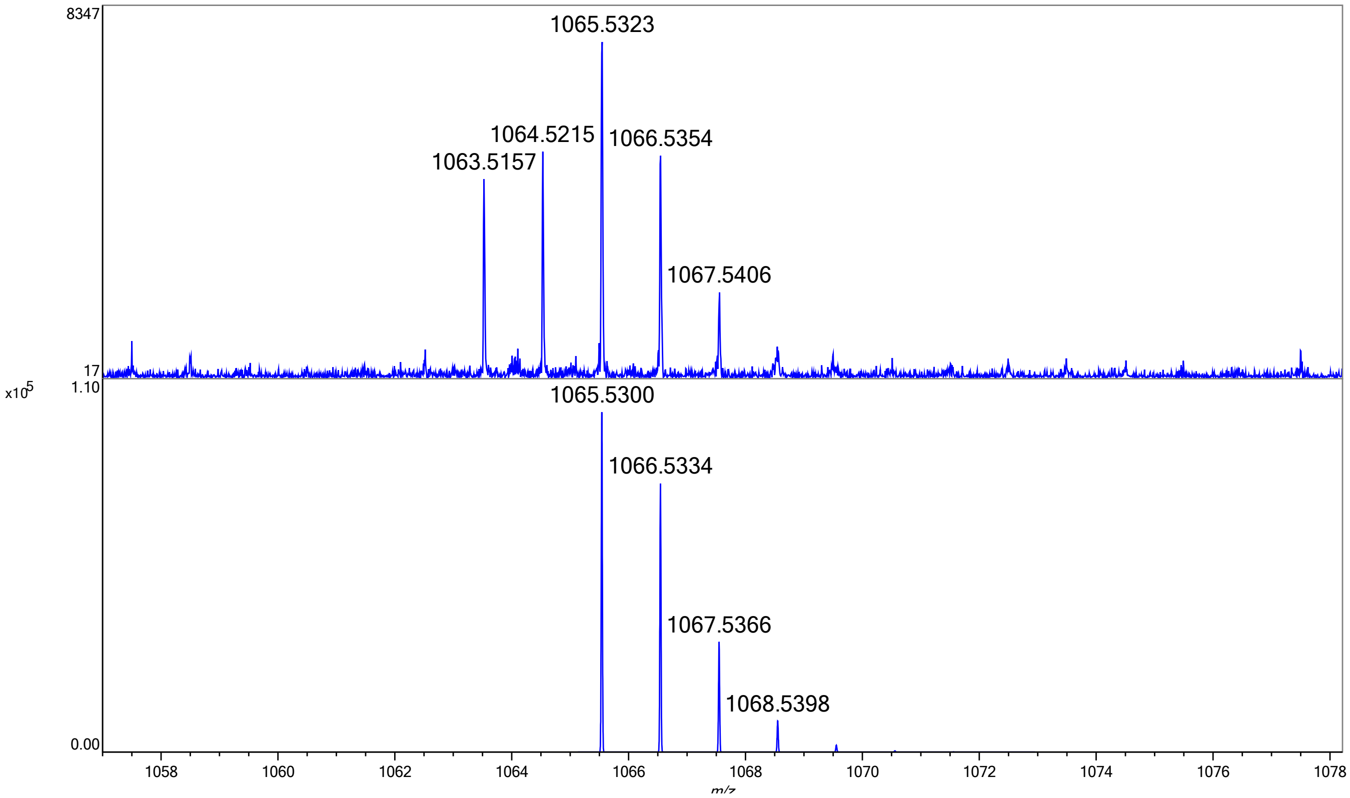

Figure S88. MALDI-MS spectrum of [**PTE57**-DH]. Additional signals in the experimental spectrum come from [**PTE57**-H_2] and [**PTE57**-H]$^-$. Top: experimental, bottom: simulated for $C_{72}H_{71}DO_8$.

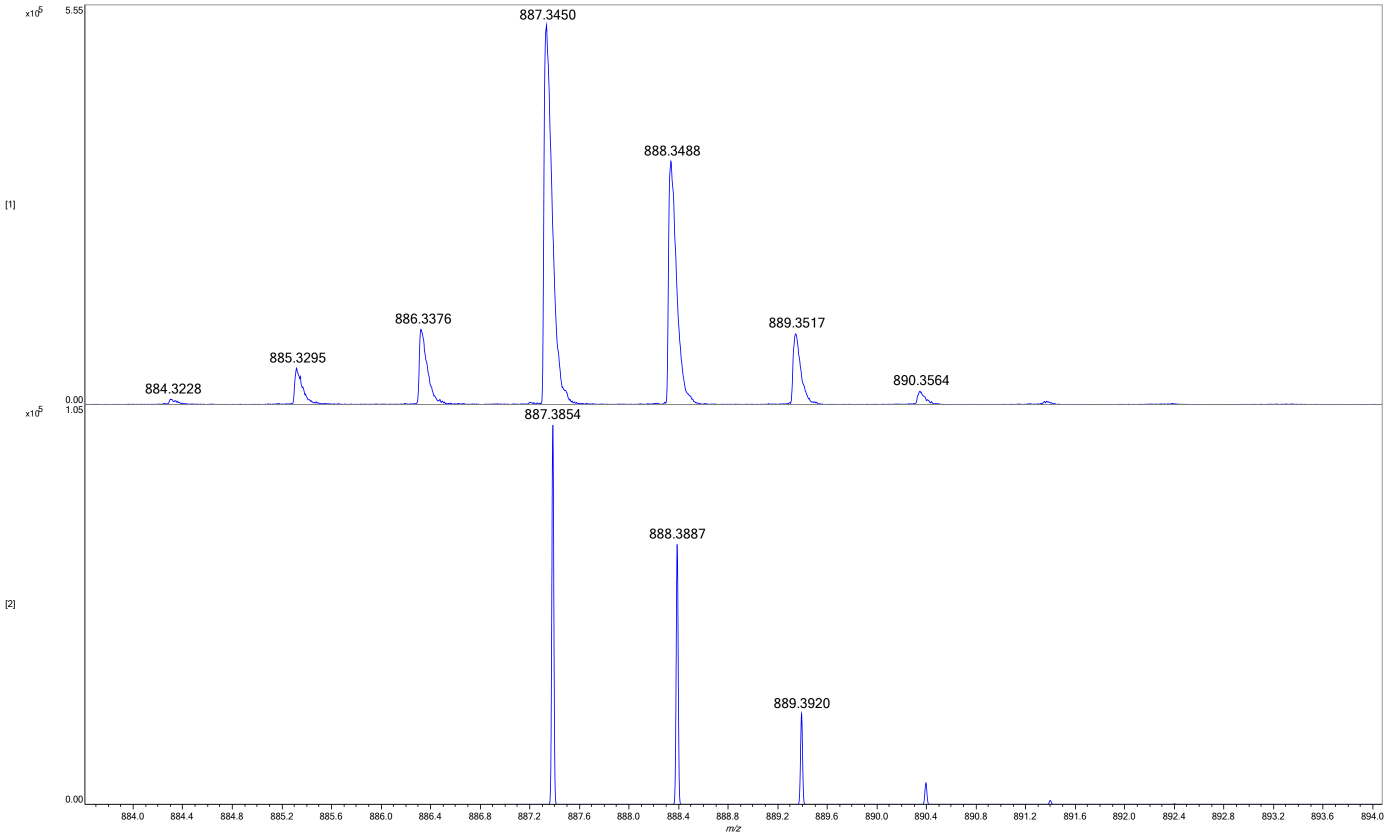

Figure S89. MALDI-MS spectrum of [**PDI57**-DH]. Additional signals come from unidentified impurities. Top: experimental, bottom: simulated for $C_{62}H_{49}DN_2O_4$.

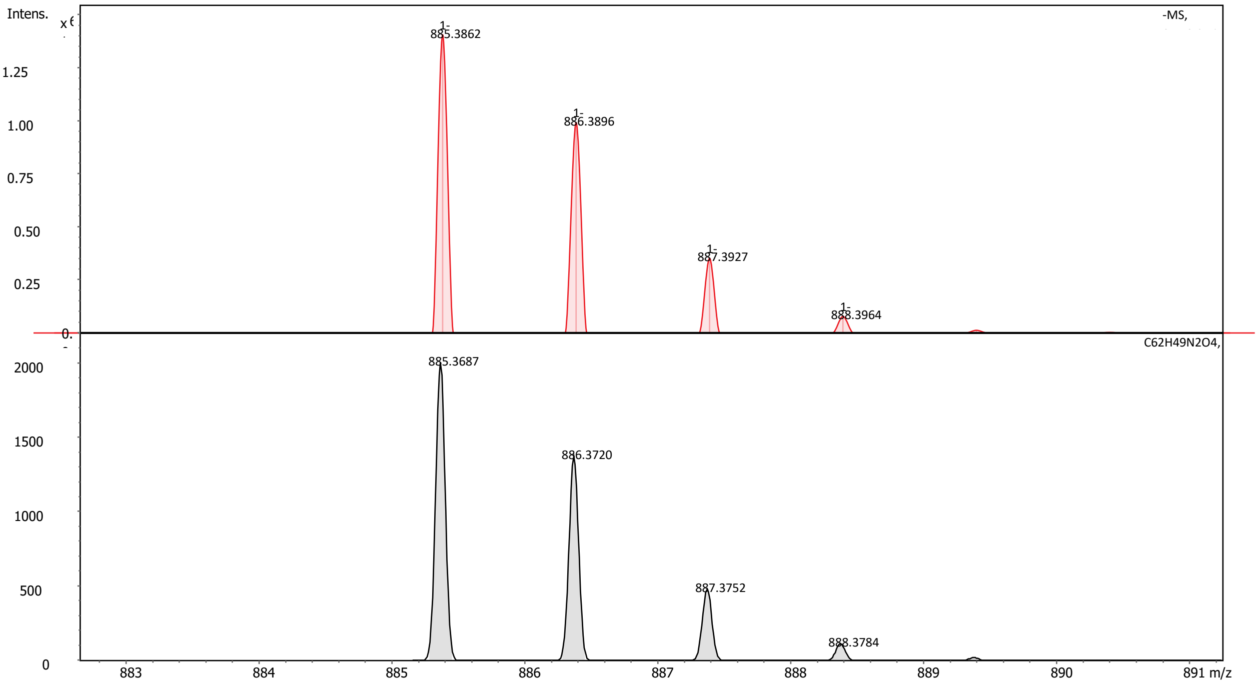

Figure S90. ESI-MS spectrum of [**PTE57**-H]$^-$.Top: experimental, bottom: simulated for $C_{62}H_{49}N_2O_4^-$.

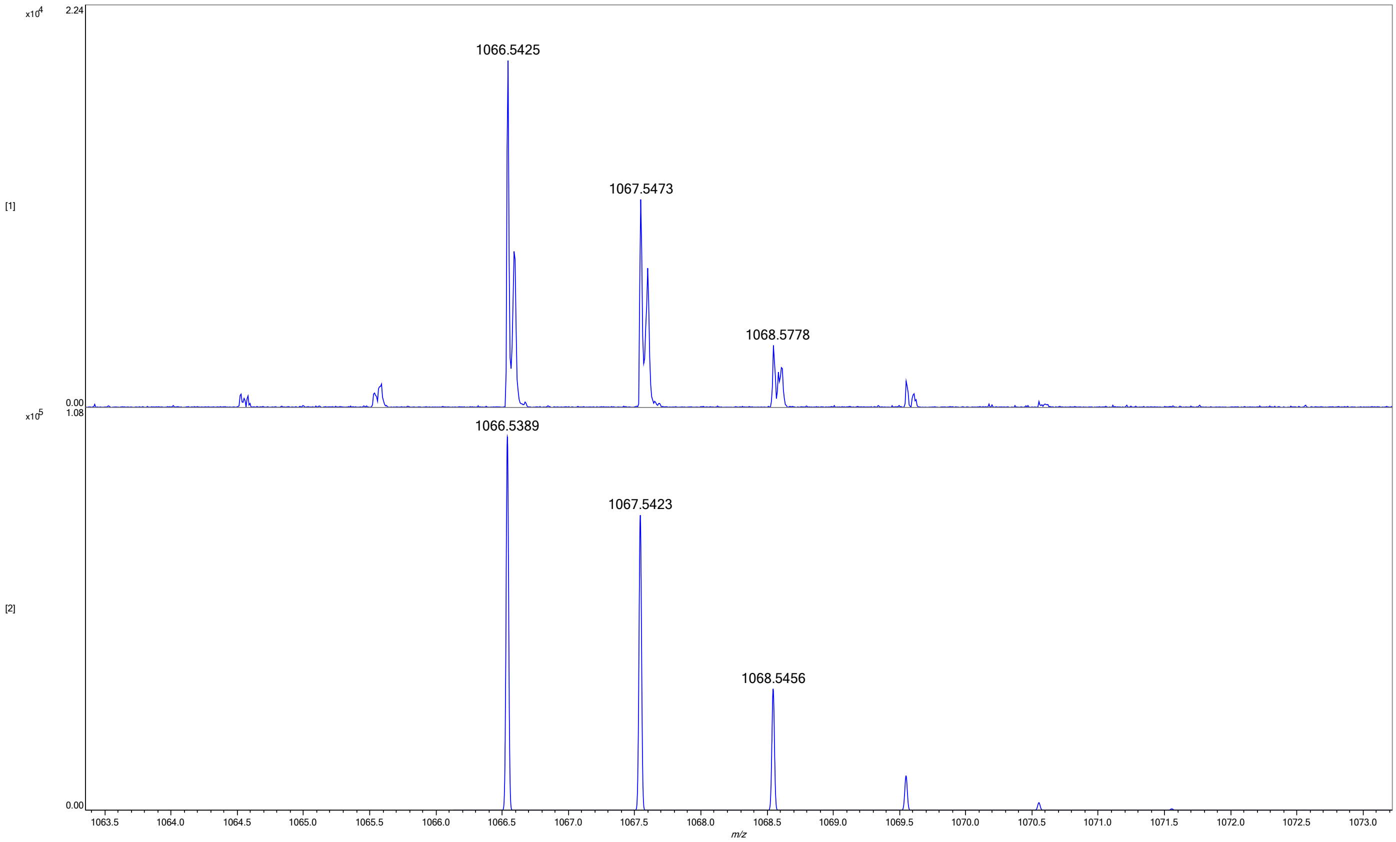

Figure S91. MALDI-MS spectrum of [**PTE55**-D_2]. Additional signals come from unidentified impurities. Top: experimental, bottom: simulated for $C_{72}H_{70}D_2O_8$.

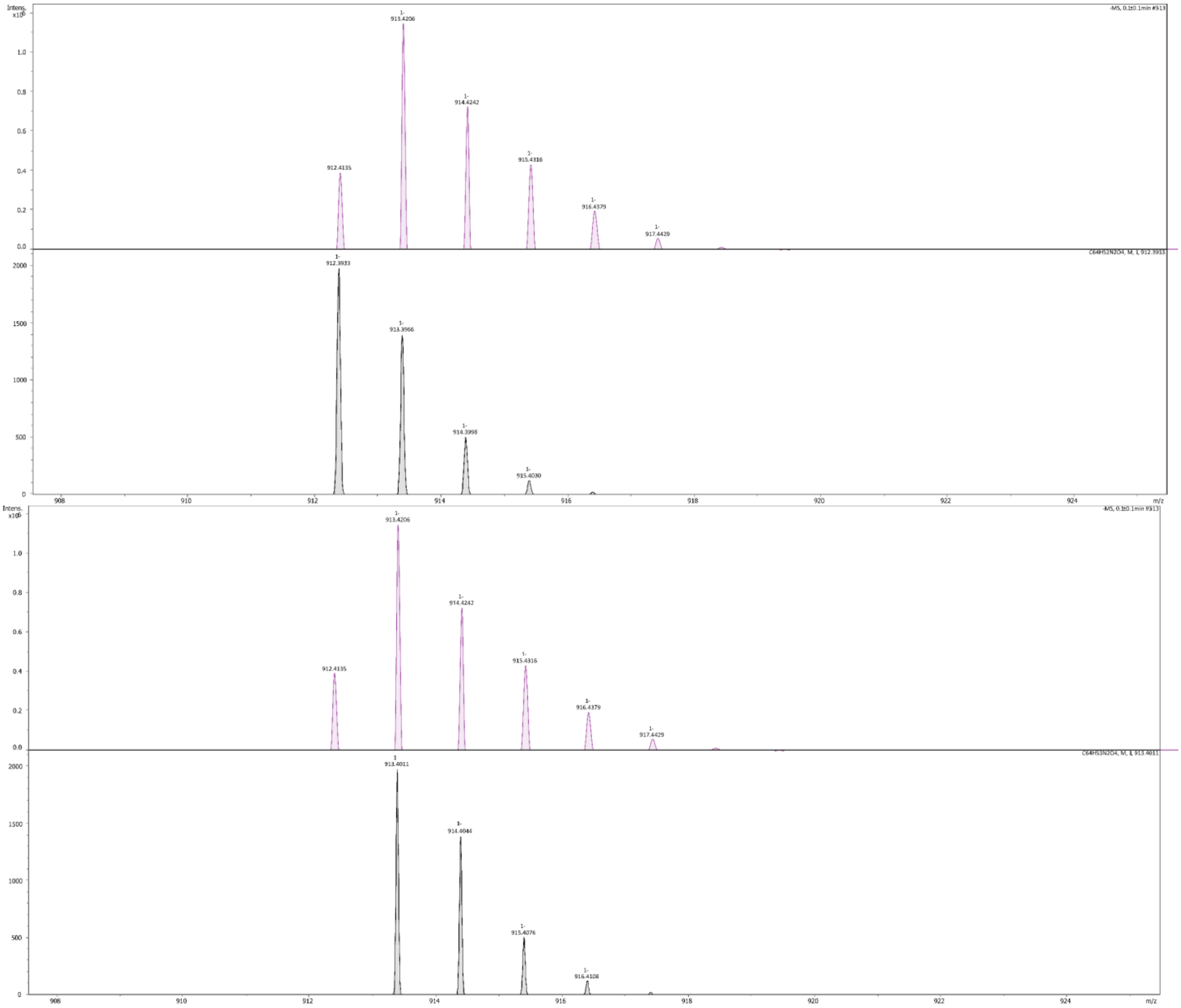

Figure S92. ESI-MS spectra recorded during measurement of [**PDI55**]$^{2-}$ sample. of [**PDI55**]$^{\bullet-}$. Purple: experimental, gray: simulated. The two species identified in the spectrum are [**PDI55**]$^{\bullet-}$ (m/z = 912.4135) and [**PDI55**-H]$^{-}$ (m/z = 913.4206). Gray spectra were simulated for $C_{64}H_{52}N_2O_4^{\bullet-}$ (top) and $C_{64}H_{53}N_2O_4^{-}$ (bottom). In these experiments, *m/z* corresponding to the [**PDI55**]$^{2-}$ species was not observed.

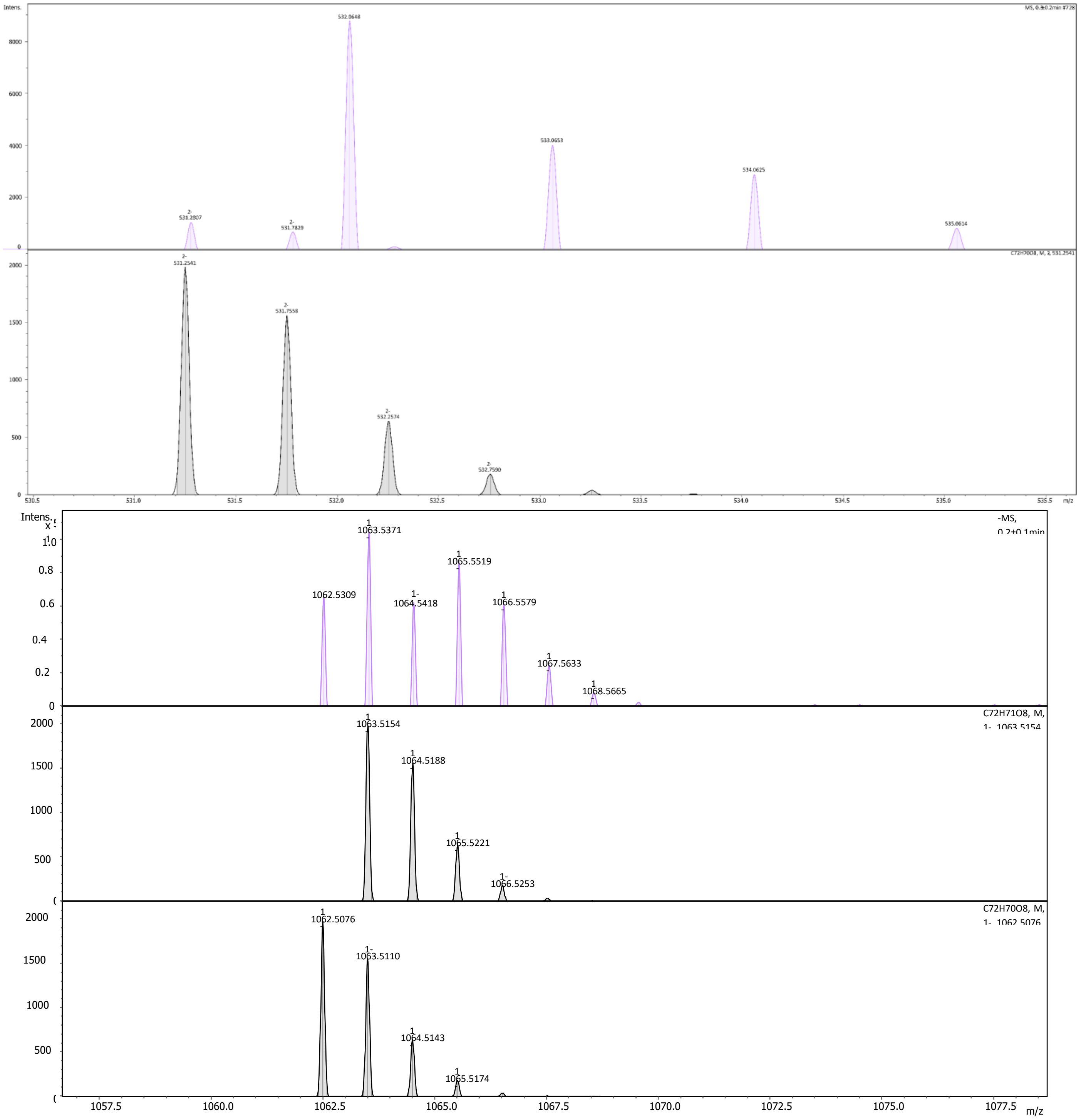

Figure S93. ESI-MS spectrum of [**PTE55**]$^{2-}$. Purple: experimental, gray: simulated. The top spectrum corresponds to [**PTE55**]$^{2-}$ (m/z = 531.2807; simulated for $C_{72}H_{70}O_8^{2-}$). Large purple signals were assigned as unidentified impurities in the spectrometer. The bottom spectrum corresponds to [**PTE55**-H]$^-$ (m/z = 1063.5154; simulated for $C_{72}H_{70}O_8^-$) and [**PTE55**]$^{\bullet-}$ (m/z = 1062.5309; simulated for $C_{72}H_{69}O_8^{\bullet-}$).

4. Computational Data

4.1. Energies and Geometries

Table S5. Data for DFT-optimized stationary points discussed in this work (CAM-B3LYP-GD3BJ/6-31G(d,p)). PDI series.

System[a]	*n*[b]	*m*[b]	SCF *E*[c]	*ZPV*[d]	*LVF*[e]	*E*[f]	*H*[f]	*G*[f]	⟨S^2⟩[g]	*.xyz[h]
			a.u.	a.u.	cm^{-1}	a.u.	a.u.	a.u.		
[**PDI55′**-H_2]	−2	1	−1868.683229	0.464240	28.57	−1868.189916	−1868.188972	−1868.276662		mofsn_55_H2(-2,1)
[**PDI55′**-H_2]	−2	3	−1868.630386	0.460955	28.17	−1868.139956	−1868.139012	−1868.228749	2.00	mofsn_55_H2(-2,3)
[**PDI55′**-H_2]	−1	2	−1868.707111	0.466186	28.99	−1868.211888	−1868.210944	−1868.299385	0.75	mofsn_55_H2(-1,2)
[**PDI55′**-H_2]	−1	4	−1868.613513	0.460927	26.56	−1868.122826	−1868.121881	−1868.212498	3.76	mofsn_55_H2(-1,4)
[**PDI55′**-H_2]	0	1	−1868.622434	0.468533	29.03	−1868.124861	−1868.123917	−1868.211946		mofsn_55_H2(0,1)
[**PDI55′**-H_2]	0	3	−1868.576227	0.465231	28.19	−1868.081695	−1868.080751	−1868.170283	2.01	mofsn_55_H2(0,3)
[**PDI55′**-H]	−3	1	−1867.931811	0.449682	28.42	−1867.453200	−1867.452255	−1867.539532		mofsn_55_H1(-3,1)
[**PDI55′**-H]	−3	3	−1867.881483	0.446214	28.39	−1867.405891	−1867.404947	−1867.494145	2.00	mofsn_55_H1(-3,3)
[**PDI55′**-H]	−2	2	−1868.061057	0.451866	28.86	−1867.580323	−1867.579379	−1867.667344	0.75	mofsn_55_H1(-2,2)
[**PDI55′**-H]	−2	4	−1867.994060	0.447957	27.54	−1867.516688	−1867.515744	−1867.605607	3.75	mofsn_55_H1(-2,4)
[**PDI55′**-H]	−1	1	−1868.077821	0.454707	28.98	−1867.594273	−1867.593329	−1867.680801		mofsn_55_H1(-1,1)
[**PDI55′**-H]	−1	3	−1868.071137	0.453306	28.73	−1867.588923	−1867.587979	−1867.676569	2.00	mofsn_55_H1(-1,3)
[**PDI55′**-H]	0	2	−1867.986332	0.455371	28.83	−1867.502037	−1867.501093	−1867.589545	0.76	mofsn_55_H1(0,2)
[**PDI55′**]	−4	1	−1867.098397	0.434960	28.18	−1866.634653	−1866.633709	−1866.720526		mofsn_55_H0(-4,1)
[**PDI55′**]	−4	3	−1867.043467	0.431195	27.46	−1866.582912	−1866.581968	−1866.670997	2.00	mofsn_55_H0(-4,3)
[**PDI55′**]	−3	2	−1867.327409	0.437879	28.85	−1866.860877	−1866.859933	−1866.947296	0.75	mofsn_55_H0(-3,2)
[**PDI55′**]	−2	1	−1867.452269	0.441179	29.11	−1866.982499	−1866.981555	−1867.068354		mofsn_55_H0(-2,1)
[**PDI55′**]	−2	3	−1867.423913	0.439002	28.65	−1866.956175	−1866.955231	−1867.043328	2.00	mofsn_55_H0(-2,3)
[**PDI55′**]	−1	2	−1867.442324	0.441619	28.78	−1866.971988	−1866.971043	−1867.058926	0.76	mofsn_55_H0(-1,2)
[**PDI55′**]	−1	4	−1867.434958	0.440433	28.50	−1866.965746	−1866.964802	−1867.053428	3.76	mofsn_55_H0(-1,4)
[**PDI55′**]	0	1	−1867.350794	0.442233	28.66	−1866.879758	−1866.878814	−1866.966363	1.74	mofsn_55_H0(0,1)
[**PDI55′**]	0	3	−1867.349667	0.442321	28.67	−1866.878543	−1866.877599	−1866.966190	2.01	mofsn_55_H0(0,3)
[**PDI57′**-H_2]	−2	1	−1868.683356	0.465272	25.68	−1868.189285	−1868.188340	−1868.275434		mofsn_57_H2(-2,1)
[**PDI57′**-H_2]	−2	3	−1868.632590	0.461694	26.23	−1868.141732	−1868.140788	−1868.229647	2.00	mofsn_57_H2(-2,3)
[**PDI57′**-H_2]	−1	2	−1868.710290	0.467168	26.54	−1868.214344	−1868.213400	−1868.301236	0.75	mofsn_57_H2(-1,2)
[**PDI57′**-H_2]	−1	4	−1868.612639	0.461605	22.38	−1868.121460	−1868.120516	−1868.210776	3.76	mofsn_57_H2(-1,4)
[**PDI57′**-H_2]	0	1	−1868.627013	0.469484	27.00	−1868.128734	−1868.127790	−1868.215193		mofsn_57_H2(0,1)
[**PDI57′**-H_2]	0	3	−1868.577200	0.466224	24.98	−1868.081913	−1868.080968	−1868.169971	2.01	mofsn_57_H2(0,3)
[**PDI57′**-H]	−3	1	−1867.894531	0.448970	23.76	−1867.416530	−1867.415586	−1867.503141		mofsn_57_H15(-3,1)
[**PDI57′**-H]	−3	3	−1867.857208	0.446414	25.22	−1867.381430	−1867.380486	−1867.469628	2.00	mofsn_57_H15(-3,3)
[**PDI57′**-H]	−2	2	−1868.043782	0.451823	25.36	−1867.563123	−1867.562179	−1867.650076	0.75	mofsn_57_H15(-2,2)
[**PDI57′**-H]	−2	4	−1867.987559	0.448413	23.58	−1867.509939	−1867.508995	−1867.598337	3.76	mofsn_57_H15(-2,4)
[**PDI57′**-H]	−1	1	−1868.070654	0.453923	25.43	−1867.587897	−1867.586953	−1867.674420	0.05	mofsn_57_H15(-1,1)
[**PDI57′**-H]	−1	3	−1868.061380	0.453576	23.62	−1867.578959	−1867.578015	−1867.666516	2.00	mofsn_57_H15(-1,3)
[**PDI57′**-H]	0	2	−1867.982238	0.455493	24.69	−1867.497898	−1867.496954	−1867.585254	0.78	mofsn_57_H15(0,2)
[**PDI57′**]	−4	1	−1867.058776	0.434304	23.17	−1866.595581	−1866.594637	−1866.681737		mofsn_57_H0(-4,1)
[**PDI57′**]	−4	3	−1867.012302	0.431712	26.35	−1866.551278	−1866.550334	−1866.639208	2.00	mofsn_57_H0(-4,3)
[**PDI57′**]	−3	2	−1867.298264	0.437584	26.30	−1866.832009	−1866.831065	−1866.918457	0.75	mofsn_57_H0(-3,2)
[**PDI57′**]	−3	4	−1867.239231	0.434028	20.91	−1866.776026	−1866.775082	−1866.864342	3.75	mofsn_57_H0(-3,4)
[**PDI57′**]	−2	1	−1867.425648	0.439521	25.97	−1866.957470	−1866.956526	−1867.043448	0.26	mofsn_57_H0(-2,1)
[**PDI57′**]	−2	3	−1867.419510	0.439729	24.87	−1866.951159	−1866.950215	−1867.038067	2.00	mofsn_57_H0(-2,3)
[**PDI57′**]	−1	2	−1867.441765	0.441914	26.11	−1866.971206	−1866.970262	−1867.057996	0.76	mofsn_57_H0(-1,2)
[**PDI57′**]	−1	4	−1867.422978	0.440669	23.33	−1866.953595	−1866.952651	−1867.041182	3.76	mofsn_57_H0(-1,4)
[**PDI57′**]	0	1	−1867.351490	0.443046	25.52	−1866.879758	−1866.878814	−1866.966127	1.09	mofsn_57_H0(0,1)
[**PDI57′**]	0	3	−1867.340229	0.442254	23.71	−1866.869208	−1866.868263	−1866.956831	2.02	mofsn_57_H0(0,3)
[**PDI77′**-H_2]	−2	1	−1868.683283	0.466332	22.80	−1868.188429	−1868.187485	−1868.273996		mofsn_77_H2(-2,1)
[**PDI77′**-H_2]	−2	3	−1868.635871	0.462256	24.13	−1868.144740	−1868.143796	−1868.231836	2.00	mofsn_77_H2(-2,3)
[**PDI77′**-H_2]	−1	2	−1868.713809	0.468184	24.30	−1868.217112	−1868.216167	−1868.303390	0.75	mofsn_77_H2(-1,2)
[**PDI77′**-H_2]	−1	4	−1868.611579	0.462107	8.02	−1868.119938	−1868.118994	−1868.209973	3.75	mofsn_77_H2(-1,4)
[**PDI77′**-H_2]	0	1	−1868.632581	0.470476	25.45	−1868.133566	−1868.132622	−1868.219363		mofsn_77_H2(0,1)
[**PDI77′**-H_2]	0	3	−1868.577670	0.467118	21.62	−1868.081712	−1868.080768	−1868.169329	2.00	mofsn_77_H2(0,3)
[**PDI77′**-H]	−3	1	−1867.889277	0.450025	18.73	−1867.410506	−1867.409562	−1867.496664		mofsn_77_H1(-3,1)
[**PDI77′**-H]	−3	3	−1867.854078	0.446394	22.34	−1867.378697	−1867.377752	−1867.466036	2.01	mofsn_77_H1(-3,3)
[**PDI77′**-H]	−2	2	−1868.042440	0.452833	21.28	−1867.561040	−1867.560096	−1867.647495	0.75	mofsn_77_H1(-2,2)
[**PDI77′**-H]	−2	4	−1867.989107	0.448955	20.76	−1867.511253	−1867.510308	−1867.598828	3.76	mofsn_77_H1(-2,4)
[**PDI77′**-H]	−1	1	−1868.073666	0.454939	22.20	−1867.590148	−1867.589204	−1867.676145	0.01	mofsn_77_H1(-1,1)
[**PDI77′**-H]	−1	3	−1868.062687	0.454566	20.09	−1867.579536	−1867.578592	−1867.666580	2.00	mofsn_77_H1(-1,3)
[**PDI77′**-H]	0	2	−1867.985716	0.456474	21.21	−1867.500644	−1867.499700	−1867.587457	0.77	mofsn_77_H1(0,2)
[**PDI77′**]	−4	1	−1867.018568	0.433550	21.17	−1866.556004	−1866.555060	−1866.642474		mofsn_77_H0(-4,1)
[**PDI77′**]	−4	3	−1866.975185	0.429180	21.46	−1866.516673	−1866.515728	−1866.604652	2.01	mofsn_77_H0(-4,3)

System[a]	n[b]	m[b]	SCF E[c]	ZPV[d]	LVF[e]	E[f]	H[f]	G[f]	⟨S^2⟩[g]	*.xyz[h]
			a.u.	a.u.	cm^{-1}	a.u.	a.u.	a.u.		
[**PDI77′**]	−3	2	−1867.257630	0.436354	24.11	−1866.792465	−1866.791521	−1866.879269	0.75	mofsn_77_H0(-3,2)
[**PDI77′**]	−3	4	−1867.222453	0.434785	30.33	−1866.758590	−1866.757646	−1866.846238	3.75	mofsn_77_H0(-3,4)
[**PDI77′**]	−2	1	−1867.405713	0.439341	25.88	−1866.937757	−1866.936813	−1867.023568	1.01	mofsn_77_H0(-2,1)
[**PDI77′**]	−2	3	−1867.404792	0.439490	25.46	−1866.936705	−1866.935761	−1867.023508	2.01	mofsn_77_H0(-2,3)
[**PDI77′**]	−1	2	−1867.433708	0.441299	25.37	−1866.963774	−1866.962830	−1867.050549	0.81	mofsn_77_H0(-1,2)
[**PDI77′**]	−1	4	−1867.411195	0.440856	16.98	−1866.941664	−1866.940720	−1867.029395	3.76	mofsn_77_H0(-1,4)
[**PDI77′**]	0	1	−1867.352354	0.444628	27.12	−1866.879230	−1866.878285	−1866.965079	0.09	mofsn_77_H0(0,1)
[**PDI77′**]	0	3	−1867.334143	0.442673	20.58	−1866.862761	−1866.861816	−1866.950335	2.01	mofsn_77_H0(0,3)

[a] Structure code. [b] System charge (n) and multiplicity (m). [c] Self-consistent field electronic energy. [d] Zero-point vibrational energy. [e] Lowest vibrational frequency. [f] Thermodynamic functions. [g] After annihilation of the first spin contaminant. [h] XYZ file containing the Cartesian coordinates. XYZ files are located in the PDI\ and PTE\ subfolders, respectively.

Table S6. Data for DFT-optimized stationary points discussed in this work (CAM-B3LYP-GD3BJ/6-31G(d,p)). PTE series.

System[a]	*n*[b]	*m*[b]	SCF *E*[c]	*ZPV*[d]	*LVF*[e]	*E*[f]	*H*[f]	*G*[f]	⟨S^2⟩[g]	*.xyz[h]
			a.u.	a.u.	cm^{-1}	a.u.	a.u.	a.u.		
**[PTE55′**-H$_2$**]**	−2	1	−2218.267949	0.602113	21.92	−2217.625310	−2217.624366	−2217.738726		mofsn_55_H2(-2,1)
**[PTE55′**-H$_2$**]**	−2	3	−2218.227669	0.600368	21.67	−2217.586561	−2217.585617	−2217.701130	2.00	mofsn_55_H2(-2,3)
**[PTE55′**-H$_2$**]**	−1	2	−2218.316226	0.605274	22.19	−2217.670508	−2217.669564	−2217.784733	0.75	mofsn_55_H2(-1,2)
**[PTE55′**-H$_2$**]**	−1	4	−2218.229561	0.601111	20.24	−2217.587482	−2217.586538	−2217.703344	3.76	mofsn_55_H2(-1,4)
**[PTE55′**-H$_2$**]**	0	1	−2218.260121	0.608826	21.50	−2217.610871	−2217.609927	−2217.724603		mofsn_55_H2(0,1)
**[PTE55′**-H$_2$**]**	0	3	−2218.207629	0.604709	20.89	−2217.562110	−2217.561166	−2217.677762	2.01	mofsn_55_H2(0,3)
**[PTE55′**-H**]**	−3	1	−2217.509086	0.586865	21.04	−2216.881843	−2216.880899	−2216.994652		mofsn_55_H1(-3,1)
**[PTE55′**-H**]**	−3	3	−2217.473550	0.584774	21.96	−2216.848050	−2216.847106	−2216.962743	2.00	mofsn_55_H1(-3,3)
**[PTE55′**-H**]**	−2	2	−2217.652570	0.590027	21.71	−2217.022198	−2217.021253	−2217.136317	0.75	mofsn_55_H1(-2,2)
**[PTE55′**-H**]**	−2	4	−2217.595253	0.587715	21.04	−2216.966972	−2216.966028	−2217.081475	3.75	mofsn_55_H1(-2,4)
**[PTE55′**-H**]**	−1	1	−2217.694200	0.594210	19.27	−2217.059788	−2217.058843	−2217.173186		mofsn_55_H1(-1,1)
**[PTE55′**-H**]**	−1	3	−2217.682044	0.592376	21.96	−2217.049384	−2217.048440	−2217.163565	2.00	mofsn_55_H1(-1,3)
**[PTE55′**-H**]**	0	2	−2217.623816	0.595539	21.59	−2216.987989	−2216.987045	−2217.102014	0.76	mofsn_55_H1(0,2)
**[PTE55′]**	−4	1	−2216.670456	0.570950	21.75	−2216.059050	−2216.058105	−2216.171863		mofsn_55_H0(-4,1)
**[PTE55′]**	−4	3	−2216.630144	0.568371	21.40	−2216.021030	−2216.020085	−2216.135757	2.00	mofsn_55_H0(-4,3)
**[PTE55′]**	−3	2	−2216.907867	0.575041	21.99	−2216.292657	−2216.291713	−2216.405860	0.75	mofsn_55_H0(-3,2)
**[PTE55′]**	−2	1	−2217.050186	0.579854	19.96	−2216.430486	−2216.429542	−2216.542816		mofsn_55_H0(-2,1)
**[PTE55′]**	−2	3	−2217.018968	0.577220	21.50	−2216.401583	−2216.400639	−2216.515514	2.00	mofsn_55_H0(-2,3)
**[PTE55′]**	−1	2	−2217.059201	0.580940	19.16	−2216.438202	−2216.437258	−2216.551872	0.76	mofsn_55_H0(-1,2)
**[PTE55′]**	−1	4	−2217.047870	0.579490	21.73	−2216.428257	−2216.427313	−2216.542274	3.76	mofsn_55_H0(-1,4)
**[PTE55′]**	0	1	−2216.987757	0.582256	21.66	−2216.365348	−2216.364404	−2216.478361	1.53	mofsn_55_H0(0,1)
**[PTE55′]**	0	3	−2216.987172	0.582309	21.68	−2216.364715	−2216.363771	−2216.478737	2.01	mofsn_55_H0(0,3)
**[PTE57′**-H$_2$**]**	−2	1	−2218.270330	0.603093	18.77	−2217.626919	−2217.625975	−2217.740449		mofsn_57_H2(-2,1)
**[PTE57′**-H$_2$**]**	−2	3	−2218.231136	0.601196	19.74	−2217.589462	−2217.588518	−2217.703415	2.00	mofsn_57_H2(-2,3)
**[PTE57′**-H$_2$**]**	−1	2	−2218.321143	0.606098	18.06	−2217.674770	−2217.673826	−2217.789445	0.75	mofsn_57_H2(-1,2)
**[PTE57′**-H$_2$**]**	−1	4	−2218.230040	0.601959	17.31	−2217.587311	−2217.586367	−2217.703147	3.76	mofsn_57_H2(-1,4)
**[PTE57′**-H$_2$**]**	0	1	−2218.267025	0.609694	20.98	−2217.617133	−2217.616188	−2217.730504		mofsn_57_H2(0,1)
**[PTE57′**-H$_2$**]**	0	3	−2218.210349	0.605831	19.22	−2217.563946	−2217.563001	−2217.679294	2.01	mofsn_57_H2(0,3)
**[PTE57′**-H**]**	−3	1	−2217.479281	0.586381	18.45	−2216.852462	−2216.851518	−2216.965307		mofsn_57_H15(-3,1)
**[PTE57′**-H**]**	−3	3	−2217.449482	0.584344	19.25	−2216.824276	−2216.823331	−2216.939849	2.00	mofsn_57_H15(-3,3)
**[PTE57′**-H**]**	−2	2	−2217.635066	0.589810	19.20	−2217.004919	−2217.003975	−2217.118932	0.75	mofsn_57_H15(-2,2)
**[PTE57′**-H**]**	−2	4	−2217.586616	0.587706	16.75	−2216.958430	−2216.957486	−2217.072860	3.75	mofsn_57_H15(-2,4)
**[PTE57′**-H**]**	−1	1	−2217.689178	0.594462	21.11	−2217.054580	−2217.053636	−2217.167433		mofsn_57_H15(-1,1)
**[PTE57′**-H**]**	−1	3	−2217.673164	0.592504	17.65	−2217.040314	−2217.039369	−2217.155511	2.00	mofsn_57_H15(-1,3)
**[PTE57′**-H**]**	0	2	−2217.621925	0.595605	18.44	−2216.986063	−2216.985119	−2217.100262	0.77	mofsn_57_H15(0,2)
**[PTE57′]**	−4	1	−2216.631516	0.569981	15.91	−2216.020802	−2216.019858	−2216.135377		mofsn_57_H0(-4,1)
**[PTE57′]**	−4	3	−2216.601823	0.568042	19.69	−2215.992788	−2215.991844	−2216.108772	2.00	mofsn_57_H0(-4,3)
**[PTE57′]**	−3	2	−2216.884154	0.575042	19.61	−2216.268929	−2216.267985	−2216.382069	0.75	mofsn_57_H0(-3,2)
**[PTE57′]**	−3	4	−2216.836771	0.572766	18.53	−2216.223534	−2216.222590	−2216.337891	3.75	mofsn_57_H0(-3,4)
**[PTE57′]**	−2	1	−2217.030996	0.579581	21.19	−2216.411462	−2216.410518	−2216.523858		mofsn_57_H0(-2,1)
**[PTE57′]**	−2	3	−2217.016387	0.578243	18.41	−2216.398054	−2216.397110	−2216.511938	2.00	mofsn_57_H0(-2,3)
**[PTE57′]**	−1	2	−2217.064536	0.581809	20.34	−2216.442709	−2216.441765	−2216.556517	0.76	mofsn_57_H0(-1,2)
**[PTE57′]**	−1	4	−2217.035416	0.579517	17.64	−2216.415708	−2216.414763	−2216.530639	3.76	mofsn_57_H0(-1,4)
**[PTE57′]**	0	1	−2216.992683	0.583333	19.50	−2216.369277	−2216.368333	−2216.482389	0.70	mofsn_57_H0(0,1)
**[PTE57′]**	0	3	−2216.980143	0.582357	18.04	−2216.357644	−2216.356700	−2216.471915	2.01	mofsn_57_H0(0,3)
**[PTE77′**-H$_2$**]**	−2	1	−2218.272720	0.604011	17.55	−2217.628600	−2217.627656	−2217.741881		mofsn_77_H2(-2,1)
**[PTE77′**-H$_2$**]**	−2	3	−2218.235167	0.602012	18.54	−2217.592940	−2217.591996	−2217.706215	2.00	mofsn_77_H2(-2,3)
**[PTE77′**-H$_2$**]**	−1	4	−2218.228996	0.602642	14.39	−2217.585792	−2217.584848	−2217.701141	3.75	mofsn_77_H2(-1,4)
**[PTE77′**-H$_2$**]**	0	1	−2218.274680	0.610611	20.09	−2217.624113	−2217.623169	−2217.737026		mofsn_77_H2(0,1)
**[PTE77′**-H$_2$**]**	0	3	−2218.211574	0.606423	16.77	−2217.564703	−2217.563759	−2217.680255	2.01	mofsn_77_H2(0,3)
**[PTE77′**-H**]**	−3	1	−2217.477688	0.587277	15.30	−2216.850127	−2216.849183	−2216.962891		mofsn_77_H1(-3,1)
**[PTE77′**-H**]**	−3	3	−2217.444523	0.584746	16.31	−2216.819271	−2216.818327	−2216.933426	2.00	mofsn_77_H1(-3,3)
**[PTE77′**-H**]**	−2	2	−2217.637088	0.590676	17.38	−2217.006306	−2217.005361	−2217.120073	0.75	mofsn_77_H1(-2,2)
**[PTE77′**-H**]**	−2	4	−2217.589881	0.588667	15.44	−2216.960953	−2216.960009	−2217.074741	3.75	mofsn_77_H1(-2,4)
**[PTE77′**-H**]**	−1	1	−2217.694990	0.595277	19.31	−2217.059799	−2217.058855	−2217.172483		mofsn_77_H1(-1,1)
**[PTE77′**-H**]**	−1	3	−2217.677193	0.593517	16.07	−2217.043640	−2217.042696	−2217.157746	2.00	mofsn_77_H1(-1,3)
**[PTE77′**-H**]**	0	2	−2217.627873	0.596534	16.92	−2216.991318	−2216.990374	−2217.105174	0.77	mofsn_77_H1(0,2)
**[PTE77′]**	−4	1	−2216.596373	0.569395	9.53	−2215.986134	−2215.985190	−2216.101939		mofsn_77_H0(-4,1)
**[PTE77′]**	−4	3	−2216.567441	0.566624	15.11	−2215.959828	−2215.958884	−2216.075243	2.01	mofsn_77_H0(-4,3)
**[PTE77′]**	−3	2	−2216.850090	0.574040	17.95	−2216.235762	−2216.234818	−2216.349028	0.75	mofsn_77_H0(-3,2)
**[PTE77′]**	−3	4	−2216.818747	0.572346	19.58	−2216.205764	−2216.204820	−2216.321458	3.75	mofsn_77_H0(-3,4)
**[PTE77′]**	−2	1	−2217.007576	0.577671	19.84	−2216.389861	−2216.388917	−2216.502239	0.56	mofsn_77_H0(-2,1)
**[PTE77′]**	−2	3	−2217.002412	0.577612	18.37	−2216.384672	−2216.383728	−2216.499046	2.01	mofsn_77_H0(-2,3)
**[PTE77′]**	−1	2	−2217.057611	0.581176	19.89	−2216.436450	−2216.435506	−2216.549938	0.77	mofsn_77_H0(-1,2)
**[PTE77′]**	−1	4	−2217.026763	0.579803	13.93	−2216.406796	−2216.405852	−2216.521938	3.75	mofsn_77_H0(-1,4)
**[PTE77′]**	0	1	−2216.994350	0.584396	19.32	−2216.369996	−2216.369051	−2216.482991	0.09	mofsn_77_H0(0,1)
**[PTE77′]**	0	3	−2216.977923	0.582823	16.60	−2216.355002	−2216.354058	−2216.469433	2.01	mofsn_77_H0(0,3)

[a] Structure code. [b] System charge (*n*) and multiplicity (*m*). [c] Self-consistent field electronic energy. [d] Zero-point vibrational energy. [e] Lowest vibrational frequency. [f] Thermodynamic functions. [g] After annihilation of the first spin contaminant. [h] XYZ file containing the Cartesian coordinates. XYZ files are located in the PDI\ and PTE\ subfolders, respectively.

Table S7. Structural parameters for selected DFT-optimized geometries of annulated PDIs and PTEs.

**Structure** [a]	**Bond distances (Å)** [b]							
	**a**	**b**	**c**	**d**	**e**	**f**	**g**	**h**
^{1}**[PDI55'**-H_2**]**	1.501	1.503	1.373	1.476	1.478	1.218	1.215	1.469
^{1}**[PDI55']**	1.426	1.419	1.392	1.473	1.477	1.219	1.216	1.469
^{3}**[PDI55']**	1.422	1.422	1.388	1.474	1.477	1.219	1.216	1.472
^{2}**[PDI55']**$^{-}$	1.434	1.374	1.423	1.442	1.469	1.229	1.227	1.469
	1.423	1.421	1.393	1.468	1.469	1.223	1.220	1.470
^{4}**[PDI55']**$^{-}$	1.420	1.428	1.402	1.461	1.460	1.227	1.225	1.443
^{1}**[PDI55']**$^{2-}$	1.431	1.378	1.425	1.436	1.462	1.234	1.232	1.476
^{2}**[PDI55']**$^{3-}$	1.420	1.391	1.433	1.433	1.445	1.243	1.242	1.450
^{1}**[PDI55']**$^{4-}$	1.407	1.407	1.445	1.429	1.430	1.252	1.254	1.427
^{1}**[PTE55'**-H_2**]**	1.502	1.509	1.369	1.493	1.489	1.210	1.212	1.471
^{1}**[PTE55']**	1.424	1.422	1.388	1.491	1.489	1.210	1.211	1.471
^{3}**[PTE55']**	1.421	1.425	1.385	1.491	1.490	1.210	1.211	1.473
^{2}**[PTE55']**$^{-}$	1.422	1.421	1.390	1.487	1.481	1.211	1.214	1.471
	1.418	1.389	1.420	1.467	1.487	1.221	1.210	1.469
^{1}**[PTE55']**$^{2-}$	1.422	1.390	1.431	1.451	1.483	1.227	1.211	1.478
^{2}**[PTE55']**$^{3-}$	1.409	1.404	1.427	1.471	1.441	1.216	1.226	1.449
^{1}**[PTE55']**$^{4-}$	1.403	1.414	1.441	1.469	1.414	1.217	1.238	1.426

[a] CAM-B3LYP-GD3BJ/6-31G(d,p) level of theory. [b] **a**–**h** bond labels are defined in Figure 7. Two values are given for structures lacking C_2 point symmetry.

4.2. Orbitals and Densities

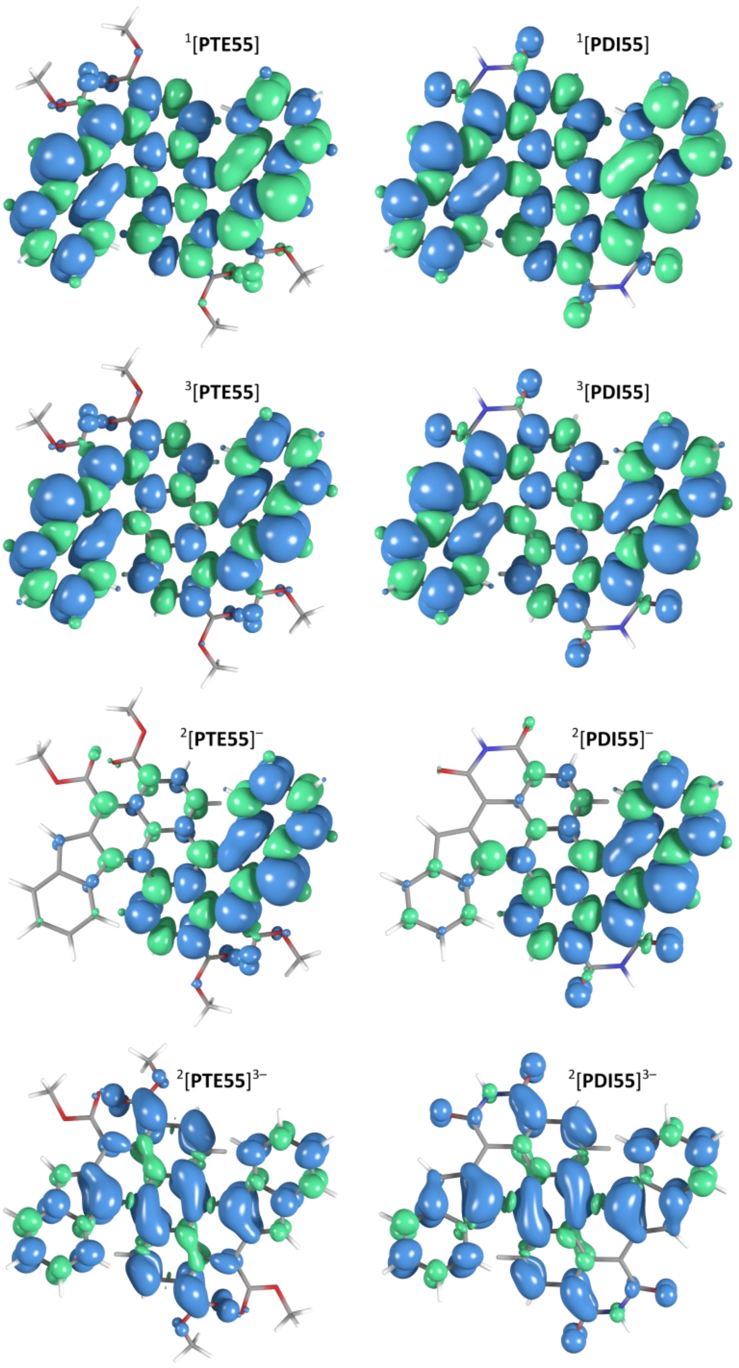

Figure S94. Spin densities in open shell states of **PTE55'** (R = H, R' = Me) and **PDI55'** (R = R' = H). Level of theory: CAM-B3LYP-GD3BJ/6-31G(d,p).

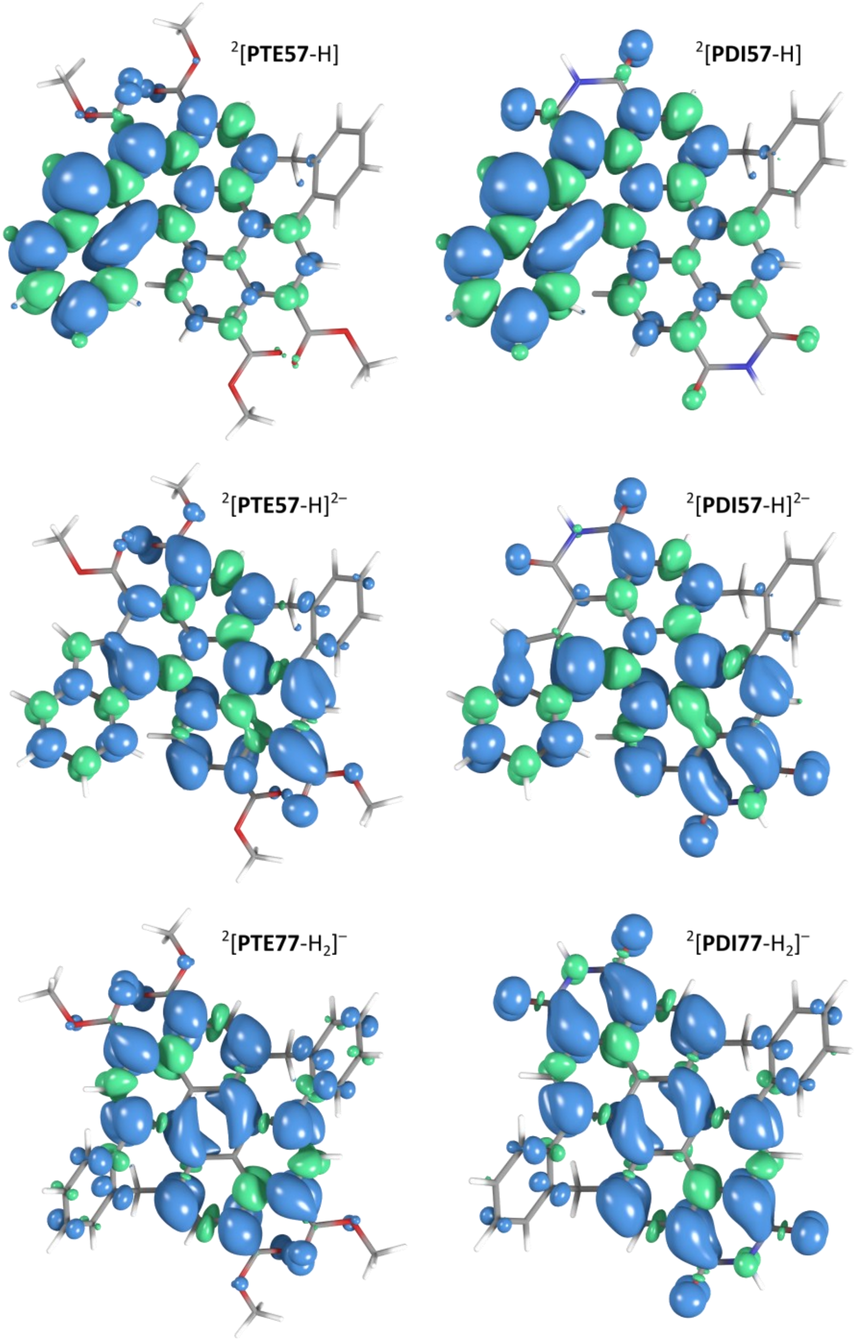

Figure S95. Spin densities in open shell states of **PTE57'** and **PTE77'** (R = H, R' = Me), and **PDI57'** and **PDI77'** (R = R' = H). Level of theory: CAM-B3LYP-GD3BJ/6-31G(d,p).

4.3. Spectroscopy

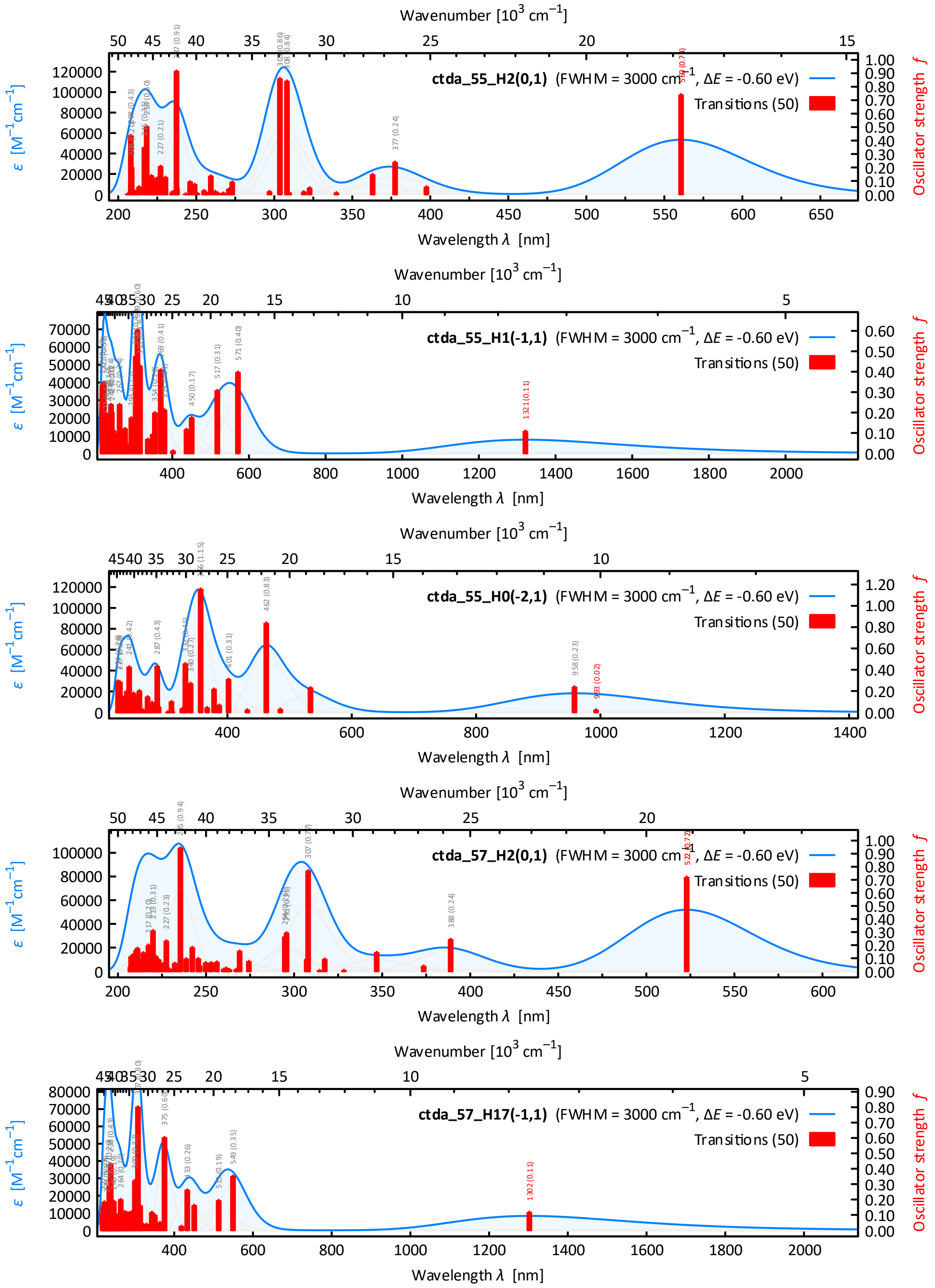

Figure S96. Calculated absorption spectra for the deprotonation sequences of **PTE55'**-H_2 and **PTE57'**-H_2 (cf. Figures S32–S34 for the corresponding experimental data). Spectra were obtained using TDA calculations (PCM(THF)/CAM-B3LYP-GD3BJ/6-31G(d,p)//CAM-B3LYP-GD3BJ/6-31G(d,p), 50 transitions, energies shifted by 0.6 eV to lower values). Panel labels are of the form ctda_mn_Hx(charge,multiplicity), wherein mn is the fusion pattern, and Hx denotes the protonation status.

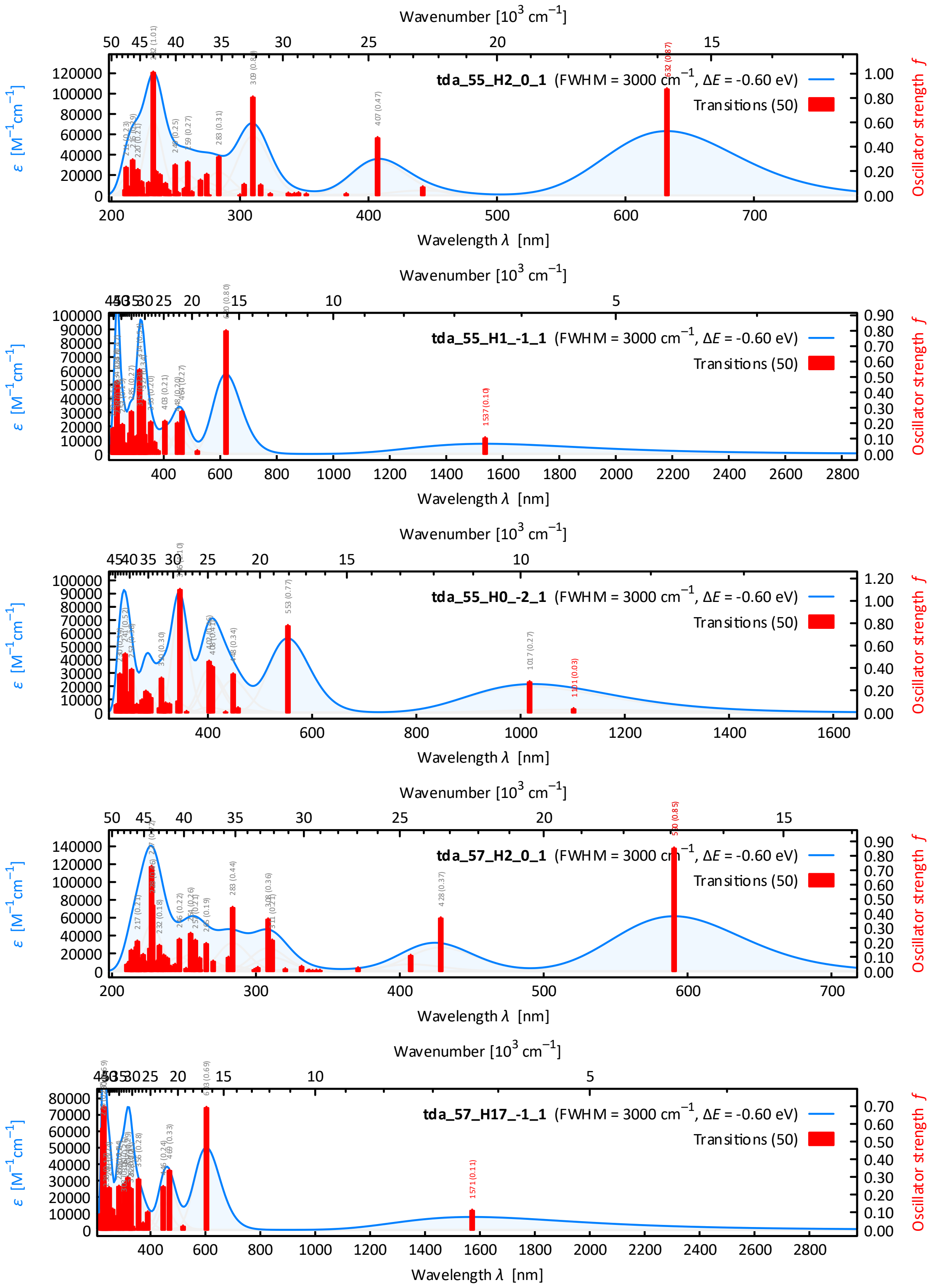

Figure S97. Calculated absorption spectra for the deprotonation sequences of **PDI55'**-H_2 and **PDI57'**-H_2 (cf. Figures S36, S41 for the corresponding experimental data). Spectra were obtained using TDA calculations (PCM(THF)/CAM-B3LYP-GD3BJ/6-31G(d,p)//CAM-B3LYP-GD3BJ/6-31G(d,p)), 50 transitions, energies shifted by 0.6 eV to lower values). Panel labels are of the form tda_mn_Hx_charge_multiplicity, wherein mn is the fusion pattern, and Hx denotes the protonation status.

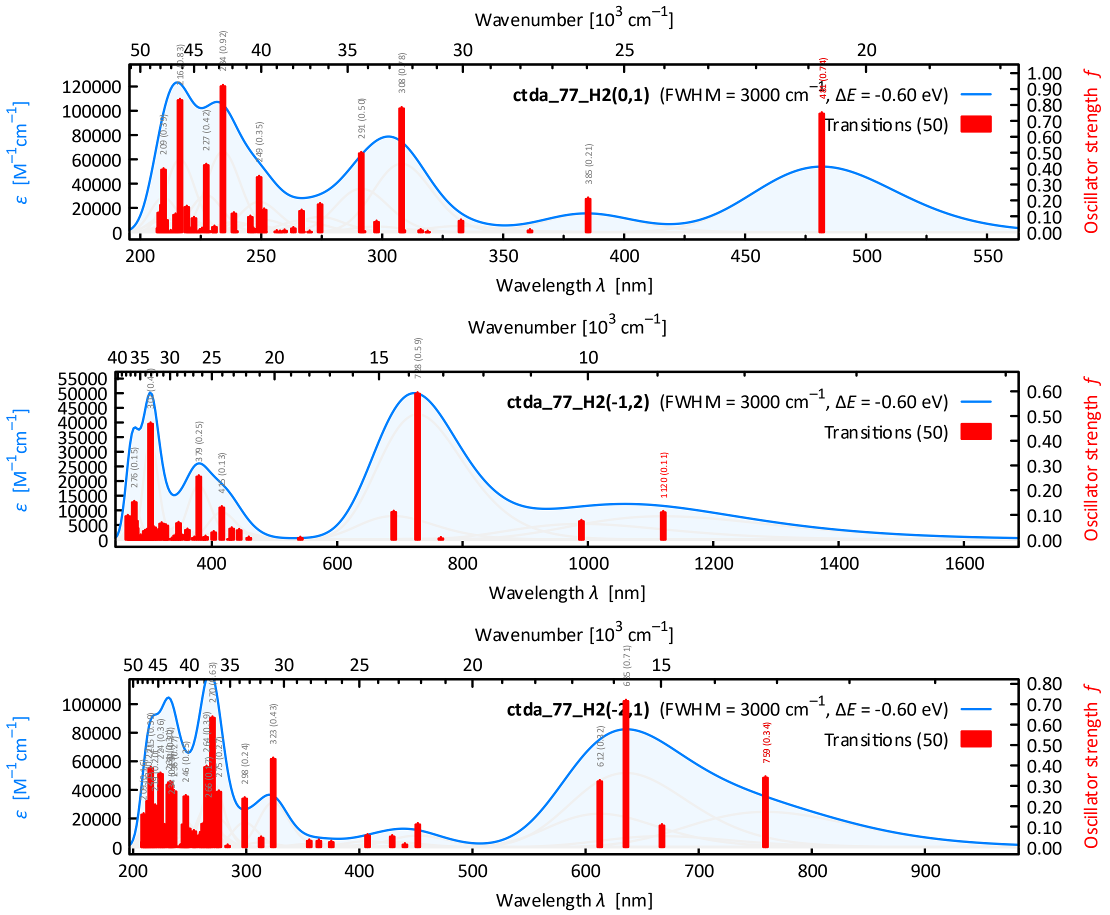

Figure S98. Calculated absorption spectra for the reduction sequence of **PTE77'**-H_2 (cf. Figures S35, S53 for the corresponding experimental data). Spectra were obtained using TDA calculations (PCM(THF)/CAM-B3LYP-GD3BJ/6-31G(d,p)//CAM-B3LYP-GD3BJ/6-31G(d,p), 50 transitions, energies shifted by 0.6 eV to lower values). Panel labels are of the form ctda_mn_Hx(charge,multiplicity), wherein mn is the fusion pattern, and Hx denotes the protonation status.

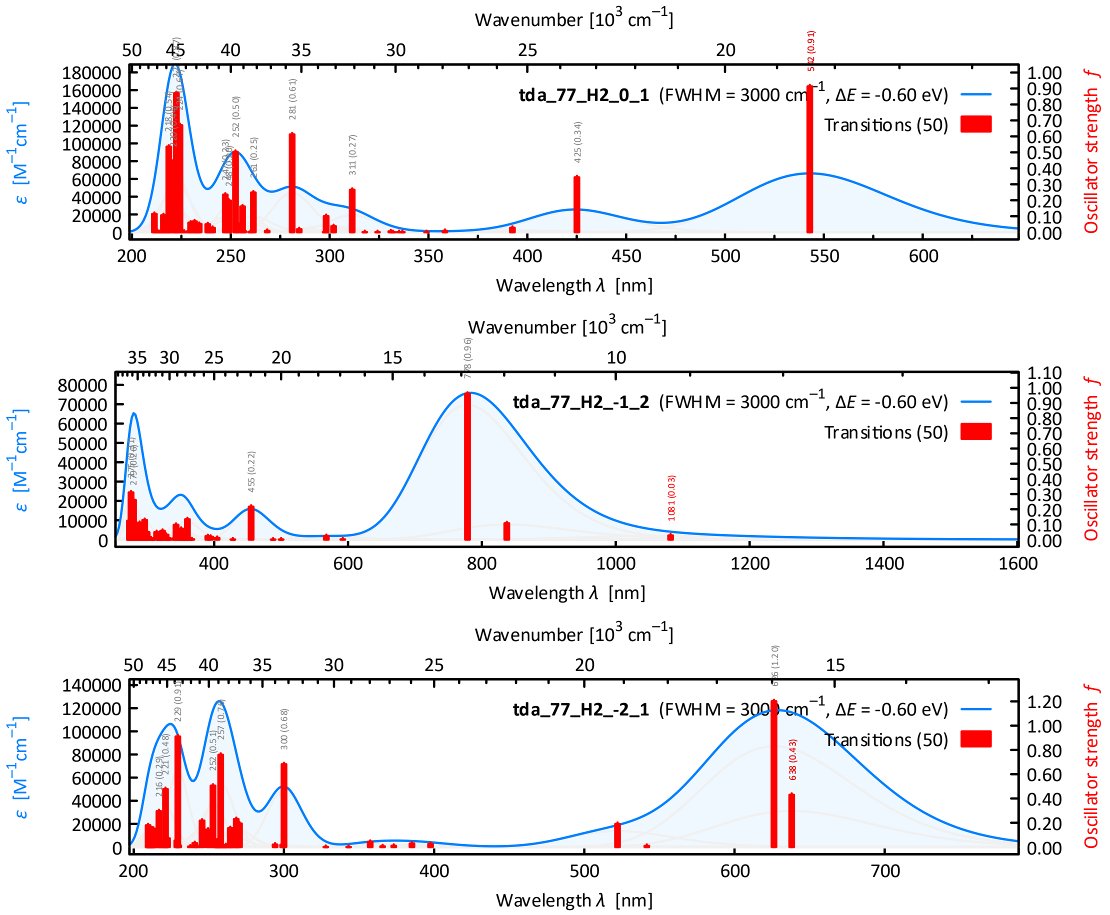

Figure S99. Calculated absorption spectra for the reduction sequence of **PDI77'**-H_2 (cf. Figures S46–S48 for the corresponding experimental data). Spectra were obtained using TDA calculations (PCM(THF)/CAM-B3LYP-GD3BJ/6-31G(d,p)//CAM-B3LYP-GD3BJ/6-31G(d,p), 50 transitions, energies shifted by 0.6 eV to lower values). Panel labels are of the form tda_mn_Hx_charge_multiplicity, wherein mn is the fusion pattern, and Hx denotes the protonation status.

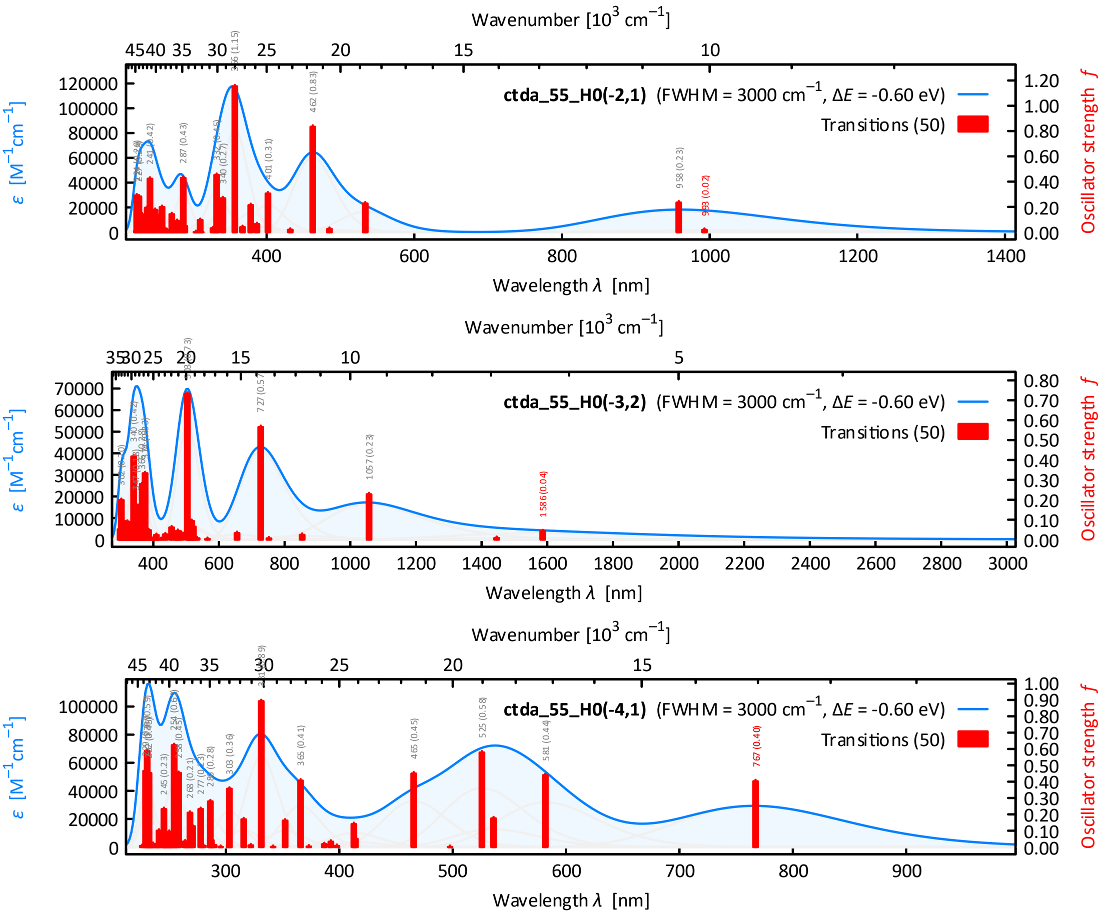

Figure S100. Calculated absorption spectra for the reduction sequence of [**PTE55'**]$^{2-}$ (cf. Figure S33 for the corresponding experimental data). Spectra were obtained using TDA calculations (PCM(THF)/CAM-B3LYP-GD3BJ/6-31G(d,p)//CAM-B3LYP-GD3BJ/6-31G(d,p), 50 transitions, energies shifted by 0.6 eV to lower values). Panel labels are of the form ctda_mn_Hx(charge,multiplicity), wherein mn is the fusion pattern, and Hx denotes the protonation status.

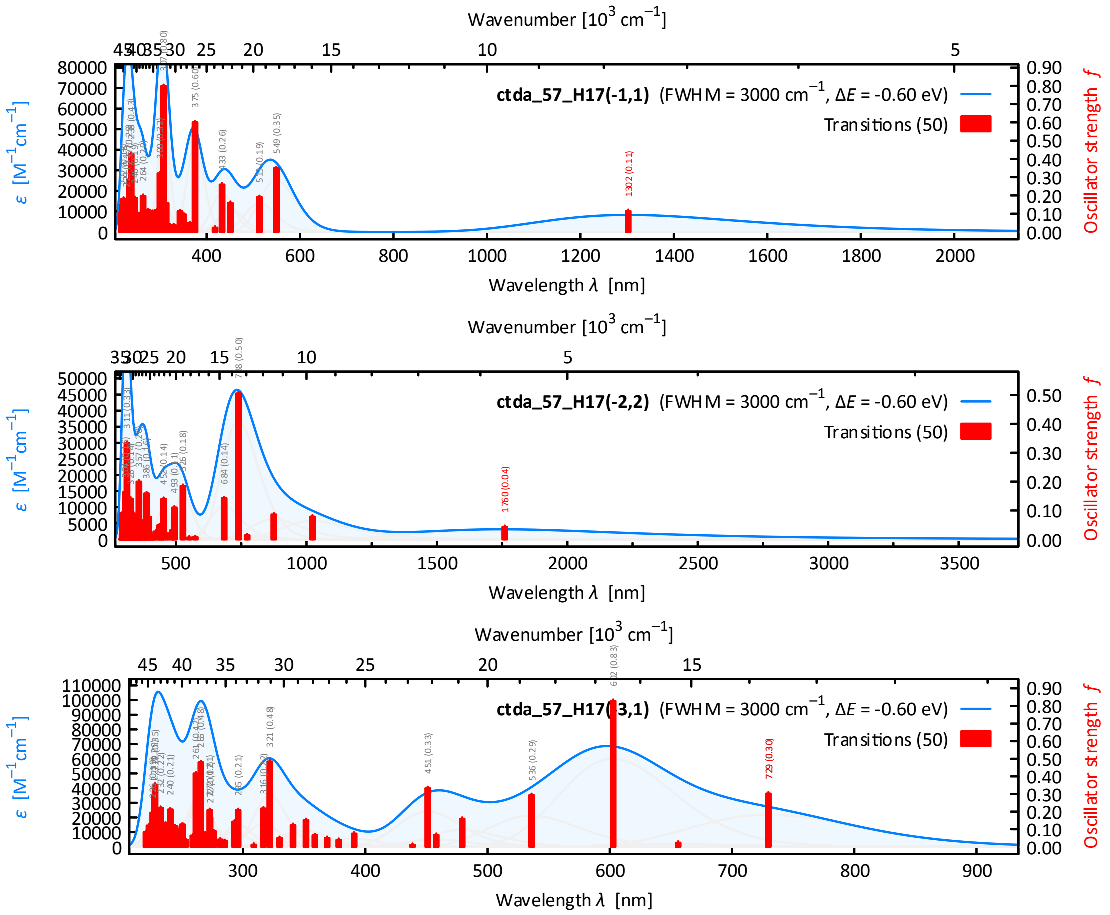

Figure S101. Calculated absorption spectra for the reduction sequence of [**PTE57'**-H]⁻ (cf. Figure S34 for the corresponding experimental data). Spectra were obtained using TDA calculations (PCM(THF)/CAM-B3LYP-GD3BJ/6-31G(d,p)//CAM-B3LYP-GD3BJ/6-31G(d,p), 50 transitions, energies shifted by 0.6 eV to lower values). Panel labels are of the form ctda_mn_Hx(charge,multiplicity), wherein mn is the fusion pattern, and Hx denotes the protonation status.

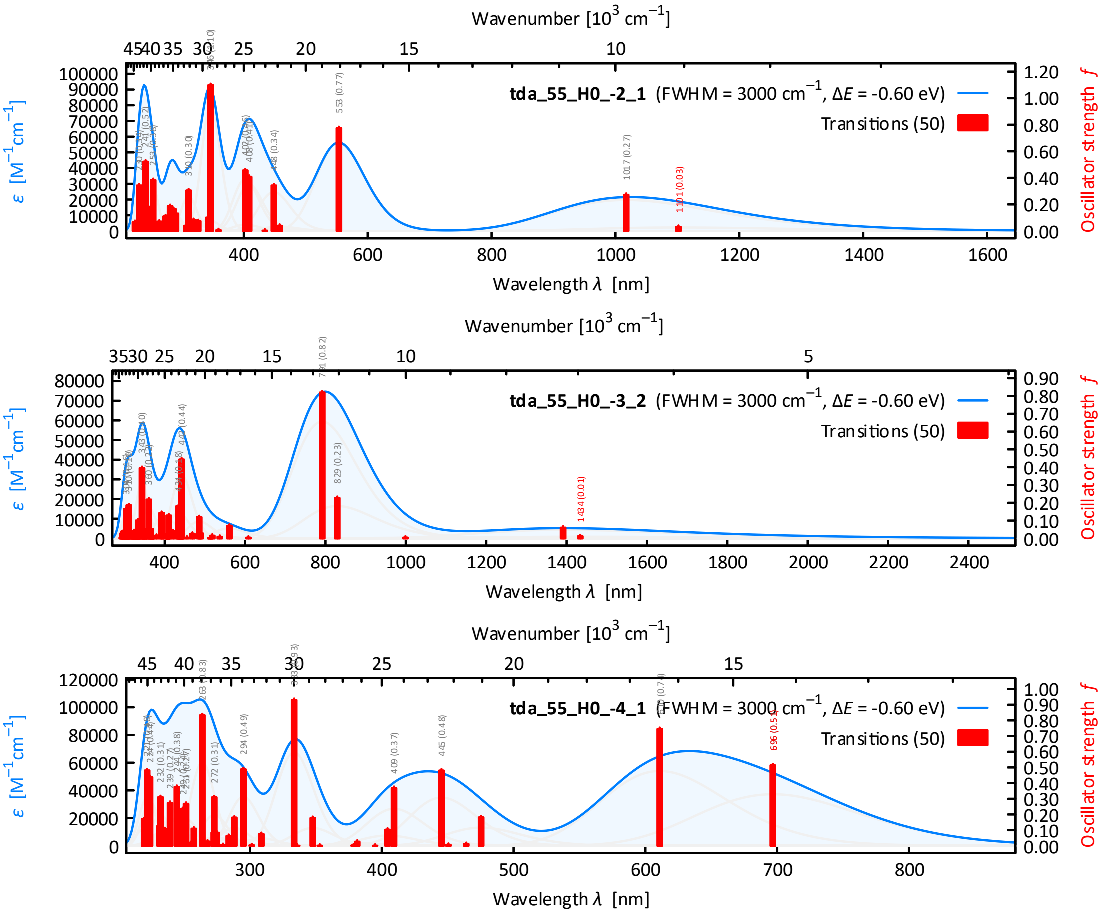

Figure S102. Calculated absorption spectra for the reduction sequence of [**PDI55'**]$^{2-}$ (cf. Figures S37–S39 for the corresponding experimental data). Spectra were obtained using TDA calculations (PCM(THF)/CAM-B3LYP-GD3BJ/6-31G(d,p)//CAM-B3LYP-GD3BJ/6-31G(d,p), 50 transitions, energies shifted by 0.6 eV to lower values). Panel labels are of the form tda_mn_Hx_charge_multiplicity, wherein mn is the fusion pattern, and Hx denotes the protonation status.

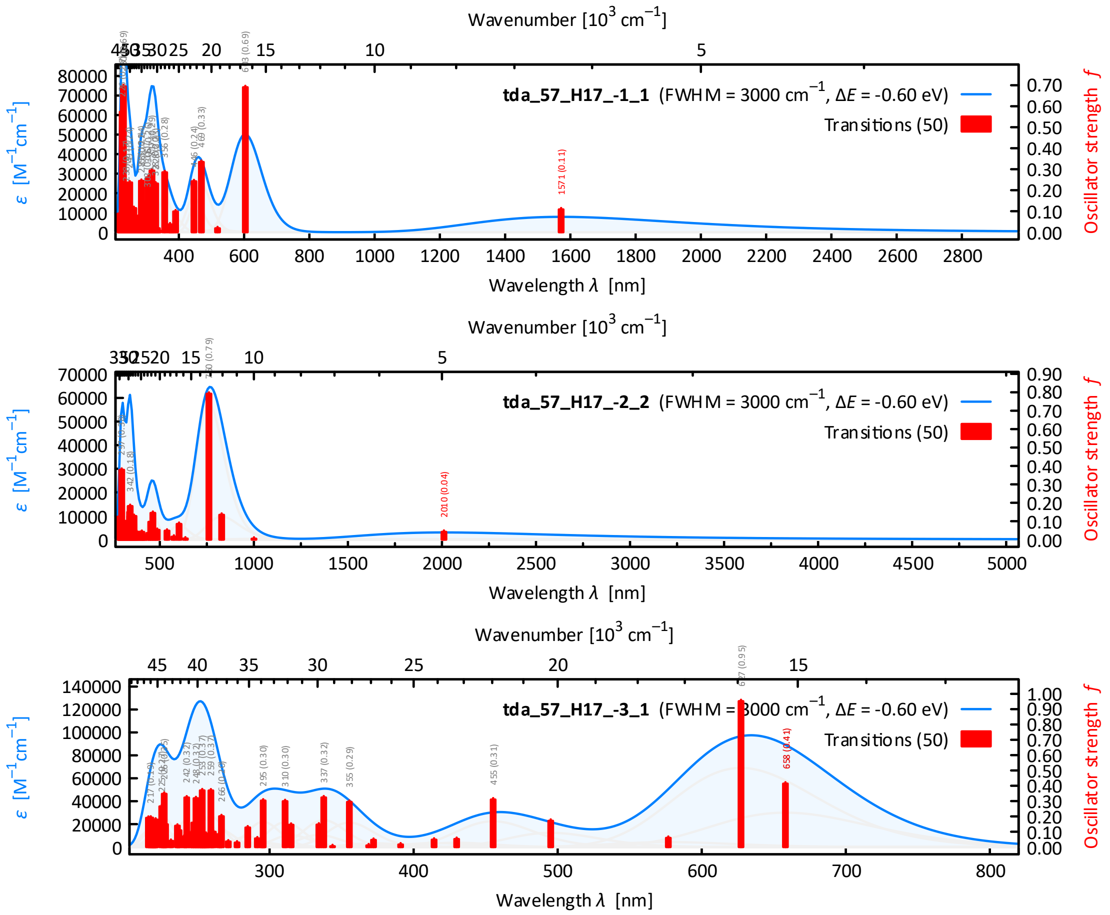

Figure S103. Calculated absorption spectra for the reduction sequence of [**PDI57'**-H]⁻ (cf. Figures S41–S43 for the corresponding experimental data). Spectra were obtained using TDA calculations (PCM(THF)/CAM-B3LYP-GD3BJ/6-31G(d,p)//CAM-B3LYP-GD3BJ/6-31G(d,p), 50 transitions, energies shifted by 0.6 eV to lower values). Panel labels are of the form tda_mn_Hx_charge_multiplicity, wherein mn is the fusion pattern, and Hx denotes the protonation status.

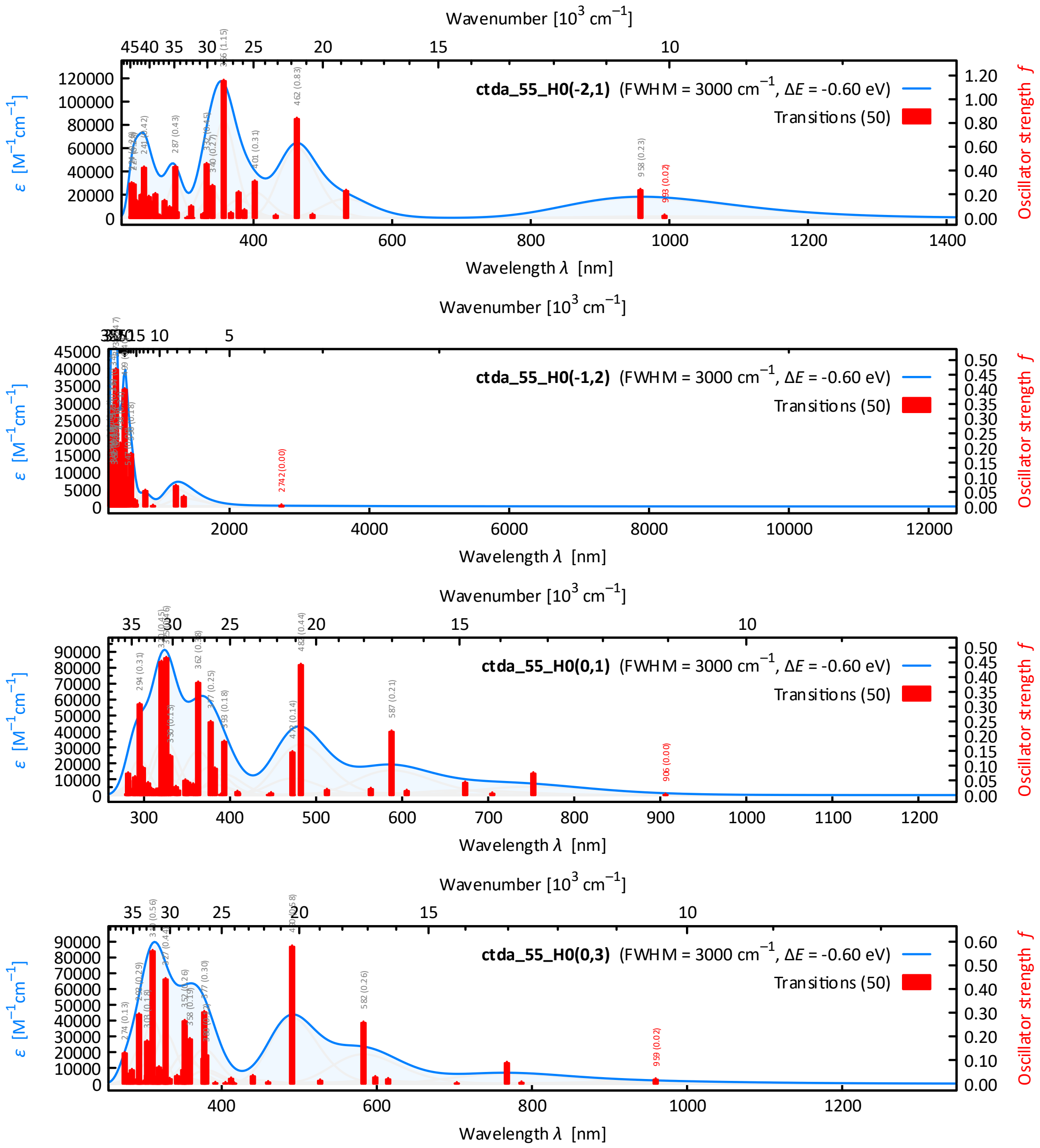

Figure S104. Calculated absorption spectra for the oxidation sequence of **[PTE55']**$^{2-}$ (cf. Figure S32 for the corresponding experimental data). Spectra were obtained using TDA calculations (PCM(THF)/CAM-B3LYP-GD3BJ/6-31G(d,p)//CAM-B3LYP-GD3BJ/6-31G(d,p), 50 transitions, energies shifted by 0.6 eV to lower values). Panel labels are of the form ctda_mn_Hx(charge,multiplicity), wherein mn is the fusion pattern, and Hx denotes the protonation status.

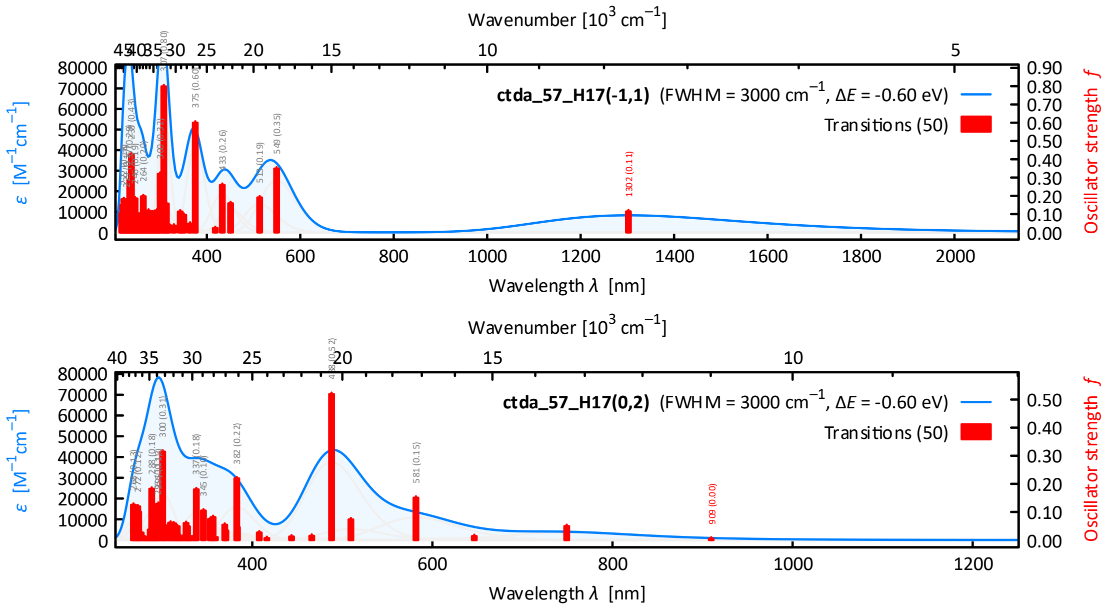

Figure S105. Calculated absorption spectra for the oxidation sequence of [**PTE57'**-H]$^{-}$. Spectra were obtained using TDA calculations (PCM(THF)/CAM-B3LYP-GD3BJ/6-31G(d,p)//CAM-B3LYP-GD3BJ/6-31G(d,p), 50 transitions, energies shifted by 0.6 eV to lower values). Panel labels are of the form ctda_mn_Hx(charge,multiplicity), wherein mn is the fusion pattern, and Hx denotes the protonation status.

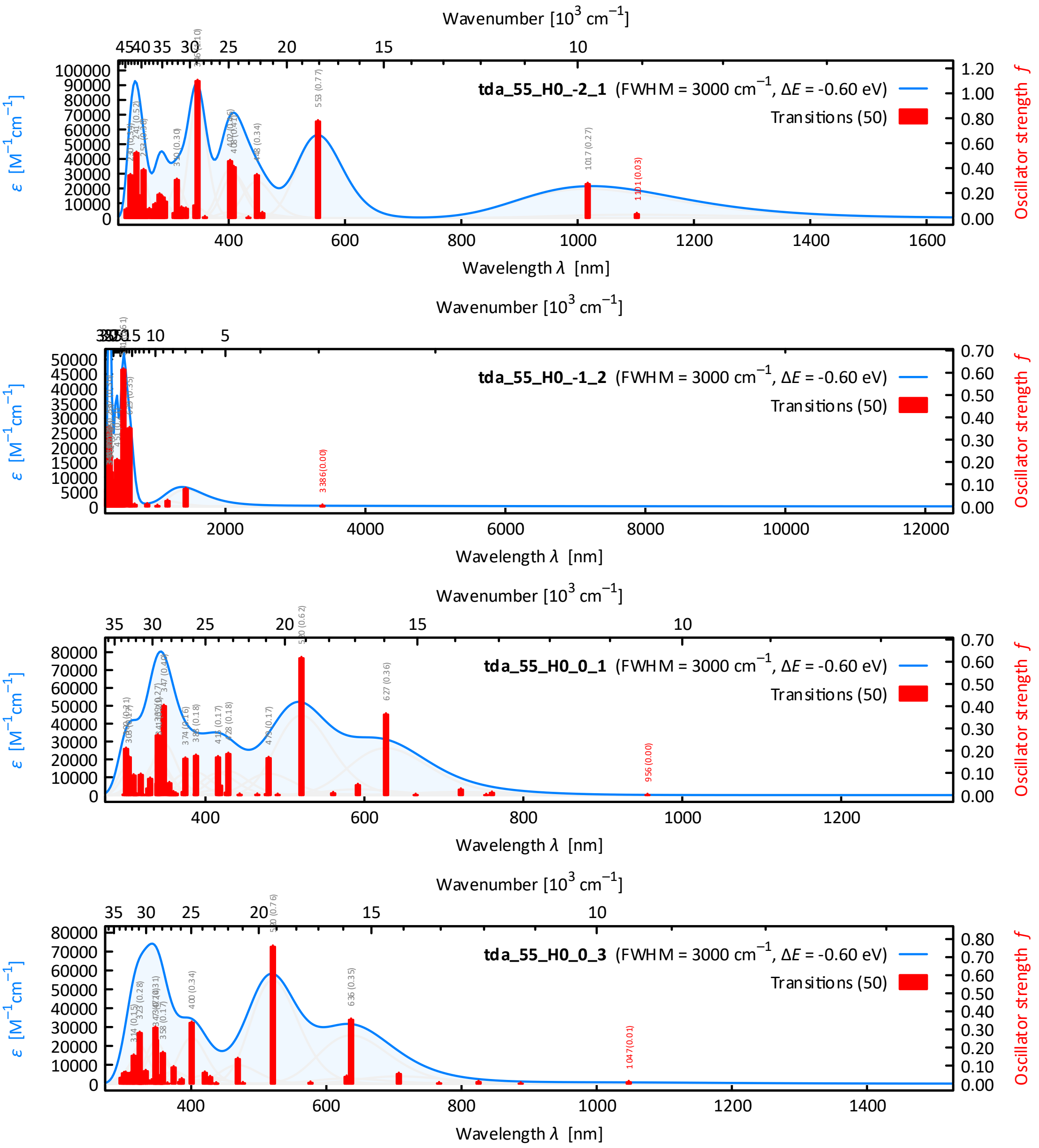

Figure S106. Calculated absorption spectra for the oxidation sequence of [**PDI55'**]$^{2-}$(cf. Figure S40 for the corresponding experimental data). Spectra were obtained using TDA calculations (PCM(THF)/CAM-B3LYP-GD3BJ/6-31G(d,p)//CAM-B3LYP-GD3BJ/6-31G(d,p), 50 transitions, energies shifted by 0.6 eV to lower values). Panel labels are of the form tda_mn_Hx_charge_multiplicity, wherein mn is the fusion pattern, and Hx denotes the protonation status.

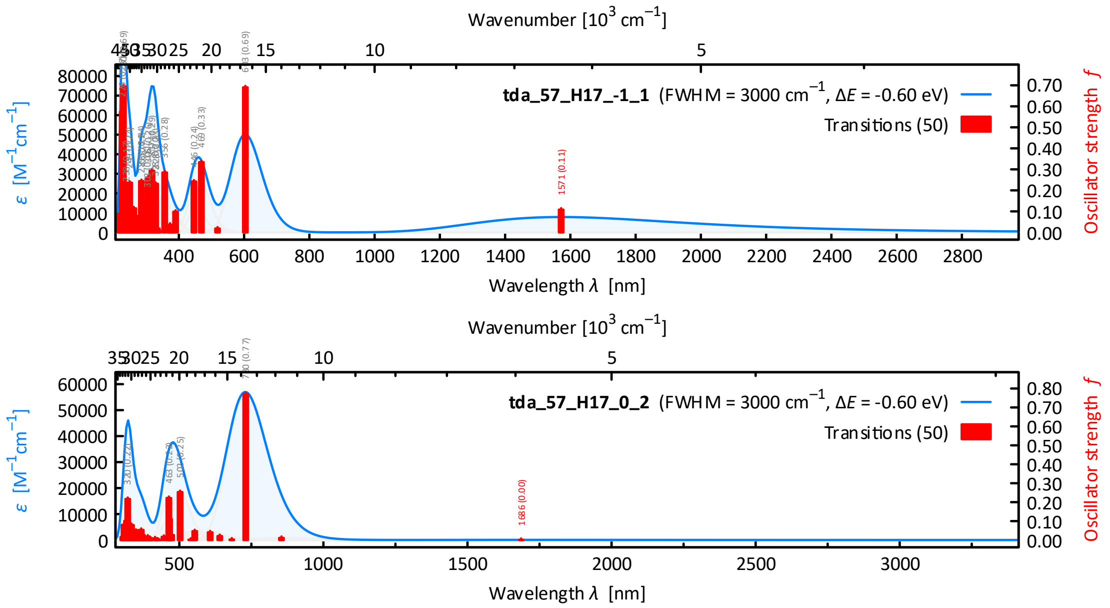

Figure S107. Calculated absorption spectra for the oxidation sequence [**PDI57'**-H]⁻ (cf. Figure S44 for the corresponding experimental data). Spectra were obtained using TDA calculations (PCM(THF)/CAM-B3LYP-GD3BJ/6-31G(d,p)//CAM-B3LYP-GD3BJ/6-31G(d,p), 50 transitions, energies shifted by 0.6 eV to lower values). Panel labels are of the form tda_mn_Hx_charge_multiplicity, wherein mn is the fusion pattern, and Hx denotes the protonation status.

5. References

(1) Wiencierz, A.; Lis, T.; Chmielewski, P. J.; Cybińska, J.; Stępień, M. Heptannulated Perylene Diimides: Formation and Reactivity of Electron-Deficient Tropylium Cations and Heptafulvenes. *Angew. Chem. Int. Ed.* **2025**, *64* (7), e202419899. https://doi.org/10.1002/anie.202419899.

(2) Fulmer, G. R.; Miller, A. J. M.; Sherden, N. H.; Gottlieb, H. E.; Nudelman, A.; Stoltz, B. M.; Bercaw, J. E.; Goldberg, K. I. NMR Chemical Shifts of Trace Impurities: Common Laboratory Solvents, Organics, and Gases in Deuterated Solvents Relevant to the Organometallic Chemist. *Organometallics* **2010**, *29* (9), 2176–2179. https://doi.org/10.1021/om100106e.

(3) Frisch, M. J.; Trucks, G. W.; Schlegel, H. B.; Scuseria, G. E.; Robb, M. A.; Cheeseman, J. R.; Scalmani, G.; Barone, V.; Petersson, G. A.; Nakatsuji, H.; Li, X.; Caricato, M.; Izmaylov, A. F.; Zheng, G.; Sonnenberg, J. L.; Hada, M.; Ehara, M.; Toyota, K.; Fukuda, R.; Hasegawa, J.; Ishida, M.; Nakajima, T.; Honda, Y.; Kitao, O.; Nakai, H.; Vreven, T.; Montgomery, Jr., J. A.; Peralta, J. E.; Ogliaro, F.; Bearpark, M.; Heyd, J. J.; Brothers, E.; Kudin, K. N.; Staroverov, V. N.; Kobayashi, R.; Normand, J.; Raghavachari, K.; Rendell, A.; Burant, J. C.; Iyengar, S. S.; Tomasi, J.; Cossi, M.; Millam, J. M.; Klene, M.; Adamo, C.; Gomperts, R.; Stratmann, R. E.; Yazyev, O.; Austin, A. J.; Cammi, R.; Pomelli, C.; Ochterski, J. W.; Martin, R. L.; Morokuma, K.; Zakrzewski, V. G.; Voth, G. A.; Salvador, P.; Dannenberg, J. J.; Dapprich, S.; Daniels, A. D.; Farkas, O.; Foresman, J. B.; Fox, D. J. Gaussian 16, Revision B.01, 2016.

(4) Becke, A. D. Density-Functional Exchange-Energy Approximation with Correct Asymptotic Behavior. *Phys. Rev. A* **1988**, *38* (6), 3098–3100.

(5) Becke, A. D. Density-functional Thermochemistry. III. The Role of Exact Exchange. *J. Chem. Phys.* **1993**, *98* (7), 5648–5652. https://doi.org/10.1063/1.464913.

(6) Lee, C.; Yang, W.; Parr, R. G. Development of the Colle-Salvetti Correlation-Energy Formula into a Functional of the Electron Density. *Phys. Rev. B* **1988**, *37* (2), 785–789. https://doi.org/10.1103/PhysRevB.37.785.

(7) Yanai, T.; Tew, D. P.; Handy, N. C. A New Hybrid Exchange–Correlation Functional Using the Coulomb-Attenuating Method (CAM-B3LYP). *Chem. Phys. Lett.* **2004**, *393* (1–3), 51–57. https://doi.org/10.1016/j.cplett.2004.06.011.

(8) Grimme, S.; Ehrlich, S.; Goerigk, L. Effect of the Damping Function in Dispersion Corrected Density Functional Theory. *J. Comput. Chem.* **2011**, *32* (7), 1456–1465. https://doi.org/10.1002/jcc.21759.

(9) Reed, A. E.; Weinstock, R. B.; Weinhold, F. Natural Population Analysis. *J. Chem. Phys.* **1985**, *83* (2), 735–746. https://doi.org/10.1063/1.449486.

(10) Shirman, E.; Ustinov, A.; Ben-Shitrit, N.; Weissman, H.; Iron, M. A.; Cohen, R.; Rybtchinski, B. Stable Aromatic Dianion in Water. *J. Phys. Chem. B* **2008**, *112* (30), 8855–8858. https://doi.org/10.1021/jp8029743.

(11) Schmidt, D.; Bialas, D.; Würthner, F. Ambient Stable Zwitterionic Perylene Bisimide-Centered Radical. *Angew. Chem. Int. Ed.* **2015**, *54* (12), 3611–3614. https://doi.org/10.1002/anie.201408067.

(12) Seifert, S.; Schmidt, D.; Würthner, F. An Ambient Stable Core-Substituted Perylene Bisimide Dianion: Isolation and Single Crystal Structure Analysis. *Chem. Sci.* **2015**, *6* (3), 1663–1667. https://doi.org/10.1039/C4SC03671A.

(13) Heitmüller, J.; Eckstein, K.; Renner, R.; Stolte, M.; Hertel, T.; Würthner, F.; Brixner, T. Coherent Two-Dimensional Electronic Spectroelectrochemistry. *Spectrochim. Acta. A. Mol. Biomol. Spectrosc.* **2021**, *253*, 119567. https://doi.org/10.1016/j.saa.2021.119567.

(14) Renner, R.; Stolte, M.; Heitmüller, J.; Brixner, T.; Lambert, C.; Würthner, F. Substituent-Dependent Absorption and Fluorescence Properties of Perylene Bisimide Radical Anions and Dianions. *Mater. Horiz.* **2022**, *9* (1), 350–359. https://doi.org/10.1039/D1MH01019K.

(15) Pearce, N.; Davies, E. S.; Champness, N. R. Electrochemical and Spectroelectrochemical Investigations of Perylene *Peri*-Tetracarbonyl Species. *Dyes Pigments* **2020**, *183*, 108735. https://doi.org/10.1016/j.dyepig.2020.108735.

(16) Niazimbetova, Z.; Treimer, S. E.; Evans, D. H.; Guzei, I.; Rheingold, A. L. Cathodically Promoted Addition of Nitromethane to Aldehydes and Exploratory Studies of the Mechanism of the Electrochemical Reduction of the Nitroolefins so Produced. *J. Electrochem. Soc.* **1998**, *145* (8), 2768. https://doi.org/10.1149/1.1838712.

(17) Sbei, N.; Rani, S.; Rahali, S.; Aslam, S.; Haq, Z. ul; Hardwick, T.; Ahmed, N. Greening Organic Electrochemistry: Harnessing Electrogenerated Bases (EGBs) for Sustainable Organic Transformations. *ACS Electrochem.* **2025**, *1* (12), 2648–2679. https://doi.org/10.1021/acselectrochem.5c00342.

(18) Li, H.; Wenger, O. S. Photophysics of Perylene Diimide Dianions and Their Application in Photoredox Catalysis. *Angew. Chem. Int. Ed.* **2022**, *61* (5), e202110491. https://doi.org/10.1002/anie.202110491.
